# Stakeholder Perspectives of Clinical Artificial Intelligence Implementation: Systematic Review of Qualitative Evidence

**DOI:** 10.2196/39742

**Published:** 2023-01-10

**Authors:** Henry David Jeffry Hogg, Mohaimen Al-Zubaidy, Technology Enhanced Macular Services Study Reference Group, James Talks, Alastair K Denniston, Christopher J Kelly, Johann Malawana, Chrysanthi Papoutsi, Marion Dawn Teare, Pearse A Keane, Fiona R Beyer, Gregory Maniatopoulos

**Affiliations:** 1 Population Health Science Institute Newcastle University Newcastle upon Tyne United Kingdom; 2 Newcastle upon Tyne Hospitals NHS Foundation Trust Newcastle upon Tyne United Kingdom; 3 Moorfields Eye Hospital NHS Foundation Trust London United Kingdom; 4 Institute of Inflammation and Ageing College of Medical and Dental Sciences University of Birmingham Birmingham United Kingdom; 5 University Hospitals Birmingham NHS Foundation Trust Birmingham United Kingdom; 6 Google Health Google London United Kingdom; 7 The Healthcare Leadership Academy London United Kingdom; 8 The Institute of Leadership and Management Birmingham United Kingdom; 9 Nuffield Department of Primary Healthcare Sciences Oxford University Oxford United Kingdom; 10 Institute of Ophthalmology University College London London United Kingdom; 11 Evidence Synthesis Group Population Health Science Institute Newcastle University Newcastle upon Tyne United Kingdom; 12 Faculty of Business and Law Northumbria University Newcastle upon Tyne United Kingdom

**Keywords:** artificial intelligence, systematic review, qualitative research, computerized decision support, qualitative evidence synthesis, implementation

## Abstract

**Background:**

The rhetoric surrounding clinical artificial intelligence (AI) often exaggerates its effect on real-world care. Limited understanding of the factors that influence its implementation can perpetuate this.

**Objective:**

In this qualitative systematic review, we aimed to identify key stakeholders, consolidate their perspectives on clinical AI implementation, and characterize the evidence gaps that future qualitative research should target.

**Methods:**

Ovid-MEDLINE, EBSCO-CINAHL, ACM Digital Library, Science Citation Index-Web of Science, and Scopus were searched for primary qualitative studies on individuals’ perspectives on any application of clinical AI worldwide (January 2014-April 2021). The definition of clinical AI includes both rule-based and machine learning–enabled or non–rule-based decision support tools. The language of the reports was not an exclusion criterion. Two independent reviewers performed title, abstract, and full-text screening with a third arbiter of disagreement. Two reviewers assigned the Joanna Briggs Institute 10-point checklist for qualitative research scores for each study. A single reviewer extracted free-text data relevant to clinical AI implementation, noting the stakeholders contributing to each excerpt. The best-fit framework synthesis used the Nonadoption, Abandonment, Scale-up, Spread, and Sustainability (NASSS) framework. To validate the data and improve accessibility, coauthors representing each emergent stakeholder group codeveloped summaries of the factors most relevant to their respective groups.

**Results:**

The initial search yielded 4437 deduplicated articles, with 111 (2.5%) eligible for inclusion (median Joanna Briggs Institute 10-point checklist for qualitative research score, 8/10). Five distinct stakeholder groups emerged from the data: health care professionals (HCPs), patients, carers and other members of the public, developers, health care managers and leaders, and regulators or policy makers, contributing 1204 (70%), 196 (11.4%), 133 (7.7%), 129 (7.5%), and 59 (3.4%) of 1721 eligible excerpts, respectively. All stakeholder groups independently identified a breadth of implementation factors, with each producing data that were mapped between 17 and 24 of the 27 adapted Nonadoption, Abandonment, Scale-up, Spread, and Sustainability subdomains. Most of the factors that stakeholders found influential in the implementation of rule-based clinical AI also applied to non–rule-based clinical AI, with the exception of intellectual property, regulation, and sociocultural attitudes.

**Conclusions:**

Clinical AI implementation is influenced by many interdependent factors, which are in turn influenced by at least 5 distinct stakeholder groups. This implies that effective research and practice of clinical AI implementation should consider multiple stakeholder perspectives. The current underrepresentation of perspectives from stakeholders other than HCPs in the literature may limit the anticipation and management of the factors that influence successful clinical AI implementation. Future research should not only widen the representation of tools and contexts in qualitative research but also specifically investigate the perspectives of all stakeholder HCPs and emerging aspects of non–rule-based clinical AI implementation.

**Trial Registration:**

PROSPERO (International Prospective Register of Systematic Reviews) CRD42021256005; https://www.crd.york.ac.uk/prospero/display_record.php?RecordID=256005

**International Registered Report Identifier (IRRID):**

RR2-10.2196/33145

## Introduction

### Background

Clinical artificial intelligence (AI) is a growing focus in academia, industry, and governments [1-3]. However, patients have benefited only in a few real-world contexts, reflecting a know-do gap called the “AI chasm” [4,5]. There is already evidence of tasks where health care professional (HCP) performance has been surpassed [6]. Reporting practices concerning quantitative measures of efficacy are also improving against evolving standards [7]. The rate-limiting step to patient benefit from clinical AI now seems to be real-world implementation [8]. This necessitates an understanding of how in real-world use, each technology may interact with the various configurations of policy-, organizational-, and practice-level factors [9,10]. Qualitative methods are best suited to produce evidence-based guidance to anticipate and manage implementation challenges; however, they remain rare in the clinical AI literature [1,11,12].

### Prior Work

Qualitative clinical AI literature was broadly synthesized until 2013 [13]. Despite accommodating eligibility criteria, the study synthesized 16% (9/56) of qualitative studies that were eligible, prioritizing only higher-quality articles for data extraction. All the 9 studied tools were based on electronic health care records to support various aspects of prescribing. All except 1 of the studies were set in the United States, and all applied rule-based decision logic preprogrammed by human experts. The main findings included usability concerns for HCPs, poor integration of the data used by tools with the workflows and platforms in which they were placed, the technical immaturity of tools and their host systems, and the fact that adopters had a variable perception of the AI tools’ value depending on their own experience [13]. Much of the subsequent clinical AI literature refers to machine learning or non–rule-based tools, which differ from rule-based tools in ways that may limit the understanding of the clinical, social, and ethical implications of their implementation [3]. An example of such a tool is a classification algorithm that distinguishes retinal photographs containing signs of diabetic retinopathy from those that do not [14]. The tool “learned” to do this in a relatively unexplainable fashion through exposure to a great quantity of retinal imaging data accompanied by human-expert labels of whether diabetic retinopathy was present. These non–rule-based tools promise broader applicability and higher performance than rule-based tools that automate established human clinical reasoning methods [3]. An example of a rule-based tool is one that applies an a priori decision tree determined by human clinical experts to produce individualized management recommendations for patients [15]. Despite the differences in their mechanisms, both tool groups satisfy the Organization for Economic Cooperation and Development’s definition of AI [16]. It is unclear whether the rule-based majority of the limited qualitative clinical AI evidence base is relevant to the modern focus on non–rule-based clinical AI [17]. However, as only 4 primary qualitative studies were identified across 2 recent syntheses of non–rule-based tools, it appears that broader eligibility criteria will be required to synthesize a meaningful volume of research at present [11,12]. Although primary qualitative clinical AI research is growing, its pace remains relatively slow. If the impact of this important work is to be maximized, clarity is required regarding which perspectives and factors that influence implementation remain inadequately explored [1].

### Goal of This Study

This qualitative evidence synthesis aimed to identify key stakeholder groups in clinical AI implementation and consolidate their published perspectives. This synthesis process aimed to maximize the accessibility and utility of published data for practitioners to support their efforts to implement various clinical AI tools and to complement their insight into the unique context that they target (Textbox 1). As a secondary aim, this synthesis aimed to improve the impact of future qualitative investigations of clinical AI implementation by recommending evidence-based research priorities.

**The research question, eligibility criteria informing a search strategy, and research databases that the search strategy was applied to on April 30, 2021 (Multimedia Appendix 1).**

- Research question
  What are the perspectives of stakeholders in clinical artificial intelligence (AI) and how can they inform its implementation?
- Participants
  Humans participating in primary research reporting free-text qualitative data
- Phenomena of interest
  Individuals’ perspectives of rule-based or non–rule-based clinical AI implementation
- Context
  Research from any real-world, simulated, or hypothetical health care setting worldwide, published between January 1, 2014, and April 30, 2021, in any language
- Databases searched
  Ovid-MEDLINE, EBSCO-CINAHL, ACM Digital Library, Science Citation Index-Web of Science, and Scopus

## Methods

### Overview

This qualitative evidence synthesis adhered to an a priori protocol, the Joanna Briggs Institute (JBI) guidance for conduct and ENTREQ (Enhancing Transparency in Reporting the Synthesis of Qualitative research) reporting guidance [18-20]. The best-fit framework synthesis method was selected using the RETREAT (Review Question-Epistemiology-Time or Timescale-Resources-Expertise-Audience and Purpose-Type of Data) criteria [21,22]. Following a review of implementation frameworks, the Nonadoption, Abandonment, Scale-up, Spread, and Sustainability (NASSS) framework was selected to accommodate the interacting complexity of factors and related stakeholders, which shape the implementation of health care technologies at the policy, organizational, and practice level [10]. The NASSS framework consists of seven domains, which categorize the factors that can influence implementation: (1) Condition, (2) Technology, (3) Value proposition, (4) Adopters, (5) Organization, (6) Wider context, (7) Embedding and adaptation over time [10]. In addition to its focus on technological innovations and its value in considering implementation factors between policy and practice levels, NASSS can be used as a determinant or evaluation framework rather than a process model, and it applies a relatively high level of theoretical abstraction [23]. This means that NASSS can readily accommodate perspectives from various stakeholders, contexts, and tools without enforcing excessive assumptions about the mechanisms of implementation, which is well-suited to the heterogeneous literature to be synthesized [24].

### Search Strategy and Selection Criteria

The research question and eligibility criteria informed a preplanned search strategy (available for all databases in Multimedia Appendix 1) that is designed with an experienced information specialist (FRB), informed by published qualitative and clinical AI search strategies and executed in 5 databases (Textbox 1) [6,11,13,25,26]. The search strings were designed in Ovid-MEDLINE and translated into EBSCO-CINAHL, ACM Digital Library, Science Citation Index-Web of Science, and Scopus. The exact terms used are available in Multimedia Appendix 1, but each string combined the same 3 distinct concepts of qualitative research, AI, and health care with *AND* Boolean operator terms. Differing thesaurus terms and search mechanisms between the databases demanded adaptation of the original search string, but each translation was aimed to reflect the original Ovid-MEDLINE version as closely as possible and was checked for sensitivity and specificity through pilot searches before the final execution. Studies concerning AI as a treatment, such as chatbots to provide talking therapies for mental health conditions, were not eligible as they represent an emerging minority of clinical AI applications [27]. They also evoke social and technological phenomena that are distinct from AI, providing clinical decision support, and therefore, risk diluting synthesized findings with nongeneralizable perspectives. The search strategy was reported in line with the PRISMA-S (Preferred Reporting Items for Systematic Reviews and Meta-Analyses literature search extension) [28]. Search results were pooled in Endnote (version 9.3.3; Clarivate Analytics) for deduplication and uploaded to Rayyan [29]. The references of any review or protocol studies returned were manually searched before exclusion along with all eligible study references. Potentially relevant missing data identified in the full-text reviews were pursued with up to 3 emails to the corresponding authors. Examples of such data included eligible protocols published ≥1 year previously without a follow-up report of the study itself or multimethod studies that appeared to report only quantitative data. Title, abstract, and full-text screening were fully duplicated by 2 independent reviewers (MA and HDJH) with a third arbiter of disagreement (GM; Multimedia Appendix 2). Eligible articles without full text in English were translated using an automated digital translation service between May and June 2021 (Google Translate). The validity of this approach in systematic reviews has been tested empirically and is applied routinely in quantitative and qualitative syntheses [30,31].

### Data Analysis

Characteristics and an overall JBI 10-point checklist for qualitative research score was assigned for each study and discussed by 2 reviewers (MA and HDJH) for 9.9% (11/111) of eligible studies [18]. The remaining 90.1% (100/111) were equally divided for the independent extraction of characteristics and assignment of the JBI 10-point checklist for qualitative research scores. Free-text data extraction using NVivo (Release 1.2; QSR International) was performed by a single reviewer (HDJH) following consensus exercises with 3 other authors (MA, GM, and FRB). Data were extracted in individual excerpts, which were determined to be continuous illustrations of a stakeholder’s perspective on clinical AI. A single reviewer (HDJH) assigned each excerpt a JBI 3-tiered level of credibility (Textbox 2) to complement the global appraisal of each study provided by the JBI 10-point checklist for qualitative research [18].

**Three-tiered Joanna Briggs Institute (JBI) credibility rating applied to each data excerpt, as described in the JBI Reviewers’ Manual The systematic review of qualitative data [18].**

- Unequivocal
  Findings accompanied by an illustration that is beyond reasonable doubt and, therefore, not open to challenge
- Credible
  Findings accompanied by an illustration lacking clear association with it and, therefore, open to challenge
- Not supported
  When neither 1 nor 2 apply and when most notably findings are not supported by the data

All perspectives relating to the phenomena of interest (Textbox 1) arising from participant quotations or authors’ narratives were extracted verbatim from the results and discussion sections. Each excerpt was attributed to the voice of an emergent stakeholder group and a single NASSS subdomain [10]. When the researcher (HDJH) extracting data felt that perspectives fell outside the NASSS subdomains, a draft subdomain was added to the framework to be later reviewed and reiterated with authors with varied perspectives as per the best-fit framework synthesis method [26]. A similar approach was applied to validate the stakeholder groupings which emerged. To permit greater granularity and meaning from the synthesis of such a large volume of data, inductive themes were also created within each NASSS subdomain. The initial data-led titles for these inductive themes were generated by the researcher extracting the data, making initial revisions as the data extraction proceeded. This was followed by several rounds of discussion with the coauthors to review and reiterate the inductive themes alongside their associated primary data to consolidate themes when appropriate and to maximize the accessibility and accuracy of their titles.

NASSS allows researchers to operationalize theory to find coherent sense in large and highly heterogeneous data such as those in this study. However, this may limit the accessibility of the analysis for some stakeholders, as it demands some familiarity with theoretical approaches [32]. To remove this barrier, the key implementation factors arising from the NASSS best-fit framework synthesis were delineated by their relevance for the 5 stakeholder groups that arose from the data. Coauthors with lived experience of each emergent stakeholder role were then invited to coproduce a narrative summary of the factors most relevant to their role. The initial step in this process was the provision of a longer draft of findings relating to each stakeholder group’s perspective by the lead reviewer (HDJH) before the review and initial discussion with each coauthor. This included a senior consultant ophthalmologist delivering and leading local services (SJT), a senior clinical academic working in clinical AI regulation and sitting on a committee advising the national government on regulatory reform (AKD), a clinical scientist working for an international MedTech company (CJK), the founder and managing director of The Healthcare Leadership Academy (JM), and a panel of 4 members of the public experienced in supporting research (reference group). In these 5 separate coproduction streams, the lead reviewer (HDJH) contributed their oversight of the data to discussions with each stakeholder representative (AKD, CJK, JM, SJT, and reference group), who gave feedback to prioritize and frame the data discussed. The lead reviewer then redrafted the section for further rounds of review and feedback until an agreement was reached. This second analytical step validated the findings, increased their accessibility, and aimed to support different stakeholders’ empathy for one another.

**Figure 1:**
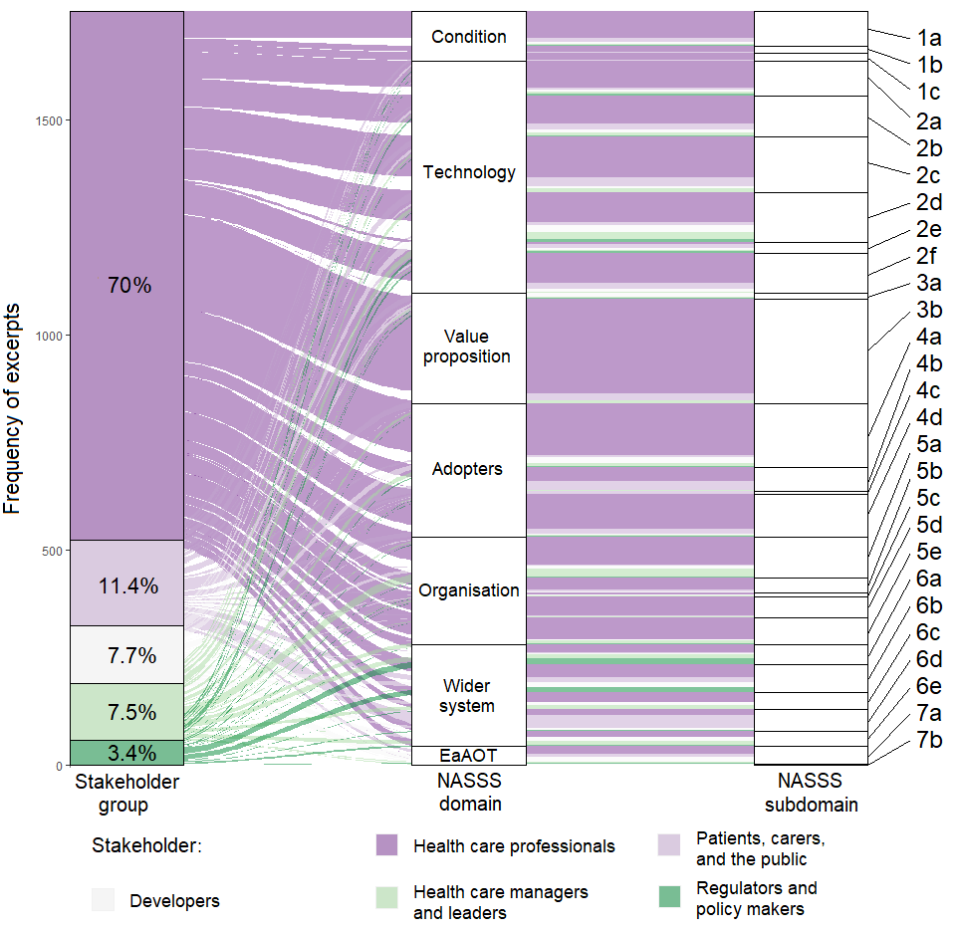
Sankey diagram illustrating the proportion of 1721 primary study excerpts derived from the voice of each of 5 emergent stakeholder groups and how each excerpt relates to each domain and subdomain of an adapted Non-adoption, Abandonment, Scale-up, Spread and Sustainability (NASSS) framework [19]. EaAOT: embedding and adaptation over time.

To preserve methodological rigor while pursuing broad accessibility, the results were presented for 3 levels of engagement. First, we used 5 stakeholder group narratives. Second, 63 inductive themes were distributed across the 27 subdomains of the adapted NASSS framework. The final most granular level of presentation used an internal referencing system within the *Results* section to link each assertion of the stakeholder group narratives with its supporting primary data and inductive theme (Multimedia Appendix 3 [33-143]). Notably, insights relevant to a given stakeholder group’s perspective were often contributed by study participants from different stakeholder groups (Figure 1 [19]). This is demonstrated by the selected excerpts contained within the 5 stakeholder group narratives, which are all followed by a brief description of the stakeholders who contributed to the excerpt.

## Results

### Overview

**Figure 2:**
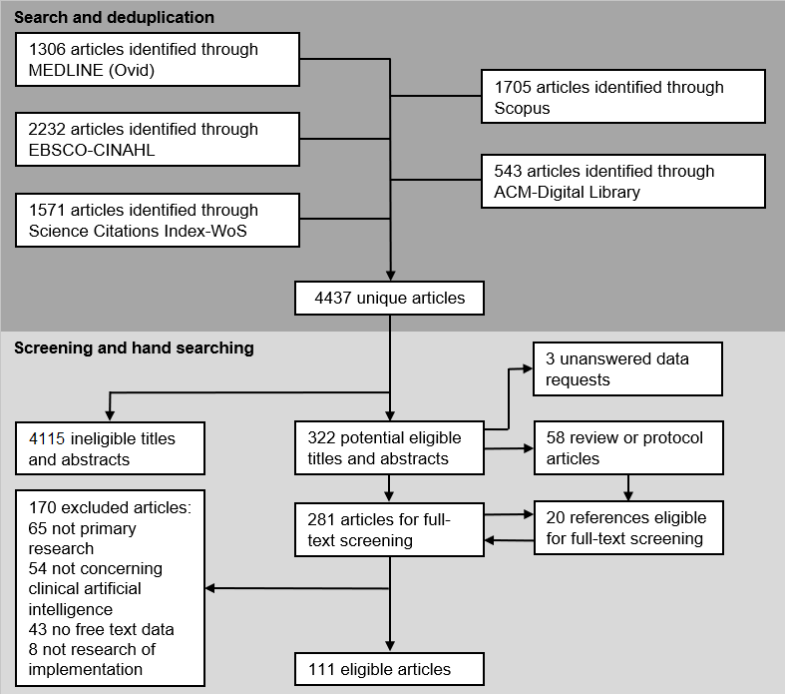
PRISMA (Preferred Reporting Items for Systematic Reviews and Meta-Analyses) style flowchart of search and eligibility check executions [39].

From an initial 4437 unique articles, 111 (2.5%) were found to be eligible, in which 2 (1.8%) were written in languages other than English [33,34] and the corresponding authors for 3 (2.7%) further studies [144-146], containing potentially relevant data, were not successfully contacted (Figure 2 [147]). Specific exclusion criteria were recorded for each excluded article at the full-text review stage (Multimedia Appendix 2), with most exclusions (4115/4326, 95.12%) made at the title and abstract screening stage. The absence of qualitative research methods was the most common cause of these exclusions. In the 111 eligible studies, there were 1721 excerpts. In assigning a JBI credibility score to each of these 1721 excerpts, 1155 (67.11%) were classified as unequivocal, 373 (21.67%) as equivocal, and 193 (11.21%) as unsupported [18]. The excerpts were categorized within the 27 subdomains of the adapted NASSS framework (Table 1) Inductive themes from within each NASSS subdomain are also listed along with the reference code applied throughout the results section and additional materials and the number of eligible primary studies which contributed.

Five distinct stakeholder groups emerged through the analysis, each contributing excerpts related to 17 to 24 of the 27 subdomains (Figure 1). Eligible studies (Table 2) represented 23 nations, with the United States, the United Kingdom, Canada, and Australia as the most common host nations, and 25 clinical specialties, with a clear dominant contribution from primary care (Multimedia Appendix 4 [33-143]). Although there was some representation from resource-limited nations, 88.2% (90/102) of the studies focusing on a single nation were in countries meeting the United Nations Development Programme’s definition of “very high human development” with a human development index between 0.8 and the upper limit of 1.0 [148]. The median human development index of the host nations for these 101 studies was 0.929 (IQR 0.926-0.944). The JBI 10-point checklist for qualitative research scores assigned to each study had a median of 8 (IQR 7-8) [18]. Detailed characteristics, including AI use cases, are available in Multimedia Appendix 4.

**Table 1:** Subdomains of the Nonadoption, Abandonment, Scale-up, Spread, and Sustainability (NASSS) framework used for data analysis with 2 data-led additions to the original subdomain list (n=111) [10].

NASSS subdomain and codes		Inductive theme	Papers, n (%)
**1a. Nature of condition or illness**			
	1a.1	Type or format of care needs	11 (9.9)
	1a.2	Ambiguous, complicated, or rare decisions	23 (20.7)
	1a.3	Quality of current care	18 (16.2)
	1a.4	Decision urgency and impact	11 (9.9)
**1b. Comorbidities**			
	1b.1	Other associated health problems	5 (4.5)
	1b.2	Aligning patient and health priorities	6 (5.4)
1c Sociocultural factors		No subthemes	13 (11.7)
**2a. Material properties**			
	2a.1	Usability of the tool	28 (25.2)
	2a.2	Lack of emotion	12 (10.8)
	2a.3	Large amounts of changing data	14 (12.6)
**2b. Knowledge to use it**			
	2b.1	Knowledge required of patients	24 (21.6)
	2b.2	Enabling users to evaluate tools	20 (18)
	2b.3	Agreeing the scope of use	19 (17.1)
**2c. Knowledge generated by it**			
	2c.1	Communicate meaning effectively	45 (40.5)
	2c.2	Target a clinical need	23 (20.7)
	2c.3	Recommend clear action	25 (22.5)
**2d. Supply model**			
	2d.1	Equipment and network requirements	23 (20.7)
	2d.2	Working across multiple health data systems	25 (22.5)
	2d.3	Quality of the health data and guidelines used	33 (29.7)
2e. Who owns the intellectual property?		No subthemes	14 (12.6)
**2f. Care pathway positioning^a^**			
	2f.1	Extent of tools’ independence	23 (20.7)
	2f.2	When and to whom the tool responds	21 (18.9)
	2f.3	How and where the tool responds	20 (18)
3a. Supply-side value (to developer)		No subthemes	7 (6.3)
**3b. Demand-side value (to patient)**			
	3b.1	Time required for service provision	27 (24.3)
	3b.2	Patient-centered care	22 (19.8)
	3b.3	Cost of health care	17 (15.3)
	3b.4	Impact on outcomes for patients	28 (25.2)
	3b.5	Educating and prompting HCPs^b^	41 (36.9)
	3b.6	Consistency and authority of care	33 (29.7)
**4a. Staff (role and identity)**			
	4a.1	Appetite and needs differ between staff groups	33 (29.7)
	4a.2	Tools redefine staff roles	33 (29.7)
	4a.3	Aligning with staff values	28 (25.2)
**4b. Patient (simple vs complex input)**			
	4b.1	Inconvenience for patients	10 (9.0)
	4b.2	Patients’ control over their care	14 (12.6)
	4b.3	Aligning patients’ agendas with tool use	11 (9.9)
4c. Carers		No subthemes	4 (3.6)
**4d. Relationships^a^**			
	4d.1	Patients’ relationships with their HCPs	30 (27)
	4d.2	Users’ relationships with tools	13 (11.7)
	4d.3	Relationships between health professionals	21 (18.9)
**5a. Capacity to innovate in general**			
	5a.1	Resources needed to deliver the benefits	29 (26.1)
	5a.2	Leadership	26 (23.4)
**5b. Readiness for this technology**			
	5b.1	Pressure to find a way of improving things	9 (8.1)
	5b.2	Suitability of hosts’ premises and technology	15 (13.5)
5c. Nature of adoption or funding decision		No subthemes	7 (6.3)
**5d. Extent of change needed to organizational routines**			
	5d.1	Fitting the tool within current practices	14 (12.6)
	5d.2	Change to intensity of work for staff	22 (19.8)
**5e. Work needed to plan, implement, and monitor change**			
	5e.1	Training requirements	17 (15.3)
	5e.2	Effort and resources for tool launch	23 (20.7)
**6a. Political or policy context**			
	6a.1	Different ways to incentivize providers	10 (9)
	6a.2	Importance of government strategy	8 (7.2)
	6a.3	Policy and practice influence each other more	15 (13.5)
**6b. Regulatory and legal issues**			
	6b.1	Impact on patient groups	19 (17.1)
	6b.2	Product assurance	14 (12.6)
	6b.3	Deciding who is responsible	8 (7.2)
**6c. Professional bodies**			
	6c.1	Resistance from professional culture	20 (18)
	6c.2	Lack of understanding between professional groups	9 (8.1)
**6d. Sociocultural context**			
	6d.1	Culture’s effect on tool acceptability	17 (15.3)
	6d.2	Public reaction to tools varies	10 (9)
6e. Interorganizational networking		No subthemes	14 (12.6)
**7a. Scope for adaptation over time**			
	7a.1	Normalization of technology and decreased resistance	15 (13.5)
	7a.2	Improvement of technology and its implementation	11 (9.9)
7b. Organizational resilience		No subthemes	3 (2.7)

^a^Indicates a subdomain added to the original NASSS framework through application of the best-fit framework synthesis method [21].

^b^HCP: health care professional.

**Table 2:** Characteristics of 111 eligible studies and the clinical artificial intelligence (AI) studied.

Characteristic		Studies, n (%)
**Clinical AI application**		
	Hypothetical	31 (27.9)
	Simulated	24 (21.6)
	Clinical	56 (50.5)
**Clinical AI nature**		
	Rule based	66 (59.5)
	Non–rule based	41 (36.9)
	NS^a^	4 (3.6)
**Clinical AI audience**		
	Public	5 (4.5)
	Primary care	45 (40.5)
	Secondary care	43 (38.7)
	Mixed	3 (2.7)
	NS	15 (13.5)
**Clinical AI input**		
	Numerical or categorical	83 (74.8)
	Imaging	9 (8.1)
	Mixed	1 (0.9)
**Clinical AI task**		
	Triage	15 (13.5)
	Diagnosis	15 (13.5)
	Prognosis	10 (9)
	Management	46 (41.4)
	NS	24 (21.6)
**Research method**		
	Interviews	54 (48.6)
	Focus groups	19 (17.1)
	Surveys	12 (10.8)
	Think aloud exercises	1 (0.9)
	Observation	1 (0.9)
	Mixed	24 (21.6)

^a^NS: not specified.

### Developers

The developers of clinical AI required both technical and clinical expertise alongside effective interaction within the multiple professional cultures that stakeholders inhabit (6e and 6c.2). This made cross-disciplinary work a priority, but it was challenged by the immediate demands of clinical duties that limited HCPs’ engagement (5a.1). State incentive systems for cross-disciplinary work had the potential to make this collaboration more attractive for developers (6a.2); nevertheless, those who independently prioritized multidisciplinary teams appeared to increase their innovations’ chances of real-world utility (2c.2). The instances when HCP time had been funded by industry or academia were highly valued (4a.3):

...she [an IT person with a clinical background] really bridges that gap...when IT folks talk directly to the front line, sometimes there’s just the language barrier there.

Unspecified professional [35]

To safeguard clinical AI utility, developers sometimes built in plasticity to accommodate variable host contexts (2a.3). This plasticity was beneficial both in terms of the clinical “reasoning” a tool applied and where and how it could be applied within different organizations’ or individuals’ practice (2e and 5d.1). The usability and accessibility of clinical AI often have a greater impact on adopter perceptions than their performance (2a.1 and 2b.1). There were many examples of clinical AI abandonment from adopters who had not fully understood a tool (2b.3 and 5e.1) or organizations that lacked the capacity or experience to effectively implement it (5e.2). Vendors who invested in training, troubleshooting, and implementation consultancy were often better received:

I’ve learned...that this closing the loop is what makes the sale...sometimes, we’re handed a package with the implementation science done.

Health care manager [36]

The poor interoperability of different systems has inhibited clinical AI scale-up (2d.2), but it has seemed to benefit electronic health care record providers, whose market dominance has driven the uptake of their own clinical AI tools (3a.1). Clinical AI developed inhouse, or by third parties, seemed to be at a competitive disadvantage (2d.1). Increasing market competition and political attention may lead to software or regulatory developments that indiscriminately enhance interoperability and disrupt this strategic issue (3a.1 and 7a). Developers were also affected by defensive attitudes from health care organizations and patients, many of whom distrust industry with access to the data on which clinical AI’s training depends (2d.3 and 2e):

For example, Alibaba is entering the health industry. But hospitals only allow Alibaba to access data of outpatients, not data of inpatients. They [the IT firms] cannot get the core data [continuous data of inpatients] from hospitals.

Policy maker [37]

### Health Care Professionals

The HCPs’ perspectives on clinical AI varied greatly (4a.1), but they commonly perceived value from clinical AI that facilitated clinical training (3b.5), reduced simple or repetitive tasks (3b.1 and 3b.2), improved patient outcomes (3b.4), or widened individuals’ scope of practice (4a.2). Despite these incentives, HCP adoption was often hampered by inadequate time to embed clinical AI in practice (5d.1), skepticism about its ability to inform clinical decisions (6c.1 and 2c.2), and uncertainty around its mechanics (2b.2). The “black box” effect associated with non–rule-based clinical AI prompted varied responses, with the burden of improvement placed on either the HCP to educate themselves or developers to produce more familiar metrics of efficacy and interpretability (2c.1 and 2b.2):

“When I bring on a test, I usually know what method it is. You tell me AI, and I have conceptually no idea.”... As a result, pathologists wanted to get a basic crash course in using AI...

HCP [38]

The HCP culture could be very influential in local clinical AI implementation (6c.1). Professional hierarchies were exposed and challenged through the interplay of clinical AI and professional roles and relationships (4d.3). Some experienced this as a “levelling-up” opportunity, favoring evidence over eminence-based medicine and nurturing more collaborative working environments (2d.3 and 3b.6). Others felt that their capabilities were being undervalued and even feared redundancy on occasion (4a.2):

The second benefit was the potential to use the deep learning system’s result to prove their own readings to on-site doctors. Several nurses expressed frustration with their assessments being undervalued or dismissed by physicians.

Authors’ representation of HCPs [39]

In some studies, HCPs felt that care provision improved both in terms of quality and reach (3b.1 and 3b.4). A virtuous cycle of engagement and value perception could develop, depending on where HCPs saw value and need in a given context (2c.2 and 2b.3). This was often when clinical AI aligned with familiar ways of working (5d.1), prompting or actioning things that HCPs knew but easily forgot (3b.5), and where the transfer of responsibility was gradual and HCP led (2f.1):

...to the physician, the algorithmic sorting constituted an extension of her own, and her experienced colleagues’ expertise...“I consider it a clinical judgement, which we made when we decided upon the thresholds”...

HCP [40]

### Health Care Managers and Leaders

Strong leadership at any level within health care organizations supported successful implementation (5a.2). Competing clinical demands and the scale of projects had the potential to disincentivize initial resource investments and jeopardize the implementation of clinical AI (5e.2). Resources committed to the clinical AI implementation held more than their intrinsic value, as they signaled to adopters that implementation was a priority and encouraged a positive workforce attitude (5b.2). A careful selection of clinical AI tools that seem likely to ultimately relieve workforce pressure may help managers to protect investment and adopter buy-in despite excessive clinical burdens (3b.1 and 5b.1). Stepwise or cyclical implementation of clinical AI were also advocated as a means of smoothing workflow changes and minimizing distractions from active projects:

I think that if you keep it simple, and maybe in a structured way if you could layer it, so that you know, for 2012 we are focusing on these five issues and in 2013 we’re focusing on these...over time you would introduce better prescribing.

Primary care leader [41]

The significant commitment required for effective implementation underlined the importance of judicious clinical AI selection and where, how, and for whom it would be applied (2f and 1a.3). A heuristic approach from managers’ knowledge of their staff characteristics (eg, age, training, and contract length) roughly informed a context-specific implementation strategy (4a.1). However, co-design with the adopters themselves better supported the alignment of local clinical AI values, staff priorities, and patient needs (4b.3 and 5d.1). There were examples of this process being rushed and heavy investments achieving little owing to misalignment of these aspects (2b.3 and 5a):

...due to shortage of capacity and resources in hospitals, business cases were often developed too quickly and procurements were made without adequate understanding of the problems needing to be addressed

Authors’ representation of health care managers [42]

HCPs sometimes developed negative relationships with clinical AI, which limited sustainability if issues were not identified or addressed (4d.2 and 4a.1). Just as clinical AI with the flexibility to be applied to different local workflows appeared to be better received by adopters, an influential factor for implementation was health care managers who were prepared to be flexible about which part of workflow was targeted (2f). Clinical AI implementation often revealed preexistent gaps between ideal and real-world care. Managers framed this as not only a problematic creation of necessary work but also helpful evidence to justify greater resourcing from policy makers or higher leadership (6a.3 and 5a.1). The need to consider staff well-being by managers was also illustrated, as clinical AI sometimes absorbed simple aspects of clinical work, increasing the concentration of intellectually or emotionally strenuous tasks within clinician workflows (2a.2, 1a.2, and 5d.2):

The problem with implementing digital technologies is that all too often, we fail to recognise or support the human effort necessary to bring them into use and keep them in use.

Authors’ representation of HCPs [43]

### Patients, Carers, and the Public

Concerns about the impact of clinical AI on HCP-patient interactions mainly came from the fear of HCP substitution (4d.1). These concerns seemed strongest within mental health and social care contexts, which were felt to demand a “human touch” (1a.1, 1c, and 2a.2). Patient-facing clinical AI, such as chronic disease self-management tools, was well received if they operated under close HCP oversight (2f.1 and 2f.2). The use of clinical AI as an adjunct for narrow and simplistic tasks was more prevalent (2f.1 and 1a.2), aiming to liberate HCPs’ attention to improve care quality or reach (3b.2). There were also examples of patient-facing clinical AI that appeared to better align patients and HCP agendas ahead of consultations, empowering patients to represent their wishes more effectively (4b.2 and 4c):

It is an advantage when reliable information can be sent to the patient, because GPs [General Practitioners] often have to use time to reassure patients that have read inappropriate information from unreliable sources.

HCP [44]

There was little evidence of research into carers’ perspectives. Available perspectives suggested that clinical AI could make health care decisions more transparent, helping carers to advocate for patients (4c). This could help anticipate and mitigate some of the reported patient inconveniences and anxieties associated with clinical AI (2b.1 and 4b.1):

One participant stated that the intervention needed to be “patient-centred”. “Including patients in the design phase” and “conducting focus groups for patients” were suggested to improve implementation of the eHealth intervention.

Unspecified participants [45]

Public perception of clinical AI was extremely variable, and with little personal experience, it was common to draw on hesitancy (6d.2 and 6d.1):

...many women, who had a negative or mixed view of the effect of AI in society, were unsure of why they felt this way...

Authors’ representation of public [46]

Popular media were often felt to play a key role in informing the public and to encourage expectations far removed from real-world health care (6d.1) However, in cases where clinical AI was endorsed by trusted HCPs overseeing their care, these issues did not appear problematic (6b.2).

### Regulators and Policy Makers

There was a perceived need for ongoing regulation of clinical AI and the contexts in which they are applied. This was both in terms of how tools are deployed to new sites (2b.3 and 5f.2) and how they may evolve through everyday practice (2a.3 and 7a.2). To make this evolution safe, stakeholders identified the need for long-term multistakeholder collaboration (6e). However, the data highlighted disincentives for this way of working, suggesting that there may be a need to enforce it (6a.2 and 6c.2). Stakeholders also raised issues around generalizability and bias for the populations they served, which were context specific and could evolve over time (6b.1). Otherwise, practitioners could gradually apply clinical AI to specific settings for which it was not appropriately trained or validated (2b.3). This “use case creep” described in the data further supported the perceived need for continual monitoring and evaluation of adopters’ interaction with clinical AI (6b):

...they reported use of the e-algo only when they were confused or had more difﬁcult cases. They did not feel the time required to use the e-algo warranted its use in the cases they perceived as routine or simple.

Authors’ representation of HCPs [15]

Stakeholders often felt that clinical AI increased the speed and strength of policy and practice’s influence over one another (6a.3). Many appreciated its improvement of care consistency across contexts and alignment of practices with guidelines (3b.6 and 2d.3). Others criticized it as an oversimplification (6c.1). An opportunity was seen for policy development to become more dynamic and evidence based (3b.4). Some envisaged this as an automated quality improvement cycle, whereas others anticipated complete overhauls of treatment paradigms (2f.1).

I could easily see us going to that payer and saying, “Well, our risk model...shows your patient population is higher risk. We need to do more intervention, so we need more money.”

Health care manager [36]

Anxiety over who would hold legal responsibility if clinical AI became dominant was common (6b.3). The litigative threat was even felt by individuals who avoided clinical AI use, as HCPs feared allegations of negligence for not using clinical AI (6b.3). Neither industry nor clinical professionals felt well placed to take on legal responsibility for clinical AI outcomes because they felt they only understood part of the whole (2b.2 and 6e). This was mainly presented as an educational issue rather than a consequence of transparency and explainability concerns (2b.2). Such high-stakes uncertainties appeared likely to perpetuate resistance from stakeholders (6c.1) although some data suggested that legislation could prompt adaptation to commercial and clinical practices that would reassure individual adopters (6b.2):

...physicians stated that they were not prepared (would not agree?) to be held criminally responsible if a medical error was made by an AI tool.

Authors’ representation of HCPs [47]

...content vendors clearly state that they do not practice medicine and therefore should not be liable...

Authors’ representation of developer [48]

## Discussion

### Principal Findings

These data highlight the breadth of the interdependent factors that influence the implementation of clinical AI. They also highlight the influence of at least 5 distinct stakeholder groups over each factor (Figure 1): developers, HCPs, health care managers and leaders, public stakeholders, and regulators and policy makers. It should be emphasized that most individuals belong to more than one stakeholder group simultaneously, and the clinical AI tool and context under consideration will transform the influence of any given implementation factor; thus, robust boundaries and weightings between different stakeholders are inevitably artificial. However, to provide a simplified overview, the common factors related to each stakeholder group’s perspective are summarized in Table 3.

**Table 3:** A summary of common factors influencing clinical artificial intelligence (AI) implementation from 5 different stakeholder perspectives.

Stakeholder group	Common factors influencing clinical AI implementation
Developers	Understanding clinical needs Producing clinical AI tools capable of adapting to clinical and organizational changes Safeguarding value in a dynamic and uncertain market
Health care professionals	Feeling able to make sense of clinical AI tools in the context of their own practice Accounting for changes to patient and professional relationships Managing disruption to current care pathways
Health care managers and leaders	Anticipating the resources required to enable implementation Engaging all adopters early in implementation Remaining reflexive and reactive throughout implementation
Patients, carers, and the public	Understanding what clinical AI will mean for access to health care professionals Gaining access into clinical decision-making Reconciling varied perceptions and experiences of clinical AI
Regulators and policy makers	Establishing mechanisms for the longitudinal monitoring of the clinical AI tool and implementation context Strengthening the bidirectional influence of policy and practice Achieving clarity over clinical and technical accountability

The strong representation of HCPs’ perspectives in the literature is an asset. However, the 30.04% (517/1721) of the excerpts from all other stakeholder perspectives clearly hold important but underexplored insights across all implementation factors (Figure 1), which should be prioritized in future research. The underrepresentation of certain stakeholders is partly masked by the need to group together the least represented stakeholders to permit meaningful synthesis, exemplified by the total of 0.35% (6/1721) of excerpts, which is related to the carer perspective. Failure to reform this clinician-centricity will limit the understanding and management of the inherent multistakeholder process of implementation. Encouragingly, the frequency at which specific factors arose in studies of rule-based and non–rule-based tools seemed largely comparable (Multimedia Appendix 5). This supports the use of the wider general clinical AI evidence base to inform non–rule-based tool implementation, which has been curated and characterized in this study to support future tool and context-specific implementation efforts in anticipating and managing a unique constellation of factors and stakeholders (Multimedia Appendix 3). This is caveated in more dominant areas of discussion for non–rule-based tools, such as intellectual property, regulation, and sociocultural attitudes, where further research specific to non–rule-based clinical AI is required.

### Comparison With Prior Work

This qualitative evidence synthesis has demonstrated that many implementation factors concerning early rule-based clinical AI tools continue to be influential [149]. However, the analysis and presentation of this work has prioritized enabling a varied readership to interpret data within their own context and experience rather than prescribing factors to be considered for a narrow range of clinical AI tools and contexts [24,32]. As a result, this study has consolidated a wider scope of research than previous work to synthesize findings that can support future implementation practice and research, considering a wide range of clinical AI tools and contexts. This approach may compromise the depth of support offered by this study relative to other syntheses for particular clinical specialties, clinical AI types, or stakeholder groups [11,12]. To maintain rigor while acknowledging the subjective value of eligible data, a systematic, transparent, and empirical approach has been adopted. This contrasts with narrative reviews in the literature, which provide valuable insights that draw more directly on the expertise of particular groups and collaborations but may not be easily generalized to diverse clinical AI tools [8,150].

### Limitations

First, some of this study’s findings are limited by the low representation of certain groups’ perspectives in eligible studies, which necessitated highly abstracted definitions of key stakeholders to facilitate meaningful synthesis. In addition to the example of carers mentioned previously, employees of academic and commercial institutions were both termed “developers.” A related second limitation of this study was the use of databases that focused on peer-reviewed literature. This search strategy is likely to have contributed to the low representation of non-HCP stakeholder groups, as peer-reviewed publications are a resource-intensive approach to dissemination that does not reward other stakeholders as closely as it does HCPs. Potential mitigation steps included the addition of social media or policy documents, but they were thought to be unfeasible for this study, given the extensive eligible literature returned by the broad search strategy applied [151]. Instead, a codevelopment step was added to the analysis process to reinforce the limited stakeholder perspectives that did arise from the search strategy with the coauthors’ lived experience. This was also valuable because it helped mitigate a further source of bias from factors relevant to given stakeholders that were often being described in the primary data by participants from different stakeholder groups. This is reflected in the sources of the sample excerpts interspersing the results section and by the 61 excerpts attributed to the patient (4b) or carer (4c) NASSS subdomains, 57% (35/61) were sourced from stakeholders outside the public, patients, and carer stakeholder group. In addition to mitigating these limitations, the codevelopment step of analysis was also intended to help improve the accessibility of implementation science within clinical AI, where theory-focused dogma often obscures the value for practitioners [32,152]. A third limitation is the likely underrepresentation of non-English language reports of studies, despite the English language limits only being applied through database indexing. Search strings devised in other languages or searches deployed in databases that focus on non-English literature could examine this potential limitation.

### Future Directions

The relatively short list of eligible qualitative studies derived from such broad eligibility criteria emphasizes the need for more primary qualitative research to explore the growing breadth of clinical AI tools and implementation contexts. Future primary qualitative studies should prioritize the perspectives of non-HCP stakeholders. Researchers may wish to couple the relevant data curated here (Multimedia Appendix 3) and a rationally selected theoretical approach to develop their sampling and data collection strategies [153]. Further exploration of implementation factors more pertinent to non–rule-based tools, such as intellectual property, regulation, and sociocultural attitudes, may also improve the literature’s contemporary relevance.

### Conclusions

This study has consolidated multistakeholder perspectives of clinical AI implementation in an accessible format that can inform clinical AI development and implementation strategies involving varied tools and contexts. It also demonstrates the need for more qualitative research on clinical AI, which more adequately represents the perspectives of the many stakeholders who influence its implementation and the emerging aspects of non–rule-based clinical AI implementation.

## Appendix

Multimedia Appendix 1 Search strategies.

Multimedia Appendix 2 Excluded studies with potentially eligible abstracts.

Multimedia Appendix 3 Codebook.

Multimedia Appendix 4 Eligible primary articles and their characteristics.

Multimedia Appendix 5 Excerpts by clinical artificial intelligence type.

## Abbreviations

AI: artificial intelligence
ENTREQ: Enhancing Transparency in Reporting the Synthesis of Qualitative research
HCP: health care professional
JBI: Joanna Briggs Institute
NASSS: Nonadoption, Abandonment, Scale-up, Spread, and Sustainability
PRISMA-S: Preferred Reporting Items for Systematic Reviews and Meta-Analyses literature search extension
RETREAT: Review Question-Epistemiology-Time or Timescale-Resources-Expertise-Audience and Purpose-Type of Data

## Data Availability

All free-text data are available in Multimedia Appendix 3 and curated using the adapted Nonadoption, Abandonment, Scale-up, Spread, and Sustainability framework detailed in Table 1. Eligible studies and their characteristics are also available in Multimedia Appendix 4.
