# Supplementary material for: Stakeholder Perspectives of Clinical Artificial Intelligence Implementation: Systematic Review of Qualitative Evidence
Source: J Med Internet Res. 2023 Jan 10;25:e39742. doi: 10.2196/39742 (PMC9875023; doi:10.2196/39742)
Supplement: Multimedia Appendix 2 [file jmir_v25i1e39742_app2.pdf]

Authors	Year	Source	Title	Exclusion criterion
Ahmed, F.	2018	Proceedings of the National Academy of Sciences of the United	QnAs with Donald Geman	Not research of implementation
Anderson, M. and Anderson, S. L.	2019	AMA Journal of Ethics	How Should AI Be Developed, Validated, and Implemented in Patient Care?	Not primary research
Ash, J. S. and Chase, D. and Wiesen, J. F. and Murphy, E. V. and Marovich, S.	2016	AMIA ... Annual Symposium proceedings. AMIA Symposium	Studying Readiness for Clinical Decision Support for Worker Health Using the Rapid Assessment Process and Mixed Methods Interviews	No free text data
Beitz, Janice M. and Gerlach, Mary A. and Schafer, Vickie	2014	Journal of Wound, Ostomy & Continence Nursing	Construct Validation of an Interactive Digital Algorithm for Ostomy Care	No free text data
Bonfils, Kelsey A. and Dreison, Kimberly C. and Luther, Lauren and Sadaaki, Fukui a	2018	Psychiatric Rehabilitation Journal	Implementing CommonGround in a Community Mental Health Center: Lessons in a Computerized Decision Support System	Does not satisfy OECD definition of artificial intelligence
Campbell, J. P. and Mathenge, C. and Cherwek, H. and Balaskas, K. and Pasquale, L	2021	Translational Vision Science and Technology	Artificial intelligence to reduce ocular health disparities: Moving from concept to implementation	Not primary research
Chokshi, S. K. and Troxel, A. and Belli, H. and Schwartz, J. and Blecker, S. and Blaum	2019	Studies in health technology and informatics	User-centered development of a behavioral economics inspired electronic health record clinical decision support module C3 - Studies in He	No free text data
Cresswell, Kathrin M. and Lee, Lisa and Mozaffar, Hajar and Williams, Robin and Sh	2017	Health Services Research	Sustained User Engagement in Health Information Technology: The Long Road from Implementation to System Optimization of Computer	Does not satisfy OECD definition of artificial intelligence
Devine, Emily Beth and Lee, Chia-Ju and Overby, Casey L. and Abernethy, Neil and I	2014	International Journal of Medical Informatics	Usability evaluation of pharmacogenomics clinical decision support aids and clinical knowledge resources in a computerized provider orde	Does not satisfy OECD definition of artificial intelligence
Drake, Emily and Howard, Erica and Kinsey, Emily	2014	Community Mental Health Journal	Online Screening and Referral for Postpartum Depression: An Exploratory Study	Does not satisfy OECD definition of artificial intelligence
Eden, K. B. and Ivley, I. and Bensching, K. L. and Franta, G. and Hersh, A. R. and Cas	2020	JOURNAL OF WOMENS HEALTH	Use of an Online Breast Cancer Risk Assessment and Patient Decision Aid in Primary Care Practices	Does not satisfy OECD definition of artificial intelligence
Gardner, C. and Moseley, G. L. and Karran, E. L. and Wiles, L. K. and Hibbert, P.	2020	CANADIAN JOURNAL OF PAIN-REVUE CANADIENNE DE LA DOU	Implementing high value back pain care in private physiotherapy in Australia: A qualitative evaluation of physiotherapists who participate	Does not satisfy OECD definition of artificial intelligence
Gil, M. and Pinto, P. and Simoes, A. S. and Povoa, P. and Da Silva, M. M. and Lapao	2016	EXPLORING COMPLEXITY IN HEALTH: AN INTERDISCIPLINARY S	Co-Design of a Computer-Assisted Medical Decision Support System to Manage Antibiotic Prescription in an ICU Ward	No free text data
Habli, I. and Lawton, T. and Porter, Z.	2020	Bulletin of the World Health Organization	Artificial intelligence in health care: Accountability and safety	Not primary research
Hardy, Seth M.	2019	Journal of the American College of Radiology	The Economic Logic for Clinical Decision Support Is Changing	Not primary research
Hoffmann, M. and V and er Stichele, R. and Bates, D. W. and Bjorklund, J. and Alex	2020	Expert Review of Clinical Pharmacology	Guiding principles for the use of knowledge bases and real-world data in clinical decision support systems: report by an international expe	Not primary research
Jones, B. E. and Collingridge, D. S. and Vines, C. G. and Post, H. and Holmen, J. and	2019	Applied Clinical Informatics	CDS in a Learning Health Care System: Identifying Physicians' Reasons for Rejection of Best-Practice Recommendations in Pneumonia thro	No free text data
Jones, Stephen and Cournane, Sean and Sheehy, Niall and Hederman, Lucy	2016	Journal of Digital Imaging	A Business Analytics Software Tool for Monitoring and Predicting Radiology Throughput Performance	No free text data
Kohli, M. and Alkasab, T. and Wang, K. and Heilbrun, M. E. and Fl and ers, A. E. and	2019	Journal of the American College of Radiology	Bending the Artificial Intelligence Curve for Radiology: Informatics Tools From ACR and RSNA	Not primary research
Kumar, A. A. and Burgard, M. and Stacey, S. and S and aradura, I. and Lai, T. and Cc	2019	British Journal of Clinical Pharmacology	An evaluation of the user-friendliness of Bayesian forecasting programs in a clinical setting	No free text data
Lenert, L. and Dunlea, R. and Del Fiol, G. and Hall, L. K.	2014	MEDICAL DECISION MAKING	A Model to Support Shared Decision Making in Electronic Health Records Systems	No free text data
Lown, M. and Yue, A. and Lewith, G. and Little, P. and Moore, M.	2017	BMJ Open	Screening for Atrial Fibrillation using Economical and accurate TechnologY (SAFETY)-a pilot study	No free text data
Magrabi, F. and Ammenwerth, E. and McNair, J. B. and De Keizer, N. F. and Hyppor	2019	Yearbook of medical informatics	Artificial Intelligence in Clinical Decision Support: Challenges for Evaluating AI and Practical Implications	Not primary research
Mahabee-Gittens, E. M. and Dexheimer, J. W. and Gordon, J. S.	2016	CIN-COMPUTERS INFORMATICS NURSING	Development of a Tobacco Cessation Clinical Decision Support System for Pediatric Emergency Nurses	Not research of implementation
Mahabee-Gittens, E. M. and Dexheimer, J. W. and Tabangin, M. and Khoury, J. C. a	2018	American Journal of Preventive Medicine	An Electronic Health RecordÃ¢Ë†â€™Based Strategy to Address Child Tobacco Smoke Exposure	No free text data
Omar, A. and Ellenius, J. and Lindemalm, S. and Lau, F. and BartleClar, J. and Bliss,	2017	BUILDING CAPACITY FOR HEALTH INFORMATICS IN THE FUTUR	Evaluation of Electronic Prescribing Decision Support System at a Tertiary Care Pediatric Hospital: The User Acceptance Perspective	Does not satisfy OECD definition of artificial intelligence
Ongena, Y. P. and Haan, M. and Yakar, D. and Kwee, T. C.	2020	European Radiology	PatientsÃ¢â‚¬â„¢ views on the implementation of artificial intelligence in radiology: development and validation of a standardized questio	No free text data
Osterhoff, G., Pforringer, D., Scherer, J., Juhra, C., Maerdian, S., Back, D. A. and Arb	2020	UNFALLCHIRURG	Computer-assisted decision-making for trauma patients	Not primary research
Paton, C. and Kobayashi, S.	2019	Yearbook of medical informatics	An Open Science Approach to Artificial Intelligence in Healthcare	Not primary research
Petersen, C.	2018	Yearbook of medical informatics	Through Patients' Eyes: Regulation, Technology, Privacy, and the Future	Not primary research
Piette, J. D. and List, J. and Rana, G. K. and Townsend, W. and Striplin, D. and Heisl	2015	Circulation	Mobile Health Devices as Tools for Worldwide Cardiovascular Risk Reduction and Disease Management	Not primary research
Poon, Sabrina J. and Greenwood-Ericksen, Margaret B. and Gish, Rebecca E. and Ne	2016	Academic Emergency Medicine	Usability of the Massachusetts Prescription Drug Monitoring Program in the Emergency Department: A Mixed-methods Study	Does not satisfy OECD definition of artificial intelligence
Rauter, C. M. and Wohlke, S. and Schicktanz, S.	2021	Journal of Medical Systems	My Data, My Choice? - German Patient Organizations' Attitudes towards Big Data-Driven Approaches in Personalized Medicine. An Empir	Does not satisfy OECD definition of artificial intelligence
Rockall, A.	2020	Clinical Radiology	From hype to hope to hard work: developing responsible AI for radiology	Not primary research
Schnall, R. and Rojas, M. and Bakken, S. and Brown, W. and Carballo-Dieguez, A. ar	2016	Journal of Biomedical Informatics	A user-centered model for designing consumer mobile health (mHealth) applications (apps)	Does not satisfy OECD definition of artificial intelligence
Shrestha, Roman and Karki, Pramila and Copenhaver, Michael	2017	AIDS Care	Interest in use of mHealth technology in HIV prevention and associated factors among high-risk drug users enrolled in methadone mainte	No free text data
Smaradottir, B. and Martinez, S. and Borycki, E. and Loudon, G. and Kushniruk, A. a	2018	BUILDING CONTINENTS OF KNOWLEDGE IN OCEANS OF DATA:	User Evaluation of a Smartphone Application for Anticoagulation Therapy	No free text data
Stanfill, M. H. and Marc, D. T.	2019	Yearbook of medical informatics	Health Information Management: Implications of Artificial Intelligence on Healthcare Data and Information Management	Not primary research
Sukums, F. and Mensah, N. and Mpembeni, R. and Kaltschmidt, J. and Haefeli, W. I	2014	Glob Health Action	Health workers' knowledge of and attitudes towards computer applications in rural African health facilities	Not research of implementation
Taft, T. and Nelsen, S. D. and Slager, S. and Weir, C.	2017	AMIA ... Annual Symposium Proceedings/AMIA Symposium	Physician Information Needs in Managing Delirium	Does not satisfy OECD definition of artificial intelligence
Yassine, H. M. and Shah, Z. B.	2020	EXPERT REVIEW OF ANTI-INFECTIVE THERAPY	How could artificial intelligence aid in the fight against coronavirus? An interview with Dr Hadi M Yassine and Dr Zubair Shah by Felicity Po	Not research of implementation
Yoldemir, Tevfik	2020	Climacteric	Artificial intelligence and women's health	Not primary research
Aggarwal, H. K. and Jacob, M.	2020	Proc IEEE Int Symp Biomed Imaging.	Joint Optimization of Sampling Pattern and Priors in Model Based Deep Learning C3 - Proceedings - International Symposium on Biomedic	No free text data
Ahmad, O. F. and Mori, Y. and Misawa, M. and Kudo, S. E. and Anderson, J. T. and	2021	ENDOSCOPY	Establishing key research questions for the implementation of artificial intelligence in colonoscopy: a modified Delphi method	Not primary research
Ahmad, O. F. and Stoyanov, D. and Lovat, L. B.	2020	Techniques in Gastrointestinal Endoscopy	Barriers and pitfalls for artificial intelligence in gastroenterology: Ethical and regulatory issues	Not primary research
Akhtar, A. and Burgi, E.	2021	COMMUNICATIONS BIOLOGY	At the intersection of machine learning, biology, and health: an interview with Lorin Crawford	Not primary research
Alfarghaly, O. and Khaled, R. and Elkorany, A. and Helal, M. and Fahmy, A.	2021	Informatics in Medicine Unlocked	Automated radiology report generation using conditioned transformers	No free text data
Alvarez, Carmen and Debnam, Katrina and Clough, Amber and Alex and er, Kamila	2018	Research in Nursing & Health	Responding to intimate partner violence: Healthcare providers' current practices and views on integrating a safety decision aid into primai	Does not satisfy OECD definition of artificial intelligence
Amann, J. and Blasimme, A. and Vayena, E. and Frey, D. and Madai, V. I. and the Pi	2020	BMC Medical Informatics and Decision Making	Explainability for artificial intelligence in healthcare: a multidisciplinary perspective	No free text data
Andersson Hagiwara, M. and Lundberg, L. and Sjoqvist, B. A. and Maurin Soderholr	2019	Journal of Healthcare Informatics Research	The Effects of Integrated IT Support on the Prehospital Stroke Process: Results from a Realistic Experiment	Does not satisfy OECD definition of artificial intelligence
Barnett, A. and Savic, M. and Pienaar, K. and Carter, A. and Warren, N. and S and ra	2020	International Journal of Drug Policy	Enacting a more-than-human care: Clients and counsellors views on the multiple affordances of chatbots in alcohol and other drug counse	Does not satisfy OECD definition of artificial intelligence
Bashir, M. and Harky, A.	2019	Seminars in Thoracic & Cardiovascular Surgery	Artificial Intelligence in Aortic Surgery: The Rise of the Machine	Not primary research
Beaudelot, C. and Bayart, E. and Thariat, J. and Bourgier, C. and Denis, F. and Hatt,	2020	CANCER RADIOTHERAPIE	Radiation-oncology horizon 2030: From microbiota to plasma laser	Does not satisfy OECD definition of artificial intelligence
Bell, H. and Garfield, S. and Khosla, S. and Patel, C. and Franklin, B. D.	2018	EUROPEAN JOURNAL OF HOSPITAL PHARMACY-SCIENCE AND I	Mixed methods study of medication-related decision support alerts experienced during electronic prescribing for inpatients at an English I	Does not satisfy OECD definition of artificial intelligence
Belle, Ashwin and Thiagarajan, Raghuram and Soroushmehr, S. M. Reza and Navidi	2015	BioMed Research International	Big Data Analytics in Healthcare	Not primary research
Benrimoh, D. and Tanguay-Sela, M. and Perlman, K. and Israel, S. and Mehltretter,	2021	BJPSYCH OPEN	Using a simulation centre to evaluate preliminary acceptability and impact of an artificial intelligence-powered clinical decision support sys	No free text data
Bhavnani, S. P. and Parakh, K. and Atreja, A. and Druz, R. and Graham, G. N. and Ha	2017	Journal of the American College of Cardiology	2017 Roadmap for Innovation-ACC Health Policy Statement on Healthcare Transformation in the Era of Digital Health, Big Data, and Preci	Not primary research
Bian, Y. Y. and Xiang, Y. B. and Tong, B. D. and Feng, B. and Weng, X. S.	2020	JOURNAL OF MEDICAL INTERNET RESEARCH	Artificial Intelligence-Assisted System in Postoperative Follow-up of Orthopedic Patients: Exploratory Quantitative and Qualitative Study	Does not satisfy OECD definition of artificial intelligence
Blease, C. and Kharko, A. and Locher, C. and DesRoches, C. M. and M and l, K. D.	2020	PLoS ONE [Electronic Resource]	US primary care in 2029: A Delphi survey on the impact of machine learning	Not research of implementation
Blum, A. and Zins, M.	2017	DIAGNOSTIC AND INTERVENTIONAL IMAGING	Radiology: Is its future bright?	Not primary research
Boag, William and Kane, Hassan and Rawat, Saumya and Wei, Jesse and Goehler, A	2021	Proceedings of the 2021 ACM Conference on Fairness, Account	A Pilot Study in Surveying Clinical Judgments to Evaluate Radiology Report Generation	Not research of implementation
Brennan, M. and Puri, S. and Ozrazgat-Baslanti, T. and Feng, Z. and Ruppert, M. an	2019	Surgery (United States)	Comparing clinical judgment with the MySurgeryRisk algorithm for preoperative risk assessment: A pilot usability study	No free text data
Bridge, Pete and Fielding, Andrew and Rowntree, Pamela and Pullar, Andrew	2017	Journal of Medical Imaging & Radiation Sciences	Qualitative Evaluation of a Novel 3D Volumetric Radiotherapy Segmentation Tool	Does not satisfy OECD definition of artificial intelligence
Brown, C. H.	2020	ADMINISTRATION AND POLICY IN MENTAL HEALTH AND MENT	Three Flavorings for a Soup to Cure what Ails Mental Health Services	Not primary research
Cakir, E. Y. and Ince, G.	2019	4th International Conference on Computer Science and Enginee	Diagnophone: An Electronic Stethoscope for Respiratory Audio Analysis	No free text data
Caponnetto, P. and Milazzo, M.	2019	Health Psychology Research	Cyber health psychology: The use of new technologies at the service of psychologycal well being and health empowerment	Not primary research
Careyva, Beth A. and Hamadani, Roya and Friel, Timothy and Coyne, Cathy A.	2018	Journal of Community Health	A Social Needs Assessment Tool for an Urban Latino Population	Does not satisfy OECD definition of artificial intelligence
Castiglioni, I. and Rundo, L. and Codari, M. and Di Leo, G. and Salvatore, C. and Inte	2021	Physica Medica	AI applications to medical images: From machine learning to deep learning	Not primary research
Chan, Lili and Vaid, Akhil and Nadkarni, Girish N.	2020	Current Opinion in Nephrology & Hypertension	Applications of machine learning methods in kidney disease: hope or hype?	Not primary research
Chorev, Nadav Even	2019	Health Informatics Journal	Data ambiguity and clinical decision making: A qualitative case study of the use of predictive information technologies in a personalized ca	Does not satisfy OECD definition of artificial intelligence
Colling, R. and Pitman, H. and Oien, K. and Rajpoot, N. and Macklin, P. and Group,	2019	Journal of Pathology	Artificial intelligence in digital pathology: a roadmap to routine use in clinical practice	Not primary research
Curtis, Katherine and Brooks, Sheila	2020	Nursing Older People	Digital health technology: factors affecting implementation in nursing homes	Does not satisfy OECD definition of artificial intelligence
Dias Ferreira, Andressa Martins and Campos de Oliveira, Joao Lucas and Suzini Carr	2019	Revista Gaucha de Enfermagem	Perceptions of nursing professionals about the use of patient safety computerization	Does not satisfy OECD definition of artificial intelligence
Dilsizian, S. E. and Siegel, E. L.	2014	Current Cardiology Reports	Artificial intelligence in medicine and cardiac imaging: harnessing big data and advanced computing to provide personalized medical diagr	Not primary research
Dubuc, Nicole and Delli-Colli, Nathalie and Bonin, Lucie and Corbin, Cinthia and Lab	2016	International Journal of Integrated Care (IJIC)	Development of a Computerized Integrated-Care-Pathway System to Support People-Centred and Integrated Care: Usefulness of the Parti	Does not satisfy OECD definition of artificial intelligence
Eyler Dang, L. and Hubbard, A. and Dissak-Delon, F. N. and Chichom Mefire, A. and	2021	Surgery (United States)	Right population, right resources, right algorithm: Using machine learning efficiently and effectively in surgical systems where data are a li	Not primary research
Fidelis de Mesquita, Keysse Suelen and dos Santos Albuquerque, Maria Ci-cera and	2016	Journal of Nursing UFPE	ALGORITHM CONSTRUCTION ON PSYCHIC SUFFERING FOR DECISION MAKING OF NURSES OF PRIMARY CARE	Does not satisfy OECD definition of artificial intelligence
Fleming, Theresa and Lucassen, Mathijs and Stasiak, Karolina and Shepherd, Matth	2016	Clinical Psychologist	The impact and utility of computerised therapy for educationally alienated teenagers: The views of adolescents who participated in an alte	Does not satisfy OECD definition of artificial intelligence
Fomin, A. and Turov, M. and Matrosova, E. and Tikhomirova, A. and Samsonovich,	2018	BIOLOGICALLY INSPIRED COGNITIVE ARCHITECTURES (BICA) FC	Medical Knowledge-Based Decision Support System	Not primary research
Francolini, G. and Desideri, I. and Stocchi, G. and Salvestrini, V. and Ciccone, L. P. ar	2020	Medical Oncology	Artificial Intelligence in radiotherapy: state of the art and future directions	Not primary research
Ganapathy, K. and Abdul, S. S. and Nursetyo, A. A.	2018	Neurology India	Artificial intelligence in neurosciences: A clinician's perspective	Not primary research
Geum Hee, Jeong	2020	Korean Journal of Women Health Nursing	Artificial intelligence, machine learning, and deep learning in women's health nursing	Not primary research
Godeke, J. and Muensterer, O. and Rohleder, S.	2020	Chirurg	[Artificial intelligence in pediatric surgery : Present and future]	Not primary research
Hansen, A. and Herrmann, M. and Ehlers, J. P. and Mondritzki, T. and Hensel, K. O.	2019	JMIR PUBLIC HEALTH AND SURVEILLANCE	Perception of the Progressing Digitization and Transformation of the German Health Care System Among Experts and the Public: Mixed M	Does not satisfy OECD definition of artificial intelligence
Hirsch, Tad and Soma, Christina and Merced, Kritzia and Kuo, Patty and Dembe, Aa	2018	Proceedings of the 2018 Designing Interactive Systems Confere	"It's hard to argue with a computer": Investigating Psychotherapists' Attitudes towards Automated Evaluation	Does not satisfy OECD definition of artificial intelligence
Hsu, Hui-Mei and Chang, I. Chiu and Lai, Ta-Wei	2016	International Journal of Medical Informatics	Physicians' perspectives of adopting computer-assisted navigation in orthopedic surgery	Does not satisfy OECD definition of artificial intelligence
Hung, Yu-Ting and Wu, Ching-Fang and Liang, Te-Hsin and Chou, Shin-Shang and Cl	2019	Journal of Medical Internet Research	Developing a Decision-Aid Website for Breast Cancer Surgery: An Action Research Approach	Does not satisfy OECD definition of artificial intelligence
Inserro, A. and Topol, E.	2020	AMERICAN JOURNAL OF MANAGED CARE	What Accounts for the High Cost of Care? It's the People	Does not satisfy OECD definition of artificial intelligence
Kang, J. and Schwartz, R. and Flickinger, J. and Beriwal, S.	2015	International Journal of Radiation Oncology, Biology, Physics	Machine Learning Approaches for Predicting Radiation Therapy Outcomes: A Clinician's Perspective	Not primary research
Karches, K. E.	2018	Theoretical Medicine and Bioethics	Against the iDoctor: why artificial intelligence should not replace physician judgment	Not primary research
Kataria, S. and Ravindran, V.	2018	Rheumatology International	Digital health: a new dimension in rheumatology patient care	Not primary research
Kazi, A. M. and Qazi, S. A. and Ahsan, N. and Khawaja, S. and Sameen, F. and Saqib	2020	JOURNAL OF MEDICAL INTERNET RESEARCH	Current Challenges of Digital Health Interventions in Pakistan: Mixed Methods Analysis	Does not satisfy OECD definition of artificial intelligence
Kenner, Alison	2015	Health, Risk & Society	Asthma on the move: how mobile apps remediate risk for disease management	Does not satisfy OECD definition of artificial intelligence
Kopanitsa, G.	2020	Studies in Health Technology & Informatics	Follow-Up on the Abnormal Laboratory Test Results with Automatic Interpretation	No free text data
Kopanitsa, G.	2020	Studies in Health Technology & Informatics	Automatic Interpretation of Laboratory Tests and Its Influence on Follow-up	No free text data
Kudina, O. and de Boer, B.	2021	JOURNAL OF EVALUATION IN CLINICAL PRACTICE	Co-designing diagnosis: Towards a responsible integration of Machine Learning decision-support systems in medical diagnostics	Not primary research
Lafage, R. and Pesenti, S. and Lafage, V. and Schwab, F. J.	2018	European Spine Journal	Self-learning computers for surgical planning and prediction of postoperative alignment	Not primary research
Li, R. C. and Asch, S. M. and Shah, N. I. H.	2020	NPJ DIGITAL MEDICINE	Developing a delivery science for artificial intelligence in healthcare	Not primary research
Li, Weiheng and Fan, Xiangmin and Zhu, He and Wu, Jingzheng and Teng, Dongxing	2020	Proceedings of the ACM Turing Celebration Conference - China	Research on the Influencing Factors of User Trust Based on Artificial Intelligence Self Diagnosis System	No free text data
Liaw, W. and Kakadiaris, I. A.	2020	Annals of Family Medicine	Primary care artificial intelligence: a branch hiding in plain sight	Not primary research
Liew, C.	2018	European Journal of Radiology	The future of radiology augmented with Artificial Intelligence: A strategy for success	Not primary research
Loftus, T. J. and Filiberto, A. C. and Balch, J. and Ayzengart, A. L. and Tighe, P. J. anc	2020	Journal of Surgical Research	Intelligent, Autonomous Machines in Surgery	Not primary research
Mühlbauer, V., Berger-Höger, B., Albrecht, M., Mühlhauser, I. and Steckelberg, A.	2019	BMC Health Services Research	Communicating prognosis to women with early breast cancer - overview of prediction tools and the development and pilot testing of a de	Does not satisfy OECD definition of artificial intelligence
Marchesoni, Maria Andersson and Axelsson, Karin and Lindberg, Inger	2014	Journal of Health Organization & Management	Digital support for medication administration--a means for reaching the goal of providing good care?	Does not satisfy OECD definition of artificial intelligence
Maulik, P. K. and Kallakuri, S. and Devarapalli, S. and Vadlamani, V. K. and Jha, V. a	2017	Journal of Global Health	Increasing use of mental health services in remote areas using mobile technology: A pre- post evaluation of the SMART Mental Health pro	Does not satisfy OECD definition of artificial intelligence
Maulik, P. K. and Tewari, A. and Devarapalli, S. and Kallakuri, S. and Patel, A.	2016	PLoS ONE [Electronic Resource]	The Systematic Medical Appraisal, Referral and Treatment (SMART) Mental Health Project: Development and Testing of Electronic Decisio	Not research of implementation
Mazzanti, M. and Shirka, E. and Gjergo, H. and Hasimi, E.	2018	Current Cardiology Reports	Imaging, Health Record, and Artificial Intelligence: Hype or Hope?	Not primary research
McCudden, C.	2017	Clinical Biochemistry	The future of artificial intelligence and interpretative specialization in clinical biochemistry	Not primary research
McInerney, C. D. and Scott, B. C. and Johnson, O. A.	2021	JCO clinical cancer informatics	Are Regulations Safe? Reflections From Developing a Digital Cancer Decision-Support Tool	No free text data
Meropol, N. J.	2018	Clinical Advances in Hematology & Oncology	Opportunities for using big data to advance cancer care	Not primary research
Mollura, D. J. and Culp, M. P. and Pollack, E. and Battino, G. and Scheel, J. R. and M	2020	Radiology	Artificial Intelligence in Low- and Middle-Income Countries: Innovating Global Health Radiology	Not primary research
Morris, D. and Bharadwaj, P.	2018	JOURNAL OF PAIN AND SYMPTOM MANAGEMENT	An Electronic Clinical Decision Support Tool to Assist Hospital Physicians with Prognosis: Development and Mixed Methods Evaluation	No free text data
Mossadegh, S. and Kyrimi, E. and Marsh, W. and Tai, N.	2016	BRITISH JOURNAL OF SURGERY	Implementation science: a Bayesian prediction tool for acute traumatic coagulopathy	No free text data
Nencka, A. S.	2020	JOURNAL OF MAGNETIC RESONANCE IMAGING	Editorial for "Top 10 Reviewer Critiques of Radiology Artificial Intelligence (AI) Articles: Qualitative Thematic Analysis of Reviewer Critiques	Not primary research
Ozdemir, V.	2021	OMICS-A JOURNAL OF INTEGRATIVE BIOLOGY	Digital Is Political: Why We Need a Feminist Conceptual Lens on Determinants of Digital Health	Not primary research
Padmanabhan, S. and Tran, T. Q. B. and Dominiczak, A. F.	2021	Circulation Research	Artificial Intelligence in Hypertension: Seeing through a Glass Darkly	Not primary research
Palmisciano, P. and Jamjoom, A. A. B. and Taylor, D. and Stoyanov, D. and Marcus,	2020	World Neurosurgery	Attitudes of Patients and Their Relatives Toward Artificial Intelligence in Neurosurgery	No free text data
Park, Jaeyeon and Cho, Hyeon and Balan, Rajesh Krishna and Ko, JeongGil	2020	Proc. ACM Interact. Mob. Wearable Ubiquitous Technol.	HeartQuake: Accurate Low-Cost Non-Invasive ECG Monitoring Using Bed-Mounted Geophones	Does not satisfy OECD definition of artificial intelligence
Pillalamarri, S. S. and Huyett, L. M. and Abdel-Malek, A.	2018	Journal of Diabetes Science & Technology	Novel Bluetooth-Enabled Tubeless Insulin Pump: A User Experience Design Approach for a Connected Digital Diabetes Management Platfc	Does not satisfy OECD definition of artificial intelligence
Rice, Sabriya	2016	Modern Healthcare	Big data yield new hopes, challenges in cancer care	Not primary research
Schaeffer, Chad and Booton, Lawrence and Halleck, Jamey and Studeny, Jana and C	2017	Health Care Manager	Big Data Management in US Hospitals	Does not satisfy OECD definition of artificial intelligence
Schmidt, Charlie	2017	JNCI: Journal of the National Cancer Institute	M. D. Anderson Breaks With IBM Watson, Raising Questions About Artificial Intelligence in Oncology	Not primary research
Shaikh, F. and Franc, B. and Allen, E. and Sala, E. and Awan, O. and Hendrata, K. an	2018	Journal of the American College of Radiology	Translational Radiomics: Defining the Strategy Pipeline and Considerations for Application-Part 2: From Clinical Implementation to Enterpr	Not primary research
Song, C. and Kong, Y. and Huang, L. and Luo, H. and Zhu, X.	2020	Biomedicine & Pharmacotherapy	Big data-driven precision medicine: Starting the custom-made era of iatrology	Not primary research
Streefkerk, Roel H. R. A. and Moorman, Peter W. and Parlevliet, Gerard A. and van	2014	Infection Control & Hospital Epidemiology	An Automated Algorithm to Preselect Patients to Be Assessed Individually in Point Prevalence Surveys for Hospital-Acquired Infections in !	No free text data
Taylor, R. Andrew and Haimovich, Adrian D. and Kline, Jeffrey A.	2021	Academic Emergency Medicine	Machine Learning in Emergency Medicine: Keys to Future Success	Not primary research
Thompson, R. F. and Valdes, G. and Fuller, C. D. and Carpenter, C. M. and Morin, O	2018	Radiotherapy & Oncology	Artificial intelligence in radiation oncology: A specialty-wide disruptive transformation?	Not primary research
Upton, Julian	2020	Pharmaceutical Executive	Expediting the Cancer Treatment Path: Following her own experience, a health tech entrepreneur turned to AI to better guide other patier	Not primary research
van Oostveen, C. J. and Braaksma, A. and Vermeulen, H.	2014	CIN: Computers, Informatics, Nursing	Developing and testing a computerized decision support system for nurse-to-patient assignment: a multimethod study	No free text data
Villamil, Valentina and Deloria, Rochelle and Wolbring, Gregor	2019	Proceedings of the 5th Workshop on ICTs for improving Patient	Artificial intelligence and machine learning: what is the role of social workers, occupational therapists, audiologists, nurses and speech lan	Not primary research
Vinblad, E. and Larsson, I. and Lonn, M. and Olsson, E. and Nygren, J. M. and Svedb	2019	JMIR Formative Research	Development of a digital decision support tool to aid participation of children with disabilities in pediatric rehabilitation services: Explorati	Does not satisfy OECD definition of artificial intelligence
Walter, S. and Gruss, S. and Frisch, S. and Liter, J. and Jerg-Bretzke, L. and Zujalovic	2020	FRONTIERS IN MEDICINE	"What About Automated Pain Recognition for Routine Clinical Use?" A Survey of Physicians and Nursing Staff on Expectations, Requireme	No free text data
Wang, Z. and Zhao, Q. and Yang, J. and Zhou, F.	2020	Chinese Medical Sciences Journal	Enhancing Quality of Patients Care and Improving Patient Experience in China with Assistance of Artificial Intelligence	Not primary research
Watson, D. and Womack, J. and Papadakos, S.	2020	CRITICAL CARE NURSING QUARTERLY	Rise of the Robots Is Artificial Intelligence a Friend or Foe to Nursing Practice?	Not primary research
Yao, X. and McCoy, R. G. and Friedman, P. A. and Shah, N. D. and Barry, B. A. and E	2020	American Heart Journal	ECG AI-Guided Screening for Low Ejection Fraction (EAGLE): Rationale and design of a pragmatic cluster randomized trial	Does not satisfy OECD definition of artificial intelligence
Yoshida, Daisuke and Suzuki, Takao and Shimada, Hiroyuki and Park, Hyuntae and I	2014	Geriatrics & Gerontology International	Using two different algorithms to determine the prevalence of sarcopenia	Does not satisfy OECD definition of artificial intelligence
Yu, K. H. and Kohane, I. S.	2019	BMJ Quality and Safety	Framing the challenges of artificial intelligence in medicine	Not primary research
Yu, Q. and Jing, S. and Tai, Y. and Wu, S. and Chang, J. and He, P.	2019	Chinese General Practice	Multidimensional analysis of the healthcare big data policy documents in china	Not primary research
Czerwinska, A., Souter, J. and Colley, S.	2016	Foundation of Nursing Studies: Improvement Insights	An holistic approach to nutrition and diet in palliative care	Does not satisfy OECD definition of artificial intelligence
Rialle V, Forest C, Verdier D, Rieu A et al	2016	Gerontechnology	How to think globally and act locally for health path, and elders' autonomy with ehealth and gerontechnology	Does not satisfy OECD definition of artificial intelligence
N/A conference proceedings	2016	Nursing Informatics conference	Studies in Health Technology and Informatics C3 - Studies in Health Technology and Informatics	No free text data
Australian Digital Health Agency	2018	Lamp	NURSING RESEARCH AND PROFESSIONAL ISSUES	Not primary research
N/A conference proceedings	2018	12th Annual Conference on Health Informatics Meets eHealth,	Studies in Health Technology and Informatics C3 - Studies in Health Technology and Informatics	No free text data
Jones, R and y A. and Hollen, Patricia J. and Wenzel, Jennifer and Weiss, Geoff and	2018	Cancer Nursing	Understanding Advanced Prostate Cancer Decision Making Utilizing an Interactive Decision Aid	Does not satisfy OECD definition of artificial intelligence
Sorwar, G. and Ali, M. and Islam, M. K. and Miah, M. S.	2016	Studies in Health Technologies and Informatics	An integrated patient information and in-home health monitoring system using smartphones and web services C3 - Studies in Health Tech	No free text data
Yu, C. H. and Casey, C. G. and Ke, C. and Lebovic, G. and Straus, S. E.	2019	CANADIAN JOURNAL OF DIABETES	Process Evaluation of the Diabetes Canada Guidelines Dissemination Strategy Using the Reach Effectiveness Adoption Implementation Ma	No free text data
Barrera, Alvaro and Gee, Carol and Wood, Andrew and Gibson, Oliver and Bayley, [	2020	Evidence Based Mental Health	Introducing artificial intelligence in acute psychiatric inpatient care: qualitative study of its use to conduct nursing observations	No free text data
Tomlinson, F. and Willis, T. A.	2019	Education for Primary Care	What are medical students' attitudes to clinical risk-scoring tools? An exploratory study	Does not satisfy OECD definition of artificial intelligence
Escobar, Jessica M.	2020	Proceedings of the 38th ACM International Conference on Desi	Understanding Patient Perception of Medical Artificial Intelligence: A Proposed Qualitative Study	Not primary research
Muller, S. D. and Lauridsen, K. G. and Palic, A. H. and Frederiksen, L. N. and Mathia	2021	JMIR MHealth and UHealth	Mobile App Support for Cardiopulmonary Resuscitation: Development and Usability Study	Does not satisfy OECD definition of artificial intelligence
N/A conference proceedings	2019	EUROPEAN JOURNAL OF GENERAL PRACTICE	Research on multimorbidity in primary care. Selected abstracts from the EGPRN meeting in Tampere, Finland, 9-12 May 2019 All abstract:	No free text data
Marjolein Lugtenberg, Dennis Pasveer, Trudy van der Weijden, Gert P. Westert & R	2015	BMC Family Practice	Exposure to and experiences with computerized decision support intervention in primary care	No free text data
Agbadje TT, Rahimi SA, Côté M, Tremblay AA, Diallo MP, Elidor H, Herron AP, Djad	2021	Patient Education and Conseling	Evaluation of a prenatal screening decision aid: a mixed methods pilot study	Does not satisfy OECD definition of artificial intelligence
Nakrem S, Solbjør M, Pettersen IN, Kleiven HH	2018	BMC Health Service Research	Care relationships at stake? Home healthcare professionals' experiences with digital medicine dispensers-a qualitative study	Does not satisfy OECD definition of artificial intelligence
Johansson-Pajala RM, Martin L, Jorsäter Blomgren K.	2018	International Journal of Health Care Quality Assurance	Registered nurses' use of computerised decision support in medication reviews: Implications in swedish nursing homes	No free text data
Currie M, Philip LJ, Roberts A.	2015	BMC Health Service Research	Attitudes towards the use and acceptance of eHealth technologies: A case study of older adults living with chronic pain and implications fc	Does not satisfy OECD definition of artificial intelligence
Bowles KH, Chittams J, Heil E, Topaz M, Rickard K, Bhasker M, Tanzer M, Behta M,	2015	Research in Nursing and Healyh	Successful electronic implementation of discharge referral decision support has a positive impact on 30- and 60-day readmissions	No free text data
American Medical Association	2016	AMA Digital Health Report	Digital Health Study	Does not satisfy OECD definition of artificial intelligence
Abidi S, Vallis M, Piccinini-Vallis H, Imran SA, Abidi SSR.	2017	Studies in health technology and informatics	A digital framework to support providers and patients in diabetes related behavior modification	Not research of implementation
Oikonomidi, T. and Ravaud, P. and James, A. and Cosson, E. and Montori, V. and Tr	2021	Mayo Clinic Proceedings	An International, Mixed-Methods Study of the Perceived Intrusiveness of Remote Digital Diabetes Monitoring	Does not satisfy OECD definition of artificial intelligence
Rauter, C. M. and Wohlke, S. and Schicktanz, S.	2019	Studies in Health Technology & Informatics	Organizations Towards "Big Data"-Driven Approaches in Personalized Medicine? An Empirical-Ethical Study in Health-Related IT	Does not satisfy OECD definition of artificial intelligence
Andrews, J. A. and Astell, A. J. and Brown, L. J. E. and Harrison, R. F. and Hawley, M	2017	Studies in Health Technology and Informatics	Technology for Early Detection of Depression and Anxiety in Older People	No free text data
Muller-Staub, M. and de Graaf-Waar, H. and Paans, W.	2016	CIN-COMPUTERS INFORMATICS NURSING	An Internationally Consented Standard for Nursing Process-Clinical Decision Support Systems in Electronic Health Records	Does not satisfy OECD definition of artificial intelligence
Capobussi, M. and Banzi, R. and Moja, L. and Bonovas, S. and Gonzalez-Lorenzo, M	2016	Recenti Progressi in Medicina	[Computerized decision support systems: EBM at the bedside]	No free text data
Clausen CE, Leventhal BL, Nytrø Ø, Koposov R, Westbye OS, Røst TB, Bakken V, Koc	2021	J Am Child Adolescent Psychiatry	Clinical Decision Support Systems: An Innovative Approach to Enhancing Child and Adolescent Mental Health Services	Not primary research
Erkelens DC, van Charldorp TC, Vinck VV, Wouters LT, Damoiseaux RA, Rutten FH, Z	2021	Patient education and counseling	Interactional implications of either/or-questions during telephone triage of callers with chest discomfort in out-of-hours primary care: A cc	No free text data
Torenholt R and Saltbaek L	2020	Sociology of Health and Illness	Patient data work: filtering and sensing patient-reported outcomes	Does not satisfy OECD definition of artificial intelligence
Yu, C. H. and Casey, C. G. and Ke, C. and Lebovic, G. and Straus, S. E.	2019	CANADIAN JOURNAL OF DIABETES	Process Evaluation of the Diabetes Canada Guidelines Dissemination Strategy Using the Reach Effectiveness Adoption Implementation Ma	No free text data
Gardner, C. and Moseley, G. L. and Karran, E. L. and Wiles, L. K. and Hibbert, P.	2020	CANADIAN JOURNAL OF PAIN-REVUE	Implementing high value back pain care in private physiotherapy in Australia: A qualitative evaluation of physiotherapists who participate	No free text data
Smith, Jane R. and Musgrave, Stanley and Payerne, Estelle and Noble, Michael and	2018	Trials	At-risk registers integrated into primary care to stop asthma crises in the UK (ARRISA-UK): study protocol for a pragmatic, cluster randomi	Not primary research
