# Supplementary material for: Stakeholder Perspectives of Clinical Artificial Intelligence Implementation: Systematic Review of Qualitative Evidence
Source: J Med Internet Res. 2023 Jan 10;25:e39742. doi: 10.2196/39742 (PMC9875023; doi:10.2196/39742)
Supplement: Multimedia Appendix 3 [file jmir_v25i1e39742_app3.zip › 1. Condition/1a. Nature of condition or illness/1a.1 Type or format of care needs.docx]

**Name:** 1a.1 Type or format of care needs

Abejirinde-2018

workflow was still broken when women needed to provide their urine samples.

Andrews-2017

Another participant commented on how technology could facilitate mood reports where patients may have struggled with pen and paper mood diaries:

P1: Yeah I think we have done kind of mood diaries with older people as well. I think that the very old and more physically unwell people might struggle to do that, and often it’s a case of being able to write things down, and track it, and that way can be very difficult, so if people have an easier way of doing that, that might be useful.

Blease-2019

Medicine, particularly general practice, is an art; listening to ideas concerns and expectations and negotiating a shared plan with the patient. Often doing nothing other than listening is required. I wonder how well a computer will be able to do this? [Participant 285]

de Watteville-2021

“The objectives are clear but the use is not necessarily adapted to the pathologies and not necessarily usable in all patients (in about 50% of cases)

Goetz-2020

Some students believed a vPCP would be inappropriate for mental health concerns, especially in conditions like depression where human connection is important: “. . .so much of medicine is not medicine. It’s being a person, being a listener, being somebody you can talk to. . .” (Fourth year medical student)

Population health. A theme that arose in multiple groups was the use of a vPCP for population health and epidemiology.

“[If] it was like a virus, and it actually, it was like spreading, and so the person who was, like, helping, like, nurses and all, they’re also getting infected. . .If there would have been a virtual machine to do that. . . let’s put the machine inside the room; let’s not enter there. . .. I feel like some extent, like, in some areas, we definitely need AI.” (First year graduate student)

“[I]n addition to that, epidemiologically. . . we could predict real-time health outbreaks that are occurring. . . And all of a sudden, we have epidemiologic data that suggests, oh, in this region the outbreak is concentrated. It was this batch of lettuce that was contaminated. We can trace it back, and so I see a place for that.” (First year medical student)

Henshall-2019

Regarding compliance, all psychiatrists felt that patients would be more likely to comply with their medications after engaging with the decision-making process via the DST. However, some patients/carers felt that regular face-to-face doctor/patient discussions were required to avoid patients feeling pressured into making decisions using the DST alone. All participants acknowledged the complexity of the decision-making process and felt the DST was a small piece of the whole picture. The app … It’s a good start … Sometimes decision-making is more complex. You as a clinician have to take more into consideration rather than just a few clicks, side effects. Psychiatrist 1

Morgenstern-2021-supplementary file 6

I mean, there's no business really in public health to be honest. But there certainly is in Radiology. [Participant ID # 3]

Mozaffar-2016

There’s only one thing […] is that there is a facility to do a free format prescription. Say for example Parkinson’s medication and you need to give it at different times of the day, specific times of the day that don’t fit in with the drug rounds, or sort of a Methotrexate dose that is given weekly, you can actually select it weekly but say if the doctor did it in this free format and said Methotrexate 10 mg weekly and the system doesn’t understand that that’s weekly, it doesn’t attach it to a frequency so it then comes up every single day for admin but they’ve raised that with [supplier name] as a risk but without looking, obviously now we know that we would need to look out for it, we’ve got sort of an alert for any drugs that are prescribed free format but if you didn’t sort of look on that thing then you wouldn’t know and obviously the staff would change every day and they wouldn’t think oh this patient on Methotrexate yesterday because it would just come up what needed charting for that day, so there’s a real risk there… that’s not good enough particularly when a large number of issues that have been raised are around absolute patient safety. (Site A, Pharmacist

Patel-2018-additional file

GP has a personal interest in kidney disease. HT was a tool that was going help GP to be more aware of risk factors for diagnosis and treating kidney disease.

Rapoport-2020

I would never rely completely on a tool. … there’s many areas like this in psychiatry where … we rely more on our overall impressions so all … the diagnostic tools and scales that we use in psychiatry are limited by that. So, would this be any different for me – no. I would use it as a piece in the assessment. I wouldn’t use it as my be-all, end-all. [MD12-SP]

Reynolds-2019

“I liked it for drips. I don’t know that it really helps that much with our basic medications that we give. I don’t think it makes it them any quicker or decreases the errors, but I do like it for drips
