# Supplementary material for: Stakeholder Perspectives of Clinical Artificial Intelligence Implementation: Systematic Review of Qualitative Evidence
Source: J Med Internet Res. 2023 Jan 10;25:e39742. doi: 10.2196/39742 (PMC9875023; doi:10.2196/39742)
Supplement: Multimedia Appendix 3 [file jmir_v25i1e39742_app3.zip › 1. Condition/1a. Nature of condition or illness/1a.2 Ambiguous, complicated or rare decisions.docx]

**Name:** 1a.2 Ambiguous, complicated or rare decisions

Ash-2020

So, but it was kind of never on my radar. I think about, oh at home, what allergens do you have? What vermin are in the shelter you’re at, whatever? And I would kind of maybe not delve into the workplace as much. So it seems like it would be helpful to have just a prompt to remind you to look at that.

Because I have no idea how to write those letters. I have no idea how to gauge how many pounds someone can lift, other than, I mean, just common sense. Like, I just don’t know.

Biller-Andorno-2021

It provides an easily accessible ‘digital second opinion’, which is particularly helpful in difficult cases. ‘Something like a ‘digital second opinion’ or so I think is actually a good thing. Even if I haven’t thought of something, the tool will have thought of it.’

Blease-2019

The other issue is the inability of a machine/AI to be able to skillfully ascertain the data required from a patient for correct analysis. [Participant 453]

They [patients] are humans and subject to the vagaries of human recall, memory, and interpretation. AI may make it easier to interpret a blood result follow a protocol or order a test. But AI will always struggle when the same human can score 1/10 for a symptom today and 10/10 tomorrow. [Participant 201]

In this way, some GPs identified a positive paraprofessional role for technology in streamlining access to physicians:

Machines should be good at initial triage of uncomplicated patients presenting to primary care. [Participant 88]

Cai-2019

Many participants noted the subjectivity intrinsic in assigning cancer grade levels: “There’s a lot of subjectivity, grade 4 vs grade 5, you ask ten pathologists, four will say one thing and the rest will say another.” (P18

Catho-2020

Lack of applicability

• GE_07 (F, resident):“Recommendations are made for a certain type of patient, we have other factors that inﬂuence decisions”

• FR_04 (F, resident):“It can improve prescribing in common situations, but really it applies to common, classic conditions. As soon as you get oﬀ the track, it can no longer be part of recommendations”

• TI_05 (M, senior physician):“We have the local guidelines booklet in our pockets. For simple cases, things are clear and we use it often. In diﬃcult cases, in general with specialists[…] precisely tailored to the patient”

• GE_06 (M, resident): “Antibiotic therapy is completely linked to a global care project, so it is diﬃcult to make a decision, there are no guidelines for that”

Chow-2015

Both junior and senior physicians found ARUSC’s recommendations for renal dose adjustments to be useful [J5, S1, S3]. Although senior physicians (who were more experienced with antibiotic prescribing) were less likely to appreciate ARUSC’s recommendations for common infections, they found ARUSC useful for patients with unknown or unfamiliar infection sources [S1, S3, S4].

Dalton-2020

Whilst some prescribers emphasised that they were happy to review the SENATOR recommendations as they knew about the patients under their care, interviewees also described that hospital prescribers were often reluctant to act on the recommendations as they did not know enough about these complex multimorbid patients (who were only recently admitted to hospital), or their pharmacotherapy. I don’t know whether that’s appropriate or not because I don’t know what the decision was to put them on it in the first place. [Surgical Prescriber 2]

Gillan-2018

Finally, the efficiency in practice and the computing power afforded through AI were seen, particularly by the two RTT groups, as enablers of complexity that could improve care. Specifically, ‘all that computing power really did was give us a set of tools and made our work even more complex than it’s ever been before. So we’re able to open the door to… do things we’d never have considered before, and do them in a shorter time frame than we ever thought was possible’—(TP06)

Goetz-2020

Students felt that a virtual PCP could be used for routine illnesses, for example:

“. . . cold, flu, or high-blood pressure. . . These very common diseases, I think [a] virtual physician can handle. . .” (First year medical students)

Students believed a vPCP would not be appropriate for rare conditions:

“What if an event that has never been present, that was never used to train that AI system, presents itself in the future? What happens?” (Fourth year graduate student)

Jackson-2017

A majority of participants agreed that the eHealth model would be the most appropriate to augment or replace outpatient face-to-face management of stable and relatively less complex patients as a priority. One participant stated that ‘strict inclusion and exclusion criteria (should) be used’. Stratifying interventions by disease subtype was deemed to be important

‘People who have had the diagnosis for at least 2 years, stable for a year’. ‘Patients escalation and de-escalation steroids would be a particularly challenging area’. ‘Only stable patients with mild-to-moderate UC should be included’

decision support tool should target patients with mild to moderate UC given the simpler management requirements. Chronicity of disease was mentioned brieﬂy, and one participant suggested that the decision support tool be used ‘only by patients with stable disease and a diagnosis of IBD for at least two years’. It was agreed that patients with complex disease such as ‘obstructive symptoms, recent surgery and those with perianal disease (if CD was included) ought to be excluded’ from using the decision support tool. It was felt that the ideal patient for a model of care incorporating eHealth decision support should have mild to moderate UC and have minimal disease activity at study entry.

The major concern regarded titration of corticosteroids, where participants agreed that face-to-face contact was more appropriate compared to eHealth decision support. However, self-management of aminosalicylates with decision support, both orally and rectally, was unanimously thought to be appropriate. Furthermore, management of psychological distress was also deemed possible with the proposed decision support tool.

Joshi-2020

“The other difficulty in general is the definition or gold standard for sepsis makes it hard for…any standard but machine learning in particular because it is much easier…if you have true positives and true negatives. And then the ambiguous cases can be used for learning but here the ambiguous cases, often nobody really knows what to do with them.”

Knoble-2015

The primary reason given was the time the application took for routine patient encounters. Of those that used the application, they reported use of the ealgo only when they were confused or had more difﬁcult cases. They did not feel the time required to use the e-algo warranted its use in the cases they perceived as routine or simple. Their feelings were that the vast majority of their cases are routine; they stated the need for the e-algo was minimal and just wasted their time. Their estimate of usage ranged from 5 to 20 patients per week or about 15% of all acute problem visits.

Liberati-2015

«I find this system very very useful, but for doctors, not for us. In my branch (surgical) it would be more difficult to apply. We try to look at the evidence, from a diagnostic point of view maybe we can do it a little more, but from the point from a therapeutic point of view it is really complicated. I do a example from my field: broken femur, nail or plaque? Find 5000 jobs but hardly one synthesis. Instead for doctors, in the prescription, in the drug therapy, it would be a great thing to have this system ". (Orthopedic surgeon, setting B)

Liberati-2017

So this morning we had a patient with hyponatremia, which is notoriously iatrogenic, but because they were taking many medications we were not sure which one caused it. Having the computer at the bedside meant that we were able to consult the evidence right away and identify which of the ten medications they were on was causing the hyponatremia. And we couldn’t have done it without the technological support... (Physician, setting C2

Morgenstern-2021

The broad implications of AI for public health are that AI can serve as an intermediary between the huge amounts of data that we generate and action

Some thought that AI’s greatest potential lies in causal inference and hypothesis generation.

What are the factors that contribute to drowning in [the] beach? [You] look for similarities, patterns, trends that were not anything that would have been [previously] comprehended because people would focus on, you know, they couldn’t swim or they didn’t have life jackets. But it may be that they all have congenital heart disease. Again, not likely […], but you’re getting my drift. [Participant ID # 9].

In contrast, other interviewees thought that AI has little potential for identifying novel causes.

… we’re not going to discover new risk factors. I don’t believe that. I don’t believe these methods are

going to reveal risks for sub-populations that we don’t already know. [Participant ID # 12]

Morgenstern-2021-supplementary file 6

it's a system, a real-time system, where we receive triage data from [a large number of] hospitals all across [the region] here in our data centre 24 hours a day, 7 days a week and, um, we're using machine learning techniques on that, in real time, on that triage data to see if we can detect anomalies and potential threats to public health. [Participant ID # 8]

Pannebakker-2019

The GPs mainly felt that the melanoma eCDS was most useful for borderline decision-making:

’My experience has been the scores have either been 5 or 6, or 1, and not much in the middle, so you either will or you won’t [refer] . . . if there are lesions that score 2 or 3 or 4, that’s going to be the grey area where actually it might help.’ (F, 40 years)

Porter-2018

. In this situation, where paramedics were making key decisions about patient care in a context of ambiguity and uncertainty, the CCDS was also seen as a useful record of the clinical assessment and decision-making process, and confirmation that the course of action was appropriate and documented:

It sort of provided extra evidence for me to say, yes, I'm quite happy that that's the way we're going to take. (End S2 02)

And I’ve now got something recorded and written, you know, legibly, that will back my decision. (Mid S2 02)

Rapoport-2020

I used it when I felt there was a dilemma. And, unfortunately, that was probably reflected in the results. A lot of the times there was no consensus. And that was kind of frustrating but, at least it allowed me to reflect and to highlight some of the different points on the decision process. And so sometimes even that would just give me a little bit of clarity. [MD01-SP]

I used it in cases which I thought were grey zones, really grey zones. And I confess they didn’t help. Either it corroborated my decision or it gave me a decision that, even when I saw that it was more lenient towards the driver, it didn’t make me change my mind. [MD07-SP]

Reynolds-2019

“[The device is most useful] for drugs that are uncommon to us, that we don’t use that often, is what I found the most beneﬁcial. Otherwise, drugs that we use, sometimes every day, again, they’re just second nature to us and we already know what we’re doing with those.”

Vanhille-2018

Surgeon Feedback on Future Applications of Virtual Surgery Based on Computational Fluid Dynamics Simulations

Category a Specific Surgeon Feedback Patient counseling

“…the undecided patient and the patient I am unsure how much benefit they will get from surgery.” “…the patient with symptoms out of proportion to their physical exam or radiographic findings.” “…be

“Very helpful in preadolescent cleft patients with nasal obstruction because they have unique anatomic obstructions.” “…useful for a fixed obstruction, such as subglottic stenosis or tracheal stenosis, in order to know how wide to open to improve airflow.

Wang-2018

Pharmacists and nurses also emphasised that patients’ risk factors associated with antithrombotic therapy were not static; therefore, they tended to see this tool as most useful for regular reviews of patients. This aspect of tool use seemed to be overlooked by the GPs and specialists.

Wang-2018-Tables

For me it (CARATV2.0) might be useful but I imagine that I would most likely be using it in patients where it is not certain either way. Like I probably wouldn’t use it if it clear in my mind that there is high risk of stroke and low risk of bleeding … Whereas for patients who a bit equivocal then I may use this tool to help me choose one or the other.
