# Supplementary material for: Stakeholder Perspectives of Clinical Artificial Intelligence Implementation: Systematic Review of Qualitative Evidence
Source: J Med Internet Res. 2023 Jan 10;25:e39742. doi: 10.2196/39742 (PMC9875023; doi:10.2196/39742)
Supplement: Multimedia Appendix 3 [file jmir_v25i1e39742_app3.zip › 1. Condition/1a. Nature of condition or illness/1a.3 Quality of current care.docx]

**Name:** 1a.3 Quality of current care

Abejirinde-2018

a few respondents specifically stated that their satisfaction of the visit was because of the otherwise absent diagnostic service provided by B4M

Adams-2020

“I don’t want to know and have to worry for 6 months before they say, ‘Oh it’s nothing.’” The time spent waiting between examinations resulted in increased anxiety and interfered with other aspects of their lives.

Ash-2020

I’m always concerned that we are quick to give the diagnosis of asthma and not question why it’s starting in someone who did not have it in childhood. So yes, it (the CDS) would be very helpful

Cameron-2017

The physician uncertainty of whether to report patients to transportation administrators, the family caregivers’ frustration with this uncertainty, and the OTs’ and transportation administrators’ concerns about standardization lent credence and importance to the development of the tool.

Catho-2020

FR_03 (M, resident):“In an end-of-life context, generally there is not much”

Collard-2020

With regard to exercise, one of the most commonly discussed beliefs about an automated insulin delivery system was the possible freedom it would provide. One aspect of this freedom was being able to partake in exercise without constantly checking blood glucose levels. ‘It’d be nice on long runs, not to have to worry about getting low blood sugar. That would be nice. As it is, I always carry sugar with me, but sometimes, when I’m on a long run, I just don’t feel like eating sugar.’ (Adult, Focus group.)

‘It would be useful during exercise. Because if it was still making my blood sugar have better control, it would stop me from getting low during exercise and then spiking afterwards. I think it would be very useful if you do a lot of sport.’ (Adolescent, Focus group.

The belief that automated insulin delivery systems would provide some relief from constant worry about the possibilities of going hypoglycaemic and/or hyperglycaemic before, during, and after exercise was discussed. ‘Yeah,… I coach a junior football team so it would be nice not to have to panic while I am doing that and I am going to run low and have to run back and grab some more sweets.’ (Adult, Focus group.) ‘The hardest time would probably be when I am doing exercise, because sometimes when I am doing exercise, I’m quite prone to go very low. And so, that would tell me if I am going up or going down, and I could just look and think I’m going to do this or do that and it would just really help.’ (Child, Focus group.) ‘Because, personally, sometimes when I do exercise, I go down, and sometimes I go up... And I don’t know how to prepare for it because if I eat food beforehand, and then I end up going up, I’ll go really high. Or if … I do exercise, I’ll go really low. It would definitely help with that.’ (Adolescent, Interview.)

Another potential benefit was the hope that automated insulin delivery systems would be able to accurately measure blood glucose levels while exercising and adjust accordingly, decreasing the need to ‘guess’ the correct dose of insulin. ‘It does seem like it would take the human error aspect out of it… So it could potentially – what I see as a benefit of this being for me is when I go out and ride for 3 or 4 hours then I come home, I don’t have to mess around with these calculations. Potentially this could help and say, okay, well I don’t need as much insulin or my blood sugar is going down and then it would react accordingly.’ (Adult, Focus group

‘Again, I can imagine that anything that reduces sort of the guesswork of needing to know where her blood sugar is going to go, and eat appropriately or take the appropriate amount of insulin. She’d have to eat so much sugar to compensate for her blood sugar dropping during a workout that it wasn’t worth it. And if the artificial pancreas [automated insulin delivery system] could help avoid some of that guesswork, I think it’d be great.’ (Partner, Focus group.)

Fan-2021

Interestingly, the disease type was also highly related to users’ experiences and their satisfaction level (Figure 10).

Flint-2019

Common themes included a perceived lack of need for the tool due to the simplicity of an opioid conversion task, frequency of performing opioid conversions

Jackson-2017

It was unanimous that ‘eHealth in IBD is required’, with one participant expressing that ‘people want this thing already’.

Jauk-2021

The application also supported the targeting of patients with a delirium diagnosis in a previous stay.

“Especially patients with a diagnosis of delirium in the past are being targeted earlier now.”

Keogh-2019

In particular, clinicians felt that iPrevent would increase the likelihood of clinicians discussing risk reducing medication with women at moderate risk (see Table 5, quote 3&4).

At the moment, those moderate risks, they're going back to their [PCPs], and their [PCP] says, “well, I don't know anything about that.” Then they get sent to the breast surgeon. Breast surgeon says, “you're not high risk, my rooms are full, I can't see you.” We're back at that limbo

Morgenstern-2021-supplementary file 6

fundamentally, we know that individually focused health promotion is limited in its population health impact. So, I don't see it transforming the way we do health promotion. [Participant ID # 12]

Nicks-2016

“I feel like I’m pretty good at being able to walk into a home and being able to assess what is around me and what I need to address and what I don’t.” (Standard practice nurse)

Porter-2018

Many paramedics held mixed views about the CCDS, reporting benefits but also questioning the extent to which it could assist them with their decision-making. Sometimes we have a difference of opinion between myself and the software, and I’m going to every time default to my idea on that one. And just because it – it’s quite basic software I think. (Mid S2 03

Ruppel-2021

If one follows this line of argument, there is nothing left to do but - as one of the psychiatrists interviewed by me aptly remarked, gradually “pulls herself out of the swamp by the head” (P6: 989). In view of this seemingly hopeless situation, many hopes are directed towards computational technologies, especially those processes that are regularly subsumed under the term “artificial intelligence”

Shannon-2021

More broadly, providers found that the intervention allows for early detection of cases of depression and AUD. As a result, patients are engaged in the healthcare system earlier in the clinical course. The providers indicate that early engagement helps prevent conditions from progressing to crisis points, making them more treatable and preventing some of the worst outcomes. One interviewee stated, “Advantages, clearly the detection of mental health problems that are sometimes under-diagnosed and are not visible, sometimes neither for patients nor for the doctors who treat them [the patients]. So, I think they [the patients] win, that’s why, and they win because obviously this means a much more comprehensive care of all their health.”

Soling-2020-supplementary file

The thing is, we have more and more chronically ill, old patients with a lot of drugs, that this also ... and that is increasing. And that is why I believe it is increasingly necessary to have this feeling of security that everything is going well concerning polypharmacy.”

Wickstrom-2020

The participants described patients with hard-to-heal ulcers as being neglected and not prioritized in health care. They spoke about a lack of continuity and quality, and this obvious medical need positively influenced engagement in the introduction of the DDSS:

If you see an area where an improvement is needed, where there’s developmental work that needs to be done. That’s something that fosters engagement. [Participant 1]
