# Supplementary material for: Stakeholder Perspectives of Clinical Artificial Intelligence Implementation: Systematic Review of Qualitative Evidence
Source: J Med Internet Res. 2023 Jan 10;25:e39742. doi: 10.2196/39742 (PMC9875023; doi:10.2196/39742)
Supplement: Multimedia Appendix 3 [file jmir_v25i1e39742_app3.zip › 1. Condition/1a. Nature of condition or illness/1a.4 Decision urgency and impact.docx]

**Name:** 1a.4 Decision urgency and impact

Biller-Andorno-2021

Even if an AI- based system were fast to access and deliver the requested prediction, it would still not be useful in emergencies, where every minute has significant impact on resuscitation outcomes. ‘You’d have to run to the nurses’ station, open the system and see what it says. And if someone is ‘CPR yes’ you have lost a minute. 10% less survival.’ (Interview 7

Bourla-2018

The main obstacle was the psychiatrists’ fear that they would do more harm than good either by generating anxious counter-reactions (especially with regard to the EMA smartphone app and connected wristband) or by creating a risk of overtreatment by diagnosing problems that did not exist

Goetz-2020

They also envisioned that the vPCP would be beneficial for continuous monitoring of chronic conditions, for example:

“You could have someone who’s a diabetic, and you feed the artificial intelligence data about your last blood glucoses and your A1C’s without leaving your home.” (First year medical student)

Jauk-2021

The consensus of the expert group on perceived usefulness was that the application offered a great support in early recognition of delirium risk patients and helped to reduce resources for screening.

“The application gives good support – I am convinced of its usefulness.” “Due to the delirium prediction application, we were already able to prevent the sliding into a strong delirium

with simple interventions.” “I see the application as a benefit, as we are able to reduce the time for delirium screening.

Lawton-2014

A few (n = 2) also discontinued use in light of their experiences of administering recommended doses, observing repeated high or low blood sugar levels and, hence, losing trust in the technology:

‘‘It was calibrated to a certain level, that other meter I got, you know, they did your carbohydrates and then your insulin and I kept questioning it and thinking ‘‘something’s not right here, I knew in my head if I give myself 2 [units] and my sugar’s nine and a half, I’m going to end up hypoing. . . so I lost faith in it and I stopped using it.’’ (M29.2)

Melo-2020

Conversely, work situations that require greater human contact and the development of therapeutic relationships will tend to be valued because they require teamwork, supervision, and divergent communication, which cannot be provided by computers. It is well known that repetitive and predictable human activities can be performed by robots and computers, but this is not the case with emergency services or any activities that require rapid response to unpredictable situations

Pope-2017

Another call handler explained that dealing with people with depression was difficult:

Some of the mental health calls are particularly hard to deal with […] A newer call adviser (CallA4) says, “after training, every time I shadowed people, they had people with mental health problems and that really panics you” (Observation, NHS 111)

Porter-2018

if they had already assessed the patient and viewed them as clearly needing hospital care, they would not use the CCDS software:

If they obviously need immediate attention – like they look on the point of collapse or they’re about to die or something, then we obviously don’t use it for that, because it’s irrelevant to be honest with you, and it’s gonna get in the way of patient care. (Mid S2 03)

Reynolds-2019

One nurse explained:

“When there is a physician at the bedside yelling orders at me with no patience, I don’t know that there will be tolerance for, ‘Let me check this machine and see if you’re telling me the right dose for fentanyl.’ I don’t know that there because it’ an emergent situation, we have to get it done and it needs to be fast. So if I say to Dr. XXX, ‘Let me look that up first.’ I’m pretty sure the answer is going to be, ‘Just get it!’”

“The only problem I might foresee is in an emergency situation, it might take too long to use it.

“I could see, just depending on how the device works, I could see it being maybe a cumbersome aspect. Sometimes you just need to get a medication in and stuff to take a minute, 3–5 min depending on how long it takes to program it in, you might just need. That baby might not be able to wait for that.”

Vanhille-2018

“…beneficial with patients who are higher surgical risk…and could predict if worth the risk to go to the operating room.”

Yang-2019

Clinicians also shared that making an objective decision could sometimes be hard. The decision to not implant was usually a death sentence for a patient.

“When I really like this patient, really want to help him or her, it sometimes helps to get a more factual view.”
