# Supplementary material for: Stakeholder Perspectives of Clinical Artificial Intelligence Implementation: Systematic Review of Qualitative Evidence
Source: J Med Internet Res. 2023 Jan 10;25:e39742. doi: 10.2196/39742 (PMC9875023; doi:10.2196/39742)
Supplement: Multimedia Appendix 3 [file jmir_v25i1e39742_app3.zip › 1. Condition/1b. Comorbidities/1b.1 Other associated health problems.docx]

**Name:** 1b.1 Other associated health problems

Andrews-2017

Another participant raised the issue of infection control, where devices are taken between patients’ houses.

P6: there’s infection control, because we’re taking it from patient to patient.

Memory was another reason cited by participants for older adults’ disengagement from digital technologies. Prompting was seen as essential for users to remember to complete required tasks.

P2: Memory is another one [potential barrier], remembering to do it, if they’re not prompted, how are they gonna remember to do it?

P5: I would have concerns about people doing it every day unless they were prompted, because from trying to get people to do exercises every day, it’s a real battle, so I think they would need some kind of prompt

P7: you could almost do with something on it, that beeps or like an alarm, to remind people, because that’s another thing is just memory, will people actually remember to do it?

Chow-2015

Both junior and senior physicians felt that ARUSC’s recommendations were inadequate in addressing the antibiotic needs of patients with multiple infections and allergies [J3–5, S2, S5].

Henshall-2019

Psychiatrists felt that the comprehensive, up-to-date and evidence-based information on the DST would increase their prescribing confidence with new medications. However, contextual factors relating to patients’ physical and social health were noted as also influencing psychiatrists’ prescribing practices

Jauk-2021

It provided support in the assessment of patients under sedation at admission, and it was used to confirm existing presumptions on delirium risk.

“It is especially an added value if patients are not responsive during admission.” “The prediction helps to corroborate my own estimation when seeing a patient.” “Also, the prediction helps us when we are not quite sure about the delirium risk.”

Orchard-2019

The only difficulty with the iECG was taking a reading for people with a tremor or arthritic fingers:

“The only difficulty was with the elderly with tremors. They got to a certain age, probably about 85, where they just couldn’t…it was almost like a screen for cognitive status.” (GP 2, Practice A).

“Some of them had essential tremor…I just couldn’t get a tracing.” (GP 1, Practice I)
