# Supplementary material for: Stakeholder Perspectives of Clinical Artificial Intelligence Implementation: Systematic Review of Qualitative Evidence
Source: J Med Internet Res. 2023 Jan 10;25:e39742. doi: 10.2196/39742 (PMC9875023; doi:10.2196/39742)
Supplement: Multimedia Appendix 3 [file jmir_v25i1e39742_app3.zip › 1. Condition/1b. Comorbidities/1b.2 Aligning patient and health priorities.docx]

**Name:** 1b.2 Aligning patient and health priorities

Ash-2020

We have people who are undocumented, which means that they don’t necessarily want to report anything going on at work.

Some of the patients come in with back pain, we get back pain because we got a lot of drug seekers coming in, and that’s why we become very defensive with them. Things like that start popping up because the more you ask, the more they’re going to, now they can’t even get out of the chair, even though they were running down the hall before they got to us. And the more you ask questions like that, it’s almost like you’re leading them. That’s what they’re looking for, and then, so we have to play that, I hate to say it, like a game, with you trying to ﬁgure out whose legit and who’s not legit

Because we’re trying to avoid getting into that. Because what happens is the next thing we know, now we got a workers’ comp case

Language, cultural as well as fear of divulging information about their work. So you know like the under the table work and that type of stuff is, there’s lots of resistance to try to share that information

Clyne-2016

Some GPs were sceptical about the benefits of discontinuation of such medications in older patients:

“Sometimes, for example, in relation to benzodiazepine, em, you know, somebody might be on benzodiazepines and has been for 40 years, which one of the patients actually was, I don’t think it’s appropriate to stop that. If they’re stable and they can get on with their lives then I think it would cause more hassle for them.” (GP1, intervention practice).

Collard-2020

Similarly, children had a notion that an automated insulin delivery system would allow them to improve sports performance. ‘It’s keeping your numbers up during the match, so you don’t have to like at half-time, test and that. You can keep on routines and focus more on the actual game.’ (Child, Interview.)

Porter-2018

Decision-making was reported as also being shaped by the time of day and the situation of the patient, with the CCDS software being regarded as being a potential cause of additional delay:

And – and it was in the early hours of the morning, and it’s not a problem from our point of view because we were – we were working, [laughs] you know, but from – from – I mean the old – the – the person who had fallen had their son and his wife were there as well, and they were there because they’d been called and they were like, sort of, ‘Well, we want to go back home to bed’, you know. (Mid S2 02

Roebroek-2020

It was mentioned several times that it is not always straightforward to turn treatment recommendations into behavioral changes for this patient group:

“Most people have been in care for a long time and suffer from several disabilities. Sometimes you are able to initiate something new by putting in a lot of effort, but sometimes it just does not work because some patients have been doing things in a certain way for so long it’s difficult to motivate them to try things in a new way.” [C7]

Soling-2020-supplementary file

“So now you get, practically all the time, calls from pharmacists who think something is not working with one or the other, but they don't see the clinical presentation. Now, if you have a Parkinson's patient and want to calm him down somehow because he's nervous all night, then maybe that reduces the effect of his medication, but seen. Parkinson's then from the pharmaceutical perspective alone, that's not The medical assistant sits there, a red light goes on, and they tell the patient, watch out, this reduces the effect of the Parkinson medication;
