# Supplementary material for: Stakeholder Perspectives of Clinical Artificial Intelligence Implementation: Systematic Review of Qualitative Evidence
Source: J Med Internet Res. 2023 Jan 10;25:e39742. doi: 10.2196/39742 (PMC9875023; doi:10.2196/39742)
Supplement: Multimedia Appendix 3 [file jmir_v25i1e39742_app3.zip › 1. Condition/1c. Sociocultural factors/1c. Sociocultural factors.docx]

**Name:** 1c. Sociocultural factors

Andrews-2017

one participant described how patients could benefit from having control over reporting their mental state.

P4: They get dignity, they get control, by identifying where they are at, what’s happening to them, giving them the means to move themselves forward, which they wouldn’t have had in the past.

Ash-2020

Those are important things to know about and be aware of… this person’s a school bus driver and they’re, you know, am I going to control them (diabetics) more closely? Less closely? Does that have an impact on their overall risk to school children?

(I would prioritize) the return to work (CDS) I think. Because you know really, the decision about the return to work is one of the very hardest ones.

I was thinking I have a patient with diabetes who is a policewoman … and I was like, “Why couldn’t you take your diabetes medicine?” And she’s like, “It’s because I can’t eat while I’m working.”

But I also think a lot of patients could be concerned about their employment, you know? And I mean, there was a patient, for example, who, you know, he was a truck driver I think. And ODOT has this rule that you can’t be on insulin when you’re on a truck. You know, driving a commercial vehicle. And he was on that verge. They were getting ready to start him on insulin and he’s like, “No, I won’t.”

So how do we put it with the employer if the employer knows that the patient’s in danger because they haven’t had their break and haven’t measured their blood sugar or whatever. And there’s no recourse except ﬁring the patient. Then that doesn’t help anybody.

Biller-Andorno-2021

It seems counterintuitive that an AI can in fact provide relevant input on highly personal, individual decision (such as for or against resuscitation) by training on data from others. Is such a system not necessarily going to be reductionist? How good will its performance be, and how good is good enough? ‘Yes, it is certainly helpful in some cases, but I believe that a resuscitation decision is also something personal. Particularly patients who are ill but want to live up to a certain event.’ (Interview 11

Bourla-2018

Stigmatization risk

Connell-2019

Third, the care pathway had an impact on the relationship between clinicians and their patients. In particular, several PARRT team members described that alerts identified patients at an earlier stage of AKI than was the case through established clinical pathways (eg, monitoring of vital signs). This may have led to an unexpected and evolving role for some members of the team. Several respondents described how the care pathway enabled them to help patients make informed decisions surrounding end-of-life care. For example:

Why do we have to talk about end of life just as I’m about to die? [...] We could plan. Every single person we’ve been referred today has a terminal disease. [...] Trying to move the decision making back, in a more timely way. [...] We are getting an alert before they have even triggered [via vital signs], so we can probably have a sensible conversation with a patient with capacity. [Respondent 4: PARRT]

Hallen-2015

Several physicians traced the source of distress to patients’ general discomfort with the subject of death and dying. One physician expressed concerns that this discomfort might lead some patients to perceive the very act of a physician initiating a prognostic discussion as inappropriate:

Cardiologist 2: And then there would also be...the perception by the patient that before you even ﬁll this out, that the physician has given them the death question. The death exam, you know. There would be some sort of...death test.

A related concern pertained to the consequences of clinical decisions based on prognostic estimates. One physician expressed the need for caution in the use of prognostic estimates in EOL care decisions because of the outcomes at stake (death due to the withholding or withdrawal of life-sustaining care):

Critical Care Physician 2: In the ICU there’s a very powerful self fulﬁlling prophecy of work which is: that if you prognosticate you have essentially ended their life, because you are dictating that aggressive care won’t be taken and if aggressive care is not taken early on, they all die.

This statement acknowledges deeper problems that can result from the use of CPMs in EOL care. The mortal consequences of decisions based on estimates of poor prognosis make the endeavour of prognostication ethically daunting. But they also pose the risk of inﬂating the apparent accuracy of this endeavour, and thereby reinforcing overconﬁdence in prognostic estimates.

Miller-2019

A few indicated they would want to get health services and/or talk with someone about sex during their visit. One person reported preference for speaking to someone over using an iPad.

“This has the potential to identify patients at risk who are uncomfortable providing honest answers face-to-face. It also deﬁnes various types of sexual intercourse that may not be comfortably (or completely) differentiated by some providers in the ED setting. It is thorough, and the same questions would be asked to each patient without concern for missing anything.

Concerns around HIV testing were similar to those in the literature – inadequate time for counseling, inadequate linkage to care, limited access to rapid test, maintaining patient privacy, and patient fear of testing.

Nelson-2020

One patient asked,“Those people who are broken by the idea of getting something scary like cancer, where do they turn? They can call and make an appointment, but that’s not going to help them feel better.

Nicks-2016

“In some houses, the parents owned the houses and they didn’t feel comfortable letting me poke around their house. And a huge factor is if the house is not kept and it is really dirty, they are embarrassed. So embarrassment would be a huge factor in a lot of cases.” (SNS-H program nurse)

Pope-2017

In common with frontline health care, we found that call handling required considerable

emotional work. Some staff reported the new system using the CDSS was highly stressful they described it as “frenetic and frantic” and described staff being in tears or visibly stressed (Interview, 999 senior manager). Call handlers had to establish rapport with each caller and manage the pace of the conversation. They also needed to translate what the callers were saying in order to make life-saving decisions:

Now, if mum’s saying they are floppy and lifeless […] we need that ambulance, but sometimes, an interpretation of floppy and lifeless, it might just be a listless, lethargic baby who doesn’t need an ambulance. How do you decide which one is life-threatening and not? You chat to the mum as much as you can […] you know, [probe] “is it like a ragdoll?” (Focus group, NHS 111).

Rapoport-2020

encounters with patients over the driving issue were emotionally fraught, and frequently posed a risk to the therapeutic relationship. This imparted a sense of immediacy to participants’ interest in the tool. Participants also described the need for a more objective, consistent, and standardized way of dealing with driving assessment, potentially making it easier for patients to accept the loss of their licence when this was deemed appropriate

Roebroek-2020

Several clinicians indicated that it became easier to discuss intimate topics because they were explicitly stated in TREAT. One respondent pointed out sexuality as an example:

“For example sexuality. That is not something you would immediately discuss, I mean you should of course, so that is my fault, but with TREAT, it is explicitly stated. Also intimacy. It therefore brings itself up, which makes you talk about it. So that’s an improvement.” [C4]

Shannon-2021

Physicians view technology as a helpful tool to start the

conversation about mental health with their patients
