# Supplementary material for: Stakeholder Perspectives of Clinical Artificial Intelligence Implementation: Systematic Review of Qualitative Evidence
Source: J Med Internet Res. 2023 Jan 10;25:e39742. doi: 10.2196/39742 (PMC9875023; doi:10.2196/39742)
Supplement: Multimedia Appendix 3 [file jmir_v25i1e39742_app3.zip › 2. Technology/2a. Material properties/2a.1 Usability of the tool.docx]

**Name:** 2a.1 Usability of the tool

Abdi-2021

many experts agreed that AI-enabled apps and voice activated devices are technically ready, have products available in the market and are already attracting interest from companies, research, older consumers and policy

“Would need extensive development from a reoriented user-led framework” AI-enabled apps, P10

Abejirinde-2018

Technical difficulties (software freezes, slow response time), and procedural issues (low user dexterity with operating the device) were two main factors that contributed to delays.

Ash-2015

Most hospital representatives purchase some content directly from content vendors and are often frustrated with the products. They are concerned that content contains too many options, is not current enough, or is not good enough (i.e., evidence-based).

We heard constant and strongly worded complaints from the clinical site representatives about the usability of all vendor-developed EHRs. We observed continuous usability problems, defined as difficulties experienced by the user directly interacting with the clinical information system, which can impact the effectiveness of CDS.

Beede-2020

This immediate gradability feedback is something that the nurses did not have before, and turned out to be frustrating as images they felt were human-readable were rejected by the system. Because of this, nurses somewhat questioned the power of the deep learning system. P6 said, “It gives guaranteed results, but it has some limitations. Some images are blurry, and I can still read it, but [the system] can’t.” P3 shared the same sentiment, “It’s good but I think it’s not as accurate. If [the eye] is a little obscured, it can’t grade it.” The system’s high standards for image quality is at odds with the consistency and quality of images that the nurses were routinely capturing under the constraints of the clinic, and this mismatch caused frustration and added work

If in one photo she was able to see the top half of the image ﬁeld, the nurse would take a second photo where she could capture the bottom half from the same eye (Figure 5). She would try to assess for DR using the top half of one image and the bottom half of another. They expected the deep learning system to perform this workaround as well; however, it couldn’t, because it requires one high quality image per eye, and cannot make assessments based on a composite from two images. “I want to be able to upload a second image with the focus on the macula. I want to focus on one area and crop it so I don’t keep getting an ungradable result,” said P7.

With poor lighting conditions commonly causing low-quality images, and many patients waiting in the queue, these ungradable images frustrated both patients and nurses. We observed nurses spending 2–4 minutes with each patient, retrying taking the photos a second time if the ﬁrst ones were unable to be assessed, but never retrying for a third due to the discomfort the camera’s bright ﬂash causes to patients. P6 expressed this concern, “I’ll do two tries. The patients can’t take more than that.” In the event that a nurse cannot capture a gradable image, the patient is to be referred to a specialist (potentially unnecessarily).

Chrimes-2014

Specifically, Phase II characterized the unpredictable aspects of use of the ADAPT that were not anticipated by the CDS designers and also identified potential disruptions caused by the ADAPT on “natural” provider workflow. Analysis of the “triggering” of the CDS and provider sequence workflow helped the development team understand previously unidentified barriers to integration of ADAPT with provider workflow and interaction sequences.7 Phase II testing uncovered new conflicting content issues with the workflows was not detected by Phase I testing. For example, the comment field box was hidden from view which contained extremely valuable contextual data. Additionally, the tool did not allow for more clinical discussion surrounding abnormal exercise or diet, such as patient comorbidities (e.g., arthritis or mental condition) that might hinder their daily activities

Connell-2019

Others pointed out that the added burden of the app was related to the volume of false positive alerts produced by the mandated NHS AKI algorithm:

...if the noise of the system could be reduced it would be a lot better. If [we were] able to get rid of all the nonsense alerts, that would be fantastic. [Respondent 1: Nephrology team]

Flint-2019

The focus group data (n = 3) (Table 3) reﬂect concerns about the strength of fentanyl patches

used in the CDS, the conversion factor to fentanyl patches, and questioned ways to improve safety of the CDS’s usability

Gance-Cleveland-2019

Users encountered a few problems when completing the

screening, including sensitivity of the touchscreen, navigation, and layout

Johansson-Pajala-2019

Maybe it should be impossible to renew any medication or make any changes at all before ticking a box showing that a drug review was made” (N2

Joshi-2020

Implementation challenges that emerged from the interviews clustered to technical build,

optimization of alerts, workflow integration, tool validation, implementation time, working with external vendors, and clinician acceptance (Table 2). These were shared by rule based and ML models alike.

Klarenbeek-2021

When professionals were asked how the CCDSS could ﬁt into existing MDTM work-

ﬂows, they requested a user-interface that was easy and intuitive with good readability of the data displayed by the system. Professionals also stated that the output must be correct, complete and up-to-date as the output serves as basis for MDTM decision-making.

Knoble-2015

algo/tablet, such as certain common chief complaints missing from the application. The headache and skin complaint algorithms were not available but were mentioned as needed. Chronic diseases such as hypertension, COPD and diabetes were also not covered. The tablet lacked a feature that allows for saving patient information to access for follow-up visits. The use of combination questions and some English terminology was also difﬁcult causing misunderstanding and resulting in wrong decision-making pathways. Technological complaints included the small size (7 inch) of the tablet, the insensitivity of the touch-screen and the lack of reliable electricity impacting use due to the less than optimal battery life

Lawton-2014

In some cases (n = 14), reliance on an advisor and/or unreﬂective practices of administering ‘‘whatever the wizard tell me’’ were later found to have had a detrimental impact on glycaemic control. This included, P2, who described recently attending a routine diabetes review appointment where, ‘‘they found out I was having readings of 20 and 25 [mmol/l] and they got in touch with [course educator] and she hauled me in and had a look at the machine and altered it [ratio], from 1:1 to one and a half to one’’ (P2.2). Likewise, P3, who had ‘‘always followed what it [advisor] is suggesting’’ described how, over a period of several months, he had had to ‘‘give myself an extra 2 or 3 [units] every other hour to try and bring it down late morning.’’ This problem was not identiﬁed and addressed until P3 attended a 6 month trial data collection appointment where, with input from educators, his morning mealtime ratio was changed.

Liberati-2015

«After integrating the electronic medical record we asked ourselves the question of how this technology could help us in our practice. Example: if I prescribe the antibiotic for pneumonia, it will never be for just one day, of course. We can teach to the electronic folder that does not have to ask me for it everyday? We can code the time X in such a way that the system gives me an alert only when this time expires? " (Internist, setting A)

Lugtenberg-2015

Irrelevant alerts for different user groups

•“It shouldn’t be necessary to override so many alerts; only the sections that apply to us [PNs] should be highlighted”.

Responsiveness of the system (loading of an alert takes too long) •“I gave up rather quickly because the loading of an alert took way too long”.

McDermott-2014

"Well I don't like it when you have to go through several sub-menus really…and a lot of them had more buried you know" (P04)

Morgenstern-2021

AI’s ability to use novel data sources for extracting meaningful public health information from unstructured data sources was considered a major advantage, that could supplement traditional disease surveillance. You know, our traditional approaches, analysis, regression, so on work with structured data and you can work with even large amounts of structured data[…] But when you’re talkin’ about data that’s coming, and, again, you can think of all these sorts of unstructured data from large numbers of people …

Nicks-2016

NHVs also reported several injury risks often seen in client homes that were not addressed by SNS-H. These risks included those related to having older siblings, older homes in disrepair, weapons, using the oven to heat the home, wood-burning stoves or furnaces, pet related injury risks, loose rugs, doors that children can open, and poorly lit staircases. Many NHVs felt strongly that to be effective, the program must comprehensively address the injury risks prevalent within the high-risk population served by the organization.

Orchard-2019

In this context, systems need to be very efficient and electronic tools need to work well, otherwise they add time. There were some reliability issues with the eHealth tools, which interrupted screening and undermined trust in the electronic tools. “When it works it’s great,and I love all the things. It’s just so unreliable…that unless you’ve got someone like me who really is willing to give it a go…[others] don’t have the time.” (GP, Practice E).

“[the prompt and EDS] wouldn’t work for quite a while, and then the phone wouldn’t work for some reason. It was fixed eventually.” (GP 1, Practice A).

Page-2019

Half of all problems described related to the speciﬁcity (n= 11; 26%) and sensitivity (n= 10; 24%) of alerts. Perceived speciﬁcity problems included the clinical accuracy of medication class categorisation used for allergy and DDI checking, DDI pairs and the local conﬁguration for DDI severity level.

Pannebakker-2019

Some compared the melanoma eCDS favourably with other CDS systems:

’It’s a lot simpler and quicker than QCancer. I’ve only used the QCancer once — I probably ought to have used it more, but it’s too long-winded to get your head around.’ (M, !51 years)

Patel-2018-additional file

Comprehension Main GP: It’s integrated well, and there needs to be still more work. Look it’s a tool, and it’s a tool which has very, very many uses, and quite a lot of potential

Porter-2018

Paramedics at both sites reported a variety of practical challenges to using CCDS as part of their routine work, such as difficulties keeping the PC charged:

So we know roughly that the laptop won't quite last a full 12 hour shift if you're using it, even if it's, um, docked in the ambulance on charge because it's only a trickle charge, it doesn't really do a full charge. (End S2 02)

Reynolds-2019

“I think it can [be useful] ... depending on how user-friendly the device is because if we’re in a rush, you have a child coding and it’s critical in the situation, depending on how hard the device is to use, it could be more cumbersome and more slowing you down than actually helping.”

“So we did the class the other day, some of the batteries were dead.”

“it is like a dinosaur ... it feels like it might have come out in 1987.. . there are much easier ways to do these things with apps such as the iPhone apps.

[Two nurses] say they do not use the ‘green machine’ because there are ‘way too many buttons’ and ‘it takes way longer than a calculator’.”

guess I wasn’t as comfortable using the keypad and so I would use the stylus and even the stylus wasn’t as quick I guess.

“Nurse 1 tries to double-check the dose using the pac2... However, digoxin per oral (PO) did not come up so she does it in her head.”

We basically are weaning the patient because the baby is growing and getting bigger, and the dose is staying the same.. . So, it will go, ‘alarm, alarm, alarm!’ You know, ‘dose is too low.’ And I’m like ‘So what?’”

The RAs noticed several instances when the nurses attempted to use pac2, but were unable—the device was inaccessible in the ward or was out of battery charge. There were also times when the device failed to print.

Sharpe-2019

The accuracy of the algorithm was not such that it could be relied upon or used without review by an electroencephalographer. An instant messaging alert function was used by only 2 of 9 study neurologists, others finding disruption from false positive alerts too disruptive to other work commitments and sleep.

Sun-2019

“When it comes to AI, there is a black box. […] It is difficult for us to solve the problem […] once diagnosis error issues appear. […] It is not like carrying out experiments, where we can see the process. AI itself is under learning. We don't know what is wrong if it [the AI system] has some problem. This is very dangerous!” [5GOV01]

This means that the AI system still needs to be complemented with the experience of human doctors. As mentioned by one of the hospital managers/doctors: “When we use Watson, the input should be structured data. We need to read the [medical image] picture and then input the data. […] Watson itself cannot read medical image data directly” [1HP03]. There is also awareness of this from the government policy-maker side: “Medical image data is quite subjective, AI cannot make judgements, at least so far. […] It requires a doctor's experience” [5GOV01].

Trinkley-2019

One clinician stated if CDS were ‘…more pro-active, preventative,…something that is not in that moment where you’re trying to just take care of the patient acutely, we could do more outreach and prevention’. Clinicians also expressed favourable views of checklists that could ‘default to fill in the [information] that the system already knew’

Wang-2018-Tables

I think if you can incorporate it (CARATV2.0) into the (practice) software and the software would populate as much as the data from your file as much as possible that would be very good. (G03
