# Supplementary material for: Stakeholder Perspectives of Clinical Artificial Intelligence Implementation: Systematic Review of Qualitative Evidence
Source: J Med Internet Res. 2023 Jan 10;25:e39742. doi: 10.2196/39742 (PMC9875023; doi:10.2196/39742)
Supplement: Multimedia Appendix 3 [file jmir_v25i1e39742_app3.zip › 2. Technology/2a. Material properties/2a.2 Lack of emotion.docx]

**Name:** 2a.2 Lack of emotion

Adams-2020

Some participants were concerned about the lack of human connection that AI may

imply and emphasized the need for “human empathy” and the “ability to understand with ﬂexibility.”

Biller-Andorno-2021

An AI- based support system generates predictions based on evidence, rather than on opinions ‘I also think that such tools are based more on facts than on opinions. At least that’s what I imagine, as emotions are probably not involved.’ (Interview 9

Understandability/explainability is a key issue for perceived reliability and trust. Knowing what the AI does helps figure out an appropriate distribution of tasks between humans and the machine. ‘It depends on how well I could understand how the tool works. I think it would be a good support. I would use the decision, or rather the hint, from the tool, as a prompter to read the chart or to have a conversation, whatever, to verify. If I know exactly ‘Ah, the thing does this and that’, I could tell myself, ok the tool has done this for me and this is what I still need to do […].” (Interview 8)

Blease-2019

It is unlikely that the human element of empathy and the subtlety of human communication and non-verbal cues can be detected by robots or machines. [Participant 517]

Technology will never be satisfactory as patients are looking for that interaction and dopamine squirt (doctor is the drug) which can only be achieved through empathic continuity of care of highly experienced General Practitioner [Participant 686]

Some suggested that there was an ineffable aspect to medical decision making that renders it an intrinsically human pursuit: Technology cannot replace doctors. There is definitely a 6th sense. [Participant 635]

Cai-2019

Due to the subjectivity inherent in cancer grading, participants desired to know from whom or where the algorithm received its medical source of ground truth. Whereas pathologists are typically aware of their colleagues’ medical experience and pedigrees, the clinical background of an AI can be opaque. Participants asked whether the algorithm had learned from diagnoses made by general pathologists, GU pathologists, or an entire panel of GU pathologists. Most felt they would not trust an algorithm unless it were based on judgments made by well-respected GU pathologists or institutions, explaining that expert consultation is how they typically resolve uncertainty in current practice: “I would send it to [name]. He has 50 yrs of experience in GU pathology and wrote [a critical text in pathology]. So you’re getting someone with a higher level of expertise to weigh in.” (P7) A few participants asked if the AI was based on an even more objective source of truth than GU pathologists, such as patient prognosis or immunostains. Overall, due to variability in grading, pathologists may not know how to assess the qualifications of an AI unless it is known to be based on an authoritative source of truth

Goetz-2020

The groups felt that the vPCP would not be inhibited by human error or emotional decision making, leading to a lower likelihood of mistakes.

“I don’t see any involvement of, like, emotion at that point, which is like something they tell to doctors. Like, ‘Keep your emotions away,’ right?” (Fourth year graduate student)

There was concern among the groups about the lack of a physical exam when using a vPCP:

“. . .the complete lack ofa physical exam. I wouldn’t be examined. My heart wouldn’t be listened to, my lungs wouldn’t be listened to.. the complete lack of touch, the complete[ly] lack of a physical exam would bother me.” (First year medical student)

Medical students expressed hesitancy over using a vPCP for mental health concerns:

“Well, I think difficult topics, difficult situations, more psych-related issues, things that you actually just want to talk to a person about, you lose the relatability. ‘Cause a lot of times, even docs, they are very relatable people.” (Fourth year medical student)

Conversely, students in the engineering/data science groups thought that a vPCP would be easier to talk to:

“Like, as a human, there is some point where you really don’t want to express everything to a human. . .without the fear of like, what does the other one think about you?” (Fourth-year graduate student)

Henshall-2019

Some psychiatrists felt that there was a danger that the DST could increase paternalism if the drugs listed on it were not perceived as clinically relevant, as this could result in doctors overriding patient preferences if they felt that the patient’s drug of choice was not credible. However, others commented that it could help to educate doctors about the efficacy and side effect profile of relatively unknown medications, increasing the likelihood of them being integrated into practice.

Certainly, there’s things there which I’m sure I’m going to learn that I will integrate into my practice as I go along. If there is a link for the evidence, that could be very helpful, actually, because every evidence needs to be looked at and can be discussed, debated, argued against. Psychiatrist

McCradden-2020

Before [the brain tumour], I might [have said] yes, because I would say … it’s the survival of the fittest. … But you can never underestimate the fight … in a person, even with a disease. And [a patient] can far surpass the expectations that are set out in these kinds of statistics. (Participant 18–001, patient

Melo-2020

Of course, one can think of the danger of dehumanization, but at some point in the process, there must be human intervention. Although AI can support healthcare, machines do not have the ability to mirror and interpret feelings

Morgenstern-2021-supplementary file 6

… it's this tool that’s just constantly scanning a whole bunch of online resources like newspapers in a bunch of different languages and health department notices, um, a couple of social media streams, just looking constantly looking for: do we see a blip? Do we see a pattern that's potentially suggestive of a disease event? So that's I think one of the, uh, the best developed systems out there. They're definitely leaders in the field. [Participant ID #6]

Rapoport-2020

Participants emphasized the importance of dynamics which were not assessed by the tool, such as contextual factors and nuances of the clinical encounter that no algorithm could take into account. For example, the fact that patients’ family members might be saying one thing but communicating another through their body language or general demeanour was considered to be important:

So the pure algorithm would have said increased risk, maybe suggest an on-road driving test. But all the vibes in the room are saying, please send the letter. So, there’s nuances which the computer is never going to have access to. [MD05-FP]

Shannon-2021

However, some patients express concern that technology is not as valuable as face-to-face interactions for creating behavioral and cultural change. As one interviewee stated, “You can rely on technology to improve or make a social change, but technology on its own is not going to allow you to make that social change that allows you to remove taboos…technology alone is not going to make that change, but it is necessary as a tool to achieve the change…” Technology is a useful supplementary tool, but may not be able to replace the patient-provider relationship

Yang-2019

They shared that the cardiologists were incentivized to implant more patients and to implant sicker patients. They found themselves often advocating for patient mortality (let the patient die). Mid-levels felt their opinions focused on post-implant quality of life. Unlike the physicians, mid-levels worked intimately “with all the problems that can come from a patient that maybe shouldn’t have been implanted.” They noted there was no right or wrong answer between length of life and quality of life.

They stressed that to understand a patient clinically, they needed to “look at the patient, talk to the patient, take care of the patient.” Social workers shared that they had not met with this patient nor talked to their family. In our field evaluation, presentations of the synthetic patient cases were always followed by a long, awkward silence. A very sick but highly motivated patient can do better than their illness would otherwise be left them, compared to a less sick, less motivated patient. These things are hard to capture. The eyeball tests. (Surgeon, B6)

Some voiced strong concerns that using DST was the same as applying “populational statistics” to individual patient decision making. They felt this was unethical
