# Supplementary material for: Stakeholder Perspectives of Clinical Artificial Intelligence Implementation: Systematic Review of Qualitative Evidence
Source: J Med Internet Res. 2023 Jan 10;25:e39742. doi: 10.2196/39742 (PMC9875023; doi:10.2196/39742)
Supplement: Multimedia Appendix 3 [file jmir_v25i1e39742_app3.zip › 2. Technology/2a. Material properties/2a.3 Large amounts of changing data.docx]

**Name:** 2a.3 Large amounts of changing data

Ash-2020

One of the strong points of (our system) is they gave us keys to the kingdom. And if you have the resources, you can set it up to make it bark and rollover and whatever you want it to, right?

Goetz-2020

Students also acknowledged that a virtual PCP has a much greater capacity for information

than a human physician.

“[H]uman brains are not really fully used. . . They cannot memorize everything. . . you cannot remember every single patient. . . what do they look like, what’s their disease look like, whatever. They may make mistakes. . . AI can record all the information." (Fourth year graduate student)

. . .when they would come out, they will be at the same levels or better than human. And as time goes by, they will only get better.” (Fourth-year grad student)

Joshi-2020

“We also decided how much of a wide or a narrow net...for looking at the surveillance. Would you want to catch…the widest net possible which would mean you'd have a lot of false positives or did you want to meet more specific in which case you would have less false positives but you might also have more missed opportunities.” (RB) “So it would be great, you know everybody wants this screening test that is perfectly sensitive and a confirmatory test that is perfectly specific but that is not always possible.” (ML)

“The biggest delay is usually anytime we have to make a change to the model, like recently we had to make a change to one of the parameters in this model because there are no values, and we didn't have the correct values for the parameter. So every time we make a change, we have to let it run for another 6 weeks in order to start using the data and to validate it.” (ML)

Klarenbeek-2021

This was seen as challenging because of rapid evolution in diagnostic and therapeutic options in lung cancer care.

For example, pulmonologists expected the second component (linking individual patient information with clinical practice guidelines) to have low impact on MDTM workﬂow and decision-making because Dutch national lung cancer guidelines were frequently outdated due to rapid development of new drug treatments.

Liberati-2017

it is essential that the CDSS is sufficiently flexible to allow frequent amendments.

We need to be able to update the CDSS quickly if we want to offer an optimal service to our patients. […] We need to be on top of this because nowadays the guidelines may change more than once every year. (Physician, setting C1)

Lugtenberg-2015

Lack of adjustability to personal preferences

•“The customization options are still rather limited. You should be able to turn off specific types of advices, for instance the ‘give up-smoking-alerts’ rather than all life style advices at ones”.

•“I wanna be able to set the threshold myself, so not all at 40 for blood pressure, for example”.

- Lack of learning capacity of the system

•“This almost asks for a system that can be overruled. You don’t want the computer stupidly, not intuitively, to state the same thing over and over again. In practice, that will result in overriding alerts. The system should cooperate with how people think”.

Also, a lack of learning capacity of the system was indicated to be a barrier with the system using only fixed rules rather than learning from the PCPs' use of the system and adjusting the content accordingly.

Morgenstern-2021

Like, basically if you look at that raw data every second of your day... There’s a record for that. Um, you can’t analyze that with traditional methods, and you don’t necessarily want to, right? So, you need tools [like AI] that will take one individual’s data [and] turn it into something meaningful. And then you’ve got millions of people’s worth of data. [Participant ID # 7].

Page-2019

Most respondents (65%) reported that alerts had remained unchanged from the original go-live conﬁguration

Patel-2018-additional file

Lead GP: you’ve just got an independent source and it’s new data so it’s just refreshing your minds because the guidelines are always changing, the targets are always changing, blood pressure targets, lipid targets, so I, yeah, just brings everything, gives you a fresh review of everything, and it’s on your computer, it’s on your desktop.

Petkus-2020-supplementary file

As long as the CDSS is built incorporating regular monitoring by a responsible group of professionals, appraising developments in the field to ensure safe evidence based practice, there is potential for it to work for the benefit of patients.

Pope-2017

Moreover, training was not a one-time activity: there were continual updates to the

computer system prompted by new evidence guidelines from NICE, changes to contracts for services, and larger policy changes and adaptations to improve operability. In one site, we observed an additional afternoon of training introduced when a single national safeguarding policy changed.

Roebroek-2020

Several clinicians experienced the suggestions as generic and comprehensive, sometimes even too comprehensive. Not all recommendations were suited for the clinical complexity of the patient, or had already been tried before:

“The treatment recommendations are sound but you always need to tailor them to a specific patient or circumstance and see if they still apply.... It’s difficult because sometimes certain recommendations from guidelines have already been tried or are not applicable anymore.” [C2]

Torenholt-2021

During an interview, Henriette questioned the algorithm’s complexity, referring implicitly to its fixed thresholds and the lack of machine-learning capabilities, something that she as an outsider considered the hallmark of complex algorithms.

Yang-2019

They recognized the value a DST might bring from its statistical consideration across many cases. “The value is you are looking at thousands of cases, I’m looking at 100 and overweighting the last three I saw.”
