# Supplementary material for: Stakeholder Perspectives of Clinical Artificial Intelligence Implementation: Systematic Review of Qualitative Evidence
Source: J Med Internet Res. 2023 Jan 10;25:e39742. doi: 10.2196/39742 (PMC9875023; doi:10.2196/39742)
Supplement: Multimedia Appendix 3 [file jmir_v25i1e39742_app3.zip › 2. Technology/2b. Knowledge to use/2b.1 Knowledge required of patients.docx]

**Name:** 2b.1 Knowledge required of patients

Abdi-2021

Moreover, one of the main reasons for agreeing on the potential of IoT enabled homes and AI-enabled wearables was their ability to collect data from older people non-intrusively

Abejirinde-2018

The ability of providers to clearly explain the device function and help women understand its benefits facilitated a positive disposition to B4M.

“I would say for us here, when you hear that you have a device that is going to help you do something, they get excited…. some (women) get excited, but others…. it is the explanation that you have given them of what (the box) can do so that they can accept it.

Observations showed that not all women received a full explanation about the device, its functions and the procedures for the screening.

Abidi-2018

Participants overwhelmingly stated that they would like some kind of technical support, such as online or face-to-face sessions on DWISE, to teach them how to use DWISE

One of the patients said:

I am not technologically adept, these are new and exciting...I like help with managing my diabetes...but there might be big learning curve for me. [Patient]

Alaqra-2020

Doctors do not necessarily want to be experts in encryption, or understand the process fully. However, most stated that it is important that they have a general level of understanding that satisﬁes them. They also needed to know that the privacy-preserving data analytics process on encrypted data is safe, of clinical value, and is reliable. Clinical staff want to know enough about the privacy preserving process so that they are able to answer questions from patients, even though questions by patients may be rare

When communicating and informing patients about their data protection and technical details, most interviewees thought that it is necessary to have information available to patients. Doubts on whether patients will ask or understand the information were raised by clinicians.

Andrews-2017

Getting older patients to engage with technology at all was seen as a challenge by an occupational therapist.

P7: I think in theory it’s a good idea, I think trying to get people to engage with that, may not be so easy, just because of the technology.

She suggested that some patients did not engage well with it because they did not understand or feel comfortable with the technology.

P4: Some people, I found didn’t like it, just didn’t get on with it, and that just, everybody’s different, everybody’s an individual with what they can cope with and what they can understand, and I think it’s the approach really, with technology.

Some participants expressed how, in their experience, it was the set-up of new technologies that posed the greatest challenge.

P1: they might say, I like to, you know, watch videos on this, but my family helped set it up. It’s the practicalities of setting it up and once they know how to do it, it’s a bit easier.

Researcher: Do you think that you’d have time to train someone to use that sort of, say a very simple app? P7: I don’t see why not. It might be that the initial assessment would be extended, but if that was part of it, then that’s fine, do you know what I mean, it would just be included and there’s no reason why it couldn’t be.

Researcher: Would there be similar sort of periods of time where you could see that fitting in? P2: Yeah, I don’t see why not. Researcher: Could you give any examples or perhaps describe a bit what that might look like? P2: Well I could be talking to a patient about how they feel, which is what I tend to do all the time anyway. And if they’ve got something in front of them they can answer in it in that way rather than just talking to me.

Ash-2020

So, that would not be hard. We create [electronic] questionnaires all the time so it would be very easy to create a questionnaire. The difﬁculty would be the education as always

More than half of our patient population speaks a ﬁrst language other than English so … we translate almost every patient material into our three most common languages … although there are hundreds of languages that our patients speak.

Beede-2020

Given that the deep learning system was deployed in an observational, prospective study, it was critical for nurses to obtain patient consent prior to using the system. The informed consent process was the ﬁrst challenge we observed, and was made more complicated by the need to explain the deep learning system.

Benda-2020

Several participants identified the patient as a stakeholder who should receive information to justify why they were receiving specific services. Physicians were also seen as playing a crucial role in endorsing interventions and introducing them to the patient. The provider’s endorsement certainly helps with the care management .... There has to be a conversation with the patient around there is a need here and the care manager is going to help us .. . improve your health.” – OPS19 [Facilitator]

Clear communication about the HNHC information to the patient could address a challenge, which was that a patient’s preferences regarding where to seek care represented a possible barrier to high-quality care.

If my patient is at home and chooses to go to the emergency room, how am I going to stop him from high utilizing? If I’ve made him aware that he can come here and see me .. . – EU04 [Challenge]

Fan-2021

In other cases, users complained that the provided information on the diagnostic report was overwhelming. Indeed, due to different levels of health literacy, knowledge, and experience, users may have had challenges in comprehending the technical aspects of the diagnostic report (eg, medical jargon and professional medical knowledge)

Haan-2019

A prominent result from the interviews is that patients’ views on radiology are diverse and sometimes incorrect. For instance, to patients it is not clear what the differences are in roles and responsibilities of different staff members (radiologist, radiologic technician, nurse, or doctor's assistant) at the radiology department. With respect to Al in general, patients noted either no particular associations or mentioned factors like “loss of jobs,” “making life easier,” or “what need do we have for that?” Patient education may be necessary to increase patients’ acceptance of and input on how to best use Al systems in radiology.

To form opinions on the use of AI in radiology, participants noted that it would be important to understand how AI would be used precisely. Patients express uncertainties about who is involved in the procedure at what stage when implementing Al. To them, the roles of the radiologist, the radiology technicians operating the scan equipment, and the referring physician in relationship to the use of Al are not completely clear.

Jackson-2017

Delivery of IBD education was thought to be important and necessary to maximise the efﬁcacy of the tool. Access to information regarding chronic disease prevention was thought to provide important additional education to both patients and clinicians.

Keogh-2019

Consumers were concerned about women's interpretation of the risk estimate in two ways; ﬁrstly, that some women may disengage from BC prevention measures if they feel reassured by a low risk estimate; and secondly, that any level of risk has the potential to provoke unnecessary fear

Lai-2020

Some interviewees said that it was necessary to avoid rejecting AI out of hand. They felt there was a need to build an operational definition to help people understand what AI can really do for patients. Thus, the education of the public has to be considered upstream, for example concerning the requirements of data (Which data? What standard of quality? etc.), as well as the need to step back and understand that AI does not possess the absolute truth.

Lawton-2014

Only a minority of participants (n = 3) reported having

independently altered their ratios since their courses. In most cases, as P2 or P3 reported above, if changes had needed to be made, these had not occurred until a routine diabetes review or trial follow-up appointment. In most cases, participants implicated a lack of conﬁdence, poor analytical skills, and/or deferential attitudes towards health professionals to account for not considering or making any independent adjustments: ‘‘I suppose I’m kind of subconsciously waiting for somebody with more expertise to sit down with me and suggest these changes’’ (M13.2); ‘‘I’m the kind of person that, as I said, I don’t like to do stuff on my own, I’m afraid in case I do something wrong and I don’t want to go hypo’’ (P24.2

Some (n = 6), for instance, reported not knowing how to change the settings on their advisors, and, hence, described leaving their ratios unchanged until they received health professional input. Others (n = 8) shared a misperception that, by virtue of being pre-programmed into their advisors at the time of their courses, their ratios and targets would never need to be altered: ‘‘well, it’s permanently programmed into the software . . . so I’d just assumed that everything would stay the same’’ (P27.2); ‘‘I haven’t ever changed it [ratio settings] because it was set up for me and I thought that was it’’ (P42.2). Hence, when these participants did identify or attempt to address problems with their blood glucose readings, they focused on physical activity patterns or on altering background/basal doses: ‘‘it’s your basal’s that going to have to be tweaked. . . cos your bolus, I don’t really think you have to tweak’’ (M18.2). Poor recollection of ratios and/or targets by virtue of them being pre-programmed was also implicated by some individuals (n = 11): ‘‘they’re [targets] not in my mind, they’re programmed into the machine, hence I wouldn’t know what numbers are [in order] to change them’’ (M14.2)

Lennox-Chhugani-2021

Women were divided on whether or not they would want to be informed if AI tools were being used as part of the breast screening process. However, they agreed overall that women should be given information about the role of AI in breast screening as part of the process of informed consent when taking part in the breast screening programme

McDermott-2014

Some GPs reported that they would have liked to use the prompts if they were available in additional languages.

"If it’s just in English it's not going to be useful specifically for us…um for our patient population Urdu or Mirpuri" (P01)

Miller-2019

Respondents also had concerns about patients not answering honestly and challenges due to limited health literacy and/or English proficiency.

Nicks-2016

While some NHVs felt that their clients liked the personalization of the printed educational report, the of NHVs using SNS-H felt the printed educational report was not effective in their client populations, noting that clients receive copious amounts of paper, and many struggle to read (Table 3, Item I).

Orchard-2014

However, patients generally had a poor understanding of AF and the aim of the screening. This is likely to be related to how thoroughly it was explained to them prior to the screening. • ‘What aspect of the screening process did you find useful? Well I don’t know what I did’ (Patient 3)

Once patients found out they did not have AF, they disengaged with the screening process. Many patients had other more pressing health concerns and were more focused on these. • ‘I said, “Oh God, me old heart’s not going to close down" but he didn’t think there was anything wrong’ (Patient

Orchard-2019

While the iECG device was quick to use, often extra time was required to explain the device, screening process and rationale to the patients took extra time, particularly compared to pulse palpation.

“I could take a pulse for 30 seconds or I could spend 3 minutes explaining to them the device and where to put their fingers and telling them what we were going to do. That’s the difference…as far as time use.” (Nurse, Practice H).

“It took 5-10 minutes because it meant you did a lot more talking about preventative stuff, explaining things”

Rapoport-2020

We work in a shared care model so I find it’s very time-consuming. I can see that adding more time and trying to walk someone through a tool like that … slowing me down even more. [MD12-SP]

Roebroek-2020

Some had cognitive problems and were easily overwhelmed by the complexity of the data presented in the application:

“I noticed that if patients are not able to process a lot of information at the same time or if they are very much stuck in their own line of thinking, TREA T’s systemic approach doesn’t really work that well.” [C2]

Shannon-2021

Another challenge from the patient perspective is that some of the older patients in the study experience difficulties with using the technology. It is more difficult for them to use the app, and therefore they are less likely to engage with it. For

Young patients are more easily able to take these technologies, and use them, and handle them, and understand them, but an old person does not, and some of the people who have come here in consultation are elderly

Torenholt-2021

The physician appeared very dedicated and thorough in her work, and attentive towards the patient’s understanding and any questions raised. When asked why she did not mention or explain the automated screening, the physician said:

The ones who are experiencing symptoms which we are worried about, we get to call those (. . .) But you’re completely right, I consistently didn’t do it [mention the algorithm]. But I don’t believe there’s anything to hide, absolutely not, at any time I would [explain it] if the patient asked. There are a few who have asked about it; ‘How do you do it?’ And then I’ve told them. In that sense, I don’t think there is any jiggery-pokery in it.

Researcher 1: [addressing IT-developer] We still need those automatic response emails to the patients, which I consider rather important. And they should come at least (. . .) 24 hours after [the patient has responded], [and say that] ‘We have now checked your responses, and everything looks fine. If anything appears before the next control, you are welcome to contact us.’

Physician: Physician: Why do you say minimum of 24 hours after?

Researcher 1: Well because, well it just seems a little (. . .) sometimes it’s just a little, you know, impersonal. [Imitating a patient:] ‘Then there’s no one who has checked this, they just send an automatic response. . .’

But we’re not going to lie to people, right? We shouldn’t make patients believe that we’ve been sitting and looking at their responses. I suppose we can say: ‘There is nothing dangerous [indicated] in your responses, [so] you should not expect to receive a phone call,’ without suggesting that we’ve studied their response and thoroughly discussed it.

Researcher 1: Well, it’s that thing, when you introduce machines [i.e. computers] like this between the practitioner and the patient (. . .). You’re of course right; we should be honest about what we do.

Researcher 2: But they’ll know [that it is based on an algorithm] if they receive a response right away.

Researcher 1: But maybe you should be very clear about that, when you inform [the patients] about the project.
