# Supplementary material for: Stakeholder Perspectives of Clinical Artificial Intelligence Implementation: Systematic Review of Qualitative Evidence
Source: J Med Internet Res. 2023 Jan 10;25:e39742. doi: 10.2196/39742 (PMC9875023; doi:10.2196/39742)
Supplement: Multimedia Appendix 3 [file jmir_v25i1e39742_app3.zip › 2. Technology/2b. Knowledge to use/2b.2 Enabling users to evaluate tools.docx]

**Name:** 2b.2 Enabling users to evaluate tools

Alaqra-2020

a couple of participants mentioned that research publications proving the soundness of PAPAYA’s analytical methods could be made available to interested stakeholders.

Benda-2020

Lastly, participants heavily emphasized the importance of transparency in building trust in the predictive algorithm. Specifically, they wanted high-level summaries of the included variables in the algorithm to help them understand why patients received a particular score. One of the big questions that comes up is like how did you get to this number? And then typically ask for like the big details. If there’s detail on what went into it, for sure it’d be a confidence booster. – EU01 [Facilitator] I think the best predictive models these days are black boxes ... you can tell people what most important set of variables are, but not tell them how they’re actually ... used. Because it’s too complicated. – EU04 [Facilitator]

Cai-2019

To understand the AI Assistant’s likely capabilities and limitations, some participants desired a summary of the volume and types of clinical cases that the algorithm was created from. However, participants felt they did not have a benchmark for evaluating what volume of data would be adequate, or what scale is reasonable for machine learning: “I am not an AI expert, so I cannot point out the number.” (P16) Some suggested that the number of data points should be on par with the volume of cases pathologists are typically trained on, with some feeling that it would need at least enough cases to have observed the “rare variants that we come across once every 4 or 5 years.” (P13)

The desire for high case volume may reflect a desire for generalizability. For example, one pathologist pointed out that having data from diverse sources would be more representative: “More variation is better...Covering from community hospital small groups, to academic medical centers, it’s more representative.” (P16) Another wondered if an AI that is “geared toward a certain type ofstain” could generalize to stains at other institutions: “Our staining is really bad and there are days when the stain is faded...In those circumstances, and with different variables, how good is that system going to be?” (P13) Overall, providing users a sense of the diversity of training data could help inform generalizability. However, numerical metrics about case volume may need to be accompanied by benchmarks to give users a basis for what is reasonable within the scale of machine learning.

As they discussed different means of assessing the capabilities of an AI Assistant, participants also expressed a desire to obtain a basic understanding of the ultimate limits and capabilities of AI in general. As an example, one pathologist described the human ability to have an inkling or “sixth sense” that cannot be rationalized, and desired to know if AI could theoretically ever capture such a human instinct: “Are there certain things that are natural limits to the technology that could never be supplanted by AI? Or is it just my human egotism? Is the sixth sense an illusion? ... Maybe I’m romanticizing it or I’m being delusional.” (P18) Others reasoned that the AI couldn’t possibly capture elements that are imperceptible to the human eye, such as proteins that currently can only be detected through staining techniques. Still, some maintained that there are no limits to what an AI can learn, so long as there is sufficient data to learn from: “As long as they learn enough and can correct itself enough, with enough data, then it can be perfect.” (P16) Although the theoretical bounds of AI can be challenging to identify given that the field is still evolving, onboarding materials could at least offer the current state of knowledge, to set realistic expectations.

I want to know if the AI is being generated off of one image or if it’s being generated based on sequential images – the levels. Sequential I would trust more.”

This lack of an understanding of how the system arrives at a decision led to the desire for an

AI primer: “Ifsomeone can explain in a simple language, this is how it does it...so that intellectually we can understand what’s going on in [the] AI’s brain, and compare to our brain.” (P1) Participants contrasted this tool to other clinical technologies for which they typically already have a conceptual foundation through years of residency and training: “When I bring on a test, I usually know what method it is. You tell me AI, and I have conceptually no idea.” (P17) As a result, pathologists wanted to get a basic crash course in using AI, with some even acknowledging that such a course would be an essential prerequisite to practicing modern medicine: “Generationally, it’s going to be one ofthe more important ideas that’s happening. How it works, just a primer.” (P18)

one pathologist explained how knowing an AI’s over-sensitivity to benign mimickers could help them discount its opinion in those situations (“I would earmark that...Say it said ‘look at this’, I would say, oh it would typically flag atrophy which is benign.” (P18)). Conversely, knowing that the AI had additional context could increase reliance on its opinion, in cases where that context matters (“If it looked at immunostains, then it has more information than I do. That would make me trust it more."). Overall, understanding the AI’s strengths, limitations, and functionality could be critical to reaching a decision resolution when their opinion differs from that of the AI.

Catho-2020

TI_05 (M, senior physician):“They give you a false sense of security. The risk is that you accept the CDSS proposal without even knowing what you’ve accepted”.

Dikomitis-2015

Some GPs referred to the original research to question its robustness, for example, they queried why the eRATs stored data for only one year, when the original research was based on patients’ records over two years.

Gillan-2018

There were a number of potential downsides to AI quality acknowledged by participants, such as the concept of AI as an untested and unverifiable ‘black box’, though MPs tended not to raise these concerns. RO01 worried that ‘… someone has to know what has gone into the AI models and what’s going into the machine learning… because, you know, crap in crap out… there’s a lot of algorithms and black box, and some of them are crap even though it looks good’

One physicist, MP05, raised the possibility of MPs needing to interact more regularly with patients at the treatment unit, given the propensity for AI to impact on treatment decision-making. This would require patient care considerations, not currently a focus in their training. The risk of losing certain knowledge and skills related to treatment planning was also a concern, in that, ‘with more automated planning…. the fear is if we don’t teach the next generation the concepts, they’re going to have no idea if they encounter a problem, how it’s supposed to be fixed…. You need to be able to troubleshoot’—(RO03). Finally, all groups referred to needing to be equipped with an understanding of the principles, functionalities and limitations of AI, in order to work responsibly with it in the clinical context. Some argued for the benefit of an appreciation of basic coding, the principles of big data and machine learning and/or related algorithms. TP06 referred to this as ‘technology literacy’. More specifically, from RO01, ‘You need to have more awareness of automation and computers and algorithms and possible solutions’, and from TP01, ‘you need to learn the basics. Just like coding… and understanding what’s going on, so that you have… the tools…. You don’t need to know how the car works, exactly, you just need to know the general thing’

Goetz-2020

Finally, the students urged transparency regarding the data source used by the vPCP. They

would want to know who was developing and implementing the system, the data that was being used to train the vPCP, how the vPCP was tested during development, and how their data would be used:

“Knowing who designed it and whether it was a physician or a group of physicians or a hospital. . . what dataset did they use. I’d want to know more about that.” (First year medical student)

Grau-2019

Internal Medicine, female: What are the long-term impacts on the things that we implement to help patients quit or there’s the medications or it’s the referral, how effective are they? Honestly, I don’t know, but I am curious.is it effective?

Joshi-2020

I am not so thrilled with the predictive model. I don't understand…it's a big black box…. I don't know who built it, I don't know what state, the four hospitals are mystery hospitals.”

Jutzi-2020

Further perceived problems that were mentioned were the non-traceability of the decision algorithms and the missing transparency of the applied systems

Lai-2020

Thus, if physicians were obliged to use AI tools, they are very open to training to better understand how they work. They also believe that society will only accept mistakes from a machine if it understands why such mistakes may occur. For example, there are master’s degrees in the field to help future doctors understand how AI tools work

Liberati-2015

[SSDCs are not most viewed as an omnipotent tool, but also as a human product, to trust which it is necessary to know the designers and "controllers" (see quotation marks), to whose work to give legitimacy timidity. In this positioning, the issue of trust cia may also invest the technological component ca of the system. It too is seen as an art-fact produced by the expertise of a le community whose skills do not naturally appear to be compatible with those of the clinical community. For overcome the distance seems necessary to be reassured about the reliability of the designers of the systems and about their competence and proximity to clinical practice.]

[integrating SSDCs seems to require a process of legitimizing its sources e of the instrument. According to a medical director involved, this goal could be achieved through: 1) the presentation of the, of their scientific authority and rigor methodological, as well as the absence of conflicts of interest; 2) an expansion of possibilities participation in scientific committees you can select and filter the sources of evidence; 3) attention to the "transferability" of evidence international relevance to the local context]

Liberati-2017

Who controls the controller? I would want to know who puts the evidence into the system, to be sure that it’s reliable. [...] We used to think that medicine should be evidence-based and not authority-based. I think we need good authorities to help us selecting the best available evidence.” (Surgeon, setting A)

Morgenstern-2021-supplementary file 6

It takes out bias, right. I believe it takes bias away, you teach it to not have bias. [Participant ID # 9]

Petkus-2020-supplementary file

“The standards and testing metrics as well as test results should be open and peer reviewed, just like drug trials. Would also suggest that a register of such devices / systems should exist as for clinical trials, with easily accessible and understood explanations of the reliability / validity etc”

Sun-2019

IT firm managers, however, minimize the challenge posited by the opacity of AI algorithms. They argue that there is no reason for AI users to have an attitude towards AI technology that is different from other popular technologies, of which the inner workings are similarly unknown to users. As stated by one of the top managers of IBM China: Doctors don't know the principles of machines such as CT [Computed Tomography] or MRI [Magnetic Resonance Imaging] either. But they are not afraid of them. It is not necessary for them [doctors or other users] to open the “black box” of AI. What is necessary is to make an evaluation standard of AI's performance. Then, they will feel safe. [2IBM02

Van de velde-2018

Insight in how the CDS is triggered is desirable in case GPs have doubts about the CDS.

Watson-2020

The performance metrics used to evaluate the myriad of ML tools are less familiar to most clinicians. Also, the tools themselves are sometimes “black box” algorithms that cannot be fully dissected and deconstructed even by experts. This, according to some interviewees, has led to healthy skepticism while sometimes limiting clinical

Yang-2019

It took a long time for us to explain the data source and the ML mechanism to clinicians with no ML experience and without a deep understanding of statistics.

Clinicians commonly expressed a need to know more about the model’s source and credibility. When they learned that the model presented has not been rigorously validated through clinical trials and published in prestigious clinical journals, they suggested we were wasting their time. Physicians and surgeons considered discussing an unvalidated model unethical; as misleading as “looking at a crystal ball”. Others tended to judge DST quality based on the journal it was published in.

Physicians also desired a model that had been validated with data from their own hospital. “It’s better to be homegrown.” Models should be published in a good journal and then validated in a national scale study across several implant centers. Some suggested including links to the peer reviewed clinical trial within the DST output on the slide. It “lends a lot of weight to a clinical model”.

ML systems make predictions based on covariance of features. They do not assess the causality of those features. When prompted, clinicians claimed that this distinction is “absolutely important”. However, in our conversations, we did not observe them distinguishing ML predictions from general statistics. They seemed to strongly believed DSTs should be able to distinguish causality from prediction and that they should present only causal features. “This is the whole point of statistical processes. A DST model should address that, right?
