# Supplementary material for: Stakeholder Perspectives of Clinical Artificial Intelligence Implementation: Systematic Review of Qualitative Evidence
Source: J Med Internet Res. 2023 Jan 10;25:e39742. doi: 10.2196/39742 (PMC9875023; doi:10.2196/39742)
Supplement: Multimedia Appendix 3 [file jmir_v25i1e39742_app3.zip › 2. Technology/2b. Knowledge to use/2b.3 Agreeing the scope of use.docx]

**Name:** 2b.3 Agreeing the scope of use

Ash-2015

Customers, they believe, often expect the software to do more than it can, but use of these products is often suboptimal. A content vendor employee said: “And I’ve seen several times where the client is trying to make the products work differently… to meet their ideal workflow in their mind and they’re fighting the product”.

Ash-2020

IT staff at all sites felt that, if analysts are given the information in the KR describing the content about asthma, back pain, and diabetes, the CDS could be built on site. In fact, interviewees felt they were underutilizing the CDS development capabilities offered by their EHRs: “they (the vendor) gave us the keys to the kingdom, and if you have the resources, you can set it up to make it bark and rollover and do whatever you want it to.”

Cai-2019

Without the ability to see the “bigger picture,” participants felt it was inherently limited in its abilities: “It’s unfair for AI because it just does whatever it was set up to do, so it doesn’t get a chance to get an overall thing ofthe big picture.” (P14)

would be important to make explicit to users what input the AI does and does not have access to, particularly if it takes in more information than what is immediately shown in the interface.

Dikomitis-2015

The second component, the interactive risk calculator, appeared to be used less frequently than the other two components, which might be related to the limited access to training in the use of the tools, resulting in a lack of awareness of all the tools’ functions

An important factor that emerged from our data was GPs’ desire to understand the research underpinning the eRATs:

‘You wouldn’t really use it without knowing what or how it was developed, why it was developed, and what it was for’.

Our data suggest that user acceptability and usability of the tools would have been enhanced had the training been more comprehensive, accessible and appropriate.

Goetz-2020

They also theorized that the vPCP could be easily misled by a patient’s misunderstanding of their own symptoms, as their reporting to the vPCP may not always be clear and straight forward:

“So, if I’m not aware ofwhat I’m going through, I might [put something] wrong into that, I might enter that I’m dealing with some other disease.” (First year graduate student)

Grau-2019

Participants believed that providers would make better use of and more informed decisions about E-STOPS with further training about the functionality and navigation of both the EMR and E-STOPS. As participants discussed their E-STOPS use, it also became apparent that many were unclear about how to navigate the alert and order set or about the results of their actions. Participants often inadvertently dismissed alerts and were unable to later retrieve them. Rather than their action being based on any conscious decision, they were often unaware of having ordered QL referrals when they accepted E-STOPS in an attempt to prevent future alerts each time they entered patients’ medical records.

Hallen-2015

Physicians also expressed concerns that CPMs may promote prognostic overconﬁdence – that is, excessive prognostic certainty – about future events on the part of patients. They worried that patients would not be able to appreciate limitations in the sensitivity and speciﬁcity of a CPM and the applicability of prognostic estimates to individual patients.

Pulmonologist 3: So – I worry that too much speciﬁcity in the information given will lead to misunderstandings on some part of the patient or family. You know, you tell them they have a 40% chance of living the next 6 months they go, oh, I’m going to die in the next 6 months and they go and sell all their belongings.

Jacobs-2014

“MDs sitting down setting individual pt thresholds (if need be)

and then all MD sticking to these as they rotate on and off service.”

Johansson-Pajala-2017

Although some RNs expressed that it took some time to use the CDSS, they could see advantages, both in relation to previous routines and for the patients. The required time was also related to the degree of knowledge and capability of using the system, as some RNs experienced it a bit complicated. ‘From the beginning I thought, oh help one more thing we must do.. ..we have so many registers to ﬁll in.. .but that was only in the beginning, I can see the advantage with it’

Joshi-2020

“I think if you sort of set the expectation that this tool is going to come in and find sepsis for you, you are going to set yourself up for significant disappointment.”

Knoble-2015

Initially, they stated some difﬁculty in learning how to navigate the application but after a few days of practice, the HCWs found the device easy to use

Lee-2015

[clinicians] logged in one day and then it [CDS] came up. So my [clinical] colleagues were saying: “Oh I got to go through this extra thing, where I have to push some buttons before it lets me do this.”.. I think that the biggest challenge was that most clinicians I’ve spoken with have this perception that it’s just another thing that they need to do before they can order it

Other radiologists expanded on this idea, stating that educating referring clinicians about how CDS could help them and their patients is integral to the success of CDS. Some radiologists raised concerns that if clinicians do not recognize the clinical value of CDS and only view it as a barrier to ordering, some clinicians would respond by “gaming the system” to obtain higher appropriateness scores. In essence, several radiologists raised concerns that referring clinicians would not take appropriateness criteria and scores seriously without greater insight and understanding

Lugtenberg-2015

Examples of perceived barriers related to knowledge regarding the (specific functions of the) CDSS - Lack of knowledge regarding basic functions

“I have no idea what this grey button [manually to be requested alerts] means. It used to have a color and now it’s grey so I think something is wrong”.

“I didn’t even know there was a feedback option, never heard of it before”.

Lack of knowledge regarding personalization functions

“I had no idea about all these options! Now, I’m a lot more enthusiastic. I’m gonna use it right away!”

Mozaffar-2016

We also found that in many sites, delays were caused by a lack of appropriate user training.

But we know we have to do that quite carefully and with the appropriate training and support and systems in place otherwise we could be introducing significant risk to the patients. (Site C, Clinical Effectiveness and Medicines Manager)

Muth-2016

9/10 reported positive experiences using the CDSS (‘it is clearly structured, it is well-arranged’; ‘I liked … the weightings (for alerts)’), 1/10 did not (‘I did not feel comfortable with this programme…because I did not completely understand it’.)

Patel-2018-additional file

GP used HT routinely for screening which became routine. Other components of HT were not used to their full potential due to unawareness and lack of confidence in tool.

GP was not familiar and comfortable with all the features of HT and only used the traffic light assessment prompts and heart-age projection graph. He does not completely understand absolute risk % and therefore does not use to explain to patients. Further, he was not familiar with CAT or IF portal in relation to intervention.

Part-time GP has reviewed reports that main GP has given her however does not motivate her because not aware of the bigger picture of the study. The reports informed the GP they are an average practice.

For the main GP, the - ‘What-if” graph – he only showed the graph and didn’t manipulate since didn’t feel comfortable with explaining rationale. The graph was engaging for the patients.

All staff believe that this intervention can improve quality of care. However many are not aware of the full capabilities of the intervention.

Wang2020

I don't fully trust the tool to identify codes. I haven't been told if it is supposed to highlight knowns or not so when I see a known not highlighted I question if the tool is working correctly.”

Yang-2019

Are the Predictions Individual Medicine OR Population Medicine? Most clinicians share that they thought of DST output as an “average”. They seemed to find the notion of personalized predictions difficult to grasp.
