# Supplementary material for: Stakeholder Perspectives of Clinical Artificial Intelligence Implementation: Systematic Review of Qualitative Evidence
Source: J Med Internet Res. 2023 Jan 10;25:e39742. doi: 10.2196/39742 (PMC9875023; doi:10.2196/39742)
Supplement: Multimedia Appendix 3 [file jmir_v25i1e39742_app3.zip › 2. Technology/2c. Knowledge generated by it/2c.1 Communicate meaning effectively.docx]

**Name:** 2c.1 Communicate meaning effectively

Abejirinde-2018

“(I like) every part of the machine. I like it because the machine will tell you the right BP…. and then it will tell you the types of disease or the problems you are finding in your system

“Because I saw that everything, everything was ok. But that place (i.e. another health facility) I can’t see. But here, everything is plain and I will know that… I can see it. I see what are the problems, what they are advising me on and other things.”

Adams-2020

Participants emphasized the importance for results to be explained in ways they could understand.

Participants emphasized the need to fully understand their imaging results so that they can be engaged in their care and have more productive conversations with their physicians.

Andrews-2017

Using technologies familiar to older adults was seen as a way to overcome some of the barriers associated with implementing technological interventions, for example using text messaging.

P4: text is a really good way, by phone, and they feel quite happy with it, that they’re not struggling to listen, and they can sit and read, and if they can’t read it, they can go to the neighbour and go ‘what does this mean then’.

Ash-2020

So the decision support in that case should link to things like the resources that are available.

They don’t like pop-ups. They get pop-up fatigue. You start throwing too many things up in front of them they just hit them and go away. Go away

Baysari-2017

Prescribers were generally very positive about pre-written orders but explained that in some cases there were large numbers of orders available for a single antimicrobial and this led to unintentionally selecting the wrong order. Um, sometimes there are a lot of options and so I don’t think I’ve personally done it but I know my colleagues have accidently clicked the wrong dose just because there are a million different regimens or dosages... I’d hate to see the quicklists [pre-written orders] go; perhaps you could take out a few. (D7) For example, ceftriaxone, there is an enormous list of quicklists [pre-written orders] and you sort of have to scroll through to find the one that you want.

Beede-2020

We found that fundus images were a deep part of the nurses’ practice, and it became clear that the images need to be prominently displayed alongside the DR prediction. Displaying the fundus images would not only provide conﬁdence to the nurse that the correct image was being used for the assessment, it also provided nurses with information they could use to convince patients to seek treatment. If a patient needed urgent treatment, but wasn’t experiencing any symptoms, nurses wanted to be able to show the fundus images directly to the patient, and point out the area of concern. They were excited about a combination of images with the prediction as a way to aid in those conversations.

Cai-2019

Although participants were told that the Assistant predicts Gleason grades, many assumed that accuracy referred to the binary classification of benign versus cancer, suggesting a potential unfamiliarity with the subtleties of assessing the performance of multi-class classification systems. This finding suggests that it may be useful to determine the kinds of performance metrics users are accustomed to seeing, and highlight any differences in the definition of accuracy being used in onboarding so that stakeholders and end-users are better equipped to understand empirical measures of an AI Assistant’s performance. Whi

Catho-2020

Lack of clarity

• GENEVA (GE_07) (F, resident): “We open recommendations that are sixty pages long and it’s complicated to ﬁnd a quick answer”.

• FRANCE (FR_05) (F, resident): “I always have trouble ﬁnding my way around the recommendations”.

Lack of accessibility and availability

• GE_03 (F, senior physician) “We didn’t have time to open the recommendations, we had four diﬃcult patients to see in an hour and a half”

Cresswell-2019

Another perceived factor potentially affecting end-user usability was the degree of local customisation needed to tailor the number of pop-ups to local needs and avoid alert fatigue. Clinicians also mentioned that they were already presented with pop-ups in their own GP systems and that alerts from various sources needed to be aligned and thresholds for alerting set carefully in close consultation with them to ensure usability. Pop-ups come up from all different places, so there’s the notes that practices put on the systems to try and remind you to do something…you’re potentially going to throw another set of pop-ups in there to have your clinical decision support… (Participant 21, male, GP

Dalton-2020

The majority of interviewees liked the design of the report. However, many pointed out that whilst the colours on the report would have grabbed the attention of prescribers, the overall length of the report and the large amount of writing would have been off-putting to readers.

It’s a little bit lengthy maybe because it takes a couple of minutes to read through this and…it’s colourful but it’s rather dull…in continuous text. [Medical Prescriber 12]

Dikomitis-2015

crucial that the threshold levels of all prompts are valid: ‘There are so many things on it, so many things popping up, so many things prompting you. You don’t probably respond to all the prompts, because there’s a box here, a box there, a box everywhere, and you don’t see everything (…) It’s such a busy screen you don’t respond to everything and this doesn’t pop up’.

Flynn-2015

capability of patients/relatives to understand the risk presentations; conveying an artificial level of certainty leading to potential problems with providing individualised information to patients/relatives; an

Benefits in risk communication were emphasised, in particular visual presentation of data:

“feel comfortable saying actually five more people would benefit, there’s no change in risk of death” (Nurse Practitioner 1).

“there’s no significant additional mortality to the natural history’…. that’s very, very difficult information to communicate without that sort of pictogram” (SP 4).

One clinician emphasised the value of graphical risk presentations to support provision of post-decision information to relatives who were not present at the time of treatment: “useful to tell the family and then explain what that treatment was and why it was or wasn’t a clear decision” (SP 6)

Grau-2019

P15, Hospitalist, male: [The order set] is displayed a little differently than the way we’re used to ordering things that are only available in some of the facilities and not all of them. So [to order something,] I have to think about what building am I in. it’s a little hiccup when you’re trying to do this quickly.

Guenter-2019

However, some found that there were more information and data fields available than what they wished to make use of. Some felt that the number of patients they managed with pain was too low to develop ease with using the CDSS. A participant reported, “I remember going in there and clicking around and finding all the different things that were in there and I think if I had spent more time in there and used it, it probably would have been valuable

Hallen-2015

Although physicians perceived mostly beneﬁts of using CPMs in EOL care, they also perceived two important risks. The ﬁrst was the potential for prognostic estimates produced by CPMs to cause emotional distress or panic in patients when estimates were not favourable. Consistent with a large body of evidence, most physicians perceived that such prognostic information would be upsetting to patients and families. Physicians saw disclosure of a poor prognosis in numeric terms as particularly distressing given patients’ tendency to ‘ﬁxate on the number’, as described previously by one geriatrician and another cardiologist:

Cardiologist 1: You can tell somebody [that] you have two chances in three of being dead in 6 months...you know they walk out of the oﬃce wondering what they should do tomorrow

Henshall-2019

The DST was described as ‘attractive’ and ‘readable’ by most participants, with its blue and green features perceived as ‘healthcare colours’. However, some visual design improvements were suggested, including incorporating the word ‘decision-aid’ into the name of the DST, increasing the font size, providing bite-size sections or drop-down lists of content and visually depicting the highest ranked medications and their side effect profiles. Most participants wanted percentages displayed as they were easily understandable, something which was not factored for by the DST. Despite this, psychiatrists felt that it could be a useful base for considering medication options with patients. You’re much more constrained in the antipsychotic you could give each patient … But it could still be used as a launch pad for those types of discussions.

I think everybody understands if you say 100% … Because if it was a 50% chance of weight gain, that’s a fair chance you’re getting it. It’s a toss of a coin. Whereas if it was just 10%, you might not, I suppose. Patient/carer

Jackson-2017

‘I think especially, you’ve got to make your IT really slick...if it’s clunky and difﬁcult, people are going to lose interest quickly’. ‘Patients’ feedback is that the more visually engaging, the less effort it takes from the patient’s perspective’.

Participants raised concerns about the sustainability of the intervention, alluding to the issue of non-adherence to pre-existing eHealth solutions. Methods suggested to improve traction and sustainability included ‘a visually engaging and interactive tool’ that ‘needed to be sticky’ and ‘slick’. A panel member alluded to the fact the ‘we want the patients to talk about the model

Jauk-2021

I like the presentation with the traffic light symbol.” The visualization in the web application (Fig. 1b) sparked much enthusiasm, because it provided a comprehensive view of a patient supporting healthcare quality not connected to delirium prevention. However, during the first month the risk of delirium had been visualised using percentages. This was criticized by the experts, as their interpretation was not clear to them. As a solution, we replaced the percentages by a bar chart visualizing the three risk categories and an arrow indicating the location of a patient on the risk dimension.

“The bar representing the range of delirium risk helps us to identify patients at the border to another risk group.”

Joshi-2020

“if you overwhelm providers with warnings, they'll ignore them. So many alerts are false positives so we're trying to find the correct balance...” (RB) “If you have a lot of false positives you are at risk for alert fatigue and if a positive alert is embedded in a workflow that involves some amount of time and attention, doing too many of those false ones, then people are going to get very frustrated and abandon the effort entirely so I think the biggest challenge is getting to that sweet spot of the sensitivity and specificity of the alert and aligning it with a workflow that is practical and that bedside nurses and providers are actually going to be able to do.”(ML)

Keogh-2019

Clinicians' suggested changes to the iPrevent prototype such as

improving: the scale of graphs; readability for lower literacy levels; the option of a printable page of output for consumers and clinicians; as well as debating the best order for presenting the options to women

The ten year risk compared to the lifetime risk I think is really good. Particularly for women of certain ages. For a 25-year old woman, what's her risk at 35, compared with what's her lifetime risk?.. I sometimes feel that women don't comprehend a lot when all we give them is a lifetime risk.

Liberati-2017

In this position, the main obstacles to the adoption of CDSSs involve the usability and technical issues that have been widely explored within literature and insufficient adaptation of CDSSs to local and contextual needs. Frequently mentioned issues include the lack of integration between the EHR and the CDSS’s interface and the fear of experiencing an excessive number of alerts.

If five alerts pop up every time and you start noticing that most o fthe times four and a half are useless, you start ignoring them. […] It’s a matter of finding the right balance and to improve the content, to make the alerts actually relevant. (Physician, setting C2

Finally, the CDSS may shape strategic and managerial choices (such as the investment in specific research streams), foster a culture of evidence-based policy, and nurture a culture of collaboration between clinicians, hospital management, and IT personnel, which is likely to be essential to guarantee the success of technology based improvement efforts. I would love to start thinking about decision support systems for our strategic and managerial choices too. Evidences for decision-makers. […] We do a lot of work on [evidence-based policy] but I think we could improve in the way we access this knowledge on a day-to-day basis… The work on CDSS is inspiring us to improve. (Hospital manager, setting C1)

Lugtenberg-2015

Examples of perceived barriers related to the format/layout of the CDSS content - Notification method (too intrusive or uninformative)

•“A pop-up means an additional action which might not be convenient at that time. Now, it’s under my own control”.

•“So, you should immediately see whether it concerns a content alert or an alert regarding patient data registration. And also the subject: diabetes, cardiovascular risk management….If you move your mouse over the alert you should be able to see it. That would be worth a whole lot!”.

- Readability of the alert text (too wordy/verbose)

•“I think the phrasing is sometimes very complex. ‘Research has shown that….’ or ‘You could consider…..’. This should be a bit more to the point really!”.

Intensity of alerts (low threshold for triggering) •“So it shouldn't be too much, not like ten alerts per patient right? Then you’ll get a little over-alerted right? Enough is as good as a feast!”.

•“… did you check kidney function, liver function…? At a certain point you’ll get overloaded with information that is actually quite straightforward…. 25 yellow [an alert] out of the last 50 patients....”.

Lytle-2015

In addition, staff felt there was redundancy between the fall plan of care and the patient education topics

McDermott-2014

The prompts were reported as being easy to control when they appeared on the screen. GPs felt that the prompts could easily be controlled as they did not obstruct the computer screen when they appeared during a consultation, and could easily be either viewed or exited with a minimal number of clicks on the screen.

"I didn't find them particularly intrusive or anything like that, that I didn't want to use them, it was easy to ignore them" (P07)

These features included adding further advice for the GP and patient in multimedia format (such as videos), links to additional healthcare services, and information relating to which antibiotic to prescribe.

"It's quite far-fetched but having some kind of recorded message as well,…….or videos detailing about you know…coughs, colds, not needing antibiotics, not needing consultations with the GP as well" (P01)

Miller-2019

A few suggested we should explain why the questions are being asked, talk about confidentiality, and define some of the terms and the contraception charts more clearly. Some

Nova-2020

This information could be reasonably presented in various ways, including with graphs, hyperlinks to more detail, numerically, or with statements and narrative notes

Orchard-2014

Patients generally were impressed with seeing their heartbeat on the iECG. • ‘It’s fascinating … well I did look at the screen as it was moving and it was quite interesting to wonder what it was actually saying’ (Patient 1)

• ‘It’s an impressive little gadget’ (Patient 2)

Page-2019

The other key problem described was alert fatigue (n= 7; 20%)

Patel-2018-additional file

HT aides in communicating CVD risk. Patients find graphs to be helpful and enlightening. The GP uses the heart age projectile graph to communicate CVD risk to patients.

AHW thought the ‘what-if’ graphs would be valuable in giving patients an Indigenous perspective.

AHW: Well, usually when a patient goes in for a consult, they don't usually see anything like this. They might get a bit of paper with their care plan on it that's written in medical jargon and they don't understand it. When you've got something as basic and straightforward as that, it's an easy to read tool for anyone.

AHW: I've been there a couple of times with GP x [lead GP] doing this [‘what if’ graphs]. And he's entered in the data. And then it showed on the projection. What would they do if they changed this, you know, change their smoking and then change that and then change this and watch it come down and see the patient's reaction to that? And even that for me, 'cause I'm a smoker [laughs], yeah, and there was a considerable jump in the risk when he just took away the smoking. So, yeah, it was a bit of an eye-opener. And it's pretty easy to read graph

AHW: It saves you heaps of time because in a blink of an eye you can see what they're due and what they're not, whether or not it could be save a load of time and provide a better care for the patient, making sure we're not missing anything.

Petitgand-2020

Some nurses were convinced that physicians were not using the medical histories because they had difficulty understanding patient information reported by the DSS. This was, in fact, a major barrier reported by several physicians. The physicians interviewed considered that the AI-based system was good at reporting simple complaints (a localized pain, a broken leg, etc.) but very poor at making sense of multicomplaint conditions (pain throughout the body, pain related to severe pre-existing conditions, etc.). This was a major concern, as most patients coming to the ED presented with the latter profile:

The history shows a multitude of symptoms to which the patient responded 'Yes, I have this'. But is it relevant? Is it active? Is it related to the current complaint? Afterwards, you need to disentangle all this. (Physician 4)

Petkus-2020-supplementary file

The level of the alerts within a system need to be targeted to high-risk errors/problems, to minimise the risk of alert fatigue.

Philips-2015

two comments addressed the need for simpler documentation of assessment and recommendations in the medical notes and

Reynolds-2019

“So while it does give you a lot of redundancy and makes sure that you know what is going on with that baby and that medication, it’s almost overkill in that it’s taking so much time..

Roebroek-2020

Graphic representation of the ROM-results made pressing issues in treatment more visible and therefore easier to discuss. Based on the UTAUT model, TREAT’s graphic representation positively affected the predictive factors of effort and performance expectancy.

Some respondents even experienced the recommendations as irritating:

“I feel guidelines are necessary as a foundation but we can also assume they are well-known. To build a system just to beat people over the head with guidelines defeats its purpose. It irritates.” [C10]

In a few cases, patients experienced TREAT as confrontational and it even scared some:

“Sometimes I would notice a negative atmosphere, caused by the results and how they are displayed. That’s because it mostly highlights problems which pop up in red graphs. That scared some patients.” [C12]

Shannon-2021

Some patients wish that the application was more interactive, suggesting that videos or activities would be better at keeping them engaged.

Silveira-2019

clinicians demanded a printed handout to deliver to patients and attach to the patient record at the end of the consultation.

Torenholt-2021

in interviews with patients it became clear, that the email was often perceived as an autoreply indicating that their data had been received, not indicating anything about who or what had been involved in evaluating their data. And as the physician only very rarely informed them about it, most patients had an inaccurate understanding of how their data were evaluated. A

Trinkley-2019

Clinicians overwhelmingly asserted they did not want interruptive or active CDS alerts because they interrupted clinician workflow. The term non-interruptive alerts used by participants was broadly defined as passive alerts that a user chooses to review, whereas interruptive was defined as active or disruptive alerts that interrupted workflow and one is forced to review before completing a task

Multiple clinicians stated the interruptive alerts were not ‘smart enough’ to recognise that a given task was already complete and sometimes fired multiple times after a task was completed. Some notable ‘irrelevant’ examples included a lactation warning for a 60-year-old patient and an outdated alert regarding foreign travel and exposure to the Ebola virus.

Clinicians thought it would be helpful for the CDS to include actual or general costs of medications when making recommendations, which could improve patient adherence and avoid pharmacy requests for less expensive alternatives.

Tsang-2021

A lot of patients just really worry about it and they don’t know what it means… providing them with the written information is an important step.” [P6, pharmacist]

Tsang-2021-Supplementary file

Summarising data quickly – making information more accessible

• “But that’s good because you can look at it quickly without going into [the clinical system] and make a judgment quite quickly.” [GP1, doctor]

• “Looking at that visually, you can get an idea very quickly as to what’s worrying and what’s not worrying.” [GP2, doctor]

• “It’s just quite visual, isn’t it? Breaks up because I’m looking at the GP records which has so much information.” [A4, administrator]

• “It just brings up the information that I need, especially when it’s connected to (the Hospital) and it’s such a faff going on the actual records.” [P6, pharmacist]

Van de velde-2018

Those that implement CDS should carefully reflect over the amount of CDS, because too much information can overload both patients and GPs:

When the information becomes too much, then you lose focus. [GP, Norway] The CDS should not overload the patient, too much information will lead to forgetting parts of it. [GP, Belgium]

Several GPs emphasized the important role of a visual display that includes illustrations.

Watson-2020

Another factor that was identified as affecting clinical utility is the challenge of configuring alerts; striking the right balance between over-triggering and under-alerting when action is needed has proven challenging. This can have a direct impact on the clinical utility of a model. Nursing alarm fatigue, particularly with some of the most critically-ill patients, is a well-characterized phenomenon.20 One interviewee explained, “one of the biggest challenges in implementation is figuring out what signals you should send and who to send them to, when and how.”

Wickstrom-2020

Another engaging factor was the opportunity to make patients more involved and engaged in their own treatment because of the possibility of visually following the healing process in the DDSS

Yang-2019

Mid-level clinicians viewed the slides as a potentially important vehicle for communicating their opinions to physicians.

A prognostic DST that indicates post-surgery quality of life could potentially amplify their voices. There is not a way to present (my reasoning) formally. It’s just me saying: ‘This, this and this’. [...] I think it’s good to have something visual for anybody to see. It’s like, OK. LOOK. Let’s slow down a bit here. (Nurse practitioner)
