# Supplementary material for: Stakeholder Perspectives of Clinical Artificial Intelligence Implementation: Systematic Review of Qualitative Evidence
Source: J Med Internet Res. 2023 Jan 10;25:e39742. doi: 10.2196/39742 (PMC9875023; doi:10.2196/39742)
Supplement: Multimedia Appendix 3 [file jmir_v25i1e39742_app3.zip › 2. Technology/2c. Knowledge generated by it/2c.2 Target a clinical need.docx]

**Name:** 2c.2 Target a clinical need

Alagiakrishnan-2016

Some drugs are metabolized through the liver, some through the kidneys. It would be useful to identify in the medication CDS which drugs and dosages are influenced as a function of the GFR value.

Others mentioned being familiar with most of the message content, already committed to medication management, and requiring minimal adjustments to their practices. This group of clinicians reported that it took a “few minutes” when adjusting medication orders to decide the course of action and provide justification when managing alerts.

Benda-2020

Challenges that could inhibit the organization’s use of a predictive algorithm included the possibility that the at-risk patients identified would be clinically obvious. I know the medical conditions of my patients and how severe they are. So, having that in my face probably doesn’t really add that much. – EU02 [Challenge]

Chrimes-2014

I think it [Flowsheet] is straightforward, which it is good. Also, it is broken up by colors, which is also good. I am not sure what to do about the ‘meals at home per day’ and I don’t know how that is going to help with my patient education

Connell-2019

However, some respondents in the nephrology team did not see the point of being alerted to low-risk patients:

...you get a lot of AKI stage 1s. They build up. Looking through those and dismissing them each time is time consuming. The AKI stage 2 and 3 [alerts] are more helpful for me to look at, so I tend to just look at those and dismiss the stage 1s. [Respondent 18: Nephrology team]

Dalton-2020

I had recommendations that were irrelevant and even, even dangerous. [Medical Prescriber 4]

de Watteville-2021

Difference with the current local protocol: range too low.”

“Readjust the glycemic targets upwards”

Haan-2019

Patients also say that it is unclear to them how Al would affect the procedure of scanning and receiving results. Patients seem to prefer an “all-inclusive” use of AI when evaluating scans. They report that they would like to receive results not only of findings based on the questions of the referring physician (ie, the primary aims of the scans) but also of incidental or unrequested findings that can be extracted from the scan.

Henshall-2019

However, a lack of familiarity with some of the DST’s recommended medications, and the fact that some were not licensed for use, provided a deterrent. Bar Loxapine, which I’ve seen once, I’ve never seen any of the others … That would not be helpful for me. Psychiatrist 2

Horsfall-2021

In relation to patient management, the responders were concerned about AI’s nonspeciﬁc approach

Joshi-2020

Well my major, major bone of contention with the algorithm is that the endpoint that they are predicting is not clinical. I understand that it's easier for them…for me the buyer and the clinician, for this to work I need a clinical endpoint.

Kendell-2020

Participants also identified potential limitations of using an EMR-based algorithm in that such tools would not be applicable to patients without a primary care provider, all deaths cannot be predicted, and it would add to physicians’ workloads.

“… I just can’t see it ever coming to be a real thing that’s ever going to be used. I don't think doctors have time for it.” [Nova Scotia, Participant #5

Keogh-2019

For clinicians, the main perceived beneﬁt was iPrevent's provision of accurate and reliable risk estimates for both ten year and lifetime risk, and standardized risk management advice for women (see Table 5, quote 1&2)

I think the beneﬁt is you're empowering yourself with information, you're possibly changing what you can change

Liberati-2015

[In the first case, clinicians propose a dualism between the “artisan component” of the trade of the doctor and the scientific evidence, hypothesizing a polarization and contrast between the two approaches. The evidence is not a guide in practice: the experiences of colleagues or the "eminence” of the sector are considered a source of more relevant information and support. The SSDCs, therefore, they are greeted with detachment and indifference: a little useful tool in the concreteness of the clinical activity. It is important to note as none of our respondents identify themselves directly with these opinions, attributing them to colleagues. This I'm suggests a willingness to mark a certain one personal distance from the positions expressed.]

Morgenstern-2021

You’ll probably be accused of nanny state-type intervention, but, you know, every time you see someone eating a cheeseburger and French fries on their [social media platform] you say, ‘Hey, [ …] how about some vegetables?’ [Participant ID # 4].

the question then becomes is the extra information that comes from loosening [the model] up actually valuable and useful? That’s what we need to still figure out. We don’t have a good handle on that yet. But I think we will. […] And then we can refine our efforts at focusing on those specific places instead of just saying, oh we need to just […] neural network everything.

Porter-2018

Many paramedics felt that the software was too simple or basic to assist them with their decision-making, stating that their own clinical judgement skills and experience levels placed them in a better position than the CCDS to decide the most appropriate onward care. I just felt that, I don’t know, some of the questions were… I don’t know, just not as advanced or a bit below paramedic level on occasions. (End S1 05)

Rapoport-2020

… It’s not like I used the tool and said, ‘Whoa. I didn’t think of that. I need to change my mind in either direction’. So I don’t really think that adding more information would help, because my mind is kind of made up. [MD07-SP]

Reynolds-2019

“It’s always in default to the one that you don’t want 99% of the time on that milligrams per kilo per unit. It’d be nice if it didn’t default to that one since that’s a minority of the ones that are chosen...”

Roebroek-2020

TREAT was not beneficial in reminding me of new things we could try for a specific problem. It’s not really a lack of knowledge I experience when drafting a treatment plan or when starting a new treatment.” [C12]

In addition, more than half of the clinicians indicated that some of the recommended interventions were not part of the available treatment resources within their team. In some teams, nearly all of the recommended interventions were unavailable.

Sun-2019

Third, there is the issue of different regulations on drugs in China, compared to other countries. As pointed out by [3IT04], the IBM Watson system might design a treatment plan that includes a drug that is legal in the U.S., but not in China.

Van de velde-2018

Knowledge about the reason for encounter could help GPs to prepare for the consultation and could prevent the CDS from generating information that is irrelevant for the reason for the encounter:

It would be interesting if the GP could indicate the reason for the encounter and that CDS is triggered accordingly. [GP, Norway]

A GP commented that CDS should cover a minimum number of potential patient problems:

CDS should at least cover 100 to 200 diagnoses before it becomes interesting to use. [GP, Norway]

Velez-2014

As articulated by one: “We just need the data for the reports; this other stuff we do not need. I would not look at that.”

Yang-2019

Clinicians also had wildly different readings into the same DST prognostics. We presented the same two synthetic cases with the same implant survival predictions to all participants. Interestingly, they generated wildly different reactions and interpretations of the cases. Some viewed the survival estimate as implying that an implant would not work. “Gee... VAD is futile here.” Others viewed the DST output as implying the patient should be immediately implanted, before things got worse. ‘‘We still have a chance.” Few clinicians believed that all VAD implant candidates would have a similar prognosis as the synthetic case we presented: “This chart is meaningless. Every VAD candidate’s projection would look like this.

Clinicians frequently asked us to clarify whether DST prognostics are predictions that carry agency and subjectivity, or if predictions are facts rooted in historic data. We sensed they wanted to limit discussions to facts, including how heart failure has played out for the patient they were treating and the statistics from previous, similar cases. We observed resistance from some clinicians toward the idea of showing predictions. Our collaborating physicians, who created the synthetic cases and helped us select contents for the slides suggested that the DST output should be “one statistical representation of 100 patients who are similar to him” rather than a prediction for this individual patient. I think if you continue to call it “VAD projections” 65%, people are going to poke holes at it. They are gonna try to prove you wrong. This [DST projection] is just what the historical outcomes were. But this guy is different, this guy has his own things that make him special. (Collaborating cardiologist, hospital A)

Clinicians were confused by this notion of “now” because it was extremely unlikely that they would implant a patient on the same day as the decision meeting. Is “that 21 days from today? If we are gonna lose the patient in 21 days [21 days following after implant], can we just wait?
