# Supplementary material for: Stakeholder Perspectives of Clinical Artificial Intelligence Implementation: Systematic Review of Qualitative Evidence
Source: J Med Internet Res. 2023 Jan 10;25:e39742. doi: 10.2196/39742 (PMC9875023; doi:10.2196/39742)
Supplement: Multimedia Appendix 3 [file jmir_v25i1e39742_app3.zip › 2. Technology/2c. Knowledge generated by it/2c.3 Recommend clear action.docx]

**Name:** 2c.3 Recommend clear action

<\Alagiakrishnan-2016

They lamented lack of specific details about exactly how best to wean or discontinue a drug, monitor for unwanted effects, or choose an acceptable alternative in the face of clinical need.

whereas physicians expressed concern about overly specific messages at the start of the study, they most commonly requested enhanced guidance toward the end of the study

<\Ash-2020

When we asked users what CDS they most appreciated, they said it was reminders and information resources. They were willing to take the time to seek out these resources if they were in the EHR, easily found, up to date, and evidence based. They wanted CDS that would be well worth their time. For example, many interviewees noted that the CDS that would help to generate a letter about returning to work after a low back pain diagnosis might offer them extremely useful guidance, so they reacted positively to it

But you know, it’s one thing to highlight their problems and what the contributions are but what does the patient do with it? And what does the physician do with it? So the decision support in that case should link to things like the resources that are available for the patients to access some kind of assistance with how the patient manages those situations. You know, you don’t want to have a situation where they run in with this thing and they get ﬁred. Or you know, you want to set up dynamics that are counterproductive and some patients will do that.

<\Benda-2020

Adoption would require easily interpretable information, linked to clear actions. The majority of participants agreed that a categorical score would better facilitate interpretability and actionability than a numeric score. For the nurses to be able to kind of plan their care, I think it does help for it to be categorical. It makes it easier than trying to get a percentage or a number and then, having to reference something else. – EU09 [Facilitator] What would be helpful is not just the identification of a patient but suggestions on what would be the most appropriate resources that the patient would need. – OPS20 [Facilitator

It was also seen as important to be able to begin the process of an intervention within the workflow of viewing the algorithm’s output. A few participants remarked that they would be open to an action occurring automatically based on the output of the algorithm. Recommendations may be helpful as well as an easy, automatic referral to case management would be great. – OPS20 [Facilitator]

<\Bourla-2018

Impact of predicting a potentially incurable disease

<\Chrimes-2014

Moreover, some providers stated that the patient instructions or behavior contact resembled too closely to a medical prescription. A less rigid form of monitoring was thought to be needed in setting goals of the patient’s behavioral change.

<\Fan-2021

The analysis of textual feedback revealed that users desired to receive more personalized and actionable information, including medical information related to their health concerns, where to seek medical help, what to do next, and detailed explanations about the suggested diagnoses.

<\Flynn-2015

Clinicians reported benefits in clinical decision making: e.g. “clear presentation of the risks and

benefits…..able to look at the charts and say yes we should do this or … confirming your no’ (Stroke Physician [SP] 2), especially for patients at extremes of the licensing criteria; e.g. the lower end of the NIHSS: “confirmation that this low level of NIHSS had benefit” (SP 6

<\Johansson-Pajala-2017

Although time was saved, some RNs expressed that the quality of the responses differed from previous medication reviews, in that the CDSS presented a quality report and no speciﬁc recommendations

<\Johansson-Pajala-2019

You feel that you want a purpose to go with it. Not that we gather a lot of information, which we give to the physicians, and nothing happens, I want a response…” (N2)

<\Jones-2017

Participants also identified the usefulness of UTI Decide:

“This is data collecting. You’re not saying “per your assessment, make these recommendations”; it’s basically saying “this is the evidence based recommendations”. They can start those. They can collect data. They can start those. [sic] And getting them to start that as quickly as possible—that may be the piece most beneficial in the long run.” (Usability Participant 1)

and: “Yes. It helps you. Because it’s what information you need before you call the doctor.”(Usability Participant 4)

<\Joshi-2020

“I think a real challenge has been figuring out who…get(s) the alert and how they were going to get it and what we were going to do in cases where a patient triggered an alert and one hadn’t yet assigned themselves to the patient so I think some of those logistical challenges of making sure the alert has gotten the attention of the provider but didn’t disrupt the provider. That at least for me was what we found the most challenging.” (RB)

<\Keogh-2019

Others expressed that risk itself is not compelling, but if, on the basis of risk, they could reduce their chance of developing BC, it would be worthwhile knowing (see Table 4, quote 3). Finally, some said that, “information is power”. These consumers regarded all information about their health as valuable. They assumed that knowledge of personal BC risk would be useful, and could put women in charge of their health (see Table 4, quote 4).

think people are more interested in their treatment options and prevention options than their actual risk

<\Melo-2020

It was evident that professionals need to be able to incorporate technological innovations and use the available information to make decisions that lead to effective practices,

<\Nicks-2016

NHVs also emphasized the importance of providing safety products to clients. The six NHVs who provided study-related safety products to clients reported that this component of the program allowed them to not only identify high-priority injury risks but also provide solutions for these high-risk families. The remaining NHVs interviewed expressed frustration

“So it is hard when you provide the education but yet they don’t have the finances or the resources to make the home safe once you provide the education so . . . this is the frustrating part.” (SNS-I program nurse)

<\Nova-2020

You need to make it succinct and if you're able to link it with an action or suggestion: about what to do.

<\Orchard-2019

Approximately 16–20% of iECG readings were abnormal (either unclassified or possible AF) and required at least some follow-up. Therefore, nursing teams performing screening need a clear and efficient protocol for dealing with abnormal screening results.

“If we had worked it out with our GPs beforehand, and had a clear protocol of how to move forward, it would have saved us a bit of to-ing and fro-ing between the GPs and myself.” (Nurse, Practice I).

This is particularly important in the context of a hierarchical general practice system, where nurses may not be empowered to take next steps for follow up without GP input.

“A nurse can’t tell the patient anything. The doctor can at least provide some insight into what the result means.” (Practice Manager, Practice I).

<\Patel-2018-additional file

HIO: …for example only 30% of the high risk patients are being prescribed with triple therapy and they go, whoa, you know. So they really like that, and we can then actually provide them with the names of people, you know, and then sort of next time they present to the service they can have a quick look at their, you know, medication management.

<\Philips-2015

Simpler documentation in case notes with clear instructions on: 1. Dose, 2. Duration, 3 and Dates for monitoring concentrations’

<\Roebroek-2020

Most clinicians have a recovery-oriented view on patient care, which sometimes contradicted the alarming nature of TREAT as this respondent explained:

“Our intention in our patient contact is to try to focus on recovery and strengths. However, TREAT draws the attention mostly to the negative points.” [C13]

<\Trinkley-2019

Participants wanted the CDS to present pertinent patient-specific information to inform their decision, such as pertinent laboratory results, vital signs, and drug allergy information.

Clinicians expressed interest in CDS that would populate text in their clinical documentation or patient instructions to make documentation easier. Clinical documentation and the patient instruction sections of the EHR encounter would automatically be populated to reflect the plan, counselling points and necessary follow-up including labs if they accepted a CDS recommendation to order a new medication for a patient.

<\Urquhart-2018

To facilitate care post-identification, participants emphasised the need for provider- and patient and family-based supports. For providers, these supports included tools such as checklists, decision support tools, and toolkits to engage in advance care planning and delivering a palliative approach to care:

‘So now we have a tool, so now we have to do something with it, we have to put those things in place. Would be possible to have an algorithm that leads you through what needs to be done? … Because otherwise you are overwhelmed with stuff.’

<\Van de velde-2018

CDS should recommend specific action. Many participants requested more information about which type of exercise works best:

I got the instruction to bike 30 minutes/day. It has never been clear why I had to do exactly this training. Is this type of training more beneficial than others? [Patient, Norway]

GPs commented that CDS should be relevant for the patient’s problem and that irrelevant CDS content can be disturbing: CDS can diverge the focus of the consultation to the topics suggested by the CDS instead of the problem raised by the patient. [GP, Norway]

<\Watson-2020

One of the pitfalls some of the groups experienced was to develop models that lacked clear actionability. For example, one interviewee explained of a model:

They validated that higher scores [are] a high risk of readmission, but what we don’t know for sure is whether you can do something about it. Someone who has a gazillion illnesses and is super old, they will always have a high risk. Whether it’s a modifiable risk is the true test of how useful it is...but what we don’t know for sure is whether you can do something about it.

<\Yang-2019

Many clinicians’ questions, as well as their discussion around the DSTs, revealed a tension between what they saw as the DST’s static view of patient conditions and the clinicians’ desire and ability to also focus on future actions and interventions. They wanted to know which modifiable factors most influenced the DST predictions. They wanted to be able to offer treatments that they could improve these factors, thus increasing the likelihood of a positive surgical outcome at some time in the future.

These predictions are (what will happen) despite our best efforts, right? (VAD manager, C8)

Having an understanding of what’s driving the risk [features that most influence the prediction] is very important for us to understand what is modifiable at that patient. [...] Is it age or something we cannot change? Otherwise there is a lot of potential here. (Hospital C decision meeting)
