# Supplementary material for: Stakeholder Perspectives of Clinical Artificial Intelligence Implementation: Systematic Review of Qualitative Evidence
Source: J Med Internet Res. 2023 Jan 10;25:e39742. doi: 10.2196/39742 (PMC9875023; doi:10.2196/39742)
Supplement: Multimedia Appendix 3 [file jmir_v25i1e39742_app3.zip › 2. Technology/2d. Supply model/2d.1 Equipment and network requirements.docx]

**Name:** 2d.1 Equipment and network requirements

Abdi-2021

“Technology is available and not costly anymore, more easily customised and therefore to be expected to be useful within the next ten years” AI-enabled apps, P7

limited access to smartphones and internet were seen as potential barriers of older people’s adoption of IoT enabled homes, AI-enabled apps and voice activated devices, despite the potential beneﬁts demonstrated by these technologies in various care domains

Abidi-2018

Participants felt that DWISE has the potential to improve diabetes self-management, especially given that mobile phones are ubiquitous and self-management plans formulated through DWISE can easily be integrated into the patients’ lives. Participants indicated that DWISE has the potential to improve diabetes-related monitoring:

Phone is ubiquitous, so more opportunities. I love apps for recording and monitoring...this can help me monitor my sugar.

Andrews-2017

Participants also commented that the requirement for either mobile reception or wifi should be considered.

P6: how does it report back? So does it, is it through that mobile phone technology, or does it rely on wifi, all of these kinds of things?

Chirambo-2019

We discovered that mHealth users who stayed near trading centers in electrified houses had no problems with charging their phones. However, those staying in the remote areas had problems with charging their mHealth devices, despite some of them being given small solar panels for energy.

“I have to walk a long distance to find a barber shop where I can charge my phone.” PSA-7 “When I want to charge my phone, I have to pay K100.00, and if I don’t have the K100.00, it means on that day I can’t charge my phone.” PSA13

The study also discovered that when the phone develops a fault it becomes difficult to repair because most local phone repairers do not have the accessories to deal with smartphone problems.

“You know the village phone repairers do not have most phone accessories. So when the phone develops a fault, it becomes difficult for them to repair. We have to go to the district township to find better phone repairers.” PSA-13

“I am worried that if my phone glass breaks, it will be difficult for me to repair it because I was told by the people who repair phones that the glasses for these phones are difficult to find locally.” PSA-7

Clyne-2016

The reviews were a positive experience for two main reasons. First, the intervention website and treatment algorithms were considered simple and easy to engage with by all GPs: “It was very straightforward, it worked well I thought, em it was clear and you know, from our point of view, actually when you actually got down to it, the patient, the actual process of going through the patient was quite quick.” (GP24, intervention practice).

Henshall-2019

All participants felt that the tool was simple and straightforward to use, whether on a mobile or a laptop, and that it was easy to navigate.

I’m all for simplicity and I found it … I’m a technophobe. I’ve got my smartphone. It’s not set up yet. But I used that smartphone with no problem at all. Patient/carer 4

The DST was seen as a means of stimulating, rather than replacing, discussion, avoiding paternalism by promoting shared decision-making between doctors and patients. However, a couple of psychiatrists felt the DST would be more accessible if it was downloadable, rather than requiring Wi-Fi access. Most participants felt it would be most useful as an adjunct to other information sources, such as the Internet, leaflets and face-to-face discussions.

It’s part of an armoury of enabling patients to be involved in their treatment decisions. Psychiatrist 4

One patient/carer suggested that a supplementary app website

would be helpful, with patients being directed to it after their introduction to the DST during their consultation with their doctor.

If you’ve got it on a website … Having done it in the office with you … They can go away if they haven’t made the decision and play with it some more, which might be useful. Patient/carer

Jacobs-2014

“Do not have a fully implemented EMR, still using a fairly manual system.”

Jones-2017

Participants found the application easy to use:

“…what I like about this is that it’s accessible. It’s more accessible than you having to write down your assessment and then go to the computer. If I had the phone on me, while I was assessing, I could just jot it down there. I think that’s nice and user-friendly, in that sense.” (Usability Participant 2)

Joshi-2020

We generally like to do things as much as possible within our EHR without involving third-party vendors and so that is what we looked to do.” (RB)

And because we have Epic, because there was no additional cost to implement their method, this is in all honesty, it was determined that that could be where we could start.” (ML)

Lawton-2014

the remainder (n = 4) highlighted practical and logistical reasons. This included a couple of MDI users who discussed how they had preferred their old metres, which did not have built-in advisors, because they were smaller, lighter and hence easier to transport: ‘‘I know this is quite silly but it’s too big for me, I feel I already carry so much in my bag’’ (M22.1)

Melo-2020

They emphasized that the implementation of 4.0 technologies will make it easier to identify risk situations earlier, leading to faster interventions, as patients will be connected by IoT, with one or more devices/wearables, sharing their biodata. Therefore, more important than data collection is the possibility of processing them in real time and screening them by algorithms. This connection to patients by IoT will enable early diagnosis, health/disease monitoring, precision medicine, and medical history intelligence.

Miller-2019

streamlined electronic health systems.

Mozaffar-2016

Our findings show that implementations could be delayed as CPOE/CDS became dependent on the implementation of other applications. Such decisions were taken particularly with non-integrated systems. Here, decisions to delay the go-live were made by the adopter organization to achieve a broader goal (e.g., smoother flow of information between different systems).

The biggest delay and the most significant one was obviously […] dependency on the delivery of the EDS [electronic discharge summary] software […]. Ifyou look at this, just to illustrate this was an updated version of the plan but the software is going to be delivered here and we’ve got future stake workshops scheduled at the time the software is delivered. That can’t happen, we have to get the software, create the future stake process maps and then hold the workshops so this date is likely to go back and this date is therefore likely to move as well. (Site E, Senior Project Manager)

Nicks-2016

Many NHVs also reported that it was easy to use the program on their laptops (Table 3, Item D) and appreciated having all injury prevention materials organized and together in one tool (Table 3, Item E).

Patel-2018-additional file

“Let’s do this, remember the password, do this, it’s the internet’s slow. Bugger it. I’m going to go have lunch”

disadvantages ..it did take a little while to load if you didn’t have it running already, and just a few software glitches, not with your programme but mainly with Medical Director, because most of the time the questions about this come up during a health assessment, Aboriginal and Islander health assessment which is a horrible programme in Medical Director that runs alone and you can’t move from it to any other programme. So you’ve almost got to hand write your little notes and then come back to at the end, and you always run out of time. So that’s just an unfortunate part of Medical Director, but no, that’s about all.

Petkus-2020-supplementary file

all CDSS in the clinical environment is burdensome unless the hardware to run it is responsive and available.”

“AI is many things and the regulation and testing of it is different in different scenarios. The patient held instrument with AI can be very helpful and difficult to regulate as it is downloadable on the web and does not have to pass a regulatory barrier of purchase by an institution (Hospital Trust or NHS). It is this group of AI that is likely to be the most disruptive and possibly most useful.

Porter-2018

At the very beginning when we – when we had the – the tablets, I think… that wherever we had the screens and we didn’t have the keyboards, or we had the keyboards and not the screens, and we didn’t have new printing paper. And when I said – about that, oh, all you get is a shrug of the shoulders sort of thing, like, you know…. It took months and months and months to get the right paper. (End S1 04)

Reynolds-2019

Finally, while participants still saw the potential utility of a portable DST like pac2, they envisioned a system that addresses some of the aforementioned usability issues: “.. . [a decision support tool] that could be easily integrated into the programs itself installed into, say, a tablet.. . or again our personal devices....” In other words, nurses wanted a tool that integrates with the other systems that they use and on a device with which they are already familiar.

Shannon-2021

Challenges with internet speed and access are another cause of frustration. While the clinics were provided with Wi-Fi access as part of the study, at times the Wi-Fi can be unreliable, which leaves providers without access to their tablets.

Van de velde-2018

Patients suggested multiple channels to deliver patient-directed CDS:

CDS should appear in all the communication channels that a patient uses. For example a smartphone, e-mailbox, etc. [Patient, Finland] Patients do not have access to CDS that is presented in the EMR. Can the electronic patient record be an instrument to provide CDS to patients? [Patient, Norway]

Vedanthan-2015

A frequently cited barrier to implementation was identified as “Server Problems”. Throughout the development and implementation of the project, several issues involving the AMRS server negatively impacted DESIRE testing and rollout

Wang-2018-Tables

I think the mobility thing is important to have. Like to have it (CARATV2.0) with the patient. Like explaining to the patient about the things. And have it portable not on a desk top. I guess there is access issues around if it is something like internet based. (N01)

Wickstrom-2020

Technical difficulties were mentioned, such as the lack of a spellchecker, sufficient space on the smartphones, immediate access to the patients’ medical records, and compatibility with Android technology.
