# Supplementary material for: Stakeholder Perspectives of Clinical Artificial Intelligence Implementation: Systematic Review of Qualitative Evidence
Source: J Med Internet Res. 2023 Jan 10;25:e39742. doi: 10.2196/39742 (PMC9875023; doi:10.2196/39742)
Supplement: Multimedia Appendix 3 [file jmir_v25i1e39742_app3.zip › 2. Technology/2d. Supply model/2d.2 Working across multiple health data systems.docx]

**Name:** 2d.2 Working across multiple health data systems

Ash-2015

Clinical sites rarely share CDS content they have developed. A hospital CDS analyst said: “Some things are hard to share. So you just get like a bunch of screen prints...somebody has to actually go and program that or configure that, and it may be a six month effort.” Some vendors enable sharing by providing web sites and discussion mechanisms for this purpose

Both clinical site and content vendor groups are frustrated that more progress has not been made by EHR vendors regarding interoperability and use of standard protocols

With respect to CDS, the content vendors would like it to be easier for their products to be embedded within different commercial EHRs: “It can be sometimes a little frustrating when their format is just different than ours.”

The product that content vendors produce must be able to be integrated into multiple EHRs in order to be commercially successful. Integrating content has implications for product design and underscores the need for effective relationships with EHR vendors.

Benda-2020

They did note that local customization might be necessary given local differences in the structure of clinical content. For example, similar information may be stored in different fields or formats across systems. If you’re talking about the universe of [EHR vendor] customers, obviously there are differing technical aptitudes and implementation agility. – INF01 [Challenge]

Participants also wanted the score to be easily accessible within their current workflow in the EHR.

Make sure that’s in their workflow. If you expect someone to go to a third-party system or to a website, you’ve lost. – INF02 [Facilitator]

Cresswell-2019

However, it did at the time of our work not integrate with Egton Medical Information Systems and Vision, primary care EHR systems procured centrally in Scotland. Participants expressed hope that this integration would be possible in the future. In the interim, developers had created interfaces that allowed interfacing with the Platform but did not provide full integration. I think it will have to [integrate], but how we get to that point, I'm not sure. So we do have a contractual requirement for [developers] to deliver, and interact with our clinical decision support systems. So any supplier would, notionally, at least, have to be able to do that. (Participant 14, male, Clinical Lead) What we do know is we are using a demonstrator environment with the look and feel exactly like it will look in reality, but for the real integration we will most likely use a third-party developer in the first phase. (Participant 9, male, Developer)

System integration was also perceived to be crucial from a usability viewpoint and clinical interviewees expressed strong concerns that the Platform may not effectively integrate with other primary and secondary care systems (as well as their existing in-built DSS). This lack of integration, it was feared, may lead to slowing down of existing systems and parallel data entry (leading to duplication of data entry and data inconsistency). We feel like we’ve got lots of systems that work separately. Now, they don’t talk to each other…so you end up having to put data into more than one system…so if they could talk to each other a bit better I think that would help. (Participant 25, male, Consultant

Dikomitis-2015

In addition to accurate training, efﬁcient use of the eRATs depends very much on the accuracy and the level of Read coding (i.e., a standard clinical terminology system). This, in turn, depends on the individual GP’s recording behaviour:

‘You have to have a consistent way of recording Read codes. As far as I know there is no agreement on how to record Read codes and the symptoms are not that much recorded, but then there is great variance in the people who record it’.

Jacobs-2014

“Multiple computer systems within our own organization that have tremendous difficulty interfacing with each other.

Johansson-Pajala-2019

“If I use a system and I want to enter another one, then I need to log out from that system and log into the next… it's really tiresome” (N2)

Joshi-2020

Hospitals for the most part preferred to use tools supplied by their EMR vendor or home

grown tools that are integrated into their EMR over third party tools. Several who experimented with a third party tool ultimately chose not to use it due to difficulties with external contracting logistics, distrust of vendors, lack of customizability, and difficulty with integration into existing workflows. One participant described the choice as follows, “Either you purchase a program through your EHR vendor, or you try to build it yourself, or you purchase a third-party solution and hope that they are not lying to you. Or you know putting lipstick on the pig. Or you know just making it sound better, oh we're going to do this for you and it's going to be amazing right.”

“But I don’t think any of these, so you can have the technology, but you still have to build the workflow around the technology. I don’t think any of them are totally plug and play. That play is going to depend on a lot of other factors.”(ML)

Klarenbeek-2021

data relevant for MDTMs is not consistently stored at speciﬁed locations in the electronic medical record (EMR) but at various locations. This makes it difﬁcult to ﬁnd and retrieve all relevant patient information. Reasons are, for example, an inefﬁcient user interface but also unstructured clinical reporting by clinicians, and storage of patient data in scanned documents or external systems. This was expected to form a barrier for accurate functioning of the CCDSS, if the CCDSS is not able to recognize and locate all data essential for decision-making.

Lee-2015

think we’re getting a little more quality clinical information, but I would say we still have to go into the electronic medical record very often to look up clinician notes, even with the amount of information that we’re provided. Most radiologists expressed the need for better integration of the electronic medical record with CDS and for better alignment of the added information from CPOE with the information that clinicians need for appropriate ordering of imaging examinations and radiologists need for protocols

Liberati-2015

[ergonomic problems (the system but it must be well integrated into the medical record informatics allowing an easy interaction); the lack of integration of information systems between them (electronic folder and SSDC must be compatible and not work through media different]

Lugtenberg-2015

Lack of integration with other systems

“The alert screen should directly be linked to follow-up actions that need to be done! So, if you are to prescribe a statin, it should go directly to that screen. If you have to register blood pressure, you should be able to register it right there”.

Miller-2019

Frequently cited potential barriers were: limited time/ED flow constraints, challenges in maintaining privacy, and technological concerns (e.g., computer theft, interfacing with electronic health record).

Morgenstern-2021

Increased standardization was suggested to improve

the usability of health data and the potential for linkage among various datasets (see Additional file 6).

Morgenstern-2021-supplementary file 6

So, number one is to work on standardizing the data right, um, going forward. So, you know, really thinking about a common data model. Again, this comes down to good data governance practices and interoperability practices. [Participant ID # 10]

Mozaffar-2016

The need to develop interfaces between various applications was also an important cause of delays. We observed that, although the systems could go-live, their implementations were delayed to achieve the longer term advantages of transferring data from CPOE/CDS to other existing systems.

In theory, and I gather from [name of project team member] certainly last week it still wasn’t fully in place within the [system name – standalone] system but in theory they’ll be able to just pull it out from the [system name] system onto the discharge summary but she was saying two weeks ago that it wasn’t, that particular bit wasn’t in place yet but they were working on it. We’ve got a meeting with them tomorrow so I might find out more then, but in theory it should make [hospital medical records] writing easier because they can just pull it straight off the [CPOE/CDS] system but that’s not ready to roll yet, she’s concerned that could be one of the delays that would stop us from starting next week. (Site C, Senior Sister)

Such interfaces, if designed well, could also be beneficial for the users of the system. However, there was also the possibility of several designs being tested to achieve the best result, which in turn could delay the implementation even further.

Although such challenges were mainly observed in standalone systems, integrated systems presented similar issues, particularly when two-way interfacing between hospitals and other health and care organizations

Furthermore, the challenges became even more when customization of integrated systems was needed. This was because of the higher complexity of integrated systems resulting from tightly coupled dependencies that existed between different modules and their underlying databases

Nova-2020

I think that the computers system, especially having it (CDSS results in a patient’s chart, if there's a way to flag it, or just a way. like you were saying. to incorporate it into the EMR, think that’s much more useful Um, and if there's a standardized way of doing it then that would be even better. (Dr D)

Patel-2018-additional file

HIO: I think it’s a very good tool to have added to our kit. The fact that it’s electronic, the fact that it integrates with the medical record, I certainly see its value and purpose. I think the doctors certainly see its value and it’s, you know, sort of streamlined their consultations from the what do I need to do point of view, and then extended their consults on the how to engage with a patient

Petitgand-2020

Moreover, the lack of interoperability between the DSS and the AHC clinical

information systems (Electronic Patient Record and Emergency Information System) meant that medical histories had to be printed

Petkus-2020-supplementary file

“We worry about (a) the lack of uniformity of systems, so that doctors working in one Trust need to be trained when they move to another Trust (i.e. the standards do not specify the user interface);

Rapoport-2020

Other aspects of the tool that participants found problematic were the need to log in to a website each time they used the tool, and the lack of interface with their electronic medical records system.

Santillo-2019

They also reported concerns about incorporating the Decision Aid categories into practice, including how long it would take to change the drug chart and how to do so in e-prescribing systems.

Wang-2018

However, both GPs and hospital-based health professionals were worried that some of the practice computer software might not interact well with CARATV2.0. They were also concerned that some of the information required by CARATV2.0 might not be available in the electronic health system, which would mean that such information would require manual entering

Wang-2018-Tables

The weakness will be if this tool is not in cooperated in the practice software, so it must be integrated. Because it would be very difficult for a doctor to go somewhere else or another website. It is going be there as you are prescribing and also in a hospital situation it has to be accessible. So you know in the patient’s file it is going to be part of that medical record and accessible to people. (G06

Watson-2020

However, regardless of the method used, integration of these models into the EHR poses a unique challenge described in this interview:

The issue right now is in the more general context of how do you implement decision support in a commercial EHR? And, how do you do it in a standardized way that a third party can build one tool and deploy it across different EHRs? And right now, none of the vendors have really sophisticated ways of embedding decision support both in triggering that decision support and then running decision support and then taking that feedback and bring[ing] it back into the record to do other things. That process is extremely naïve right now.
