# Supplementary material for: Stakeholder Perspectives of Clinical Artificial Intelligence Implementation: Systematic Review of Qualitative Evidence
Source: J Med Internet Res. 2023 Jan 10;25:e39742. doi: 10.2196/39742 (PMC9875023; doi:10.2196/39742)
Supplement: Multimedia Appendix 3 [file jmir_v25i1e39742_app3.zip › 2. Technology/2d. Supply model/2d.3 Quality of the health data and guidelines used.docx]

**Name:** 2d.3 Quality of the health data and guidelines used

Alagiakrishnan-2016

Others reported annoyance when a calculation was not performed for lack of current height, weight, or laboratory test information. Clinicians felt that either old data should be used or the prompt should be suppressed

They trusted the guidance because they could quickly discern that it had been reviewed and updated by a reputable organization.

Ash-2020

Many clinicians expressed the desire for truly evidence-based clinical decision support.

If you could tell me that, “lately the evidence shows that this is the best prescription for returning to work” that could be very (good) for me…. Yet in other words, that would be very powerful if you said to a doctor, “I’m giving you evidence based suggestions here.”

I think that’s needed and if we have a speciﬁc template for what has, you know, evidence base … would deﬁnitely be amazing.

Or you are working and we just never updated it. I mean it’s just totally bogus. There’s a lot of bogus data.

Benda-2020

Participants also described concerns related to how the clinical content would be utilized in the predictive algorithm to create meaningful, reliable knowledge. There would be huge problems there because the [REDACTED] data is far in arrears ... then the scores aren’t contemporary anymore. – INF04 [Challenge]

Anomalies in the data sources used to create the predictive algorithm—in particular, timeliness and data quality—were of great concern and a potential challenge to implementation. This mirrored the hardware/software concern related to how the predictive algorithm would utilize the clinical content.

If no one codes for diabetes, and no one codes for amputated leg, it’s a refresh [ie, the data may be overwritten]. So, the patient is no longer diabetic, or has grown back a leg

Catho-2020

Importance of providing the sources of the guidelines

• GE_03 (F, senior physician): “When you explain something to a young doctor you like it to be evidence-based, to have the proof”

• GE_07 (F, resident): “I think we like it, as residents, to have a quick answer and we won’t necessarily read the sources. At my level, we trust [the guidelines]”

• TICINO_03 (TI_03) (M, senior physician):“They must be based on reliable sources and must be regularly updated!”

Dikomitis-2015

Disparities in Read coding was evident throughout the data, as some respondents indicated that they coded everything, whereas others did not use Read coding very often. This variability in the use of Read codes by GPs is a limitation to the usefulness of any tool that relies on such coding.

Gillan-2018

MPs were, in particular, cognizant that the more standardised practices necessary to implement AI would lead to better data. Both ROs and MPs valued that this could subsequently be fed back into the AI system to support more systematic clinical decision-making. MP0

Goetz-2020

The students added that a vPCP may have less diagnostic bias. They visualized the ideal

vPCP as incorporating large amounts of data from patients worldwide, potentially eliminating diagnostic bias towards locally frequent illnesses: “. . .it might catch some of the more obscure diagnoses or things that are frequently missed from a normal human physician perspective.” (Fourth year medical student)

Jackson-2017

It was unanimous among the participants that treatment algorithms should be developed from the ECCO guidelines

Johansson-Pajala-2017

The CDSS could be perceived to promote evidence based practice and thus patient safety overall. The RNs expressed that they continuously tried to reduce the number of drugs, and hence the risks of ADEs. They were supported in these attempts as they assumed that the obtained reports were based on the latest research and current guidelines. This increased their conﬁdence that the patients were receiving appropriate and safe treatment.

‘I’m thinking that it is a way [using the CDSS] to review and modify drug treatments overall -to reduce the use of inappropriate medication, I think that this in some way must be the overall aim’

Joshi-2020

“And I think the biggest issue was at the discrete data points…you want to know if you have a pneumonia…there's no discrete data field that says pneumonia in it… so it is a little bit more difficult to pull some of those discrete data elements when you're doing the data mining unless you have some sort of artificial intelligence that can read words and recognize terms...synonymous with pneumonia. So that's where I think everybody's biggest challenge is in trying to figure out how to make that work right.”

Klarenbeek-2021

Some professionals expressed concerns regarding the reliability of the output generated by the CCDSS because of scepticism about the accuracy of the system’s algorithms. They also questioned the adaptability of the system to vast diversity of clinical presentations and the impact of structured patient data on quality of decision-making as clinical nuances (e.g., psychosocial determinants) potentially lack in these templates. [Professional ID: 19] ‘I do not know how variables are deﬁned and processed by the system. How reliable is this? Who is going to check all these data?’

The majority of the professionals identiﬁed pulmonologists and nursing specialists in pulmonary oncology as key opinion leaders, as their support was perceived as essential for successful implementation in clinical practice. Those opinion leaders could provide input on the systems’ design and deﬁne parameters essential for MDTM decision-making. Pulmonologists were seen as superusers who could lead the implementation process and could inspire and encourage other disciplines to use the system

Lai-2020

From the point of view of those interviewed who are in the health industry, AI is going to revolutionize medical practice and be a true breakthrough thanks to the progress of research in this field. According to them, the real challenge they face is access to health data for the purpose of training machine-learning algorithms

Liberati-2015

[… Usually when preparing a guideline there are conflicting opinions, which means that too experts have conflicting opinions on one line- driving, then usually there is a compromise. [...] It is important to understand which publications have a real scientific weight and which ones not, with what criteria is it that publication was born and with what criteria it was born another ... there are different criteria. [...] Maybe you are looking for guilty of making the system more interactive, in order to add some information to the system, albeit I don't know if that way you become a little peril-colose because you lose control of those who have generated it rato. You probably should go back to whoever it is generated to insert changes ». (Surgeon orthopedic, setting B)]

Lugtenberg-2015

- Lack of trust in reliability of the source of the content •“Well, then it makes me wonder: do they own any stock options? Yeah, I know it sounds a bit silly. But it makes me wonder which pharmaceutical company is backing this?”.

- Lack of trust in currentness of content •“How current are the guideline recommendations? Are the alerts really up to date? That’s what you [the researchers] should include in your advice, that the content of NHGDoc should be updated on a daily basis”.

Morgenstern-2021

Experts frequently emphasized that most AI approaches are predicated on access to clean, high-quality data, which can be difficult to find for health applications.

… the fuel for artificial intelligence is data and the state of data, of health-related data right now is... It’s pretty dismal. [Participant ID # 13].

For example, while there has been significant interest in using AI to leverage clinical notes in electronic medical records (EMRs) for disease surveillance, the quality of this data is a concern.

I must say I’m not overly optimistic of the value of [applying natural language processing to EMRs]. […]I’m not [sure] what you get out of there that’s not already in structured format [and] that you can do consistently in a repeatable manner. […] It’s going to be highly variable depending on the EMR. […]I think it’s a value but […] I think it’s a big heavy job to do it across multiple EMRs and the [whole region] and do a good job of it. [Participant ID # 3].

It was also a concern that datasets used for AI may have selection bias, meaning participants that are not representative of the population as a whole. They also noted that there may have been inadequate attention paid to this issue when applying AI. Definitely [traditional epidemiological] studies have the potential for those biases, although we do a lot to try and minimize them or at least them and quantify the impact. And so, I think the difference is we’re not talking about this with AI. I haven’t seen any discussion of oh, we should quantify the impact of the selection bias in our, you know, neural network. Or I’ve seen much less of that. [Participant ID # 12]

Furthermore, it was pointed out that much of the data used with AI is generated as a by-product or for purposes unrelated to proposed applications. This can make it more difficult to understand potential biases

… the public are a lot savvier than we give them credit for and people are very good at distinguishing between corporations use of data for […] targeting advertising […] versus […] medical and scientific use of data to improve peoples’ health. […] We often are afraid that we’re going to be painted with the same brush that people paint […] [social media companies or political consulting firms, thinking that] if we use anything to do with that and if we even say the word data people are going to get really angry. [Participant ID # 6].

Finally, many datasets of interest to public health are owned by private companies, making their use difficult.

[This computer’s company’s] got data, [that wearable company’s] got data, there’s other firms that have... [This sporting goods company’s] got their systems, right. So, everyone’s got their systems. And there’s really no way for public health, if we felt we wanted to use that for surveillance purposes, we would have to go out and negotiate with each one. [Participant ID # 7]

Morgenstern-2021-supplementary file 6

With AI for the most part we're actually talking about passive data collection. Data that's collected for other purposes, which may or may not have been designed for that application that we're using it for. And it may have measurement error in it. [Participant ID # 12]

… if you want a public health AI platform that is going to speak to resolving health inequities, you better make damn sure that everybody is represented in your training data that you're using, you can't be leaving out any sector of the population. [Participant ID # 6]

Page-2019

Sensitivity problems were reported to be due to errors in documentation (e.g. uncoded allergies, allergy vs intolerance) and alert logic (e.g. checking not speciﬁc to the current inpatient encounter, failing to recognise intentional duplication such as split or divided dosing).

Pannebakker-2019

Several felt that expected features, such as family history or sun exposure, were missing, and a

few felt that the melanoma eCDS was too simple and not useful:

’I’m just concerned that it doesn’t ask about other things which are important, because maybe I would even refer a person who scores three, but has a lot of exposure [or a] strong family history.’ (F, 41–50 years

Patel-2018-additional file

PM was sceptical. PM later was a believer in the intervention due to the substantial improvement in data quality at the health service.

Main GP initially participated to improve health service patient data quality however realised the importance of HT in screening and prescribing according to guidelines.

Petkus-2020-supplementary file

CDSS are important to ensure that patients' management is in line with best evidence based practice.

“Risk factor analysis apps and AI for monitoring advice in hospitals is relatively low risk as long as the latter works. The problems will arise if the inputs are made incorrectly whereby it generates authoritative but misleading advice.”

Roebroek-2020

The recommendations were used to evaluate previous steps and to discuss and decide on current treatment plans, as this respondent explained: “TREAT is helpful in aligning treatment with the evidence-based recommendations. It can be used to start a conversation about treatment options and help explain why alternative treatment options might be more preferable.” [C11]

. In some cases, TREAT motivated clinicians to recruit professionals for missing resources elsewhere in their organization:

“I really feel a lot of the added value lies in the fact that we are used to recommending treatments we have available. TREAT reminds you of treatments you do not have available directly, so you can try and find those treatments elsewhere within the organization.” [C8]

On the other hand, some clinicians actually used TREA T as a driving force to try new steps in treatment without postponing them:

“I think it [TREAT] helps clinicians to stay closer to and be more professional in chronic treatment while remaining evidence-based without postponing the next step in treatments.” [C8]

Santillo-2019

Participants wanted more information about the evidence regarding the risks and beneﬁts of shorter versus longer courses of antibiotics. They also suggested providing information about current guidelines for antibiotic durations indifferent countries

Sawan-2021

Open-ended responses indicated that ACPs found the DBI score helped to provide an evidence based reference for their recommendations in the HMR report.

(DBI) Number value provided proof. [ACP 8] A (DBI) figure was good. [ACP 1]

However, one ACP felt that the DBI score did not add to their recommendations.

Doesn’t change my recommendations that I was otherwise going to make. [ACP 7]

Silveira-2019

There was a unanimous impression that the device has the potential to deliver updated information to health care providers and education promotion is a major feature of its use. It was highlighted that the device has the potential to promote implementation of evidence-based recommendations. Great potential to promote best scientific evidence implementation for the individual patient. [Family physician]

The clinicians reported that the cardiovascular risk calculator sometimes presented spurious results and made them confused about the correct estimated risk. This error was found to occur when the date of birth was not entered or entered incorrectly.

Sukums-2015

“To me the CDSS is an excellent system; it helps providers on how to do things right” (male medical attendant).

Sun-2019

“The characteristics of diseases in China have many differences with North America. […] people's attitude towards diseases is different” [2IBM01]

The Watson system has been originally trained by mainly North-American patient data. But different national contexts have different disease profiles (Liu, 2017; Xie, 2017c). As pointed out by one of the informants: “Because of the racial differences [between China and Western countries], the cause of a disease is different. For example, Western countries have more vascular-related diseases, while China has more hepatic diseases” [5GOV01]. As a result of this, there is less data available on disease profiles that are more widespread in China, but less frequent in Western countries, where the Watson system is originally trained.

the insufficient size of the available data pool – comes about because the adoption of AI technology in healthcare is still at an experimental stage and there are no large data sets available yet (Xie, 2017a). As remarked by a government official: “It is still at the early stage of research. […] There is still a long way to go to the market. […] Watson is trained by limited data. [The adoption of AI in healthcare] is still in the dark” [5GOV01].

the Chinese system lacks integrated and continuous data sets. As remarked by a hospital manager/doctor, and confirmed by one of the top managers at IBM China [2IBM02]:

At each point, it can be seen as big data. But what about when we look at the whole experience? [It does not qualify as big data] […] So far, China doesn't have such good patient data from diagnosis, treatment, and observation. [1HP05]

EBM databases are based on scientific paper publications, reports, medical cases, etc. As remarked by the director of IBM China:

Machine learning cannot work without an EBM database. […] The most important characteristic of healthcare is practicality. [2IBM01]

China does not have a consistent standard of healthcare data collection (Hua, 2017) in either private firms or hospitals. In private firms, this is because of the different interests each firm pursues. As stated by an IT firm manager: “Everyone [i.e., each firm] has different opinions on [health] data collection. […] Whose interests are being considered?” [3IT02]. In public hospitals across the country, data is also structured differently. As stated by a government official: “Which kind of data problem is there? […] Data structure is not consistent among hospitals” [5GOV01]. In addition, there are no standards on which data is to be included in the databases; this results in missing data that might be critical for AI decision-making. A top manager at IBM China makes an illustrative example of missing data collection and its impact on the capabilities of AI:

For example, after a surgery treatment, a patient will not get bedsores if he/she has a son/daughter. But this information is not captured [by the AI system]. This kind of data is critical for bedsore disease, but it is not included and collected by the database. [2IBM02]

Torenholt-2021

The PRO-tool was then adjusted in discussion between the physician and the six oncologists (Saltbæk et al., 2019). Therefore, to the physician, the algorithmic sorting constituted an extension of her own, and her experienced colleagues’ expertise, which ensured the authority of the algorithm:

I think that it’s a kind of inserted clinical judgement (. . .) I consider it a clinical judgement, which we made when we decided upon the thresholds (. . .) we had six consultants (physicians) contributing by looking at it, and if they didn’t want to call the patient at that time, then I don’t need to know anymore.

Trinkley-2019

The clinicians acknowledged that uniform acceptance of the CDS recommendations ‘can lead to a lot of harm’, especially if the recommendation was based on inaccurate or incomplete information

Van de velde-2018

Some GPs found it positive that CDS could identify and help filling gaps in the patient’s record. Some said that CDS would motivate them to improve the quality of their EMR. Another GP mentioned that requests to register extra data (such as BMI) should be limited:

Physicians should only be asked to enter patient data when this is having a positive impact on the patient outcome. [GP, Norway]

Some GPs emphasized that CDS has to be based on evidence-based guidelines that are up to date. The participants perceived the presented CDS intervention as a reliable tool. The certainty of the evidence should be clear for every CDS recommendation

Vedanthan-2015

This was based on the assumption that the majority of hypertensive patients would be referred by the home-based testing counselor within the dispensary's catchment area. However, a substantial number of patients had incorrect geographic information in the record, chose to seek care at a dispensary outside of their geographic locale, or presented at the local dispensary without having had community based testing but with elevated blood pressure during the dispensary visit. These factors often led to an incomplete or incorrect patient cohort on a nurse's device.

Watson-2020

“the fidelity of the inputs themselves are quite incomplete” while another emphasized that, “people need to trust that the score is accurate and that really only happens when the data is complete.” Further exacerbating the data challenge is the local customization of workflows and system configurations. As one interviewee pointed out, “[The data at] every EMR is different at every health system”.

Wickstrom-2020

The participants expressed engagement in positive terms, saying that they were excited about and looked forward to the challenge of staying up to date with technology and modern treatment methods
