# Supplementary material for: Stakeholder Perspectives of Clinical Artificial Intelligence Implementation: Systematic Review of Qualitative Evidence
Source: J Med Internet Res. 2023 Jan 10;25:e39742. doi: 10.2196/39742 (PMC9875023; doi:10.2196/39742)
Supplement: Multimedia Appendix 3 [file jmir_v25i1e39742_app3.zip › 2. Technology/2e. Who owns the intellectual property/2e. Intellectual property ownership.docx]

**Name:** 2e. Intellectual property ownership

Adams-2020

Many participants emphasized that any data used for research or development should not be identiﬁable (ie, traced back to them as an individual); for example, participants were willing to have their images, diagnosis, age, and sex shared, although sharing additional information such as where they live would require their explicit consent. Some participants were uncomfortable with the concept of data “ownership” and preferred the term “access,” especially as it pertained to the use of images for AI development.

Alaqra-2020

While some might argue that since the patient identiﬁers are usually separate from the ECG signal data, and that the ECG signal alone is not identiﬁable; most of the interviewees regard the ECG to be sensitive requiring strong protection, and even likened the ECG to biometric data such as a ﬁngerprint.

several participants praised the notion of having a privacy impact assessment (PIA) for the use of the PAPAYA platform conducted by the service provider.

Ash-2015

Even so, some clinical sites do not freely share because they would like compensation or they believe sharing could cause liability problems. Another hospital analyst noted that an EHR vendor may be averse to the idea of customers sharing content they paid for but the vendor developed for them: “[CDS rules] created by the vendor consultant are the client’s property but should not be shared because the vendor wants to continue to make money off it [i.e., by selling the content to other healthcare customers]”

However, dialogue is time-consuming and difficult, and because the EHR vendors are often customers of content vendors, it can be especially complicated for them to speak freely. An EHR vendor representative praised the work of the HL7 standards body for promoting open dialogue in a legally safe environment: “in HL7 meetings you would have the best developers from each company come together and they would share ideas in a non-competitive environment”.

Goetz-2020

Data security. This theme had one subtheme: information theft. Each group was concerned about the potential for their personal information to be stolen, sold, or otherwise shared without their consent:

“. . .it could be sort of sold, it could be hacked, it could be taken into a wrong direction.” (Fourth year medical student) “I would never consult a virtual physician. . . The data. . . would go into hands of someone that I do not trust.” (First year graduate student)

While medical students generally did not trust vPCPs, citing data privacy and potential for misdiagnosis, the engineering/data science students were supportive. “. . .we certainly live in an era of big data, but like, I think that I would not be okay with sharing my information. . . I don’t even care if it’s depersonalized. I don’t care if it’s numbers attached without my name. I wouldn’t want to share my information with anybody.” (First year med student) “

Jackson-2017

Privacy concerns were identiﬁed as a potential barrier to implementation. However, the Manager of Strategy and Engagement of IT Services commented that ‘delivering care outweighs the risk of security’ in this setting. Participants agreed with this statement. Methods to minimise the risk of security were discussed and included the ‘minimisation of email content’ and ‘entering more content into the eHealth intervention as soon as it is available’.

Jutzi-2020

Participants feared that their data might not be anonymized, and hence might be misused to their disadvantage by health and other insurance companies or employers. Also, there was a concern that personal data might be published by hackers.

Kendell-2020

However, there were a number of reservations expressed regarding this approach. The primary concern about the use of an algorithm was privacy. Specifically, participants were concerned that individuals other than their physician (e.g., insurance providers, staff at other facilities) would have access to the information generated by the algorithm. “I don’t see anything wrong about it but, uhm, again I guess I suppose computers, you are always wondering if this goes out to insurance companies and whatever.” [Nova Scotia, Participant #3]

Lai-2020

all participants agreed that an immense amount of individual health data were essential to develop reliable AI tools for health. However, they also pointed out that a balance still needs to be found between widening access to data and ensuring confidentiality and respect for privacy

They pointed out the question of data ownership as being very difficult, because people believe that their data belong to them, whereas this is legally not true. The notion of “non-belonging health data” appeared to be problematic. Indeed, some participants highlighted that this notion did not respond to social reality. Indeed, the common belief is that an individual owns his/her health data. Moreover, clarification was also needed about the duty to inform or give consent following the application of the GDPR.3

They also complained about the difficulties induced by legislation when it comes to collecting data for researchers. According to them, this was the only way to see their research funding increase and thus allow effective development of AI and a guarantee of its quality.

McCradden-2020

The world that we live in, there’s all kinds of access to information even though it’s protected, but you hear all kinds of scenarios where sensitive information gets leaked. So yeah, I would have some concerns. (Participant 18–042, patient)

You should have to give up some [privacy]. … You want to be cured and [the company is] providing you with this cure, so you balance it out. (Participant 18–012, caregiver)

I think … in a democratic society, for members of the public to have faith in the health care system … individuals need to believe that what they believe to be confidential is held confidential, and not shared. But also for me to have confidence in health care systems, I have to believe that leaders in health care systems will make decisions for the greater good of people, right? (Participant 18–032, patient)

Most participants felt strongly that selling health data to private companies should be prohibited entirely. The few who disagreed argued that loss of privacy is an acceptable sacrifice for the prospect of benefit to the larger population, indicating that, as long as the product being developed would help people and adverse effects were minimal, selling health data was justified. Others described the difficulty in not knowing what kind of product would be developed; 1 participant noted “every company thinks [it’s] honourable, but it depends on your perspective” [participant 18–002, patient]).

Overwhelmingly and regardless of their view, participants advocated for transparency about how health data would be used, communicated openly by a trusted institution or custodian of health information.

No health care providers felt that selling either identifiable

or deidentified data was appropriate. They perceived that selling data conflicted with the responsibilities of health data custodians. One provider described patients as a “vulnerable population,” as patients are eager to support any endeavour purported to help others with the same disease, even if they know they themselves will not benefit directly (participant 18–010, provider). The idea that research might be able to “find a cure” was echoed repeatedly in this context by patients and caregivers, seemingly supporting providers’ views.

Morgenstern-2021

Privacy concerns were acknowledged as another concern when obtaining data for public health purposes. Yeah, some hospitals, their privacy officers are just so concerned. There’s such a fear now of, you know, the potential for re-identification and privacy. Sometimes it’s about the time and resources for them to come on. It’s about risk and liability and it’s really unfortunate. We’ve done like just so much work. We’ve spent so many hours. We’ve had legal counsel involved. Uh, just to overcome privacy concerns. Yeah, it’s quite an issue. [Participant ID # 8].

Nelson-2020

nefarious use of AI

Sun-2019

“The data is in the hospital. [IT firms] cannot get the data. […] For example, Alibaba is entering the health industry. But hospitals only allow Alibaba to access data of outpatients, not data of inpatients. They [the IT firms] cannot get the core data [continuous data of inpatients] from hospitals.” [5GOV01]

IT firm managers mention the current lack of ethical guidelines for sharing data in connection to AI:

Data comes from each person's sharing. […] But now the shared data has become a core competitive advantage for a certain organization. This [some organizations keeping the patients' data for commercial purposes] raises a number of ethical challenges. [2IBM01]

This raises a new dilemma: who should own the data? While the Chinese government has some thoughts on data storage and data using, this issue has not yet been resolved. As remarked by a government official, speculating about possible alternative ownership of the data: If the data belongs to the hospital, it is hard to use. If the data belongs to a firm, there may occur a monopoly problem. […] Maybe one way is for the government to develop a data center to push this issue. [5GOV01]

Vedanthan-2015

DESIRE's secure password and storage in a safe instilled a greater feeling of health information security:

Participant:.... it's confidential

Moderator: confidentiality is priority, okay, than those files that you have all over the room, anybody can come in

Participant: even the cleaner can sink into the papers (laugh)

Wickstrom-2020

The participants experienced that their engagement was positively influenced by the possibility of using a technically secure way of communicating with other caregivers:

If we can find a system in which I can securely transfer information to a colleague in another organization, that would be fantastic. [Participant 8]
