# Supplementary material for: Stakeholder Perspectives of Clinical Artificial Intelligence Implementation: Systematic Review of Qualitative Evidence
Source: J Med Internet Res. 2023 Jan 10;25:e39742. doi: 10.2196/39742 (PMC9875023; doi:10.2196/39742)
Supplement: Multimedia Appendix 3 [file jmir_v25i1e39742_app3.zip › 2. Technology/2f. Care pathway positioning/2f.1 Extent of tools' independence.docx]

**Name:** 2f.1 Extent of tools' independence

Adams-2020

Most participants supported an integrated approach that relies on the advantages of both radiologists and AI and felt that a radiologist is still critical to interpret the AI output

Biller-Andorno-2021

An AI- based support system should assist, not replace human judgement. It should make suggestions, not decisions Then I would say: ‘Okay, now that’s a good hint […] and then I would feel obliged to somehow verify it myself.’’ (Interview 8

Ultimately, the decision should remain with humans who can choose if they want to be supported by an AI or not—but what if evidence starts showing that AI- based predictions of preferences are more accurate than what a human would have predicted? ‘At the end of the day, I think that people should still make the decision, at least for the time being. Although there are clues that AI may decide better than humans, I think this has been seen in diagnostics.’ (Interview 9

Blease-2019

Supporting this perspective, some respondents were adamant that advancements in AI would buttress rather than replace the core roles of GPs and “help GPs with workload issues” [Participant 517].

Be useful to develop AI to do analyses of pathology returns, and read all the letters, to provide another presence in the consulting room, and to write the referral letters, organize investigations and the like, ie, act like a personal assistant might do. [Participant 135]

Bourla-2018

Delegate a monitoring task to a machine

Catho-2020

The fear of errors caused by blindly accepting suggested recommendations was another issue raised by some participants who also stressed responsibility of physicians who should remain those who make the ﬁnal decision

Dalton-2020

Overall, participants expressed positivity toward the concept of the intervention, and would welcome computerised interventions like SENATOR to be an aid to prescribing in multimorbid older adults. However, most participants recognised that the computerised recommendations could not be trusted without careful consideration, and that appropriate clinical knowledge is required to judge if recommendations should be implemented or not.

I would not trust them blindly. I have gotten bad and good recommendations, and that’s just because the computer programme can’t know the whole story. [Medical Prescriber 3]

Flint-2019

Of those that did engage with SPOT, common themes included using SPOT as a “double-checking

device” and as a safety check as identiﬁed by the focus group that highlighted reassurance as a beneﬁt of SPOT.

Goetz-2020

Participants noted that the vPCP could provide knowledge support to a human physician.

“If I was in the room, and essentially got two doctors, basically, cause you got the machine and the doc, that would be another cool thing.” (Fourth year medical student)

Students agreed that the vPCP would be a tool for the human physician, but could not

replace them:

“Not necessarily as a stand-in for a physician. I just see it as a way of having 10,000 brains about this one problem as opposed to just one.” (First year medical student)

The students also noted that any data or algorithm used by the vPCP would need to be

checked and verified by human physicians.

“You end up building some kind of algorithm and have it take all these factors in consideration, then you test it. . . you have human physicians check it. . . anytime the computer makes a diagnosis or prescribes a treatment or whatever, then a physician would look at what the computer’s doing and say, okay. Yes.” (Fourth year medical student)

Haan-2019

They indicate that Al in radiology is a nice instrument to double check the conclusions of the radiologist, but only using Al could result in restricted views with wrong diagnoses

Hallen-2015

Even though prognostic estimates produced by CPMs were judged superior to physicians’ own estimates, physicians felt CPMs had more utility as a conﬁrmatory tool, validating or ﬁne-tuning rather than substantially changing their pre-existing estimates:

Hospitalist 1:

I think it would be very helpful [to have a tool] and I have to say oﬀ the top of my head I would [use it] mostly for either conﬁrming or not conﬁrming my clinical instinct.

Internist 2:

to actually have numbers come out of this ... [CPM] would improve what you’re already doing or add to it. I guess it would ﬁne tune it.

Geriatrician 4: I’m just looking for more ways to validate what I’m seeing with patients or give me some contradictory information, you know to have another look at the patient, because maybe I am missing something entirely or maybe I’m seeing too much.

Geriatrician 3: If I were in primary care practice and ran this tool on all my patients over 80, you know it would seem like maybe every now and then something surprising would pop up but then you might go, huh, I wonder why, and then .... it might make you look at their medications a little diﬀerently, it might make you talk with them a little bit diﬀerently... that sort of thing...

Joshi-2020

“[We] have to manage expectations that we are not yet at a point where these rules are going to be able to define sepsis and without help from humans…”

Lai-2020

Only one person, a researcher working in the aeronautics field, spoke about the human–computer interaction (HCI). For him, even though HCI is not specific to AI, questioning the automation of tasks and limits should be considered as one of the main goals of the integration of AI into healthcare tools.

Lee-2015

Many radiologists also stated that CDS, with future improvements, would further solidify their role as consultants and increase productive conversations with referring clinicians dealing with complex clinical scenarios. One radiologist described how CDS could help in development of a more efficient two-step process for ordering advanced imaging examinations in the future:

I think decision support is good.. I think you’d refer to it as an entry-level sort of advice. And then, on top of that, layer on radiologists’ consultation for the cases where the decision support is not answering all the clinicians’ questions or concerns. So I’d see it as a kind of a multitiered approach

Specifically, radiologists noted that the ACR appropriateness criteria, which formed the basis for the most of the CDS interventions in the MID, were not exhaustive enough to cover many prevalent clinical conditions that prompt advanced imaging. Explained one radiologist:

The way the ACR appropriateness criteria are designed is that [the process] starts with a condition, symptoms, or a cluster of symptoms. Then CDS shows you what the appropriate tests are. [However,] the way ordering is done currently is that a clinician goes in and selects the test first, and then they try to justify it. I think one of the issues that we’re currently struggling with in terms of cleaning up the interface for the CDS is that people had to go backwards and assign appropriate indications for different studies, and they’re sort of pulling them out of the ACR appropriateness criteria, sort of backwards for how they’re listed. You almost wonder if, with CDS, it would be a better way for the clinician to say: “I’ll start with the symptoms, low back pain or whatever. Then, is it greater than a month? Yes. Do they have radiculopathy? Yes.” And then [CDS] tells them what the correct study is to order, and that may be a way for radiology to try to make it better for the clinician, rather than [asking physicians to pick] a study that they may not have ordered very often and then justify it

Lennox-Chhugani-2021

When asked what kind of actions they thought would mitigate some of their concerns, the women suggested that breast screening process would always need to involve humans. For some women this meant human oversight of the AI technology which undertakes most of the activity including decision- making. For others, the human role is pre-eminent, with AI used only to augment clinical activity and decision- making.

Liberati-2017

We see a lot of patients every day, most of them with comorbidities and a lot of medications… It’s not unlikely to make a prescribing error, we are human after all! [...] To have a support that can double check for us, and provide us with updated, ad hoc evidences… I think it’s a blessing!” (Physician, setting C1)

We hear things like “The CDSS is just a stupid machine that cannot give me orders!” Well, I think the CDSS is indeed a stupid machine, but that stupid machine can sometimes be crucial to avoid mistakes. (Physician, setting C2)

The CDSS is perceived as a working tool at the service of its users, which can complement their competencies and skills, rather than threaten their professional autonomy

McCradden-2020

A minority of respondents readily accepted the idea that an output from a “computer” should allocate patients to treatment or no treatment based on a prediction from a computer regarding their probability of benefiting. The lone provider who agreed with this idea likened this to the obligation to not offer treatments that are unlikely to benefit a patient. Those who resisted this notion appealed to fairness or equality (“trying is more important” [participant 18–008, provider]), fair opportunity (“everyone deserves the chance to be treated” [participant 18–017, provider]), evidential uncertainty (“should do more research” [participant 18–015, caregiver]) and individual factors influencing prognosis. All but 1 provider rejected the notion of allocation of treatment by AI, appealing to the need for these decisions to be made collaboratively with patients.

Morgenstern-2021

In a related sense, experts pointed out that AI may prove capable of sifting through big data to facilitate knowledge translation broadly, as well as the creation of guidelines

Morgenstern-2021-supplementary file 6

… it's imaginable that evidence to decision frameworks will be completed by machines […] That will still require human judgments. But also looking very far into the future, I mean the way that things are developing, it's not totally unreasonable that, you know, you would have recommendations produced through machine learning exercises.

Nelson-2020

The dominant theme in both interview groups was the importance of a symbiotic relationship between humans and AI (45 [94%]). Patients envisioned AI referring to a physician and providing a second opinion for a physician. “The problem comes from replacing a person with [AI],” which this patient described as a “tool for a dermatologist.” Credibility (30 [63%]) was another common theme that emerged. “I would probably need…feedback from a medical professional to…trust the app,” stated one patient, “because it’s like a black box…Algorithms with databases behind them…can make errors.”

The most common response in the event that human and AI reached conflicting diagnoses of melanoma and benign skin lesion was to seek a biopsy (32 [67%]). As one patient put it, “Let’s get the biopsy and find out what the story is.” The second most common response was to “put more faith in the doctor” (29 [60%]).The third most common response was to seek an opinion from another physician (20 [42%]). “I would get another opinion from another human,” a patient stated, “another dermatologist.” And the fourth most common response was to seek longitudinal follow-up from the same physician (11 [23%])

Pannebakker-2019

However, GPs were keen to discuss the importance of their clinical expertise and knowledge of their patients, and how they placed more value on their clinical judgment than the melanoma eCDS:

’Our clinical knowledge still has to come through, you know, and it has to be tailored to individuals ...’ (F, 41–50 years) ’I’ve referred ones that didn’t score very highly. I’ve not referred ones that did score highly. I think they are helpful but I wouldn’t tie myself to them entirely.’ (M, 40 years)

Ruppel-2021

[The interviewee, on the other hand, pleads for a research strategy that is even more strongly guided by the potential of computational technologies: “I don't want any constructs. I want to generate that from the data. " (P15: 1063-1064) To make this possible, the scientists should abstain from all analytical steps as far as possible and instead delegate them to “the machine” (P15: 1066). With regard to the identification of biotypes, a picture emerges in which the task of the researchers is less to remove layers and collect findings in small-scale work, but rather to create suitable conditions under which the data can, as it were, be discussed: “Allowing the data to speak for themselves:”]

Yang-2019

Seasoned physicians and surgeons voiced their appreciation for what a prognostic DST might bring, stating that it would “give its perspective” and offer a chance for an “occasional recalibration.”

Clinicians appreciated that DSTs could inform their discussions, “though the discussion is unlikely to center around the DST."
