# Supplementary material for: Stakeholder Perspectives of Clinical Artificial Intelligence Implementation: Systematic Review of Qualitative Evidence
Source: J Med Internet Res. 2023 Jan 10;25:e39742. doi: 10.2196/39742 (PMC9875023; doi:10.2196/39742)
Supplement: Multimedia Appendix 3 [file jmir_v25i1e39742_app3.zip › 2. Technology/2f. Care pathway positioning/2f.2 When and to whom the tool responds.docx]

**Name:** 2f.2 When and to whom the tool responds

Alagiakrishnan-2016

Some respondents recognized that suboptimal use of the EMR, particularly lack of medication reconciliation, could lead to false-negative CDS:

The danger is because we don’t always update our med review and then the [CDS] doesn’t pop up when it should sometimes the one-to-one meds review with the patient or caregiver does not take place.

Andrews-2017

One participant had experience using an app for mental health support with younger patients which featured alerts. In her experience, these prompts could be annoying or upsetting for patients.

P3: a couple sort of like said, ‘well actually, it kept reminding me, at sort of like inconvenient times’, things like that, or one person said it just made them feel, it put them in a bad mood, because they didn’t want to be reminded of how they were and it sort of like reminded them.

Ash-2020

Each one of them seems like a simple question but that question might take ﬁve minutes of explanation of, you know, tangents, of emotion. And all those things are not bad necessarily but in 15 minutes to see a patient, you can’t usually deal with them. So this kind of thing particularly is an area that I think we should be leaning toward and that’s decision support for patients.

Benda-2020

A strong theme was that the information should not create more work for providers. In addition, many of the appropriate responses to the HNHC classification (such as home or telephone visits, or referring to social services) were seen as potentially out of practice scope for these sorts of providers. Therefore, stakeholders suggested that the score should be integrated into team-based activities and used as a means for delegating tasks to appropriate staff.

They’re [primary care providers] looking for things that can offload them, not burden them more. – OPS05 [Facilitator]

Chow-2015

‘. . .The consultants make the decision. I don’t think they would wait for you to check ARUSC’ [J2]

Clyne-2016

Where reviews were conducted without patients present (adaptation), GPs made notes in the patient charts regarding any changes to specified medication(s). One practice did not undertake face-to-face reviews with patients by choice. The second practice conducted the reviews without patients present due to study time constraints. Both were single-handed practices and the GPs were confident that their patients would accept the changes:

Dalton-2020

There was no clear consensus on when the recommendations should be provided—some participants suggested they were provided too early as the patient was still at an acute point in their hospital stay to make pharmacotherapy changes, whilst others stated they may have been provided too late as prescribers may have preferred them immediately when reviewing patients on admission. However, what was clear was that the recommendations were usually not provided at the time the patients’ medications were being reviewed by the prescriber.

…there was a disconnect between when I saw the report and when I saw the patient, which kind of made it hard maybe to implement any changes that may have seemed reasonable. [Medical Prescriber 1]

If, however, the recommendations were related to the reason for admission, then this would increase their relevance, facilitating recommendation review and implementation.

…if it’s related, if someone’s come in with a fall and then it’s related to the admission, I’d be much more likely to look at the SENATOR recommendations in detail. [Medical Prescriber 6]

Grau-2019

Participants discussed the vicissitudes of the inpatient environment, how firing of the E-STOPS components fit with an unpredictable inpatient workflow, patients’ acute versus long-term needs, and providers’ customary practice. The desire was for the firing of E-STOPS to occur at the most appropriate times during patient evaluation, treatment, and discharge. Many suggested preferred times for E-STOPS to fire and for repeat firings to occur. Possible suppression of initial firing until acute medical needs are addressed or the patient has been seen and their desire to receive NRT while hospitalized has been discussed was also frequently mentioned.

Lack of familiarity with the EMR could influence E-STOPS behaviors and ultimately become habit. These observations raised the question for researchers about whether ESTOPS activation should be delayed until new staff become accustomed to the EMR

The first was the belief that inpatient treatment for tobacco use was only appropriate for specific admitting diagnoses, most often identified as chronic obstructive pulmonary disease or cardiac problems

Finally, participants often rationalized their reluctance to use E-STOPS based on their expectations about patients or (less frequently) the belief that the patient should always initiate the request for treatment.

P13, Hospitalist, female: This discussion, while it is important, I don’t think it needs to be done by me in the middle of the night when I only have about twenty minutes or so to see this patient.

P9, Internal Medicine, female: I definitely think that while they are already not smoking while in the hospital that that’s a perfect time to bridge them to quitting or cutting back as they leave

Internal Medicine, female: I always try to be cognizant of the amount of information that I give patients during a hospitalization. I do feel like I overwhelm them as it is with just their inpatient [problem]. I just don’t know that they’re ready to take one more thing

Henshall-2019

Psychiatrists felt that the DST was unsuitable for use in primary care as general practitioners may lack the knowledge or experience to prescribe antipsychotics. However, some patients/carers felt it could be used by general practitioners as a referral tool or to help monitor psychiatric patients in primary care, especially if patients were unable to see their psychiatrist immediately due to long waiting lists.

You’d see a doctor a lot quicker than you’d see a psychiatrist if you’re feeling awful. Wouldn’t you want to see your doctor pretty quickly for a potential change of drug that might assist you, rather than wait? Patient/carer 1

Keogh-2019

While it was agreed across all of the focus groups that most women would be able to input the data at home, consumers took a strong stance on how women should receive their risk assessment and advice. Our previous research had indicated that clinicians favoured risk assessment being delivered in a medical setting, yet the majority of consumers said that women should have the option to receive their result at home. Many consumers felt the involvement of their PCP would be a barrier to them using iPrevent (Table 5, Quotes 12&13). Barriers were caused either by time and cost associated with visiting a PCP or fear that a PCP's recommendation may conﬂict with the advice provided by the tool (Table 5, Quotes 14).

If I had to be sitting there going through it with my [PCP], I wouldn't do it. 13 I lost interest the minute it told me to go to my doctor to get the results. It's difﬁcult to get to the doctor. I have to do it between 9 and 5, Monday to Friday. My doctor, it costs me $40 to go. I'd have to book a double appointment for this. And the language is plain spoken … It's not like you're using any medical terms at all. I can read through all of that information myself. 14 For me, I don't know if I am just being, but some of those things sound quite extreme measures … Tamoxifen, surgery, whatever. Not saying that they're bad or that I wouldn't do them, but they wouldn't be things you take lightly, but if you sort of sit with a PCP, you get all that stuff, and you go, wooah, that's something I should maybe do, but then the doctor may not think that you should do it, and that creates a lot of confusion and conﬂict and you go, well why can't I have it? And it creates a lot of stress.

Lai-2020

AI tools could have an impact on the organization of the healthcare system, as they are not intended to be developed only in the care setting. Thus, for them, the public could well take advantage of these tools (e.g. self screening). AI

Lytle-2015

Recommendations for changes to the CDS tools and the EHR related to falls included (1) having the fall risk assessment question that was specific to admission (fall within 3 months prior to admission) display only on admission

Miller-2019

“Ideally screening could be integrated with regular clinical care using devices available within each patient room. Positive responses could cue clinicians .. . to order testing, provide treatment, involve other resources such as health educators, .. . and link them to resources available in the community.”

Nelson-2020

The greatest perceived risk of AI for skin cancer screening was increased patient anxiety (19 [40%]), identified more often by patients in the direct-to-patient interview group.

Another commonly perceived strength of AI was patient activation (19 [40%]) to seek both health information and health care. This strength was identified more often by patients in the direct-to-patient interview group. “Rather than…pondering for weeks or months whether it’s time to go see the doctor,” one patient noted that AI could be “an immediate indicator.”

Patients perceived AI as both a dynamic and static diagnostic tool (25 [52%]). One patient suggested, “Maybe if a mole was changing, there would be a way to track that.

Nicks-2016

would like to be able to provide the version [of the printed educational report] that we are working on in the home to them at that time. I don’t know how to do that, but that would be a smooth delivery of what you just talked about and that seems to me that they would see an outcome to their question and answer phase much quicker.” (SNS-H program nurse

Nova-2020

I think that workflow is a really key thing. You know, how do we, sort of, embed this into a primary care providers workflow and then how & they act on those things within their workflow. (Dr A)

Orchard-2019

About one quarter of practices found that screening patients intensively for a shorter period worked well. Perhaps it is easier to maintain motivation and ‘momentum’ for a shorter period. For example, screening during dedicated flu clinics worked well in practices where there was extra time allocated for nursing staff and a clear protocol for dealing with abnormal results.

Philips-2015

Three comments pertained to the need to extend the service from 5 to 7 days,

Rapoport-2020

How participants used the tool

… I do the majority of the assessment, … then I’ll present my case to the physician … in between that time period … I used it at that point … it was easier for me to do it after the fact. But it’s still while the patient is still here. [NP08]

Trinkley-2019

There was no consensus regarding the most appropriate timing of an interruptive alert. Roughly equal numbers of clinicians preferred the CDS to alert at different times such as: (1) the first opening of an encounter; (2) when ordering a medication or reviewing medications, (3) entering the patient’s visit diagnosis or (4) at the end of the encounter

To minimise instances when the CDS is considered irrelevant, they desired the CDS to be patient specific and provide assistance only in the appropriate context, considering the setting and clinician.
