# Supplementary material for: Stakeholder Perspectives of Clinical Artificial Intelligence Implementation: Systematic Review of Qualitative Evidence
Source: J Med Internet Res. 2023 Jan 10;25:e39742. doi: 10.2196/39742 (PMC9875023; doi:10.2196/39742)
Supplement: Multimedia Appendix 3 [file jmir_v25i1e39742_app3.zip › 2. Technology/2f. Care pathway positioning/2f.3 How and where the tool responds.docx]

**Name:** 2f.3 How and where the tool responds

Abdi-2021

“Could be useful for home screening and helping to access health care”

“These will be essential to remote healthcare

Andrews-2017

Some participants suggested that predictive technology could be implemented within the same Telehealth system.

P2: So that could be used in, with Telehealth, couldn’t it? So they could do their inputting, or, however they, answer the questions could be through Telehealth, as well, if they’re not seeing us every day.

Beede-2020

nurses had a large degree of autonomy on how they organized the screening workﬂow, and different resources were available at each clinic. For instance, one of the clinics in Chiang Mai was part of a larger hospital and had an ophthalmologist on staff, while the others relied exclusively on on-site nurses to read the image. Some clinics in Chiang Mai had the ability to use dilation drops at their discretion, but none in Pathum Thani did. At a clinic in Pathum Thani, eye screening was organized in an assembly-line fashion, with a camera technician taking the fundus photos and a nurse leading the consultation with the patient.

Dalton-2020

The location of the report may also have been an important factor. Interviewees stated that prescribers simply may not have seen the recommendations, which could easily go unnoticed in the medical notes or in an email inbox.

Well it could be because people didn’t look at it… if the information doesn’t get to them, they probably don’t accept anything…. [Medical Prescriber 10]

Dikomitis-2015

We received a range of conﬂicting responses with respect to how well the eRATs ﬁtted into current practice. For example, the tools sat within an electronic system separate from the clinical system, which necessitated additional log-on. This was reported as a barrier by some users. An additional factor in the GPs’ perception of the eRATs’ compatibility with their clinical systems was whether iCAP or Audit + (which runs on top of the iCAP) was new to them, which was the case for approximately half of our respondents: ‘I think this issue of the computer resources is important. It’s got to be something that becomes a regular part of your practice, really. So from that point of view, certainly the IT people ought to sort that side of things out’.

Goetz-2020

Students noted that using a vPCP would eliminate transportation demands for the patient:

“I was ill last semester. . . And it would have been really helpful for me not to have to drive to a clinic. . .” (First year medical student)

Participants felt it was advantageous that the vPCP could be accessed anytime and any-

where, including in rural areas and underdeveloped countries where access to healthcare is limited.

“. . ..you can basically contact whenever you have the problem.. you can access it really easily.” (First year graduate student)

Grau-2019

E-STOPS use appeared more likely to occur when providers believed that inpatient settings are an appropriate environment in which to encourage patients to quit or try new approaches to smoking cessation—particularly for patients who lacked access to ambulatory healthcare services and may have never thought about quitting

A second barrier concerned the belief that, with little or no opportunity for patient follow-up once discharged, the issue of tobacco cessation was more appropriately within the purview of outpatient primary care.

Hospitalist, male: So I think if it were something that when you were entering the admission orders, it was just part of that instead of being a best practice alert. Since the study [started], many more best practice alerts have been added, so people sometimes develop muscle memory to just dismiss them

P10, Hospitalist, male: These patients may not be regular consumers of healthcare or may not have regular follow-up. So, I think that when they’re hospitalized, it is an opportunity [to intervene]

P1, Internal Medicine, female: I make big differences in primary care that I don’t strive to in the inpatient side…And tobacco cessation, drinking cessation, those things are all outpatient primarily

Jackson-2017

Participants discussed the speciﬁc functionalities that the eHealth decision support tool should possess. A web based platform was thought to be more appropriate compared to a mobile phone application for initial testing of the tool. Participants agreed that disease monitoring should form a component of the tool. The recording of patient-reported outcomes (PRO), clinical disease indices and laboratory biomarkers in the tool were all considered feasible, but it was agreed that only validated outcome measures should be used.

Jutzi-2020

Conversely, AI could allow ﬁrst self-tests at home without visiting a physician—which could become even more important due to the increasing paucity of physicians

Keogh-2019

Facilitator: Would you go to the internet before going to a [PCP]? Consumer: Yes, I would. Because you get big amounts of information and you have it now. You don't have it in ﬁve days when you can get an appointment and for $35 out of pocket plus your Medicare rebate. And you can sit there and read for two hours if you want to, whereas you've got ﬁfteen minutes with the PCP.

Klarenbeek-2021

Professionals expressed need for change in current MDTM workﬂows. They also expected that most of those required changes could be addressed by the CCDSS. Besides the already mentioned MDTM preparation time and total MDTM duration, professionals are in need of methods to discuss complex and non-complex patients, to visualize pathology slides and to improve MDTM reporting. In addition, the majority of professionals believed limited additional resources and technical facilities were necessary to facilitate implementation of the CCDSS.

[Professional ID: 6] ‘The MDT report has to reﬂect the content of the MDT discussion. This is often not the case as essential details are not reported. Consequently, certain aspects need to be discussed again, making this process very inefﬁcient

Liberati-2015

«Here perhaps more than in the ward with us it could be useful in the clinic ". (Internist, setting B)

Miller-2019

Several had concerns that visit time will be taken up addressing issues that were not related to the chief complaint, leading to increased resource utilization, increased length of stay, and potentially misuse of the ED setting as a site of care.

Morgenstern-2021

Rather than simply personalizing health promotion, it

was proposed that we might apply social networking models to target public health messaging for maximal impact.

… machine learning plays a critical role in sort of identifying where your opportunities are to influence and, you know, if I look at who’s following whom on [social media], I actually only need to influence four people and now I can influence 400,000. [Participant ID # 4].

Patel-2018-additional file

GP would prefer HT to be incorporated within MD. He finds it frustrating that it is separate.

Rapoport-2020

Family physicians were generally more receptive to the idea of incorporating the tool into their routine and did not experience it as unmanageably time-consuming, especially if it could be integrated with their electronic medical records.

Reynolds-2019

Instead of me handwriting a system out while I can enter my dose, concentration, whatnot, into there, it will print all that out on the sticker, and I can attach to my drugs at that point...”

Roebroek-2020

All clinicians agreed that TREAT was properly imbedded into the existing technical infrastructure of the electronic patient record. Therefore, the facilitating factor referred by the UTAUT model was perceived as optimal and enhanced TREAT’s use

Wickstrom-2020

The participants were engaged by the expectation of being able to provide better service and equal health care to the patients regardless of where they lived, that is, the patients could be treated at home, especially patients living in the countryside far from health care
