# Supplementary material for: Stakeholder Perspectives of Clinical Artificial Intelligence Implementation: Systematic Review of Qualitative Evidence
Source: J Med Internet Res. 2023 Jan 10;25:e39742. doi: 10.2196/39742 (PMC9875023; doi:10.2196/39742)
Supplement: Multimedia Appendix 3 [file jmir_v25i1e39742_app3.zip › 3. Value proposition/3a. Supply-side value/3a. Supply-side value.docx]

**Name:** 3a. Supply-side value

Ash-2015

The companies want to continue to grow and change to keep a competitive edge. They are working to identify new markets and strategies and to pursue them aggressively.

However, there are some undercurrents of disappointment as well. One vendor representative expressed frustration that the customers were motivated more to increase their revenue than to improve patient care: “So while we have decision support...the bulk of it supports making sure they get paid.”

The overall vendor environment is increasingly competitive and deliberately non-integrated. In order to remain competitive as a business, the vendor must clearly brand products and keep pace with changing market expectations.

The clinical site employees had mixed feelings about the quality of training they received from vendors. Conversely, the vendor representatives complained that often, clinical organizations were too reluctant to pay for training.

Measurement of the use and effectiveness of CDS is a challenge. Representatives at one clinical site discussed how they had difficulty accessing data for reporting because the data were maintained in a proprietary format by their vendor. Other representatives perceived that the difficulty in their ability to generate reports was related to their vendor’s preference to be paid to generate reports for them. One

Ash-2020

The ability to build CDS tools inhouse is important because, as we were told by an IT staff member, “it’s going to cost” to have it built by the vendor unless the vendor decides to provide it for all customers.

Benda-2020

Participants also noted the large number of existing risk prediction algorithms and the challenge of differentiation.

What makes your tool actually any more accurate or unique compared to the 500 other vendors out there? – OPS09 [Challenge]

Joshi-2020

“When we were doing the homegrown model, that process actually took about a year-and-a-half. Because we would create the model, our own internal model, put it into the system, have it run silently in the background. We would notice some odd behavior or something that we wanted to tweak, we would tweak it put it back in the system, watch its behavior for a few weeks to months and then keep adjusting. It took a long time for us to get to the point where we felt comfortable enough to put it out.” (ML)

Lai-2020

Moreover, most of the people interviewed (physicians, industrial partners, participants without a conflict of interest, and researchers) expressed the opinion that AI development is increasing because of international competition.

Another matter of concern that the population should be aware of is the aspect of social justice. Some participants agreed that the primary motivation for the development of AI was financial. Thus, given the intrinsic logic of AI, namely the prioritization of the collective above the individual, they questioned whether there will remain a place in society for individual vulnerabilities.

Sun-2019

The IT firm stakeholders highlight the worries brought on by AI adoption in treating patient data. As claimed by a top manager at IBM China: “I am worried that some firms will abuse the shared data for commercial purpose” [2IBM02].

there is no shared official definition in the market of what AI technology is. This brings about uncertainty among competitors in the market. As remarked by the Vice CEO of EWELL, “Everyone [the firms] works on AI business […]. People [entrepreneurs] are so excited with AI. This leads to market disorder” [4IT01].

For a hospital, data means value, even if hospital managers still don't clearly know how to use the data the hospital owns to make profits. As remarked by a government official:

The data is in the hospital. [IT firms] cannot get the data. […] For example, Alibaba is entering the health industry. But hospitals only allow Alibaba to access data of outpatients, not data of inpatients. They [the IT firms] cannot get the core data [continuous data of inpatients] from hospitals. [5GOV01

Watson-2020

Only a couple institutions—those who stated they had the highest number of models in development—indicated no concerns with funding:

Some of the actual developmental work is funded from grants, some of it is done in a research collaboration under an NDA, and some of it is internally developed and internally funded from a variety of different sources, mostly institutional operational funds.
