# Supplementary material for: Stakeholder Perspectives of Clinical Artificial Intelligence Implementation: Systematic Review of Qualitative Evidence
Source: J Med Internet Res. 2023 Jan 10;25:e39742. doi: 10.2196/39742 (PMC9875023; doi:10.2196/39742)
Supplement: Multimedia Appendix 3 [file jmir_v25i1e39742_app3.zip › 3. Value proposition/3b. Demand-side value/3b.1 Time required for service provision.docx]

**Name:** 3b.1 Time required for service provision

Abejirinde-2018

“I: So in your opinion which particular place do you spend a lot of time? R: At the machine I: So where do you think when worked on, will help reduce the time spent? At the records, the machine, the palpation area, the dispensary?

R: No, just at the machine I: ….so the machine is what causes the delay R: Yes”

ANC workflow is usually structured in stations such that women have to move from one point to another for specific tests or different aspects of the consultation (palpation, updating records, weight, height and blood pressure check, collection of routine drugs). In this context, workers felt the integrated function of B4M made ANC routine easier and more comfortable

Adams-2020

Participants identiﬁed that new technology—new equipment and software, including AI—may result in decreased times for image acquisition and interpretation, creating opportunities to increase the volume of examinations performed and reduce wait times

Ash-2020

Interviewees consistently noted that our proposed CDS might add to their time burdens or to that of others in their clinics. As is the case with most organizations, some of these sites had received complaints from users about the number of alerts causing alert fatigue.

Like, nobody will use something if it slows them down even if it makes them more accurate.

Hands down, I think if you give a clinician a support tool that makes them ask all of these questions, I think it’s going to be very frustrating and take a lot of time.

Bourla-2018

Used to compensate for increasing shortages of health professionals in some areas

Catho-2020

Some physicians also mentioned the potential time saved by the use of CDSSs, which could allow them to focus on other tasks.

Gillan-2018

Gaining efficiency in practice with the implementation of AI strategies was framed in value to both patients and professionals. TP06 noted that ‘all things being equal, if patient throughput is increased, then it’s a good thing’, reducing patient wait times.

Guenter-2019

As one participant reported, “...I thought it was a great idea and I was enthusiastic...if it takes more than 3 seconds or something like that, it quickly falls off your priority list to do

Haan-2019

Patients expect that Al will allow more scans to be analyzed in a shorter amount of time. They report that this can have consequences for being assisted sooner and that this will reduce costs.

Johansson-Pajala-2017

perceptions ranged from that the system was time consuming to that it was time-saving.

A common perception was that the use of the CDSS in medication reviews had saved time. The new procedure limited the time required by not having to send documents to distant pharmacists and wait for their reply. Instead, the RNs received an immediate response which they presented to the physician, and necessary actions could be taken. Through this faster procedure, RNs perceived that they were able to ﬁnish the job and also see that their patients received help more quickly with drug adjustments. ‘Great that we do not have to send a lot of paper back and forth and back and forth, and that we get a quick result and a quick response’

Johansson-Pajala-2019

“It is time-saving [using the CDSS]” (N1)

Jutzi-2020

AI was also expected to improve processes, and to reduce the burden on healthcare by helping to avoid unnecessary diagnostics and treatments.

Klarenbeek-2021

In addition, it would reduce administrative burden. Less impact was expected on the quality and accuracy of decisions taken during the MDTM and on clinical outcomes because the professionals believed that MDTMs already facilitated high-quality lung cancer services due to the presence of extensive medical expertise and knowledge. Compared with existing EMR tools, the CCDSS would be able to offer insight into whether the data essential for decision-making was complete. This was seen as a facilitator for using the system as it provided guidance which patients should be discussed at the MDTM, reducing time and effort wasted on patients of which crucial information is missing.

[Professional ID: 17] ‘If the system provides a summarized and structured overview with all relevant patient variables, we do not have to extract those data manually from clinical notes and medical letters anymore. This will save us lots oftime.’ [Professional ID: 4] ‘After a patient case is introduced during the MDTM, it frequently becomes apparent that key diagnostic reports are not available yet. Time and effort is wasted on preparation and introduction for clinicians involved as these patients have to be discussed again in the next MDTM.’

Lai-2020

For many healthcare professionals surveyed, AI tools developed by industrialists would be able to save time for the doctor, carry out watchful and alert work, better monitor the population, alleviate some deficiencies related to medical deserts, and even improve management difficulties in the healthcare sector (especially at the hospital). AI could therefore be a means to enter an era of more effective medicine, improving care and reducing costs, while increasing patient safety.

Lawton-2014

The remaining participants (n = 5) claimed their primary reason for using their advisors was because they had a data storage facility:

‘‘because I’m on a 1:1 ratio it’s pretty simple. . . I mean the main reason I’m using it is because when I get round to downloading my results onto my laptop or whatever, it will offer me more information, it has all my dosages and carbohydrates on there as well.’’ (M15.1)

Hence, as M15 further suggested, using a paper diary, which would have been more burdensome and time-consuming, was not necessary.

Lennox-Chhugani-2021

Increased efficiency (n=162).

Help to address the workforce shortage within the breast screening programme

Melo-2020

One of the main impacts identified was the efficiency and effectiveness of healthcare.

Pannebakker-2019

Most were familiar with using the melanoma eCDS and felt that it was clear, useful, and easy to use. Some reflected on how using it did not intrude in a consultation, and that it could help with saving time during or after a consultation:

’It’s simple to use and it’s on the referral form so it’s going to be easier to use it because then you can put the score on the form, speed things up really.’ (F, 41–50 years) ’I think it’s more time-efficient because it’s tick, tick and you don’t have to sit there and write "changed in size" and "changed in this" ...’ (F, 40 years)

Patel-2018-additional file

GP: it’s a bit of a nuisance and apart from the fact that it is beneficial in terms of putting figures down and calculating it, it appears as if I’m working for somebody else with no compensation and no recompense and it’s very annoying sometimes, especially when the thing clogs my computer now.

Roebroek-2020

Apart from novelty, TREAT also increased consultations times by bringing up a larger array of topics for discussion:

“I think my consultations became longer, because I noticed some time shortage. Therefore, you probably take or just need some more time to discuss all the results. It depends of course, on what ends up in TREAT. If someone has few problems you are quicker to discuss everything.” [C12]

Shannon-2021

The technology that was developed or that was designed for the project was used in order to streamline the processes and optimize the time for both the patient and the doctor

Trinkley-2019

One clinician stated clinic staff could do ‘non-physician work that would free up physicians to do some of the higher level patient care management that we just sometimes don’t get to’

One clinician suggested a CDS recommendation ‘… could just say ‘would you like to order it?’ and you click ‘yes’ and boom, it just happens’ instead of needing to leave the current screen and enter the orders module. One clinician stated ‘if you save the clinician time, you'll always win’

Urquhart-2018

benefit the healthcare system via reducing healthcare provider stress and workload, for example, by preventing crisis situations or reducing the need to rapidly access needed services or supports for patients close to death because they were not anticipated or planned earlier, and ensuring more effective use of resources

Van de velde-2018

CDS should fit in the workflow so that it has no negative impact on the amount of patients seen by the clinician. [GP, Belgium]

Vanhille-2018

“Time required to create the post–virtual surgery models…” “Time involved to go through the models…”

Vedanthan-2015

Prior to receiving the device, many nurses felt that using a handheld device would be more time-consuming and confusing than managing hypertensive patients with the previous method of a paper form. Participant : I thought it was going to be a lot of work. Moderator: In what way?

Same participant: Like now you are now coming to write to the tablet, go through the tablet, like when you are seeing a patient then you swipe the tablet, the queue is outside, so I was thinking maybe it will be a lot of work for me.

Wells-2014

However, they raised doubts about its usefulness and the time required to use it.

Yang-2019

Mid-level clinicians enthusiastically welcomed the idea of a decision meeting slide generator. They envisioned a number of possible benefits. They shared that the slide generator would automate work that is not currently billable. At hospital A and B, meeting slides were prepared by staff who had little to no medical training. Physicians could get frustrated with the result

Both seasoned physicians and mid-levels expressed appreciation for DSTs that could slow them down “only when necessary".
