# Supplementary material for: Stakeholder Perspectives of Clinical Artificial Intelligence Implementation: Systematic Review of Qualitative Evidence
Source: J Med Internet Res. 2023 Jan 10;25:e39742. doi: 10.2196/39742 (PMC9875023; doi:10.2196/39742)
Supplement: Multimedia Appendix 3 [file jmir_v25i1e39742_app3.zip › 3. Value proposition/3b. Demand-side value/3b.2 Patient centred care.docx]

**Name:** 3b.2 Patient centred care

Abejirinde-2018

the structured format of B4M compelled prolonged woman-provider interaction leading to improved counselling that was otherwise previously rushed or absent.

Abidi-2018

The participants felt that although there are many diabetes educational resources, they want DWISE type apps that consider a patient’s personal preferences and psychosocial concerns, when designing self-management strategies. Bo

It helps me gain more information about diabetes-related behavior change and about my patient and both my patient and I can see if my patient is on the right track...we will have something to talk about next time we meet.

PCPs felt that DWISE could also improve communication between patients and PCPs around diabetes-related self-management. One of the PCPs said:

When a patient is first diagnosed with diabetes...DWISE can be a good avenue for discussion...about how a patient is feeling, what is it they want...how can they fit the self-management in their lives.

Patients also felt that DWISE could potentially help them to communicate personal issues that might affect their self-management practices and that otherwise would not come up during an appointment:

Doctors don’t live with diabetes...I live with diabetes...I have lived with diabetes for so long...this type of technology and apps can support me to better communicate with my doctor...what I am going through...why I am not able to follow proper diet or...not exercising..

Andrews-2017

Participants spoke of how the Telehealth system benefited patients in unexpected ways.

P5: it was surprising how a lot of the older adults quite enjoy it. (R: right, ok) There are some that struggle, maybe with dexterity, but there was a lot that took us by surprise, who enjoyed being monitored

Blease-2019

Technology won’t replace GPs as patient management is about negotiation and managing risks and different patients have different views. [Participant 703]

Please hurry up with the technological advances to take away some of the crap that I still have to sort out–then I will be able to get back to proper diagnosing and doctoring. [Participant 693]

Gillan-2018

Others acknowledged that time-savings in certain clinical areas, particularly relating to mundane but time-consuming tasks such as contouring and planning, would allow more focus on ‘high touch’ (RT02) or value-added tasks, final plan evaluation, managing complex patients and non-clinical responsibilities.

Aligning with the perception that AI would increase efficiency in certain areas of practice, there was also the belief that time saved by AI in certain areas would facilitate focus on other areas. In some cases this was noted to be tasks or roles that always existed but that could not be afforded the necessary time (i.e., patient care). In other cases, it would relate to being able to do more of something if it took less time to do it in the first place (i.e., adaptive re-planning). While there was some overlap, participants tended to envision different impact on each professional group

One physicist noted the benefit in this, that ‘sometimes I feel like maybe I’m struggling with checking all of the routine stuff…I would hope that I don’t have to spend that time there and I could use my expertise and focus on that extra conversation… maybe we have time then to figure out how to push that further with time that we don’t necessarily have to invest now’—(MP05).

This represented a common view that the relative time spent on quality assurance would not ultimately be reduced. A discussion within the MP group highlighted this: MP01: We can spend time in different places. MP04: I don’t think that will speed up our process of checking plans, unfortunately, but I think it will…. MP04: Right. SO I think that’s probably it. MP05: It can change the conversation we have about the plans, maybe? To something that maybe could focus on stuff that…. MP04: …actually matters! Focus on actual quality!

Goetz-2020

Students saw the potential for more personalized patient engagement when using the vPCP.

“. . .it would be nice if the AI came in and took exactly what the doctor said and was able to formulate something specific to you, you know, not generic, not just the standard machine jargon, specifically.” (Fourth year medical student)

Jacobs-2014

“insufficient complexity of CDS to account for patient-specific needs”

Johansson-Pajala-2017

Many RNs perceived the CDSS as an eye-opener. By viewing the quality reports they were alerted to possible connections between the individual patients’ condition and their drug treatments. The information could make them think in new ways or conﬁrm their own previous suspicions.

‘You might become a little more attentive to certain things... maybe a lamp lights up and you think oh well maybe that is why this patient is falling so much. Yes a little wakeup call sometimes’

Jutzi-2020

Participants also assumed that using AI in skin cancer diagnosis might ultimately result in more transparency, if AI based tools were able to objectively quantify the likelihood of a lesion being malignant objectively in a way that patients could comprehend

Keogh-2019

Consumers most often described the beneﬁts of iPrevent in terms of its ability to empower women; putting the information in their hands, allowing them to make decisions based on the best available data; allowing them to have more meaningful and informed conversations with their PCP; giving them more conﬁdence that there are things they can do to prevent BC (see Table 5, quote 6&7).

Lee-2015

Although many radiologists acknowledged that use of CDS may reduce the number of imaging studies ordered, they did not see the decrease in volume as a professional disadvantage. Rather, they saw CDS as a tool that could increase the overall quality of radiologists’ work. Explained one radiologist:

The number of appropriate studies will increase, and the number of studies will decrease. So the radiology department might be doing less cases. So financially you can weaken the hours spent, but the quality of what we do will increase

In general, radiologists agreed that they did not perceive CDS implementation as having an important effect on how they write protocols for advanced imaging orders. Approximately the same amount of time was required to review medical records to ensure the appropriateness of an imaging protocol. One radiologist explained:

I think the quality of the clinical information is significantly improved, compared to before [CPOE]. I’m not sure that the decision support piece of it has changed ordering as much as the change we have seen just from using [CPOE].

Lennox-Chhugani-2021

Release staff for higher value patient- centred activities.

Morgenstern-2021

Some interviewees went on to suggest that these real-time AI interventions could be used for more personalized health promotion, particularly through using social media information.

Morgenstern-2021-supplementary file 6

It's kind of like precision public health. So, so when you're looking at a given population [you] can assess the characteristics of that population in terms of, you know, from the indicators. [There’s] age, sex all that kind of stuff and [socioeconomic status indicators] and, uh, predominant health conditions and then [it can] basically recommend […] interventions that are evidence-based [and] that address the most important determinants of health in that population. [Participant ID # 3]

Muth-2016

Five of 10 GPs reported that the GP–patient consultation was a positive experience (‘clearly more systematic than regular consultations’;

‘more often focused on adverse effects’; ‘cooperation with patients has been improved’)

Nelson-2020

and patient education (11 [23%]).

Nicks-2016

Using SNS-H helped nurse home visitors cover injury prevention in a more systematic way.

“Oh yes it has been helpful for me because I would kind of haphazardly teach safety but now I am much more focused and much more intentional if you can go through and actually go through it and ask them a couple of questions. This was very helpful to me. I would like to move forward with this and set up an intentional program like this.” (SNS-H program nurse)

I found that . . . this has led to a lot of teaching moments that maybe I would have not covered with them had we not been discussing the small parts in the assessment tool so that brought up some teaching moments.” (SNS-I program nurse)

Patel-2018-additional file

occasionally when a patient is a bit difficult to understand and difficult about realising that he has got a risk with cardiovascular disease, then I produce the file in the hope of convincing them they have to do something about it.

… “I’m quite happy with that. That’s a good sign. Thanks, Doctor. Thanks very much, you know, you’re the first one who showed me”. So, you know, things like that do happen. Out of the 10 patients that I would use that with, about seven of them would say that.

GP: it’s worthwhile knowing, however when it comes to treating people, they want to be looked upon as individuals and they want to be shown where they are going, not relative to somebody else

Roebroek-2020

“TREAT also provides information about possible future steps in treatment such as for example electroconvulsive therapy. If recommendations are presented on a screen it feels more natural to address it as an option. You can inform patients of different options in case the current treatment doesn’t work.” [C8]

Even though clinicians held different opinions regarding the benefits of TREAT, nearly all of them agreed that it contributes to shared decision making (SDM):

“It [TREAT] did have a positive influence on shared decision-making. You have multiple options to choose from. That was most obvious with things like negative symptoms. You can tell someone music therapy or cognitive behavioral therapy is available, but scrolling through these options together makes it easier for patients to say: ‘that doesn’t suit me, but this is something I’d like to try'.” [C8]

Sawan-2021

These participants further reported that being asked their GOC by the ACP was beneficial as it gave them the opportunity to consider what they wanted to gain from their medications.

It made me think about what my goals are. I probably hadn’t thought about it until somebody asked me. [HMR recipient 1]

Shannon-2021

Providers also find that the intervention creates a space to speak about mental health with their patients, helping patients feel heard. As one physician described, “I have seen that with screening they [patients] open more like they had never spoken in the consultation, because they can talk openly. That seems to me to increase the uptake and detection of patients in the depression part.” By creating the space for patients to discuss their mental health, the patient-provider relationship is strengthened and patient care is improved

Vanhille-2018

“…can show patients that surgery will not get them much benefit over their medical treatment.”
