# Supplementary material for: Stakeholder Perspectives of Clinical Artificial Intelligence Implementation: Systematic Review of Qualitative Evidence
Source: J Med Internet Res. 2023 Jan 10;25:e39742. doi: 10.2196/39742 (PMC9875023; doi:10.2196/39742)
Supplement: Multimedia Appendix 3 [file jmir_v25i1e39742_app3.zip › 3. Value proposition/3b. Demand-side value/3b.3 Cost of healthcare.docx]

**Name:** 3b.3 Cost of healthcare

Abejirinde-2018

Analysis showed that pregnant women appreciated the device for detecting their health problems and saving time and money that would have been otherwise expended on diagnostic referrals

Andrews-2017

Participants discussed the cost of digital technologies where participants do not currently own them, and described how this could be a barrier to adoption if the cost of the devices fell on the health service. This was particularly of concern in managing the care of older people, since they were less likely than younger adults to own digital devices.

P6: With the sort of, screen thing that you’re talking about, there would be things like the cost to the organisation [..] who pays for it?

P5: technology as such, we have very limited… Predominantly because of cost.

P3: Is there a cost for us to buy technology for them to use, whatever kind of technology that is?

Participants mentioned that finding money to pay for these would depend on funding streams within the organisation.

any supplied technology may be lost, broken or stolen.

P7: It’s how safe would that be in someone’s house? Researcher: Oh right ok, in terms, safe as in, to not get broken? P7: To not get broken, or to not get stolen, or just get mislaid in general.

Ash-2020

When asked about other priorities, one IT manager said “well, ﬁrst priority is how it’s affecting billing.” Efﬁciency was offered as another priority: “and then it would be, you know, how can we streamline the process?

Money. It would have to help us generate more revenue.

Well ﬁrst priority is how it’s affecting billing.

Well, we prioritize based on funding, regulatory requirements, and clinical need.

Benda-2020

Participants in systems with more value-based contracts saw reimbursements as a facilitator, while those in fee-for-service dominated settings viewed the payment structure as a challenge to using the HNHC predictive algorithm. But there’s certainly more pressure now and more focus on it now than there was five years ago ... there are places that have 30, 40 percent of their dollars at risk. – OPS04 [Facilitator] There’s not that much of a return on investment for us, from a financial point of view ... there’s not that much that’s driving full adoption. – OPS04 [Challenge

Cai-2019

Beyond the information needed to make effective use of AI, pathologists also brought up several key factors that would influence their initial decision to adopt or purchase such a tool to begin with. These include: evidence of FDA approval and published validation in peer-review journals, social endorsement by well-respected medical leaders, impact on existing workflows, impact on legal liability, and cost of purchase.

Catho-2020

Several participants believe that the economic constraints of current healthcare budgets can promote the purchase of these tools that may help to save money in the long term

Goetz-2020

Students felt that using a virtual PCP would cost less than visiting a human physician. “Cheaper and faster. . .. as a student, I really care about like, how much I would pay for a visit in a hospital sometime.” (Fourth year graduate student)

Horsfall-2021

cost of software or hardware (3/33; 9%).

Jackson-2017

Finally, participants discussed the technical and funding requirements of the eHealth intervention to ascertain the feasibility of the project. Participants agreed that developmental costs of the eHealth intervention may be signiﬁcant, but cost savings of the intervention might be more substantial. eHealth was therefore unanimously considered to be a worthwhile investment of public healthcare dollars.

Jacobs-2014

“It always seems as though there is a financial barrier!”

Klarenbeek-2021

Other potential obstacles for uptake were implementing an extra clinical system on top of the EMR and high purchase costs

Lennox-Chhugani-2021

Save money for the service

Melo-2020

Some believed that it is sustainable because it is not necessary to create a new “digital health system” or “health industry 4.0”. Rather, existing health systems need to adapt to an increasingly digital context. These interviewees believed that applying these technologies would reduce the costs of health systems rather than increase them, provided that the selection and introduction of technologies is planned to be sustainable. However, other participants believed that it is not sustainable because the acquisition and maintenance of new technologies require strong investments that are not compatible with the levels of expenditure and waste identified for health,

Patel-2018-additional file

GP: if I was running my own practice I'd pay for this software even if I was charged to use it and I'd use it often

Pope-2017

if you can handle this with call-handlers and just a few nurses, adjust that skill-mix, you could save a shed load of money (Interview, key stakeholder).

The CDSS allowed cheap, non-clinical staff to substitute expensive clinically trained staff

The technology simultaneously proposed to reduce costs, reduce pressures on clinical services, encourage self-care, and even save lives. There were occasional dissenters who questioned the value of this substitution, but even they accepted the labour substitution and cost-saving premise of the system:

Call handler: But my understanding of it was, I thought the government have brought it in, so we don’t have to pay as much to the nurses, we could pay call handling rates, so it saves money for the government by not paying the nurses, but the bit of money you’ve saved, like you’ve just said [overtalking] […]it’s costing more, definitely […].

Call centre manager: So then they decided to cut costs and put nurses on, and the nurses generated a lot of appointments, and a lot of visits, that meant you needed more doctors. [Laughter]. And now, they’ve like, split the cake again, so now it’s just bigger than ever (Focus group, NHS 111).

Sun-2019

This group of stakeholders point out that treatments carried out with the support of Watson entail an expensive fee to be covered by the patients: “The price in our hospital is 2500 RMB for one appointment with Watson. They [patients] think it is too expensive” [1HP05]. Furthermore, the adoption of IBM Watson is also costly for the hospital management, a cost that is not matched by increased profits, as hoped: “At the beginning [of introducing IBM Watson in the hospital], we wanted Watson to bring profits. But so far, we did not see any profits from Watson” [1HP03].

Wickstrom-2020

Additionally included in this subcategory were factors such as participants’ engagement in doing good for society and in saving taxpayers’ money by becoming more efficient in wound management
