# Supplementary material for: Stakeholder Perspectives of Clinical Artificial Intelligence Implementation: Systematic Review of Qualitative Evidence
Source: J Med Internet Res. 2023 Jan 10;25:e39742. doi: 10.2196/39742 (PMC9875023; doi:10.2196/39742)
Supplement: Multimedia Appendix 3 [file jmir_v25i1e39742_app3.zip › 3. Value proposition/3b. Demand-side value/3b.4 Impact on outcomes for patients.docx]

**Name:** 3b.4 Impact on outcomes for patients

Adams-2020

Participants expressed interest in the potential of imaging and AI to result in earlier detection of ﬁndings such as malignancy and desired expanded screening programs.

Andrews-2017

participants described how they believed predicting future conditions could reduce the burden of treating people at later stages of illness.

P1: I think the idea of picking it up earlier though, probably means that it would be easier to manage

P4: I think it’s ideal because you catch these people before they tumble down into a downward spiral

P3: if we can pick that up in the very early days, we can really, it might only be a bit of psychoeducation [..] I think there’s massive health gains and savings to be made, if we can start routinely

30

screening and signposting people to the right help at the right time.

Benda-2020

Obviously, the nirvana or the Holy Grail is better patient outcomes .... If the patients are doing better ... that is ... a good metric. Obviously, we would like to reduce cost. – OPS02 [Facilitator]

Bourla-2018

Impact of pre-emptive antipsychotic treatment

Catho-2020

Several physicians perceived CDSSs as innovative solutions that can improve many aspects of their professional activities. The assumption that CDSSs can reduce errors and facilitate access to up-to-date information was indicated as a potential beneﬁt of CDSSs

Clyne-2016

Second, the majority of GPs (8/10) reported that patients were overall “very receptive” (GP16, intervention practice) to the review process, making the consultation a positive and rewarding encounter from the GP perspective:

“I actually think that this study has made me review my patients more closely, so I think it’s good for me personally, which means it’s good for my patients in the end.” (GP23, intervention practice).

Connell-2019

This in turn expedited rapid intervention for deteriorating patients, wherever they were in the hospital: The speed at which it happened was impressive. [...] I happened to be in A&E [Emergency Department] and got the alert of someone with severe kidney injury. [...] The patient was admitted to [...] a specialist renal ward [...] within 2 or 3 hours, which I don’t think would have happened without the app. [...] I think it streamlines care and speeds up the time in which they get a specialist renal review. [Respondent 9: Nephrology team] I personally have noticed [...] patients who have flagged up on the app that the clinical management has been poor up to that point. When we get involved, or the renal team get involved, that management changes [...] It has definitely saved people’s lives. [Respondent 14: PARRT] Being able to access all the bloods for the patients in the hospital and to be able to be alerted to the sick ones and already know about them before we usually do... Sometimes you know about them before the crash bleep comes through. You turn up and you think, “That was actually the alert I was coming to see.” [Respondent 10: PARRT]

Respondents in both teams pointed out that the AKI algorithm identified deteriorating patients at an earlier stage than was possible through other means: It’s a good thing from the point of view that I know there are patients that are potentially sick out there. [...] You could have an AKI and look relatively well initially. But [...] nobody would have known about those patients. [Respondent 8: PARRT]

Others emphasized the benefits of early recognition for patient health and in terms of reducing the complexity of required interventions:

I think it does a good job. We pick up patients that would maybe sit for another day or so before we pick them up. It’s certainly beneficial. It is more work, but we might be saving ourselves work in a couple of days, we might have to do more stuff to catch up. [Respondent 15: PARRT]

Flynn-2015

potential to interrupt clinical flow, and ultimately delay decision-making and treatment.

Gillan-2018

A common perception of AI was that it would improve quality of care. Participants mentioned efficiency, availability of new and accessible data, value to clinical decision-making and potential advances in facilitating higher precision and complexity of care

Grau-2019

E-STOPS use appeared to be linked to the belief that an intervention requiring minimal time or effort that can improve patients’ health was well worth doing and “certainly made it a lot easier to do the right thing”

Horsfall-2021

AI was acknowledged to feasibly predict and improve outcomes (5/33; 15%). Regarding hospital admission and inpatient management, AI was considered to beneﬁt patient care (6/33; 18%), support standardization of care by potentially reducing human error (5/33; 15%), and reduce, augment, and enhance workload (5/33; 15%). For example, AI enables prompt handover and information sharing for postoperative patient management, as well as organization of post discharge follow-up visits. Lastly, responders highlighted the vital role of AI in education and research (7/33; 21%), such as virtual reality~based neuroanatomy teaching platforms for medical students and surgical trainees, AI-guided robotic surgical training, and radiogenomics algorithms for better understanding brain neurophysiology.

Jackson-2017

Participants agreed they would support the proposed decision support tool if it were ensured that every patient would be provided with ‘meaningful clinical care’, equivalent or improved quality of care compared to patients’ existing care. Despite some participants voicing a degree of concern about the adoption of eHealth to aid disease management, the overarching consensus was that the introduction of eHealth in IBD was timely and necessary.

Johansson-Pajala-2019

There must be a purpose for the CDSS; that is, the use has to yield results. The quality reports generated need to be thoroughly reviewed to make improvements to the medication process. Moreover, the adjustments made should somehow benefit the patients. Some of the RNs reported that the use of narcotic drugs had decreased after implementation of the CDSS and that they could identify inappropriate drugs and drug combinations more easily, which they considered beneficial for the patients. Improvement in working routines was another benefit, including easier drug review procedures and time savings. Some of the RNs reported that the greatest benefit was reduced time compared to previous routines used for drug reviews

Some of the RNs emphasized the fact that the motivation for using the CDSS is the desire to do what is best for the patients, so that their treatments will be as safe as possible. Therefore, all available tools should be used to accomplish this

Joshi-2020

Institutions describe turning to prediction tools in order to “enable earlier intervention”, be able to “follow trajectories”, and improve how sepsis can be “identified in data-driven ways”, given how “clinically complex it is to define”. Multiple institutions described being specifically motivated by sepsis outcomes that were inferior to other regional hospitals.

Jutzi-2020

Participants expected that by integrating AI algorithms into skin cancer diagnostics, waiting time due to the requirement for tissue analysis by the pathologist might be reduced. Many participants also hoped that AI would lead to earlier detection of skin cancers, maybe even at the precursor stage, thereby decreasing the required therapeutic intensity for those lesions. Moreover, they stated that the use of AI might lead to more reliable and less subjective diagnoses, which might lead to fewer unnecessary biopsies and less overlooked malignant lesions. As AI oﬀers the possibility of evaluating large amounts of data in the context of diagnosis, its use might increase quality and ensure objectivity of skin cancer diagnostics. AI algorithms can be trained with larger amounts of image data than even an experienced dermatologist can assess, which may increase the accuracy of the algorithm relative to physicians.

Lai-2020

Because giving the best care to their patients emerged in the interviews as the primary goal of physicians, they were not opposed to change and were often ready to reconsider their role, as long as it remains central. They believe that AI should not become a “consumer good” that health professionals would not need. During the interviews, some radiologists complained about the fact that, too often in radiology, there is a tendency to focus on the innovative aspect of a tool and not its utility (what does it bring to the patient?). For the healthcare professionals concerned (in particular, radiologists), the primary interest of physicians in AI is not just a pointless desire but comes from the fact that, today, the ability of physicians to establish diagnoses is made complicated by the massive flow of data. Thus, as they said, they need tools to analyze and classify such data.

Lennox-Chhugani-2021

Greater safety (n=139).

Morgenstern-2021

In addition to making use of novel sources of data, participants pointed out that AI could allow us to perform disease surveillance in a more timely and ongoing fashion.

… looking at things like mortality data. We’ve had a very significant lag. It gets coded, it gets cleaned, it gets deposited at the provincial or national level. The data can be a year or two out of date. […]

Experts also thought that AI’s ability to better leverage real-time insights from big data may facilitate nearly instantaneous design and enactment of preventive interventions, creating a much nimbler loop of learning and action.

I think [AI’s] also going to enable us to design and implement interventions in real-time. So, to be able to do like […] internet companies[, who] will do A/ B testing on the color of that button that you got right there and see which one people are more responsive to. We can start to think about [similar real-time interventions] in public health. Uh, can we go into supermarkets and change the way in which signs around vegetables are presented, and monitor cash register data in real-time. So, you can design health promotion interventions in real time.

There is also a concern that AI-based errors could be

even more damaging than those of poorly performing human public health practitioners or doctors.

… if it were just one doctor making a mistake, then that is fine, […] I mean that’s not fine, but at least only one patient is harmed. But if an AI makes a mistake, then potentially tens of thousands of patients will […] be harmed. [Participant ID # 13].

Nelson-2020

Patients associated increased diagnostic speed with early skin cancer detection and life saving potential

Patients perceived more accurate diagnosis (33 [69%]) as the greatest strength of AI compared with human skin cancer screening. This perception was based on the ability of AI to draw on more data or experience than humans, to learn and evolve, and to share data. One patient noted that AI “has a huge database of what diagnosis A is supposed to look like as opposed to a human who only has their own life experiences.”

At the same time, patients perceived less accurate diagnosis (41 [85%]) as the greatest weakness of AI. This perception was based on the potential for false-negatives, false-positives, inaccurate or limited training set, lack of context, lack of physical examination, and operator dependence.“ Examining a photograph that you get with your phone invariable light, ”commented one patient, “is not a substitute for [an] in-person exam.”

Orchard-2014

Interestingly, despite the automated algorithm for predicting AF, GPs tended to rely on their own interpretation and were often able to see other detail in the trace. GPs noted that possible uses of the iECG were broader than AF screening, and a negative result (for AF) provided reassurance. • ‘It’s actually helped us to pick up other people with other minor abnormalities’ (GP1)

Park-2020

Students remarked about “AI programs that have learned to interpret chest radiographs with an accuracy rate at or better than current radiologists”

Comments such as “supplement our knowledge and abilities,” “assist with evaluating radiology images,” and “help radiologists” are certainly hopeful, although understandably lacking in speciﬁc details of how AI might be utilized in the future

Patel-2018-additional file

GP: seeing their cholesterol going down and their kidney functions improving, you know, that’s the real, yeah, when those results come through that makes me happy

Effort PM: I’m going to be running these lists and I know who’s going to be looking at these people and cleaning that data and it is a really, I mean it’s actually quite exciting when you do it. And I actually quite like doing that and I think if at the end of the day you’re helping that patient you know, that’s the best thing about it

Petkus-2020-supplementary file

IF half the population used an instrument that had a 95% sensitive, but very non-specific threshold for finding their melanoma we would be challenged to advise them against using it but also in meeting the demand for consultations to assess those alerted due to the low specificity. The risk of these diagnostic scenarios means that the value of an approved range of Apps and AI for public and institutions may be needed to help avert chaos. But it will carry its own challenges and costs.”

Porter-2018

Data collected before CCDS implementation suggested that paramedics who volunteered for this study largely felt positive about the new CCDS assessment referral pathway as an opportunity to improve care for older patients who had fallen. I think in general that paramedics, if it’s going to be beneficial for both the public and for the paramedics, they are quite open to change and they are quite eager for anything that will improve our practice … a standard approach is really needed rather than individual approaches. (Pre S1 FG2)

Torenholt-2021

He argued that using PRO and algorithms was a necessary disruption of established but costly and inefficient practices and directed a mistrust towards the physicians, who believed that they were effective at finding recurrences, but who – according to scientific studies – did not have a great success rate in spotting recurrences during clinical check-ups.

Van de velde-2018

After a while you will no longer give attention to the information that you have read several times before. This includes the risk that you do not notice that new information is available. [GP, Belgium]

Wickstrom-2020

The participants experienced that the reason and power to become engaged in the DDSS came from their professional commitment to do good for their patients, as the patients were the core value for doing anything

Nevertheless, in the first place, engagement was described as increasing due to the professional commitment to make things better for the individual patient and to make this patient group and its medical need more visible: I’m not providing care to some financial system or a budget or something like that—it’s supposed to benefit an individual person who is ill. [Participant 8]
