# Supplementary material for: Stakeholder Perspectives of Clinical Artificial Intelligence Implementation: Systematic Review of Qualitative Evidence
Source: J Med Internet Res. 2023 Jan 10;25:e39742. doi: 10.2196/39742 (PMC9875023; doi:10.2196/39742)
Supplement: Multimedia Appendix 3 [file jmir_v25i1e39742_app3.zip › 3. Value proposition/3b. Demand-side value/3b.5 Educating and prompting clinicians.docx]

**Name:** 3b.5 Educating and prompting clinicians

Abejirinde-2018

Some women also believed the device improved the knowledge of health workers and made them pay closer attention to specific aspects of the woman’s health

I think the midwife has more knowledge). Because it is through those things that she used to get the information about my condition. If not, (the results I got from) outside, she could have recorded it and give me the card to go away, but because of the machine she has took her time to also take me through this procedure to know whether it is really true that I have this disease and …she is sure that what they brought outside is comparing to her machine.”

Alagiakrishnan-2016

the SMART system improved drug management by stimulating physicians to

[...] think about the drugs before prescribing and take some time to review drugs that patients are already on. This extra step adds to patient safety.

Positive CDS perceptions included the value of reminders to reduce or discontinue drugs in association with CG GFR alerts.

Ash-2020

Some non provider interviewees noted that they had never considered asking patients about their jobs. One said the diabetes KR summary appealed to her “because I’ve worked with diabetics so long, and I never thought about their occupation.”

Beede-2020

The ﬁrst was using the system as a learning opportunity—improving their ability to make accurate DR assessments themselves. In their typical practice, nurses enjoyed fundus photo reading as an educational experience and an opportunity to apply their training.

I like to study and learn things—and this is where you learn, the way you become more knowledgeable. -P4 In the past, I got some things wrong. I made referrals when I shouldn’t have. The doctor told me it was something other than DR, and I learned from this. Now my readings are better. -P6 I went to training to learn how to read images. I’m very interested in it. I asked for images from the ophthalmologist—he sent them to me, I read them, and then I asked him to check my work. I got 850/1000 right... right now no one can replace me. - P11

Cai-2019

Pathologists felt this feedback would be valuable particularly because they currently lack formal, personalized feedback on subjective thresholds in current practice; they explained that standardized exams tend to only test classic scenarios rather than grey area cases, and that most feedback is received through inductive observation when getting a second opinion

Catho-2020

“It has to make sense for the patient, that’s really what matters to me”.

“A help to rectify shortcomings that can be present at any given time and that can be corrected by the computer support”

suggest them what to do, this may negatively impact their training, critical thinking and clinical judgment.

“I support the local antibiotic therapy guidelines but if we misuse them or use them too much, we forget to think”

Chow-2015

‘I already know what I want to use but I want to cross-reference to see what they recommend, and when it’s different, I’ll think about why it’s different’ [J5]

Clyne-2016

Conducting the reviews with the patients was described positively by all GPs:

“Oh yeah, it was very good, yeah, because actually, because they were coming in you were able to look at everything properly and they were coming in a structured review … just to give you a time to review the whole situation you know, in regards to all of their prescribing. It was very useful, yeah.” (GP18, intervention practice).

Connell-2019

Some respondents in this team also pointed out that early identification could not necessarily be aligned to early intervention because of a lack of knowledge with respect to appropriate management:

The patients you definitely need to see are the patients that have acute renal failure with a creatinine of 300 or 400 [µmol/L] that and it’s going up - patients you’d normally want to see [...] The other patients [...] that have a rising creatinine, but the creatinine is not very high - it doesn’t mean that they don’t need to be seen necessarily. We are not trained as doctors to look after those sorts of patients. [Respondent 1: Nephrology team]

Thus, the shift toward earlier detection highlights the need to consider the resources required to manage both early and late disease and the training needed to enable clinicians to effectively intervene at an earlier stage.

Dikomitis-2015

The most signiﬁcant function of the eRATs, ﬂagged up by the vast majority of GPs, is that the tools raised awareness about potential cancer symptoms and both reminded and alerted GPs to risks:

‘It’s just electronic highlight, makes you think even if you immediately dismiss it, at least that millisecond you’ve thought about it, and I think that is going to be useful at some point, but for how many people I don’t know’.

Flint-2019

The end users that participated in the focus group felt that the tool was useful as an educational resource, and had safety and quality improvement beneﬁts

Flynn-2015

One stroke physician used COMPASS as a clinical training aid with an emergency medicine physician to show the likely outcomes if a patient had arrived within the time window for thrombolysis

Gillan-2018

MP02 suggested that AI would provide ‘a greater opportunity for some of us to catch weird things in a plan […] the computer is helping to draw the attention to some relevant features’.

Horsfall-2021

concerns about overreliance on AI systems (5/33; 15%)

loss of surgical skill,

Johansson-Pajala-2017

Many of the RNs expressed that they received further training in the area of pharmacology. They learned more about drug effects and ADEs. The obtained knowledge increased the RNs’ pharmacovigilant awareness in the sense that they became more attentive to potential risks with drug treatments for older persons, in general, as well as possible associations between drugs and various symptoms.

‘You learn what things the system refers to, interactions or potential risks and you will then get everyday knowledge, like when you get a new patient and see all these drugs and think this we have to look into’

Jutzi-2020

They hoped that physicians would even be able to learn from the AI-based systems. In their opinion, the direct comparison might also motivate specialists to strive continuously to improve their own performance.

Keogh-2019

Clinicians also felt that iPrevent would provide support for PCPs to advise women about BC risk

Clinician: Do we currently speak to moderate risk about drugs? Clinician: No, we don't. Clinician: No, we haven't tended to. We should be, according to guidelines. Clinician: Yes I suppose it's formalizing and making sure you're less likely to miss something for a particular patient, if you were. I suppose we don't normally talk about medical prevention with like moderate risk, so I'm just saying we formalize. You're less likely to miss a step if…

Consumer: But it helps them [PCPs] focus too I think rather than fafﬁng around like you said. Consumer: And they would know which bits to focus on too. They don't necessarily know which bit to focus on, why you're there. Consumer: They can't faff if you walk in with this. Consumer: This gives them the focus

Knoble-2015

The HCWs were also generally positive about the e-algo application. They stated that it improved their decision making and enabled them to view a full differential diagnosis for a speciﬁc complaint. They saw the tablet having a dual purpose: learning as well as diagnosis. One HCW said that the application made it unnecessary for him to refer to books when dealing with patients.

The algorithm questions helped refresh their memory of things they had missed in their history and examination. They also valued the disease management section which standardized and helped them focus their treatment rather than their traditional broad treatment approach. Finally, the “Warning Signs” list at the beginning of each chief complaint made them think about these issues in advance.

Liberati-2015

["The patients are many and pluri-pathological and the attention at the end of the day it is what it is. We doctors we have limits of memory, attention, […]. A support that accompanies us to apply caring for the evidence can be more than useful ". (Doctor internist, setting A) ]

Liberati-2017

Some participants emphasize the potential danger of CDSSs disseminating poor or not updated evidence. They are concerned that young physicians may become accustomed to following CDSSs’ recommendations without questioning them—an eventuality that would be particularly problematic in the event of outdated evidence. This issue is sometimes referred to as the “prompter effect.”

It’s this sense of false security given by the CDSS… Thinking “Oh well, the computer will tell me the answer”… And people sort of switch their brains off. […] Especially young physicians […]. CDSSs have an incredible potential, but they do expose us to unexpected risks. (Manager, setting C1)

Marcolini-2021

The structured clinical evaluation was perceived as an advantage, as exemplified below:

The questions we always ask (using the software). Are you feeling shortness of breath? Do you wake up out of breath? Sometimes in a rush, we forget (to ask those questions to the patient). But we always ask them (when using the software). It is as if you take the query and say: this is indispensable for you to ask to the patient. You always have to remember to ask about it. So, for me it's great. I will always remember to give counselling, for example, when the patient is a smoker, at the end there is always the ‘Did you talk about smoking?’ reminder. So there you will remember to tell the patient “Look, you have to stop smoking”. For me, this is very cool. [Female, 31-year-old physician, 4.5 years’ experience working as a primary care physician]

it. They considered the alerts and recommendations very useful for patient care, and reported that it influenced their patient care plan. We usually don’t have time to calculate cardiovascular risk and kidney function. The software helps a lot. [Male, 39-year-old physician, 6 years’ experience working as a primary care physician] When attending a patient with high cardiovascular risk, who was taking 40 milligrams of simvastatin, and also amlodipine for hypertension, I received an alert to adjust the dosage of simvastatin to 20 milligrams, as amlodipine may increase the serum concentration of simvastatin and thus increase the risk of adverse effects. I had no idea, I learnt with the alert and changed patient prescription. [Female, 31-year-old physician, 4.5 years’ experience working as a primary care physician]

McDermott-2014

The use of the prompts as a reminder and reference tool was often reported as being used outside of the consultation time when a GP would have more time to read through these details if required.

"I think that it is a useful reminder, it doesn’t take long before having seen them and you've refreshed your memory of the NICE guidelines" (P04)

Moullet-2020

Three fellows and all senior physicians explained that patient energy and protein requirements were discussed during morning medical rounds. Some physicians mentioned they were discussed daily, and others not on a daily basis. “What has been changed is having to enter the calorie and protein goals. Now we realise what goals we are aiming for.” [JP9] “It is true that now the night team has to enter the data on MetaVision and during the morning medical rounds, we can see where we are in terms of total intake, if there has been a change compared to the previous day and we can adapt the new nutritional prescription.” [F11] “Every day during the morning medical round, we read the order sheet and at some point we come to the nutrition goals, whether they have been met or not. I look at them to see if they seem to be adequate and then we discuss the nutrition strategy.” [SP3].

The majority of physicians reported that the introduction of the

tools made them more attentive to nutrition: “Everyone is more attentive to nutrition and during the morning medical round, we look at this point.” [JP7] “We used to have children who fasted for several days … that has not been the case anymore, I do not see it anymore and I feel like there is more attention to nutrition. Before, it was heartlung, if it was ok, we used to look at digestion and now it is becoming something to be systematically controlled.” [F9] “I have become more rigorous, I pay more attention to nutrition. I am even more interested in nutrition than before.” [SP2]

Nicks-2016

NHVs also noted that using this program broadened their awareness of injury risks, bringing specific injury risks not typically covered to the forefront of the education provided (Table 3, Item B) and helping NHVs become more aware of safety hazards in clients’ homes (Table 3, Item C).

In addition, many NHVs felt that the organization already used too many assessment tools with clients, and the focus on injury prevention would take away from discussions of other areas.

Orchard-2014

GPs overwhelmingly liked the portability and instantaneous results of the iECG, and that it acts as a prompt.

‘I think it’s fantastic. It helps us to concentrate for a moment on cardiovascular health’ (GP1)

‘It’s the awareness you need to look for AF in the GP setting…an opportunistic check’ (GP2)

Orchard-2019

“It made me improve my ‘patter’ about AF. I got the statistics right in my head about risks per annum per patient and what we were doing to try and get them down, which helped my medicine.” (GP 2, Practice A).

“When I do a study and I’m focused on a particular topic, it does help me to be more diligent and vigilant on that topic.” (GP 1, Practice C).

“[Screening] is adding to our conversation about heart health”. (Nurse, Practice C).

Patel-2018-additional file

Both GP and PM found the study to be valuable to the practice in different aspects; GP got involved initially to improve CVD management/knowledge

GP: …highlighted the issues that’s important in treating these patients, and so it’s becomes second nature now to know what to do and how to do it.

GP: the routine of checking for the microalbumin is something I didn’t do before, and now I realise its benefits.

PM: It helps our nurse, so then we ah look, this person hasn’t had such and such for a while wo when you’re doing a chronic disease management say for cardiovascular or diabetes or anything like that, that all shows up as well. And so then we can then say to Dr. XXX (main GP) well look this hasn’t been done either because he might not have seen that patient in that last six months….so it’s like feedback from the nurse and also the doctor as well.

Main GP: was using it [HT] as a risk measurement tool and as a patient teaching tool that people would, we would, a registrar would say “Gee I’ve just seen somebody who’s come in, who’s new, and have got a cholesterol of this and a blood pressure of this, and their sugar’s a bit high but they claim they’re not diabetic. What do I do?” and instantly we’d use it to say “Okay, let’s have a look at the risk. Let’s see what things we can modify. What can we do straightaway? What can we do down the track? What’s what? What can be, which order do you do it in” and so on. And at times what’s the evidence for it? Use it to teach wider range

Philips-2015

two comments referred to more pharmacokinetic education for doctors

Pope-2017

Along with the formal training necessary to do the job, we found that the call handlers undertook a variety of formal and informal training. This included such things as taking evening classes to learn anatomy and participating in ad hoc lunchtime “seminars” from paramedics to learn about symptom presentation and disease management. Surrounding all of this, there was a continual exchange of knowledge and experience in everyday interactions with other call handlers in-between calls, which call handlers used to learn how to use the technology and manage the calls. It seemed that introducing this digital technology entailed more tasks and required a more intellectual and organisational effort. The work was subjectively experienced as intense and objectively appeared to entail considerable effort.

Porter-2018

It – it asks the question, you know, that: Is an EC- an ECG required? And it, again, you know, those sorts of things that maybe three or four o’clock in the morning, when – when you’re – when you’re not at your best is – it’s always nice to receive that kind of prompt at times. (Mid S2 08)

it’s made me think more about making – about – about referring patients. (Mid S2 08)

Hmm, it got me thinking a little bit more about how we’re treating falls. (End S1 07)

Rapoport-2020

Similarly, a specialist physician used the tool to teach family practice residents and found it effective in encouraging students to think more holistically about driving assessments.

Roebroek-2020

It was often mentioned that TREAT prevents you from missing certain issues during consultations. This opens up the opportunity to discuss these issues with patients, as this respondent revealed:

“Of course, that’s the beauty of this system. TREAT suggests things that you otherwise might have forgotten or wouldn’t have thought of. Sometimes it can be used to engage in conversation. For instance saying something like: “According to the guidelines, you would have to start with an antidepressant. What do you think? Oh, you don’t want another pill? Okay.” [C6]

Soling-2020

like to use it [digital tool] and see also a lot of sense in it, because I also learn again, refresh again, knowledge that is perhaps still present somewhere in the back of my mind, but to update this again, but I find this information very good.[ … ] It makes my work as a doctor much easier when prescribing, so I think that makes a lot of sense.

I now find myself with my patients, well, coming to their routine visits, simply perceiving these risks more intensely and then changing it, yes, with the other patients as well, if I consider it initiated. And I found that, for example, quite good. [FG3, GP_DD, p.9]

Soling-2020-supplementary file

“And the advantage of this system, or this program in general, is that I have reviewed the patients who are now enrolled […] that I looked again on the medication plan and see, does he still take everything that I have there now

Tsang-2021-Supplementary file

“I think there’s also an educational thing because I think a lot of the time, we wouldn’t check some things when we probably should be, as the system suggests.” [GP1, doctor]

• “The whole idea is you want everyone thinking like this, don’t you, and not everyone relates things together. Whether you show it in a format that makes them think…it’s almost like you’re coaching them into better prescribing. [P6, pharmacist]

• “AKI was not something that was on my radar as something that required coding or required follow-up, even if they weren’t on anti-hypertensives. So I’ve learnt to do that.” [P7, pharmacist]

• “I think it’s got real potential and for people like technicians and pre-regs and all the others… it’s quite a useful tool and learning aid” [P8, pharmacist]

• “It has given me a different learning avenue as well. So, some of the things that it’s asking me to look into, it’s given me ways to look at evidence to understand why and what I should be doing. So, it’s, kind of, underpinning some of my trainee work as well, which is quite useful to be honest.” [N10, Nurse]

Urquhart-2018

the introduction of primary care-based identification would necessitate enhanced training among primary care providers, as emphasised by one provider:

‘I think we require a lot more [training] than we’re getting … the clinical implications will be way more powerful and appropriate. People just aren’t trained up to do this.’ (Dual role, FG5)

Van de velde-2018

It is obvious that all GPs do not know all treatment options to all diseases, so CDS could help them. [Patient, Finland]

Wang-2018-Tables

When I see a patient … there may be aspects that I wouldn’t have thought about that I should have thought about that wasn’t there. So it is a very good tool that has everything on it.

I think it (CARATV2.0) is good because it would make you also think about things that you might not think about when prescribing these medications that you might forget about the drug interactions and things like that. (N06)

Wells-2014

prompted them to do all the necessary checks

honed their skills with this patient group

Wickstrom-2020

An interest in seeking new knowledge produced and nourished the personal liaison of engagement:

I saw it as a huge opportunity, both for myself and for the workplace, to ... to, like, develop in this, both for my own part, like, for myself, just because I think it’s ... I think it’s fun to gain new knowledge and to get better at things and so on. [Participant 11]
