# Supplementary material for: Stakeholder Perspectives of Clinical Artificial Intelligence Implementation: Systematic Review of Qualitative Evidence
Source: J Med Internet Res. 2023 Jan 10;25:e39742. doi: 10.2196/39742 (PMC9875023; doi:10.2196/39742)
Supplement: Multimedia Appendix 3 [file jmir_v25i1e39742_app3.zip › 3. Value proposition/3b. Demand-side value/3b.6 Consistency and authority of care.docx]

**Name:** 3b.6 Consistency and authority of care

Abejirinde-2018

“Using the box makes me actually believe that it is indeed true, because with the box she is telling me what she has seen”

Many health workers felt that women’s compliance to referral and counselling recommendations happened because the machine, which was seen as a more knowledgeable or accurate medium, was ‘demanding’ it.

Alagiakrishnan-2016

uncertainty was often diplomatically resolved, respondents observed that the management of CDS-related disagreement was inconsistent.

Ash-2020

Several interviewees involved with quality improvement or management

thought that the proposed CDS would be useful for standardizing clinical practice.

Catho-2020

• GE_05 (M, resident): “They allow me to be eﬃcient and above all to be a little more conﬁdent about the choice”

Chow-2015

Colleagues from other hospitals have also requested hard copies of ARUSC’s recommendations that were unique to the hospital.

When we write our plans, we’d say “suggested by ARUSC”

Chrimes-2014

Confusion seemed to stem from a lack of familiarity with shared goal setting and its CDS components. For example one provider stated that “I think the Hemoglobin A1c is helpful. But I have never put in my notes the number of sweetened drinks per day like this (subject’s mouse points at 5 on the screen), nor number of steps per day (subject’s mouse points at 3000 on the screen). ‘Get off one stop early on public transport’, is that a pre-populated option

Clyne-2016

GPs from both intervention and control groups expressed a desire to learn and a willingness to change their prescribing practices:

“When you are a GP you get practices and you get bad habits, and you get good habits, and sometimes you are too busy to change your habits until it is pointed out so, anything like this is a good thing.” (GP19, control practice).

Connell-2019

In addition, the visualization of each other’s triage decisions within the app (a feature specifically requested by users) revealed the hitherto unrecognized variations that exist in professional judgements. Respondents in both teams raised the fact that knowledge of others’ decisions sometimes confused rather than clarified the clinical decision-making processes of colleagues:

I was quite surprised about how other people triaged initially. I felt we’d be much more similar in our thinking, because when we talk about other things we do think similarly about other stuff. [...] I felt like - probably naively - that everyone would do what I did. And they didn’t at all. [Respondent 11: PARRT]

Flynn-2015

“emphasises the importance of not only documenting a very high quality conversation but also puts our focus of mind that this is an important piece of managing the patient in that very difficult time” (SP 3).

“it then becomes part of the record which I think will stand up better in court” (SP 4).

Guenter-2019

Another perceived value of CPMs was their ability to increase prognostic authority by reducing ambiguity – i.e., the presence of conﬂicting opinions about prognosis, especially among physicians:

Hospitalist 1: [A tool] would be particularly helpful in situations where I didn’t agree with the patient and or the patient didn’t know what they wanted or a subspecialist didn’t want to do the procedure...where again a big decision was overdue and it might help push us in one direction or the other....Or I thought someone looked just barely alive and [it] told me they had a good chance of survival I’d think – what am I missing here? Maybe I need to scale back my assessment.

Geriatrician 4: The surgeon says he’s not a surgical candidate and if you’re able to say actually he has a really good survival... I mean I don’t think would be swayed necessarily by this alone but it would certainly perhaps fortify one’s own resolve in presenting it again as something that should be considered.

Cardiologist 1: I don’t want to do it, ‘cause they don’t look like they’re a great candidate, but I’ll do it because they meet the criteria. And then you say but wait, this says they have a 90% chance of being dead in 6 months, I’d say, ‘Praise the lord I’m out of here’...

The point was also made that the authority CPMs provide may even mitigate medicolegal risk if prognostic estimates and any resulting decisions proved incorrect:

Cardiologist 1: I was thinking [that prognostic tool data could be helpful] where the level of litigiousness is higher and the family of that 89-year old just very well might sue you if they drop dead at the age of 90 and you didn’t put in an implantable deﬁbrillator. If you could just say: ‘this says that they didn’t qualify,’ then I could see that has having some advantage.

Oncologist 3:

... the model would help me have an evidence base to be able to say to the patient, look, this reliable model says your life expectancy, factoring out the cancer, is already very limited... And it would help them understand the situation and make a treatment decision about the cancer...this model would give me a way to have something to point to help make that case. It would help me help the patient see the situation more clearly; it would help persuade them...it would push them to make a decision.

Importantly, the clinical circumstances raised by each of these physicians were one of low prognostic uncertainty – where future outcomes and the optimal course of action appeared clear

Henshall-2019

All psychiatrists commented that the DST would help to increase their awareness and consideration of a wide scope of medications. They felt this was necessary as they admitted that they generally prescribed a limited selection of drugs, based on anecdotal evidence and simplistic cost-effectiveness considerations. Ho

Johansson-Pajala-2017

Within this structural aspect many of the RNs stated that the CDSS provided them with a structure for how to conduct a medication review; a standardisation which was perceived as beneﬁcial as it implied that all RNs would perform the review in the same way. The RNs’ clinical practice also became more explicit since their activities were clearly documented.

‘If you imagine that there are many cooks in the broth here, then it’s good if we get a standardised way of looking at different things. As individuals we have different approaches ... but you get a standardisation that I think is good’

Keogh-2019

They used a range of different tools to estimate a women's risk: IBIS model [20]; BRCAPRO [27]; BOADICEA [21]; or Cancer Australia guidelines [28] (se

Clinicians felt that iPrevent would increase the likelihood of consistent practice across clinics, and reduce the chance of inaccurate advice being offered to women. In

Certainly, if breast cancer risk assessment moves more away from an FCC and more into the [PCP] realm, then you need a way to make it as consistent as possible. So that high-risk women are getting called high-risk, and moderate, moderate, et cetera.

Klarenbeek-2021

A structured way of working was seen as beneﬁcial for accurate functioning of the CCDSS. Components of a structed way of working include consistent use of terminology by all medical disciplines and storage of information at dedicated locations within the EMR, including retrieval of information from scanned or external documents.

Professionals suggested that if the CCDSS was able to provide easily accessible and well-structured patient data, this would enable a more to-the-point, structured, standardized and transparent MDTM discussion. Well-structured patient data was deﬁned as a clear customized patient-record consisting of discrete data ﬁelds with standardized response of parameters (e.g., age, weight, stage) that can be directly incorporated

Prioritize development and implementation of the ﬁrst component (structured overview of patient variables) because this component was expected to improve MDTM workﬂows most

Perform a usability test and validation of the prototype CCDSS in real-life setting prior to roll-out

Willingness of professionals to change current workﬂows in order to beneﬁt from CCDSS decision-making.

Lai-2020

For some physicians, AI would therefore represent a revolution in their practices and patient care, whereas for the others, it would only be a continuation of the ongoing improvements in medical practice

Lennox-Chhugani-2021

Improved reliability (n=263)

McDermott-2014

This issue was reported as being closely related to communication difficulties within practices

-Delays due to practice staff unawareness of study.

-Improvements to staff awareness needed.

providing the GP with confidence in their decision and presentation of advice not to prescribe antibiotics. GPs were happy and willing to engage with and use the prompts if they perceived them as a tool which could support their own decision to either not prescribe antibiotics or issue a delayed prescription.

"First of all they give confidence to the doctor, that there is some evidence behind the decision" (P08)

The prompts were often reported as providing GPs with assistance in persuading patients of the benefits in following the advice recommended in the guidelines. This particularly referred to persuading patients that antibiotics were not necessary for a RTI in patients who the GP perceived as being unwilling to or apprehensive about accepting this advice.

"There’s always that kind of feeling like 'oh' (they want antibiotics), but actually it’s very good because it's helpful in guiding patients"

Moullet-2020

All physicians described that they used the nutrition protocol as a main source of nutritional information. The majority explained that the nutrition protocol clariﬁed and standardised the management of nutritional support for all medical staff: “I think that now we understand better what to do, how to do it and why, we know better how to calculate what we need to give in each case.” [F13]

“Our care is even more homogenous because we follow a clear protocol.” [SP1] “The other thing that has changed, I think for junior physicians and fellows is that it is clear how much to give, it is not everyone deciding what they want. We all agree on the calories we give to intubated and sedated children.” [SP3]

Nelson-2020

more consistent diagnosis (13 [27%]), more objective diagnosis (11 [23%]), and

Orchard-2014

• ‘The reassurance aspect of it was great’ (GP3)

Pannebakker-2019

Some discussed using the melanoma eCDS for reassurance, either for themselves or for their

patient: ’. . . if someone was very worried and they scored zero then I might be able to say, “Look, this is a scoring system that’s been developed,” and it might just aid reassurance. Equally, if I was worried about someone and I wanted to explain why, I might just say, “Look, this is the scoring system, you’ve got quite a lot of points on this. It doesn’t mean it’s anything serious but it does mean we need to look into it more closely".’ (M, 40 years)

Patel-2018-additional file

GP was an advocate in calculating absolute CVD risk, and prescribing according to their risk. HT made GP aware of prescribing guidelines and gave him confidence to prescribe “early”. Further GP had professional development motives for using the audit tool and absolute risk calculations

GP: It's helped my practice individually because I can get an accurate complex summary that takes into account all these additional factors and other relevant history, all the numbers, it gives me the reassurance that I'm getting an accurate cardiovascular risk, the most accurate really that I've seen by a long way. So it gives me confidence to prescribe the medicines that I prescribe knowing that my cardiovascular risk is accurate.

Pope-2017

Its introduction was also welcomed as a chance to standardise triage practices and offer – at last – a service that could “give the same disposition to every patient who presents with the same condition” (Interview, 999 manager).

Porter-2018

I found that it wasn't making the decision for me, it was just agreeing with the decision that I'd already come to. (End S2 02)

The bottom line is I like this because I have got evidence to show that I have thought about what I am doing. (Pre S2 FG2)

Rapoport-2020

Participants’ reasons for wanting a tool

… part of what holds many of us back is we know it’s going to fracture the relationship. They’re pleading with us not to do this and it’s just so heart-wrenching. I want any tool that can help me. [MD03-FP]

… what exactly are we going to do with every single patient, every single time. So that if it’s called into question by family or patient we can say, ‘Well, no, this is the standard we’ve set. These are the guidelines we’re using …’ [NP01]

Perceived value added by the tool

I’m here on my own sometimes, so I don’t necessarily have somebody to run down the hall to … And I’m thinking of this concern about a licence. So it helped to sort of ease my concerns being somewhat solo. [NP03]

The problem we have in family medicine is we’re not a specialist and people will say, ‘You’re not a specialist’. So, it’s like your judgement is just not as important. And so this is almost like that specialist saying, ‘Yep, I agree with you’. [MD03-FP]

A widely shared perception was that the tool added value in the form of consistency and standardization of the driving assessment process in persons with MCI and mild dementia. Several participants commented on the perceived value of having something more objective than the clinician’s opinion to offer patients:

It makes it seem like it’s not just me saying ‘I think you should stop driving.’ [NP04]

Several participants pointed out that while some patients or family members experienced the tool as adding credibility or objectivity, others were mistrustful of decisions seemingly made by a computer. Some participants mentioned that the tool could be beneficial for families who had concerns about their relative’s driving capacity.

Soling-2020-supplementary file

So, they [patients] feel safer and also, I think, more confident about why they take something. Because you can explain what the tablets are really good for.” [GP7, p.4]

“And at the moment, when a project like AdAM is running, we can, of course, say, okay, we've had

it externally again. Someone looks at it again, and of course, we are a bit more on the safe side from a legal point of view. Of course, our responsibility is still to give or not to give medication at all, but we can at least say what the medication is like, what the consequences are and that interactions have been checked externally.

Tsang-2021

Users of the dashboard reported gaining third party authority, with information from the dashboard seen as a trusted source by wider practice staff and commissioning groups

“We’ve got some really good stuff out of the dashboard, where you can look at things, and go, we want you to do this, because of this, and that’s why, and it’s a high risk indicator…You can say this stuff until you’re blue in the face, and nobody cares until you go, and there’s my proof. And they go, oh.” [A4, administrator]

“It is helpful sometimes having this kind of information for teams like ourselves who have to do projects and we have to demonstrate the value of the projects that we do to the CCG” [P5, pharmacist]“I can present my case better. Because I am giving them the right relevant information… It makes sense to me what I am looking for, that I can then understand it to say to [the GPs], that this is what we need to do with my patients” [P7, pharmacist]“Especially because I am a trainee, if I am suggesting things, it just gives me that, kind of, back up. That it says this is in alignment with this standard.” [N10, nurse practitioner]

Tsang-2021-Supplementary file

• “Normally it does make your workload more directed, so I think that’s something to definitely bear in mind, that it means that you’re targeting those patients that bit more to figure out what’s going on with them.” [GP2, doctor]

• “It requires individuals to recognise what's happened and where we go from here, rather than there being any processes in the background…the system helps that” [P5, pharmacist]

• “What I found is by setting the standard you raise everybody's standards as well, so everybody else is seeing this is what is expected from the indicator and this is what needs to happen” [P6, pharmacist]

• “I’ve been recently using PINGR to direct my work: so what’s not looking good, oh let’s have a look at this.” [P9, pharmacist]

“I guess you’re sort of relying on individual sort of clinicians that if medications have been changed on a discharge summary, to organise sort of follow-up appointments themselves by sending a task to reception. I’m not sure that that would happen consistently.” [GP2, doctor]

• “Then we can kind of standardise and reduce variation a little bit…and it’s just much easier to mobilise teams” [P5, pharmacist]

• “I would be looking at restarting [medications], whether or not the discharge summary has highlighted that to us. Sometimes they do, sometimes they don't. That process isn't always 100% reliable.” [P7, pharmacist]

Urquhart-2018

As a result, participants believed end-of-life conversations need to be systematised within healthcare settings, and viewed a practice-based electronic medical record algorithm plus supports as tools that would help facilitate a more systematic and coordinated approach in primary care.

Vanhille-2018

“…benefit in the patient who you treat medically, and they say they have not improved…you can show them that they have improved.”

Wells-2014

Paramedics reported that CCDS generally supported their decision making

provided documented evidence of their patient assessment
