# Supplementary material for: Stakeholder Perspectives of Clinical Artificial Intelligence Implementation: Systematic Review of Qualitative Evidence
Source: J Med Internet Res. 2023 Jan 10;25:e39742. doi: 10.2196/39742 (PMC9875023; doi:10.2196/39742)
Supplement: Multimedia Appendix 3 [file jmir_v25i1e39742_app3.zip › 4. Adopters/4a. Staff/4a.1 Appetite and needs differ between staff groups.docx]

**Name:** 4a.1 Appetite and needs differ between staff groups

Alagiakrishnan-2016

It has the potential role [as an educational tool] depending on the comfort level of the physician and their knowledge of drug interactions, risks and side effects. I think that it is a supplemental tool for doctors and I think that their use of that tool is dependent on their level of expertise

Benda-2020

Unsurprisingly, operational personnel were most likely to discuss external and intraorganizational drivers; informatics stakeholders described elements of the hardware/software infrastructure; and end users tended to focus on people, communication, and workflow.

Chang-2017

. although the training did explain the system and practice. It would be a little rusty regarding to the operation the system if I did not use the ED triage computerized system frequently. This definitely affects the triage decisions. (P3)

Chirambo-2019

“We have welcomed this development wholeheartedly. But we don’t know if really the HSAs would use them as some of them may not be technologically competent to use these apps.” PSA-5

Top-level managerial concern with regard to the HSAs acceptance to

use the apps was remedied when the HSAs stated that they accepted the use of these mHealth decision support apps as they made their work easier.

“We have accepted using these apps, and they are making our work simpler when doing eCCM” PSA-2

Chow-2015

Junior physicians accepted ARUSC’s recommendations most of

the time, whilst senior physicians were willing to accept its recommendations if the source of infection in the patient was unknown [S1].

Junior physicians trusted the credibility of ARUSC’s recommendations and would use them as a ‘conﬁdence booster’ and to ‘cross-reference’ their antibiotic choices [J3, J5]. Senior physicians would often advise their juniors to refer to ARUSC when in doubt [S2, S5]

Junior physicians found ARUSC to be particularly useful when on-call [J3, J5].

It’s good when it’s at night and you don’t feel like thinking. . .’ [J5]

Connell-2019

Participants in both teams found alerts to be particularly valuable for patients whose lead consultant was not a physician:

The most value came from patients under [...] surgical patients, for whom the list of priorities for their clinicians are very different from what [physicians] look for when they are looking after a patient. For those [patients], getting a rapid alert about deranged renal function is very valuable. [Respondent 6: Nephrology team]

Dalton-2020

Some recommendations were not implemented as prescribers tended to confine prescription changes to those within the professional boundaries of their specialty. In particular, interviewees stated that surgical prescribers were much less likely to implement the SENATOR recommendations than medical prescribers.

It looks like instead of holistic treatment of the patient, each consultant is treating their part. [Primary Researcher 9]

Interviewees recognised that junior prescribers may be more reluctant to change patients’ medications than their more experienced colleagues. However, several participants felt that many junior prescribers have the knowledge and skills required to implement these recommendations, but lack the authority to adjust patients’ medications without consulting a more senior colleague.

…they are not in a position to change the medication. They have to discuss with the senior person, either registrar or consultant. [Primary Researcher 9]

Participants indicated that whilst prescriber experience may be influential, it was more important that the recommendations were reviewed by a ‘decision-maker’ in the prescribing team, whereby participants most commonly considered the ‘decision-maker’ to be a more senior prescriber. I think it’s helpful, or more helpful, to speak with the senior doctor, who is a decision-maker. I think the senior person on a medical team would be more likely to implement changes. [Primary Researcher 7]

It was clear from the interviews that prescribers were much less comfortable acting on recommendations that were outside their field of specialist knowledge. They have conditions that are out of my range of knowledge, and their treatment often…their treatment of one condition might collide with another condition that I’m not an expert in. [Medical Prescriber 12]

Horsfall-2021

In an operative environment, AI systems’ complexity (5/33; 15%) and surgeons’ reluctancy to change (2/33; 6%) were considered barriers to adoption.

Jacobs-2014

“Lack of understanding on the part of organization administrators that may not understand the need for or value of technology.”

Johansson-Pajala-2019

Staff continuity facilitates collaboration and implementation. From the RNs' perspective, it is important that all RNs are working toward the same goal and continuously supporting each other. Continuity also applies to their relationships with patients, so that the RNs always manage the drug reviews of patients for whom they are responsible. The RNs explained that continuity is also essential among the physicians; however, some suggested that the CDSS would be even more useful for the temporary physicians

Joshi-2020

“I think the fact that we as the clinical effectiveness team are clinicians, I think really helps.”

Jutzi-2020

If used as an assistance tool, participants expected AI to be able to reduce error rates in diagnoses especially for less experienced physicians.

Keogh-2019

Clinicians described PCPs as lacking the knowledge and expertise to assess BC risk, and as unfamiliar with online tools

Lai-2020

At the time of this study, all the interviewees agreed that radiologists would likely be the first to work with those new tools and thus be confronted with these issues.

Lee-2015

With additional probing in focus group discussions, radiologists emphasized the need for additional

training and education of referring clinicians on the importance of CDS.

Liberati-2015

[It is an exhilarating thing! Me with this system (SSDC) I might have the hope of finding the evidence right away that I need, without going to search on the internet, where I also find the non-stick pans ... is history of the electronic medical record to me not like… An electronic file in the hospital, where everyone could access ... internists, diabetologist, cardiologist, orthopedist, the operating room ... I am from a different era, the fact that it is so accessible to me upset a little. And then now the folder comes written in the patient's room. We internists don't is that we sit at the table, at the computer, to write. We visit the patient, see how he is, and write. [...] It seems to me that it is not for me, I would never want to that they imposed it on me. " (Internist, setting C)]

McDermott-2014

GPs often reported the prompts as being particularly useful for a group of staff which they described as ‘inexperienced practitioners’. GPs described inexperienced staff members as including newly qualified GPs, student doctors, locums, and nurse practitioners. The GPs interviewed in this sample reported that these ‘inexperienced staff’ may benefit from the prompts as they would be less aware of the guidelines in general, the evidence and the recommendations not to prescribe antibiotics. (However, transcripts did not provide details of the level of experience obtained by GPs within this sample).

"New colleagues or new prescribers might be needing to look at it more" (P01)

A key barrier to using the prompts was that some GPs reported not needing them as they claimed that they were already following the advice recommended in the guidelines and had their own methods and procedures for doing this. GPs in this group did not report any problem with the functions or features of the prompts specifically, but simply that they were not needed.

"I mean I don't find or look at them…because I'm usually relatively comfortable with my respiratory management shall we say, I do very few respiratory referrals etc." (P02)

Miller-2019

Most providers seemed to think a system such as this could work in the ED, in particular if staff are well trained, if the project is supported by nonphysicians, and if workflow and follow-up issues are addressed

Orchard-2014

While receptionists were reluctant, practice nurses were very confident with screening patients and explaining the process. • ‘It gave you a chance to speak with the patient and ensure they understood … Patients were very eager, actually’ (Nurse)

• ‘[the nurse] opportunistically spoke to them about the study, educated them about the importance of it and then subsequently did the tracing and ... then called me down to have a look at it, so that I found it worked really well’ (GP2)

Pannebakker-2019

also felt that it could be more useful for less experienced GPs or those who felt less confident when examining skin lesions:

’I think that’s right for those . . . certainly trainees, and maybe younger GPs and people who haven’t done a lot of dermatology, yeah.’ (M, !51 years

Patel-2018-additional file

There was lack of relational integration. GP is only staff using HT and CAT. GP has mentioned it to the PM however she is not keen on taking on any extra tasks outside her current role due to time and resource constraints.

GP set in his ways in patient care. He needs a lot of hand holding to become comfortable with new method of assessing CVD risk.

Main GP: ..my age range is against me. It was, I’m not someone who’s intuitively familiar. So I attempt to use [HT], as I say I really hate

computers…I tend not to sort of sit and say “Ooh I wonder what this will do” or “I wonder if I can find out this? I wonder if I can find out that?”….therefore if that’s my approach then it’s difficult for me to pass that on to other people [GPs]. I can’t sort of say go and do this [use HT] when I don’t usually do it [use HT].

PM: I think the younger doctors found it [HT] useful,

Petitgand-2020

Perceptions of DSS-induced errors were shared among physicians, and this led

some to develop a persistently sceptical attitude towards the usefulness of the DSS. The AI system was thus viewed as introducing a real risk into clinical practice that was capable of causing harm to patients. This is a perception that tends to increase clinician resistance to health information systems.

Porter-2018

However, it was suggested that some—including colleagues who had not volunteered for the study—might be more resistant to adapting to new technology:

I think we’ve got some crew members are very resistant to change and that’s just a natural thing. They’re just worried about anything new coming in. They think – they wouldn’t bother with it. (Pre S1 FG1)

Rapoport-2020

Other physician participants, however, did not attach the same value to receiving a recommendation that aligned with their own assessment. For these clinicians, the process was redundant rather than a source of reassurance.

I think it has huge value for people that are going to have to do this, you know, once every six months or once a year to their most senior patients. When … they’re doing well baby care and then the next patient they’re supposed to take away someone’s licence. I think it has huge value, there. [MD05-FP]

People who are addressing driving a lot in their practice may find it less helpful than people who aren’t doing it routinely. [MD07-SP]

… because I have the time to sit down with the patient and do some of that … I’ve spent more of that time looking into the patient … a different way that they may not have had time to do as a family doc. And so I found it was a great tool to be able to use as an NP. [NP02]

In general, physicians indicated that they would err on the side of caution regardless of the recommendation generated by the tool, and would either report the patient or refer them for a specialized road test if they had any doubts about the patient’s fitness to drive. In contrast, no nurse practitioners mentioned that they would refer the patient to be reported when the tool did not recommend doing so.

Family physicians and nurse practitioners were generally more enthusiastic than specialist physicians about the potential of the tool to add value to their practice. They emphasized the wide range of issues they had to deal with and the long intervals that might elapse between driving assessments. They generally appreciated the tool’s evidence-based approach to assessment of an issue that was emotionally complex, highly consequential to patients, and often required them to make judgement calls. Using the tool helped de-personalize the assessment process so that patients and their families were less prone to perceive the recommendations as arbitrary and subjective. In contrast, specialist physicians consistently pointed out that their current approach to driving assessment was more detailed and nuanced than what the tool had to offer. For this reason, the specialist physicians generally felt it had limited potential to add value to their current practice.

Specialist physicians generally did not foresee themselves incorporating the tool into their future practice because they felt their current approach to driving assessment in patients with MCI and mild dementia was already more sophisticated than the tool:

I do a very, very comprehensive assessment that I’m positive no family doctor would ever have time for. So, maybe a tool is more useful in that setting compared to, you know, my assessment where I have a lot of the information. [MD12-SP]

Reynolds-2019

“I think that it’s important and it would be a good tool especially for people that are mathematically challenged or something...”

Santillo-2019

Participants were also concerned about the potential impact of staff turnover

Silveira-2019

After a while, you get tired of reading the same things. You don’t have much time available [to deal with the repetitive content]. [Clinician]

However, some considered the repetition a CDSS strength.

Sometimes we remember to guide the patient only about medication, and we forget to talk about nonpharmacologic treatment. The alerts are repetitive, but it helps us to remember. [Family physician] I used to forget to ask about the salt in their diet. With the CDSS, I remembered to ask about it with every patient. [Geriatrics]

Soling-2020-supplementary file

“Such prescription chains are created, and I believe that these chains cannot be broken by specialists because they think too narrowly. And we as general practitioners, we have to try to break them up again with such instruments [digital tool].”

Sukums-2015

Newly recruited or transferred staff and those who were still unfamiliar with the software, particularly at the sites where less teamwork was practiced requested for more frequent IT support

Vanhille-2018

Unfamiliar technology “I can’t be absolutely certain until I have personally used this and confirmed the data…”

Wang-2018

Some pro-NOAC health professionals perceived that CARATV2.0 was biased towards warfarin and thus distrusted CARATV2.0’s recommendation when it did not recommend their preferred therapy. Similarly, several pro-warfarin health professionals questioned and disliked CARATV2.0’s recommendations because it did not allow negotiation with their preference.

To solve this problem, some suggested that pharmacists, junior medical residents, medical students or practice staff (e.g. nurses) could populate CARATV2.0 manually, allowing senior clinicians more time to review CARATV2.0’s recommendations.

Wang2020

Also, given that the senior coders have higher skill levels, they are more likely to detect imperfections in the AI output, which further deteriorates their trust in the AI:

“Many areas of the record are highlighted that are not appropriate for coding. Once one area of the record, whether or not appropriately, is highlighted, I need to review the entire record. I have not found this to be helpful.”

The comments suggest that as a result of their low trust in AI, these senior coders opted to review all the information in the charts rather than solely focusing on the areas highlighted by AI
