# Supplementary material for: Stakeholder Perspectives of Clinical Artificial Intelligence Implementation: Systematic Review of Qualitative Evidence
Source: J Med Internet Res. 2023 Jan 10;25:e39742. doi: 10.2196/39742 (PMC9875023; doi:10.2196/39742)
Supplement: Multimedia Appendix 3 [file jmir_v25i1e39742_app3.zip › 4. Adopters/4a. Staff/4a.2 Tools redefine staff roles.docx]

**Name:** 4a.2 Tools redefine staff roles

Ash-2020

So, the more this could be done on the front end by the front staff or by the board clinical staff, nursing or whatever before it gets into the provider exam room when they’re doing the document and charting I think the better it’ll be.

Primary care coordinators that are attached to our physicians…. The MA’s do not have a licensure. They can take a test if they want and become a certiﬁed medical assistant but they don’t require that right now for hire at the clinic.

• So yes, the MAs are awesome … everyone really has stepped up to the plate. Because I think if we’re truly going to have a working interdisciplinary and interprofessional clinical team, you have to empower your folks.

Benda-2020

Most respondents thought that the primary recipients of HNHC information would be care managers or social workers. Most also thought the information should be easily available to all care providers to help with care coordination.

If everybody is in the loop about it then of course that would be for the best. – EU01 [Facilitator]

Cai-2019

Several participants cautioned that any such human–AI calibration sessions should be tempered

to be as non-confrontational as possible: “The average pathologist, ifyou say, ’Oh you’re wrong,’ they’re going to be uncomfortable with that.” (P14). Rather than providing a direct comparison between the clinician and the AI’s performance, they recommended more implicit approaches that make the calibration feel less like a comparison, and more congenial: “When it does better than you, it’s always nice to have a gentle safe environment...Hopefully it’s done in a way where it’s positive, constructive." (P2) Participants suggested that, rather than providing a raw numerical comparison, it would be much more meaningful and actionable to illustrate what those differences were, as case examples.

Catho-2020

TI_09 (F, resident):“It removes the part of the study and personal research”

• GE_06 (M, resident): “I support the local antibiotic therapy guidelines but if we misuse them or use them too much, we forget to think”.

• GE_06 (M, resident): “We’re going to lose the ability to think by ourselves”

• GE_10 (F, fellow): “I just feel like I would be less interested in thinking about what I’m going to do”

• GR_01 (F, resident): “You want to be free to decide what you are prescribing, when you are prescribing it and you want to be free to decide if you are going to get the information or not”

Cresswell-2019

It was also viewed as important to avoid over-reliance on systems by those with insufficient clinical experience and to ensure that the system did not attempt to replace the expertise of clinicians. It’s useful to see amount of information available, but it could lead you down the wrong pathway and be in charge rather than the doctor. They [GPs] have the background knowledge of the patient and want to use their expertise. (Glasgow Workshop, Table Feedback

Dalton-2020

Participants acknowledged that prescribers must take ownership of the medications prescribed for patients under their care in hospital. Whilst most prescribers were happy to review the SENATOR recommendations, some attending prescribers showed a reluctance to act on the recommendations in the hospital environment or to take sole responsibility for older patients’ pharmacotherapy.

…whose role it is to actually do it? At the moment, I’d say it’s nobody’s role. Nobody really takes it upon themselves, I would say, to actively review patients’ medication like this. [Primary Researcher 2]

Gillan-2018

As AI technologies were seen to execute the ‘task-based’ elements of basic planning, computers would do the ‘heavy lifting, [with] a few people still doing the manual, classic stuff’ (RT07).

‘We’ll have to take on more complex tasks. Like probably those types of plans that are more complex, that require somebody thinking about what are we actually trying to achieve with this plan’ (RT05). MP02 noted a similar perception: ‘…it’s going to be the planners [who are most impacted] because they use the planning system all day long… and the computer will be doing that. And the planner’s job will be plan evaluation and maybe integrating it a little bit with the longitudinal considerations for the patient treatment’.

The perspective on how the daily workflow of ROs would change with AI was primarily discussed by the two RTT groups, with ROs suggesting only that they could focus on other priorities, such as that, ‘we don’t want to be doing stuff like contouring if the machine can do it for us. I mean I want to be able to use my time more efficiently in the most high-value activity. So you know - dealing with patients… looking over plans’ (RO02).

Expanding on the notion that adaptive planning would increase with AI technology implementation, RTTs raised the potential for a shift in responsibilities for ROs, primarily that

‘they’re going to have to be on the unit a lot more, especially for making decisions about delivering [treatment]’ (RT08).

It was noted that the volume of chart checking tasks would likely increase as the number of plans increased (with adaptive planning), but also that increased automation of the rule-based and repetitive aspects of these tasks would allow greater focus on evaluation of more complex plans and decision-making

TP-RTTs debated whether fewer treatment planning positions [full-time equivalents (FTE)] would be required, noting a general but perhaps unfounded fear of job loss. ‘That’s the buzz that’s going around, that we’re going to need less, and that’s freaking out a lot of people, but with the technology there, our jobs are going to change into something else, but they’re not going to go away’—(TP01).

TP01 suggested the need for all RTTs to be open-minded to accommodate the need to change, suggesting: ‘This is big. It is moving fast, and we all have to learn different ways of doing things. We all have to be… flexible’. For RTTs, it was felt that changes to their role with the introduction of AI would lead to making fuller use of their scope of practice. RT06 framed it as follows: ‘I think it would broaden the horizon… So you’re involving more, everything that we’re trained to do…. What we all train and learn for, we will use. Which I think really is a good way to go’

Those at the front line of treatment delivery also expect to become gatekeepers to adaptive re-planning. To evaluate the appropriateness of the current treatment plan, they would need to build on foundational dosimetry knowledge to ‘have an increased level of planning literacy to engage with the plans and the treatments in a different way’ (TP06). This would also introduce the need for the RTT to act increasingly as a clinical decisionmaker. ‘Role expansion – if AI could help us make clinical decisions. Like adaptive re-planning, if we have therapists and also an AI machine telling us we need to re-plan… when all the other indicators are there… it would be nice if we could use it that way going forward in our profession. There’s an opportunity there’—(RT07).

The need for new competencies and education required for this was also discussed for ROs and MPs. They were thought to require a strong appreciation for harnessing newly available data to guide clinical decision-making for adaptive planning. As noted by MP04, ‘I think clinical decision-making. If we’re doing more adaptation, there will be a lot of questions like when to adapt, what to adapt, things like that. I think AI will help with that, but ultimately they’re the ones making clinical decisions…I think now we just say ‘oh at 40Gy we take off the bolus’ or, you know ‘we rescan’. ….So… it will be a bit of a paradigm shift and [ROs] have to also get used to that. That will apply to their training as well, because right now I don’t think a resident would know how to do these things. It’s not in their training, in their curriculum’.

Goetz-2020

The participants believed that the vPCP could complete simpler tasks (documentation, pre-

scription orders, etc.) so that human physicians could focus on and complete more complex tasks.

“I could imagine a physical could be something the machine could do ‘cause it’s not really something you need to diagnose. Just putting in a bunch of data.” (Fourth year medical student)

The use of a vPCP would also lessen manpower demands, both for clinics and at-home caregivers. “. . .there are caregiver roles that you have to play for family members, and if this work could be done by the tool, it could actually be very helpful.” (First year medical student)

Johansson-Pajala-2017

Some RNs spoke of the CDSS as yet another computer system which increased the time they had to spend on administrative tasks. They claimed that they had become more like administrative personnel than nurses participating in bedside care. Subsequently, the CDSS was perceived as depriving them of valuable time that could have been spent, caring for their patients.

‘It always takes time to sit by the computer, it actually takes time away from the patients ... ’

Some RNs perceived that the CDSS was a tool mainly for them. This was because RNs were closer to the patients and thus could see and assess changes in the patients’ health condition which could be related to their drug treatments. Some RNs even thought that they should take more responsibility and view the reports in more detail. ‘He [the physician] reads the reports from the CDSS... he looks at them and then makes a decision... I’m a little on the side, too much on the side, which I do not like, and it depends on myself’

This aspect included perceptions about shared responsibility. According to the existing routine, it was the RNs who planned the medication reviews and entirely handled the CDSS. Many RNs expressed that the physician should take more responsibility in this process. They requested greater involvement and interaction with them, which could include aspects of dedicated time, interest and suggestions about potential drug adjustments.

‘If the physician had more time, and more, if I am to be mean, interest, then you could work more actively with it [the CDSS report]’

Jutzi-2020

Respondents were also worried that physicians might be tempted to rely on the AI-based algorithm so much that they would lose their own expertise and diagnostic skills. This would reduce their ability to classify lesions without the assistance system as well as their ability to notice obvious mistakes or malfunctioning of the algorithm itself, which could occur due to various technical problems or even deliberate manipulation by hackers

Keogh-2019

There was little point in saying, “Go back to your [PCP],” because it was the [PCP]s that had said, “I don't know what to tell you, I'm referring you on.” I had one [PCP] try to use FRABOC [Cancer Australia's tool ‘Familial Risk Assessment e Breast and Ovarian Cancer’] and got completely confused … They didn't know what to tell these women. These women wanted something, they were a very vocal group.

Lai-2020

Finally, physicians remained cautious about the message being delivered by those in industry who, depending on the context, may or may not have suggested that some AI tools will replace physicians (e.g. medical imaging).

He explained that, in this discipline, one of the main ideas is to delegate only when necessary. If not, there is a risk of deskilling. Deskilling is the loss of competence of the human who does not know how to carry out a task that he did before because he stopped performing it for the benefit of the machine

Lennox-Chhugani-2021

A fear about a combination of over- reliance on AI and job losses that might ensue (n=32)

Liberati-2015

[“Either me or him”: the SSDCs and the spectre of control

"I disagree that medicine should to be based and guided simply from a system ... I believe that to guide decisions must first and foremost be the preparation of a doctor, his conscience and experience. It is demeaning to think that any activity of the doctor can be driven by a computer! Because I have one my professionalism to my deontology. In the si- patient safety, I have to act my own way and have the courage to write or do what I think is useful, I don't have to settle on the guidelines dictated by a program. What you can give me see 150 patients is definitely my baggage and not me a system of this type (SSDC) gives it ». (Surgeon orthopedic, setting B)

Clinicians who identify with this position operators see SSDCs as a "usurper" of its own competence and expertise if: in their representation, the system cannot be controlled or governed, but rather its alone presence forces the clinician to abdicate exercise of their profession. The SSDCs, in other words, are depicted as an "omnipus-tente ", leaving the doctor only two options: nose or total adherence to the suggestions proposed. In this positioning, clinicians hypothesize one real possibility of using SSDCs, which is nevertheless lived in a conflictual way. If the SSDCs come activated, the doctor is forced to renegotiate the contract control and mastery of its activities with respect to new system]

Liberati-2017

These barriers resonated with the opinion of some IT staff:

Physicians are afraid of being turned into bureaucrats. Physicians’ power and authority derives from the diagnostic and therapeutic process: physicians don’t want to take their hands off the patient and sit at a desk to write at the computer, they don’t want feel like they are doing data entry. (IT specialist, setting A)

The CDSS is perceived as encroaching upon physicians’ competency and jurisdiction; rather than a useful support to their practice, the CDSS is described as a potential hindrance to the exercise of clinicians’ judgment and critical thinking. Clinicians seem to be left with two opposite options: either rejection or unconditioned adherence

Disputes over power and control seem to involve other organizational actors as well. One physician felt that, since the CDSS allows widespread access to scientific evidences, the system might lead to nurses’ control or oversight of medical decisions, thus providing an occasion to renegotiate professional boundaries. If for some reason I don’t follow the recommendations of the CDSS, a nurse may notice this and say “According to the CDSS, the doctor made a mistake”. Do you see what I mean? [...] If we want to implement it it’s key to discuss the rules of access for each profession. (Surgeon, setting B)

Maybe I could use it. I think it would be more useful for young physicians, those who have only just graduated, or those with little experience... You know, to avoid mistakes... (Senior physician, setting A)

I think it would be ideal for general practitioners… More than for us in the hospital. (Physician, setting B)

Melo-2020

Some participants considered the relocation of work resulting from the application of 4.0 technologies as a risk factor, while others considered it an added value because professionals can work without physical offices and in a much more flexible manner

Miller-2019

Most providers believed nurses and physicians can use the system. Some felt it would be better for the RN to administer the survey; some felt any member of the team including front-line ordering providers could administer the survey

Nelson-2020

human deskilling (10 [21%]

Orchard-2014

Receptionists expressed ease with using the iECG device but were often reluctant to ask patients and

generally felt inhibited. • ‘I’d always get a little bit nervous about asking people’ (Receptionist 2)

Receptionists were unsure how to respond to patients' questions and generally felt this duty was not part of their role. They did not see the relevance of screening for stroke prevention. • ‘Patients would say, “Is this my heart rate?” and I would say, “I don’t really know”’ (Receptionist 2)

Pannebakker-2019

Furthermore, there were no discussions around how GP colleagues could support its use. A few considered whether people other than GPs, such as practices nurses or non-clinical staff, could safely use the melanoma eCDS. Although they were felt to be technically capable, it was generally felt that a GP needed to make the clinical decision: ’[Practice nurses] haven’t done the training and they haven’t done the years of looking at skin lesions that we get every day.’ (M, !51 years)

Indeed, some showed clear antipathy to any form of guideline or checklist: ‘ ... checklists are for robots.’ (F, 41–50 years). ’I think the downside of . . . making everything a tick-box exercise, does take away your clinical judgment, you can deskill. I don’t think that’s such a risk with this actually.’ (M, 40 years)

Park-2020

Negative responses included concerns that AI would result in “doom for diagnostic radiology,”“AI will take over all interpretation of imaging, EKG’s and anything else not acquired from an H&P,” and “artiﬁcial intelligence could potentially make radiologist(s) obsolete.” There was even some dark humor, with one student saying that “a mentor of mine told me that radiology is Blockbuster and AI is Netﬂix.

Patel-2018-additional file

Main GP: the administrative side of it needs improvement. If you ask the doctor to just go and look at it [CAT] himself every three months or so I think you’ll be hitting the wall. Okay. But if you get an allocated person to say “Well I’ve been allocated this task, and so every three months I do it [review CAT].

Main GP: A good single person allocated, keep monitoring, keep going, these tools will be very, very good. Yeah.

Effort Main GP: It cannot be based on just me to do the analysis all the time, which is impractical at the end of it…fully integrated in terms of not just the doctors are using it, the staff are using it at the same time. So you need a bit of education for the staff, show them how to install it, how to log in, how to logout, where to get the information, which area they should be looking at, maybe even our administrative side of it, to help the doctors maybe.

Non-GP staff were not involved and not interested. This would have assisted in overall use of the intervention and integration

AHW: it was interesting though that we were still shown it [intervention], and, so, you know. So you sort of, look we’ve got this great tool here, but guess what, you can’t use it. It was sort, that’s what it felt like to me.

Pope-2017

This work appeared intense. Call handlers with experience of previous systems suggested that the content of the job had grown and that it was exhausting:

Call handler: After a shift, I’m going home, and I’m just, like, [sighing]. [Laughter]. […]it’s mentally draining. […]it’s not like somebody’s ringing up to book a car for something, […] what I’m finding hard, as well, some of the calls, they can last for 20 minutes, and you finish that call, and then the phone rings again, and bang, you’ve got to go straight on to the next one, and just put that one behind you, and start again (Focus group, NHS 111).

This labour intensification was noticed by other staff. One of the doctors we observed said that the new system had doubled the workload for general practitioners (Observation, Urgent care centre). In addition to increasing the everyday work, the new system required new training activity for the system to be brought into use. Call handlers received six weeks’ formal training in the early sites we studied, and as the NHS 111 service expanded, this was condensed into two more intense weeks in the classroom. Delivering this training was made complex by the presence of a largely part-time workforce, so that formal training sessions were often duplicated to ensure that all staff were able to attend. This intensified the work for the staff engaged in training.

Porter-2018

Several paramedics discussed how the CCDS contributed to a shift towards a greater role as independent decision-makers, without taking over from their own clinical judgement:

Clinical decision making is still my primary role, like, so it’s up to me. (End S2 04)

Sun-2019

“Doctors may feel they will be replaced [by Watson]. Because they [i.e., the doctors] made many efforts to achieve their status. [3IT04]”. Such a fear, however, is nuanced by the fact that AI is framed as not capable of replacing specialized, skilled work. As pointed out by a government official: “Some simple and boring work may be replaced by AI. But not all jobs” [1GOV01].

Trinkley-2019

One clinician stated, ‘in a lot of ways we are creating clinicians that don’t necessarily know pathophysiology and patient assessment and what they are learning is algorithms for treating patients and best practice alerts that tell them what to do’

Tsang-2021-Supplementary file

“I hate having to use my brain for something that a computer can do for me.” [P7, pharmacist]

Van de velde-2018

Several participants preferred that the GP would act as an intermediate recipient of the patient-directed CDS:

An alternative approach is that the GP gets an overview of the CDS for the patient and then decides if it is relevant to forward it to the patient. [GP, Norway]

Vedanthan-2015

since the vitals need to be entered directly in DESIRE, nurses have adapted their workflow in different ways. Some nurses have trained the patient assistants to input vitals into DESIRE while others record information on paper and then input data into the system after the encounter.

Wang-2018-Tables

we might be in a situation especially with patients in nursing homes things like that. I would not feel confident without a pharmacist’s recommendation, but with that tool (CARATV2.0) I can possibly.

Also, we have got a chronic disease nurse, and she could administer it and fill the answer then that could also make it quite easier for us.

Wells-2014

In principle there was support for the potential of CCDS, it was not seen as a threat to paramedic autonomy but as another tool in the kit bag

Yang-2019

Physicians highlighted that the predictive models, regardless of how well they measure medical uncertainties, would never replace human, clinical decision-making. They viewed their own decision making as focused on managing and reducing uncertainties. “If we think that we will be able to tell everybody what to do based on a model, we ignore the fact that we also have tools and mechanisms for dealing with the uncertainty that is inherent when putting VADs in patients.” (Cardiologist
