# Supplementary material for: Stakeholder Perspectives of Clinical Artificial Intelligence Implementation: Systematic Review of Qualitative Evidence
Source: J Med Internet Res. 2023 Jan 10;25:e39742. doi: 10.2196/39742 (PMC9875023; doi:10.2196/39742)
Supplement: Multimedia Appendix 3 [file jmir_v25i1e39742_app3.zip › 4. Adopters/4a. Staff/4a.3 Aligning with staff values.docx]

**Name:** 4a.3 Aligning with staff values

Andrews-2017

Participants also expressed concern about the requirement for staff to follow up with patients who were predicted to score highly for depression or anxiety. Several participants believed staff would avoid conducting preventative screening practices if there was the possibility it could lead to extra work.

P5: With the pressures that we’re under, predominantly to get people out of hospital, to get them home and to keep them safe, and then the other end prevention, it would be another piece of work really, and it’s not so much answering it, it’s then doing something with that knowledge isn’t it

P3: Once you’ve screened for something, you’ve got a responsibility to follow that through and do something about it, otherwise it’s neglectful. And I think that’s actually… P4: So if you don’t ask, you don’t have to follow it up. And that depends on the professional really, as well.

Baysari-2017

Regardless of the method used to record an indication in the CPOE, it became immediately apparent that prescribers did not view documenting an indication for antimicrobial use as a priority. They are still getting the right dose, it’s still the right thing, it’s still on the computer, I guess what difference does it make. (D6)

Several participants explained that seeking approval for non pre-approved indications was time consuming and interrupted their workflow. When time pressured and rushed, prescribers often entered or selected an approved indication, even if not completely accurate. This removed the requirement for prescribers to step away from the prescribing process to contact the antimicrobial stewardship doctor and saved time. Many doctors reported choosing indications that were partly consistent with what they intended to prescribe the antimicrobial for. I reckon maybe, if they are in a really big hurry and they just want to prescribe a drug and it fits the criteria in terms of dose and time but might not fit the actual indication, so people might just take that short cut if there is a long list. (D6) But I know some of my colleagues will just type something in which won’t make any sense. They just type a few keys just to proceed to the next level…I don’t think that that is done to challenge the system, it is just done so we can give the patients the right antibiotics. Cause you know we are never going to chart something that we don’t think is appropriate but…I guess you could say that the on-line system could be viewed as a barrier.

Beede-2020

As a result of the prospective study protocol design, and potentially needing to make on-the-spot plans to visit the referral hospital, we observed nurses at clinics 4 and 5 dissuading patients from participating in the prospective study, for fear that it would cause unnecessary hardship

Nurses had to consider their willingness to follow the study protocol, their trust in the deep learning system’s results, and whether or not they felt the system’s referral recommendations would unnecessarily burden the patient.

because of the large inconvenience it would cause patients to go to Pathum Thani Hospital, nurses were generally hesitant to refer patients there as a result of ungradable images alone, given the low likelihood that they have severe or proliferative DR.

Instead, we observed nurses working around the prospective study protocol; relying on previous workﬂows and criteria for determining whether or not to refer the patient to a specialist. I look at results and then determine the blood sugar result, the HbA1c. [Sometimes] people go to the hospital and it’s a waste of time. You should always look at the blood result and if one eye is okay [and the other is ungradable], then I don’t recommend it. But every time I ask if they want to go. -P6

We found that nurses took this approach across the three sites: in the case where an image was ungradable by the system, but the patient had no history of diabetic retinopathy and their blood sugar was well-controlled at the time of the visit, the nurse would make her own judgement call to send the patient home without a referral. P8 described what she does in the case of an ungradable image at her clinic, “I look at their history. If it was bad last year, I refer. If it’s okay, I send [the photo] to the doctor.”

Bourla-2018

Much of the feedback focused on the third scenario (which had the highest acceptability profile), with questions about which course of action to pursue if the MRI or the blood tests predicted a transition to psychosis and pointing out the risk of jumping to diagnostic conclusions. Several respondents indicated that they would refuse to introduce a pre-emptive antipsychotic treatment based on a prediction made in this way. Several commented that there was no point in predicting an incurable disease. In

Cai-2019

To develop an understanding of the AI’s grading competence and potential biases, some pathologists envisioned assembling a set of cases with ground truth (e.g., as assigned by GU pathologists), and comparing their diagnoses and the AI’s diagnoses with the ground truth in a calibration phase. This practice would serve dual purposes: It would give insight into the AI’s diagnostic tendencies, as well as their own: “[The] AI would look at a slide, I would look at a slide, and then we would know what the expert said. I think that would be interesting just to see where I stand.”

Chow-2015

Senior physicians acknowledged that they tended to have personal preferences for antibiotics and that they would ‘ignore’ ARUSC’s recommendations in situations when they needed to be ‘aggressive’ with therapy based on their prior experiences with similar patients [S3, S4].

‘I think part of the challenge is that we all have our personal preferences’ [S3]

Dalton-2020

It was noted that some prescribers would not implement the recommendations unless there was a clear risk or benefit to the patient. I’m not gonna start interfering with somebody’s medications unless there’s a glaring danger in them or I see something that’s absolutely contraindicated…. [Surgical Prescriber 2]

Interviewees pointed out that many of the recommendations were focused on the patient’s chronic disease management, whereas the prescribers were primarily focused on the acute issues.

…the clinical team only deals with the acute problem. They are not interested in looking into the other medications…. [Primary Researcher 9]

Some of the prescriber inertia was due to individuals’ lack of motivation to review pharmacotherapy—unwavering in their intentions despite the SENATOR intervention.

…the doctors that don’t want to make a change, they’re not going to make a change…. [Primary Researcher 4]

Dikomitis-2015

Some GPs indicated that, although the eRATs might not have greatly inﬂuenced their referral rates, use of the tools meant that they reﬂected more often on symptom presentations or looked back at patients’ records: ‘Yes, I mean, I suppose a lot of us as GPs we do feel we’ve got a bit of a nose for a problem, you know, and what we want it … you know, I don’t send everybody with these symptoms up but I’m sending this one up because I’ve just got this gut feeling that this doesn’t feel right, you know. And you want that respect in a sense. And it might not ﬁt in with your grid of symptoms [laughs]

Hallen-2015

Another concern related to overconﬁdence was that prognostic estimates from CPMs could be misused either for ﬁnancial gain or to ration care at the end of life:

Oncologist 1:

I mean [a tool] may even be used to discourage referral. Sort of a stairway to manage healthcare resources but I’m sure it’s done a bit. Sort of a rationing tool....

Geriatrician 1: Well, it is hard enough to certify that somebody has a 6-month prognosis for hospice... So suppose you refer somebody...and he doesn’t meet the [tool’s] criteria for some reason and they reject him because of that... I just worry that if he doesn’t ﬁt in that model then they can exclude it inappropriately.

Interviewer:

Alright, so your worry is that then people would use it in a way that denied appropriate hospice care to certain patients.

Geriatrician 1: Primarily that. I suppose you could take it to another extreme and say that somebody is going to go ﬁshing for hospice patients with this model too

Klarenbeek-2021

Other suggestions to increase uptake of the system were involvement of clinicians from multiple disciplines in the further development of the system and implementation, and creating opportunities for users to become familiar with the CCDSS.

A few professionals also highlighted involvement of management teams in the implementation process because of their responsibility for ﬁnancial and contractual agreements with suppliers. [Professional ID:6] ‘Involve the managing directors of all involved medical departments. I assume that due to the system, these departments will get an additional ﬁnancial burden. To ensure back-up from the management, you must be able to provide insight in these costs.’

Lytle-2015

Discussion about the fall risk assessment indicated there was confusion about the definition of high risk among the staff on the medical unit. Staff felt that even though some of the fall risk assessment answers were “yes” or positive the patient was not at high risk in their judgment.

Miller-2019

Not an ED physician’s responsibility to provide emergency contraception for every patient having sex.”

“A small portion of this goes against some of my personal beliefs, although the beneﬁt is there as well for a large majority of this system being used in our patient population.”

Morgenstern-2021

It was thought that cross-training of those with public health expertise, including medical students who become public health physicians, would be helpful. They need to be exposed to, here’s really basic high level intuition of how machine learning works. Here’s what the tools can do for you as a physician whether you become a radiologist or public health physician. And here are some examples of how they’re applied. And that’s really all you can do. [Participant ID # 4].

Mozaffar-2016

Implementations also overran due to issues with human resources, which were particularly intractable as projects spanned many departments and professional groups across the organization. These complex projects thus required an implementation team that spanned the organization and continued to support the implementation over an extended period. However, some hospitals lacked the local expertise needed in the implementation team. They failed to recruit people on time or lost project employees during the course of the implementation.

… the reason we slipped from our February go live initially, we wouldn’t have done it then anyway, to April and then May is we didn’t have our full team in place, we couldn’t recruit them. (Site C, Project Manager)

These teams sometimes had difficulty in engaging the necessary stakeholder to execute their plans. On the one hand, certain professional groups were not involved/invited to become part of the implementation team or when they did, their input was seemingly ignored; on the other hand, there was a feeling among the implementation teams that there were many individuals who chose not to get involved (i.e., they deprioritized the CPOE/ CDS implementation).

Pannebakker-2019

There was a range of views about whether using the eCDS was of more value during the consultation to help with decision-making, or after the consultation for record-keeping, referral, and to confirm their management decision:

’Without the checklist I already know what to look for. I know that if it’s changed in size, if it’s irregular, that those are all serious ... So I would have already gone through it anyway, with or without the [list] in front of me, so does it really matter? Probably not. It’s in my head like any other medical problem, I mean, I consult all day long.’ (F, 41–50 years)

Patel-2018-additional file

Main GP: I remember you and xxx [chief investigator] would come around and you’d explain things and there would be things that I would see the benefit of, and then it was like “Okay, I’ve learned 25 new things. I remember three very well, I know how to use five, and I know I can sort of do this but I don’t quite remember how to do it”, and then after three weeks you’d think “Was there something like that there or not?” And you thought “Okay, I can go for a walk or I can sit here for half an hour and work out how to do it.

I’ll go for a walk”.

Petkus-2020-supplementary file

My concerns however about CDSS include to whether the systems may inhibit junior clinicians' thought processes about individual cases particularly about looking at the bigger picture of the case, especially when a presentation might be atypical or complex or an alternative diagnosis should be considered. CDSS have the potential to alter training and the benefits of a comprehensive clinical experience.”

Porter-2018

Although the CCDS was designed to be used at the point in time and place where the patient is being assessed, some paramedics reported using it retrospectively to save time, not as decision support but rather to document their assessment and care decisions.

I tended to use it after the event to be honest. I said we'll pick them up off the floor, do all our checks, decide what we're going to do then–, and then kind of go through the software. (End S2-03)

Rapoport-2020

This person was having some issues with diabetes management. And so had a little bit of a setback, and didn’t do as well in terms of the cognitive testing that was usual for this person … it could have been that the blood sugars were … out of target range for the past prior three months … I didn’t feel comfortable with reporting, because I knew there were other factors. [NP05]

Roebroek-2020

TREAT offers several treatment recommendations (Fig. 3) for clinicians to consider with their patients. Some clinicians found these recommendations helpful: “That’s what I like about TREAT; you are not forced to follow for example a recommendation to start an anti-depressant in case of persistent negative symptoms. You just discuss it, like is this something you would prefer or not. Maybe you both decide to try something else. Either way the recommendation is still valid, it’s just not mandatory.” [C6]

However, others found the recommendations bothersome or felt pressured:

“I mean, I know it’s not mandatory to follow the recommendations, but it still feels that way. Sometimes, you’re just happy that somebody is using the medication you prescribe at all, and then you get the recommendation to switch the medication.

Sun-2019

“Most of the IT personnel should have a PhD degree, and the same with medical personnel. It is really hard to find this kind of talent [in the local market]” [3IT04]

Trinkley-2019

Clinicians reported CDS might be better accepted if the CDS could be temporarily dismissed. One clinician stated, ‘if the patient is going through an acute thing and you don’t want to address [the interruptive alert] right now, say remind me in ninety days or something like that, I think would be helpful’.

Clinicians also wanted more flexibility of how to respond to CDS, stating ‘I always want to do something else’, other than the options given to select. Some clinicians suggested having an ‘other’ response option that allowed them to explain and prevent them from feeling forced to respond in a given way

Tsang-2021-Supplementary file

Ability to feedback system improvements

• “I think that it will help improve the programme if we know who...yeah if we know about the accuracy, so that's why I do it.” [GP3, doctor]

• “I couldn’t work out how to stop them flagging. So I sent a comment to the team, and changes happened. Until you start doing stuff like that, you don’t really get involved…we all just need more time to do it” [A4, administrator]

• “It’s quite intuitive in the ability to send a comment straight back as well. A very responsive team to work with and that’s helpful cause nobody’s got time to separately contact whoever’s developing it.” [P5, pharmacist]

• “I know that there’s feedback button, I try to think what the system could be doing to make our processes better as well. So that two-way feedback.” [P7, pharmacist]

Wang-2018-Tables

I can see it (CARATV2.0) would be useful for GPs that don’t have access to a cardiologist … It is good for doctors who are not certain or don’t have access to cardiologist … The new doctors love to have something like that, because it gives them the confidence to manage the patient and to be able to assess the patient to know what they should be doing.

if you have cardiologist who have been using warfarin for the last 40 yrs and why they are going to change to NOACs? No. If they have someone who is on warfarin and adherent why change it … So it (CARATV2.0) doesn’t really consider the clinicians preference … Well it is nice to give a recommendation based on international guidelines validated risk assessment tools, I think it doesn’t consider the clinicians previous experience of the agents and that is going to influence their decision making. But I think this is still useful tool. (N01)

Watson-2020

Independent of the cultural barriers to clinical implementation of these models, there were personnel limitations. The demand for people skilled in the creation and maintenance of these models is significantly larger than the number of people available to work the models. Shortages and turnover of personnel with the requisite skill to develop the model as well as maintain the model created barriers to implementation. One interviewee captured this challenge as follows:

When people with institutional knowledge move on to other institutions and their institutional knowledge is particularly targeted on the machine learning models that are in production, that creates a knowledge gap and also a sort of responsibility gap that must be filled by someone if these are to be continued or being entirely abandoned due to lack of funding.

Wickstrom-2020

They experienced increased engagement when personally taking part in the development of a new eHealth solution and were excited to evaluate how it could be used within health care. The participants’ engagement was also influenced by whether or not they were personally affected in the introduction of a new working method, for example, if the new work tool facilitated their work and gave them a direct personal gain or if they just used the DDSS in passing.

Yang-2019

Some surgeons described that, for some cardiac surgeries that have officially defined models used to rate surgeons and care teams, their decision meetings had became centered around risk models. This is not yet the case for VAD implants.
