# Supplementary material for: Stakeholder Perspectives of Clinical Artificial Intelligence Implementation: Systematic Review of Qualitative Evidence
Source: J Med Internet Res. 2023 Jan 10;25:e39742. doi: 10.2196/39742 (PMC9875023; doi:10.2196/39742)
Supplement: Multimedia Appendix 3 [file jmir_v25i1e39742_app3.zip › 4. Adopters/4b. Patients/4b.1 Inconvenience for patients.docx]

**Name:** 4b.1 Inconvenience for patients

Abdi-2021

“right cut-off between intrusiveness and quality of the data.” AI-enabled wearables,

Abejirinde-2018

One midwife mentioned an extreme case where the turnaround time was too long, and a woman got restless and left the facility.

Abidi-2018

Patients were worried that if they failed to achieve the goals that they have set through DWISE, they might lose respect in the eyes of the PCP, or disappoint them, and might feel burdened or stressed:

I mean respect is a two-way street...what if I don’t meet that goal...what would my doctor think about me?

Andrews-2017

A particular area of concern was cost. In participants’ experience, older adults were often concerned about the cost of running technologies provided to them.

P3: And the cost of the patients using it, whether it is electricity or, or they’ve got, if they’re using a mobile, have they got to top up

P4: they just worried about breaking it, and how expensive it is, those sort of things, and how much electricity it’s using, is a big thing. How much it’s costing.

Ash-2020

However, even though the CDS modules we described were not triggered by work information interviewees were quick to spontaneously point out that patients could be routinely entering job information themselves, so that it is always up to date

So, that would be kind of nice if the patient enters the data. So, I don’t think it’s a question of how reliable it is … it’s just as reliable as anything else you’re going to get from a patient.

You know, we’re trying to ﬁgure out how not to overwhelm the patients with the amount of information we want to collect from them.

Beede-2020

Nurses were also concerned about the consequences for patients if the algorithm produced a false positive, including the additional travel burden to follow up on a referral, the cost of missing work associated with travel, and the emotional strain a positive result could place on them

While patients at all sites were given the same information via written consent forms, some nurses felt the need to “warn” patients that they would need to travel should a referral be given. Given the far distance and inconvenience of getting to Pathum Thani Hospital, 50% of patients at clinic 4 opted out of participating in the study, even though it was unlikely that they would be referred.

[Patients] are not concerned with accuracy, but how the experience will be—will it waste my time if I have to go to the hospital? I assure them they don’t have to go to the hospital. They ask, ‘does it take more time?’, ‘Do I go somewhere else?’ Some people aren’t ready to go so won’t join the research. 40-50% don’t join because they think they have to go to the hospital. - P6

Bourla-2018

Generating anxious counter-reactions (wearable device, prediction)

Lawton-2014

Some participants (n = 2) reported stopping using their advisors because they did not know how to change the settings or they found data entry too time-consuming and burdensome:

‘‘you know it takes quite a long time to type all the numbers in and it’s quite ﬁddly and stuff. I just want to do a blood test, see what I am, wallop some insulin in . . .I think if I used it, I would get tighter control. It’s just that, in using it, it interferes with life more than I want it to. It would be, you know, a frequent inconvenience’’ (M32.2).

Nelson-2020

patient loss of privacy(14 [29%])

Other perceived strengths were more convenient diagnosis (14 [29%]

Pannebakker-2019

The patients were universally unaware that the melanoma eCDS had been used during their consultation about a concerning pigmented lesion.
