# Supplementary material for: Stakeholder Perspectives of Clinical Artificial Intelligence Implementation: Systematic Review of Qualitative Evidence
Source: J Med Internet Res. 2023 Jan 10;25:e39742. doi: 10.2196/39742 (PMC9875023; doi:10.2196/39742)
Supplement: Multimedia Appendix 3 [file jmir_v25i1e39742_app3.zip › 4. Adopters/4b. Patients/4b.2 Patients' control over their care.docx]

**Name:** 4b.2 Patients' control over their care

Abidi-2018

Participants felt that DWISE may help improve patient’s awareness of the disease and can be used as a teaching tool for patients: DWISE makes me more aware...more informed...I feel like I want to know more so that I can better take care of myself.

A patient also stated that DWISE has potential to improve power dynamics between the patient and PCP and help patients gain more control over their diabetes management: I feel balance of power is always in favour of my doctor...it’s not bad...but I like to be more involved...make decisions that fits my life...DWISE can give me more control.

In general, participants felt that using a tool such as DWISE might make them feel more empowered to self-manage their condition.

PCPs underscored that a patient might be more prepared during the encounter:

Every patient is different...and self-management requirements vary so much...so patients coming prepared will be so good for the appointment...I think appointment time will be better spent.

Patients expressed that they will be more motivated to comply with plan set through DWISE to have a meaningful encounter:

There are higher problems that are not in my control...that might mix the schedule...but I will still try to do this or change it to have a better appointment...I’ll go to the appointment with something...

Adams-2020

Participants expressed a desire for patients and the patient community to have a role in developing AI algorithms and collaborating on implementation.

Ash-2020

Several interviewees emphasized that their most vulnerable populations would receive the greatest beneﬁt from our proposed CDS.

Cresswell-2019

The expected areas of impact frequently aligned with important policy drivers around guideline implementation and person-centred approaches to decision-making in health and social care settings.22 23 For example, stakeholders talked of giving patients more power to decide on their own treatment. A typical response was:

…I think hopefully it’s a better care for that patient and a more informed patient as well, so they know more about their treatment and why they’re on a treatment and therefore can help…making a decision in conjunction with the clinician. I think that’s going to be quite beneficial for patients

Several stakeholders in the workshops and in interviews also highlighted some potential tensions between decisions of clinicians and patients, with GPs generally being more risk averse than patients. Some therefore argued that DSS should be patient informed and not patient led, with the ultimate decision of treatment being in the hands of the clinician.

Patients take more risks than doctors but the clinician is responsible for them it should be patient-informed but not patient-led. (Participant 20, male, GP)

Hallen-2015

Physicians reported that the prognostic conﬁdence provided by CPMs would empower them to take a more directive role in cases in which a right decision truly existed but was somehow unable to be discerned by patients – when patients ‘really just aren’t seeing the forest for the trees’, as one oncologist put it.

Cardiologist 3: For the most part, though, I really try to get a sense of what the patient wants and what the family wants ... I try to respect that. It’s really when I think they’re making a mistake in judgment. I think there are situations where sometimes they think they’re too old for a procedure but really they don’t have a lot of other morbidity and the risks probably are pretty good to have something done. So to me it might be helpful as one other factor that could point them in certain direction

Henshall-2019

Patients/carers felt that receiving information about the likelihood of experiencing a side effect and its subsequent severity would enable them to weigh up the risk versus benefit ratio, informing their decision-making. Weight gain in 3% of the population … You’d probably say, I’ll be okay with that even if you are already overweight. Whereas if it’s 50% of patients that use this put on weight, you’ll probably think, then I’m not so sure. Patient/carer

something which was not factored for by the DST. Despite this, psychiatrists felt that it could be a useful base for considering medication options with patients. You’re much more constrained in the antipsychotic you could give each patient … But it could still be used as a launch pad for those types of discussions. Psychiatrist

All participants felt that patients should be involved in decision-making around medication choices and that the DST promoted informed choice, patient engagement and discussion around potential trade-offs between side effect tolerability and drug efficacy. This fulfils, or could fulfil, a valuable purpose in getting a bit more buy-in to treatment from patients. Because the drug has come out of their preferences, and not you saying, you should go on this. Psychiatrist 4

Jackson-2017

‘Participatory design and evaluation is increasingly highly regarded. To get representatives of the community, right through the whole process is really essential’

A majority of the participants endorsed the use of automated algorithms to facilitate self-management as a primary aim of the decision support tool.

One participant stated that the intervention needed to be ‘patient-centred’. ‘Including patients in the design phase’ and ‘conducting focus groups for patients’ were suggested to improve implementation of the eHealth intervention.

Lai-2020

Moreover, patients felt that they were not sufficiently consulted by industry, especially concerning the evolution of these tools.

Lawton-2014

While two participants chose not to use an advisor from the outset because they were worried it would deskill and disempower them: ‘‘I did feel like it was talking the control out of me, I mean they spend a lot of time teaching you DAFNE, the principles and then it’s suddenly said, ‘now forget about that; the machine will do it for you’’’ (P28.1),

McCradden-2020

It’s ethically incorrect, as you are picking and choosing who gets treatment. You need to give them options and have conversations with the patients. (Participant 18–008, provider)

Roebroek-2020

Another clinician noticed a greater sense of ownership for patients while using TREAT:

“It really has to do with ownership of the data. If I have a ROM-letter with a lot of text, it feels like I own the data. With TREAT there is a subtle nuance in how it feels, like you give the patient more ownership and make them the owner of the data.” [C2

Shannon-2021

Specifically, the Laddr© app provides educational content that helps patients better understand their conditions.

Torenholt-2021

‘With the implementation of PRO-data in clinical practice and in the quality of care development, the aim is to put into reality the vision of involving patients more in their own treatment’ (Trygfonden and ViBIS, 2016: 37)

Van de velde-2018

Both patients and GPs mentioned that informing patients better can increase the potential for shared decision making:

Patients need direct access to CDS so that they can prepare themselves for a consultation. [Patient, Norway] It is an advantage when reliable information can be sent to the patient, because GPs often have to use time to reassure patients that have read inappropriate information from unreliable sources. [GP, Belgium]

GPs asked if CDS could help them to identify patients that are coping badly with their disease. This would make it possible to devote extra attention to those patients that need it most.
