# Supplementary material for: Stakeholder Perspectives of Clinical Artificial Intelligence Implementation: Systematic Review of Qualitative Evidence
Source: J Med Internet Res. 2023 Jan 10;25:e39742. doi: 10.2196/39742 (PMC9875023; doi:10.2196/39742)
Supplement: Multimedia Appendix 3 [file jmir_v25i1e39742_app3.zip › 4. Adopters/4b. Patients/4b.3 Aligning patients' agendas with tool use.docx]

**Name:** 4b.3 Aligning patients' agendas with tool use

Adams-2020

Participants wanted their physicians to have the best tools available that are validated and trusted by their health care providers.

Andrews-2017

This participant also discussed how patients may feel keener to engage through technology since they would not be taking up clinician’s time:

P4: it gives them an added reassurance, they’re not bothering clinical people

Ash-2020

Just the thought process, you know, for some patients would be, so since you’ve addressed this, and this is part of my medical record, I want to apply for disability, and it may send them down a different path than they had originally arrived to see the provider for

Dalton-2020

Some interviewees stated that the patient’s preference was a factor in whether the recommendations were

implemented, and that patients would be resistant to deprescribing of certain medications.

Of course, also patients’ will. Like it always suggested to stop the sleep medication but most of the people we try to stop the sleeping medication, they will shoot you. [Medical Prescriber 4

Jackson-2017

It was agreed that, although feasible, social media was not a priority and should not be integrated within the decision support tool

Keogh-2019

While consumers were not offered information about their personal risk of BC as part of this study, they were asked whether they would be interested in knowing this information, should it be

available. Some expressed that, personally, BC risk was not on their “radar”, and not something they had ever considered, either because they hadn't been “touched” by it (no BC among friends or family), or because other issues took priority (see Table 4, quote 1). Some women would only be interested in their personal BC risk if it was reassuring, and expressed concern about being at high risk (see Table 4, quote 2)

Unless you've been touched by it, it's a bit head in the sand for a lot of people. Blissful ignorance

2 If I knew I was from a high risk category, and I'm pretty sure I'm not, I reckon I would ﬁnd that so stressful. I would just worry every time there was a pimple.

I think you could scare people when they don't need to be scared, and I think you could also make people feel overconﬁdent that it’s not going to happen 11 I can see some people getting tipped over the edge if they're already an incredibly high risk and have a high anxiety state, have other medical issues, but I think you look at the greater population and you say well, do you avoid giving all these women access to this tool because o fa small number of people who may have a problem?

Lawton-2014

Participants (n = 14) who considered themselves to have poor mathematical skills highlighted a gratitude for, and reliance on, their advisors from the outset: ‘‘Because I was the worst, I was terrible at maths at school, I rely on it’’ (M10.1); ‘‘I absolutely live by that machine; it’s fantastic, it’s been invaluable’’ (M8.1). Indeed, these participants, who were mostly older/retired and from unskilled or semi-skilled occupational groups, questioned how they would have successfully implemented a FIIT regimen without access to this technological support:

‘‘I mean, for example, this morning my blood was 10 and I knew I was having a bowl of quick porridge, that’s 3 units, so I had the six for the three lots of carbs, and then it, my machine said you need 2, 2 more units, so I had 8 this morning, I don’t know how I would have managed to work that out.’’ (M8.1)

Other participants (n = 17) who expressed greater conﬁdence in their mathematical skills described choosing to use

advisors because they saved time and effort: ‘‘it just makes it less work, to be honest’’ (M20.1); ‘‘I’m just lazy with the maths really. I don’t want to be working that stuff out, so if it’s going to do it for me, that’s ﬁne, it’s much easier to let it do it’’ (P23.1). In addition, by virtue of being fast and easy to use, these participants described bolus advisors as facilitating accurate determination of doses when they experienced poor concentration due to hypo or hyperglycaemic excursions. As a consequence, participants worried less about miscalculating doses:

‘‘Cos, like I’ve just said, if I were running 4 points high, I’d be trying to think back, how many units I need and then you start getting ﬂustered, and start trying to, which makes you worse, your sugars are going up, and because your sugars are high you start feeling ratty anyway, and then you start thinking ‘‘Oh, I can’t work this out’’ so you dial too many on then, before you know it, your sugars have dropped.’’

Lugtenberg-2015

Irrelevant alerts for individual users, with varying needs across time •“Well, for example, you don’t wanna see the ‘advice to give up smoking alert’ again, when it’s already clear that it aint gonna happen with this patient. You don’t want to receive that alert over and over again”.

Relevance of alert content for patient (discrepancy between patient’s reason for visit and alert content) “The patient’s reason for visiting that absolutely does not match the content of the alert. If someone visits with his ankle, you don’t want to receive an advice on statins”.

Nelson-2020

Patients also identified setting as important (21 [44%]) in terms of both the health care institution and the patient. “I would have to know that this application was set up by the dermatologist… or [my] medical group,” said one patient, “I wouldn’t want it to be in the hands of a private company.”

Patel-2018-additional file

GP has a difficult time convincing high risk patients in taking medication. This is a regular occurrence and assumes patients wouldn’t be interested so doesn’t bother trying.

GP: this one here [HT], bugger, it’s just a matter of collecting data. That’s how it feels, you see, so I haven’t got any, how shall I say it, enthusiasm about it

Pope-2017

In other cases, the patient refused an option offered and this had to be resolved:

The triage finishes with a disposition of “A&E department- 4 hours” I believe the caller asks if she can take the child somewhere else. The call handler reiterates “I think it’s saying go to A&E because it’s a child” and calls over the supervisor for clarification. […] the call is now put through to a nurse adviser, essentially because it is a “refuse disposition”. The call handler explains to me “it’s a big loop; it will just come back to us. It was and ED 4 hour, but they didn’t want that” (Observation, NHS 111)
