# Supplementary material for: Stakeholder Perspectives of Clinical Artificial Intelligence Implementation: Systematic Review of Qualitative Evidence
Source: J Med Internet Res. 2023 Jan 10;25:e39742. doi: 10.2196/39742 (PMC9875023; doi:10.2196/39742)
Supplement: Multimedia Appendix 3 [file jmir_v25i1e39742_app3.zip › 4. Adopters/4c. Carers/4c. Carers (available, type of input).docx]

**Name:** 4c. Carers (available, type of input)

Collard-2020

‘Just being able to say we’re going to take a walk and we go take a walk. That would be great.’

Parents and children also highlighted the potential benefit of an automated insulin delivery system, especially during and after exercise for glycaemic control. ‘It’s also the “after exercise” effect that you can never be quite sure what’s going to happen and how long it is going to last for. It [would] be useful for that if you could go and do the exercise and not have to have all the extra thinking afterwards.’ (Parent, Focus group.)

Flynn-2015

The value of being given a paper copy of the risk presentations to keep was noted by one relative (it enabled reflection on the consent discussion and provided reassurance that the most appropriate decision had been made): “I was able to just reflect and say okay I’ve done the right thing for my wife” (Relative 3)

Henshall-2019

Patients and carers commented that the DST could provide a useful starting point for discussions with their doctor about medication preferences, as it ranked medications in terms of suitability to their own preferences.

I want to have the greatest chance of success on proven clinical stuff so far … But number one would be the one to start with … If it didn’t work, then try number two. Patient/carer 1

Psychiatrists commented that doctors often make prescribing choices based on efficacy, without sufficiently considering the impact of potential side effects. Conversely, patients/carers stated that a drug’s side effect profile would substantially influence their likelihood of compliance and that this information was crucial. All participants felt that information relating to a patient’s age and comorbidities on drug efficacy, mode and frequency of administration and potential side effect severity should be included within DST. They also felt it should provide a balanced level of information on the most common side effects of each drug listed, to allow patients to decide how acceptable these side effects were.

[Otherwise] you’d be selecting a drug without necessarily thinking about what the other side effects could be … And then you get prescribed one that actually has another side effect that you don’t want at all, but it wasn’t even mentioned. Patient/carer

Soling-2020-supplementary file

then a relative comes by and says, you wrote down something that possibly reduces the effect of the Parkinson medication, we cannot take that. What impression does that make? If then ... that's what it is, if there are a lot of people interfering, that's bullshit. Somebody has to say how it works and then it's okay.” [GP6, p.8
