# Supplementary material for: Stakeholder Perspectives of Clinical Artificial Intelligence Implementation: Systematic Review of Qualitative Evidence
Source: J Med Internet Res. 2023 Jan 10;25:e39742. doi: 10.2196/39742 (PMC9875023; doi:10.2196/39742)
Supplement: Multimedia Appendix 3 [file jmir_v25i1e39742_app3.zip › 4. Adopters/4d. Relationships/4d.1 Patents' relationships with their clinicians.docx]

**Name:** 4d.1 Patents' relationships with their clinicians

Abejirinde-2018

Some health workers were also of the opinion that the device mediated woman-provider relationship by enhancing trust.

“Even here the moment you work with them and they have trust with you, that is all. They believe and they trust whatever you tell them…. And with the box, it has even enhanced our work. They believe and they trust that if we tell them something, it is true because the box has actually said it.”

Abidi-2018

Patients also stressed on preference for direct patient-provider contact:

Sometimes I just want to talk during an appointment with my doctors...maybe I don’t want to talk through an app during this time.

Alagiakrishnan-2016

Some respondents mentioned referencing the alerts during patient education, discouraging specific problem medications (eg, hypnotics), emphasizing the importance of medication compliance, or simply printing linked information for patients to take home

Blease-2019

Technology will never attain a personal relationship with patents. We are essentially a people business. It’s personal relationships that count. [Participant 45]

Bourla-2018

Alteration of the therapeutic relationship

Cameron-2017

many physician participants felt that a decision support tool had the potential to improve consistency of practice amongst physicians and to reassure patients and caregivers that decisions regarding driving capacity were not being made in either an arbitrary or ad hoc fashion. However, in order to gain credibility and buy-in, participants emphasized that the tool would have to be evidence-based, align with established routines, and support the ethos of patient-centered care

Catho-2020

They perceived the CDSS implementation as a potential way to devote resources to other tasks that cannot be replaced by machines. FR_03 (M, resident): “Computer tools are a good way to focus the work on things that computers can never replace, like the human [interaction], the time of consultation. We, doctors, are clearly all overwhelmed.”

Chow-2015

Junior physicians were inclined to accept ARUSC’s recommendations most of the time, but had to override its recommendations when senior colleagues decided on a different antibiotic [J2, J3].

Chrimes-2014

The positive commentary was related to the simplicity of printing the contract and providing a way for the provider and patient to finalize the negotiation and dialogue.

Dikomitis-2015

this in turn directs their attention away from the patient: ‘There’s a dichotomy between the very useful information that’s on the computer, and actually, you know, sort of, looking at the patients, and giving them, you know, proper attention, as they perceive it, you know’. (GP/15)

Goetz-2020

Another concern was that the vPCP would not involve the patient in the decision-making process.

“. . .having that shared discussion as far as these are the positives and negatives ofthis treatment. . .that removes a lot of the personal decision that come into healthcare.” (Fourth year medical student)

Haan-2019

Patients report that personal interaction when receiving information about the results of a scan is important to them; this human contact allows them to safely ask questions and gain mutual understanding of the impact of results and reliability of findings. Patients express their concerns about depersonalized procedures in which patients become numbers. Also, to discuss the results of a scan in a sensitive manner, human dialogue is important

Hallen-2015

In addition to increasing prognostic conﬁdence, physicians valued CPMs as a means of enhancing their prognostic authority. Several physicians reported a lack of perceived authority as a barrier to prognostication, especially in circumstances where a long-standing relationship with a patient or family was absent. Physicians felt that this lack of authority was signiﬁcant and diminished their capacity to inﬂuence decision making and that CPMs could augment their credibility and authority by conﬁrming their prognostic estimates. They viewed CPMs as providing a de facto independent conﬁrmatory opinion in EOL care discussions, especially in situations in which trusting relationships with patients and family members were yet to be developed.

Geriatrician 3: I know some of my colleagues use [CPMs] particularly when they are in contact with a patient whom they don’t know well. It gives some additional support to what they are saying that it is not just my opinion but we have got this tool we can use and it is saying the same thing I am.

Geriatrician 2: I mean if a relationship with people that goes from between 15 and 30 years, to then talk ... is pretty strong, as opposed to the hospitalist who comes in for the ﬁrst visit and has a zero relationship and I think just them giving their opinion about qualitative things is a much more diﬃcult discussion... it’s hard to have these discussions without really having strong quantitative data...

Physicians also saw value in the ability of CPMs to reassure and persuade patients to make medical decisions. Similar to the concept whereby CPMs can increase prognostic authority by providing a de facto independent conﬁrmatory opinion, physicians also viewed CPMs as oﬀering a level of proof that could facilitate patients’ agreement on particular courses of action when clinical circumstances seemed clear:

Cardiologist 3: I think it would be most useful if I felt strongly that a patient ought to have a certain intervention but the patients didn’t feel that way and I really felt like trying to push the patient in certain direction. I could give them some data that said, you know, look, somebody has done this study and these are the outcomes...

Internist 1:

... families think, you know, yeah Granny’s old, but she still lives on her own and she’s kind of getting by ‘cause we’re helping her out. [Families] may have a 5-year plan in their brain and I may be looking at this patient and saying, man, you know, this ain’t 5 years. This is 6– 12 months or something like that and having a way to convince people of that may be very helpful.

Horsfall-2021

the potential loss of human touch (4/33; 12%),

Jackson-2017

One participant raised the possibility of patients losing their relationship with the IBD clinical team by relying too much on electronic care. However, participants agreed that strict inclusion criteria should apply to the intervention such that only stable patients who have already built a relationship with the clinical IBD team would use the intervention.

Jutzi-2020

Another common concern regarding the use of AI was a diminished physician-patient relationship—consultations could become more sterile, with no personal conversation with the physician and less time for questions

The use of AI-based tools might save time that physicians could use for more personal contact to the patient

Lai-2020

AI could also interfere with the physician– patient relationship. In the case of machine learning, for example, the “black box” phenomenon could prevent the doctor from providing clear information to his patient, depending on the degree of the tool’s independence in the final result.

Lennox-Chhugani-2021

Concern about the absence of the human touch in interactions (n=46)

The main concerns that were expressed by the women were: 1. The absence of the ‘human touch’ in the diagnostic process

Liberati-2017

Moreover, as found by previous studies [34, 45], the EHR is perceived has having a negative effect on doctor-patient communication during consultations and seen as a potential obstacle to the development of a relationships of trust between clinicians and patients

Lugtenberg-2015

Doctor-patient communication (too much time spent on the computer during consultation)

“It just takes a lot of time and makes you focus too much on your computer and the patient just does not like that. I can see the patient thinking… while I’m only staring at that stupid screen”.

“I click [on the computer] like there’s no tomorrow, also during patient consultation with the patient next to me. And I sometimes find it disturbing, that I spend so much time on the computer…”.

Miller-2019

interfering with the PCP-patient relationship,

Nelson-2020

Other perceived risks included human loss of social interaction (18 [38%])

Other commonly perceived weaknesses of AI were lack of verbal communication (28[58%]), lack of emotion (20 [42%]) ,and lack of nonverbal communication (19 [40%])

In the realm of verbal communication, patients called attention to the inability of AI to answer follow-up questions, discuss treatment options, and educate and reassure the patient. “People can…be really anxious, sad, fearful, ”one patient commented, “and the app’s not going to be able to sense that.” In the realm of emotion, patients noted AI’s lack of compassion and empathy. One patient expressed, “You can’t write an algorithm to love somebody. ”In the realm of nonverbal communication, patients called attention to AI’s lack of emotion perception, “eye contact,” and “human touch.”

Pannebakker-2019

it facilitated patient–GP communication in the consultation

Pope-2017

In dealing with callers, as in many customer-facing occupations, the call handlers often bore the brunt of callers’ anger and frustration. The nature of healthcare work was that they had to manage anxiety and distress, and could be subject to hostility or abusive language:

Call supervisor 3 reports that a patient also gave her “loads of abuse”. Call taker joins in with conversation, remarking “I took the original call, yes lovely young girl she was! She can’t have been that ill, she was leaning out of the window calling [out], she can’t be that ill” (Observation, NHS 111).

Rapoport-2020

While study guidelines requested that the tool be used with the patient and/or family member present during the assessment, the majority of participants used the tool after the patient encounter. The central reason for this was feeling that it was insensitive to be dealing with a computer rather than focusing entirely on the patient. A family physician commented on using the tool with the patients ‘rather than on them’ [MD03-FP], and a nurse practitioner indicated that it ‘often paved the way for more fruitful discussion’

The one lady that I did take away the licence, I phoned the family member and I said, ‘I’ve gone through the tool and this is what it says’. And he said, ‘Thank God’. [laughs] And I said, ‘I’m going to report her’. And he said, ‘Good. That’s great’. [MD03-FP]

The tool] takes away that it’s me doing it. You can say ‘We do this for all people in your situation’, and … you kind of depersonalize it. Because people think, ‘You don’t like me’. Well, no. I’m recommending you don’t drive because you’re not safe. So, I think the tool is very useful for that. [NP04]

In some cases, the tool indicated ‘report’ but the participant chose not to do so because they felt that the risk to safety was low and due to contextual factors other than cognitive impairment, or they felt that reporting would alienate the patient

Roebroek-2020

Most clinicians indicated that they did not notice significant changes in the therapeutic relation with their patients when using TREAT. However, some clinicians did:

“We think that the traditional treatment relationship between patient and clinician is fundamentally changing, it is becoming more horizontal, not in every aspect but in many. That is where it is supposed to go. I really think TREAT can facilitate this because it increases commitment and a feeling of ownership.” [C4]

Silveira-2019

Due to the scarce time available, some of them attended patients without the CDSS and filled it out later, saving remarks for the next appointment. In the case of one clinician, this choice was due to fear of patient opinion.

...I was concerned that they might find I was distracted or writing something else, like sending messages during the consultation. [Clinician]

Van de velde-2018

Participants mentioned some potentially negative consequences of providing CDS directly to patients, including anxiety, inappropriate management, and the risk that CDS might replace the personal contact with the GP

A patient commented on the limitations of CDS: Technology alone is not enough, a lot has to do with the personal contact between the patient and the physician, and the physician needs to know the patient’s perspective. [Patient, Norway]

Vedanthan-2015

Participant: the relationship now has reduced simply because we concentrate too much in the gadget. The patient likes to talk to you directly but you, you just concentrate in the gadget.

Nurses who were concerned about the device negatively impacting their relationship with patients felt that this barrier could be surmounted with continued use and increased familiarity with the device

Wang-2018

Some GPs believed that CARATV2.0 might be able to assist in negotiations with patients by providing evidence (e.g. stroke risk score) for explanations. However, one GP argued that CARATV2.0 could not help in persuading patients to take certain OACs because the negotiation to persuade or convince patients to take antithrombotic therapy involves managing individualised health expectations rather than only presenting scientific evidence about this form of therapy.
