# Supplementary material for: Stakeholder Perspectives of Clinical Artificial Intelligence Implementation: Systematic Review of Qualitative Evidence
Source: J Med Internet Res. 2023 Jan 10;25:e39742. doi: 10.2196/39742 (PMC9875023; doi:10.2196/39742)
Supplement: Multimedia Appendix 3 [file jmir_v25i1e39742_app3.zip › 4. Adopters/4d. Relationships/4d.2 Users' relationships with tools.docx]

**Name:** 4d.2 Users' relationships with tools

Bourla-2018

False-positive, false-negative

Cai-2019

“If the person you do normally see eye-to-eye with tells you that it’s wrong, then you’ll believe that more.” (P20) To many, an ideal collaborator would be one who shares similar medical points of view as oneself, but who also adds additional insights: “What are they worried about? And I’ll think, was I worried about those areas as well?...If they overlap [with mine] but then add a little bit [of extra information] ...I think that would make me more trusting.” (P15)

This practice of learning the clinical styles of one’s colleagues led pathologists to inquire about

where the algorithm lies on the spectrum of subjectivity, similar to how they might calibrate to their peers: “It’d be interesting to know how you calibrate your eye to a system that you’re going to be using. What are you calling and what is it calling? I know what my friend...will call, and what I call...What would AI call it?...I’m treating it as a peer.” (P18)

As with choosing peers for second opinions, participants expressed a desire for the algorithm

to have similar diagnostic styles to oneself, such as being more liberal or more conservative in assigning higher-severity cancer grades: “Does it have a bias a certain way? I kind of want it to think the way you do...I certainly wouldn’t want it to be completely discordant with what I think are the subtle nuances between 3 and 4.” (P2) Having similar subjective thresholds could be key to developing trust, so that they could later rely on it when uncertain: “If I find every time I call [grade] 4, it calls 4, then like a person, you build up trust in each other. Then next time if I can’t decide, then I would trust the computer. That would build up trust, it’s very important. Let’s say I use it for one week, and 30% of the time I don’t agree, then I cannot trust 4 is 4.” This suggests that the subjective operating points of a model may need to be made transparent or even adjustable by end-users. However, there may exist a tension between the subjective alignment with users needed to establish trust, and the shift in current user biases needed to improve clinical practice.

Participants described how an awareness of the AI’s strengths and weaknesses could support their strategic allocation of attention. Some compared this to the ways in which they currently allocate energy depending on the known expertise and weaknesses of their co-workers: “It’d be like working with a partner. Like I know what my co-workers are strong and weak in...I probably anticipate weaknesses and pitfalls and dedicate more mental energy towards trying to fill in the gaps...I’d develop a working relationship with AI where my awareness is heightened knowing the AI’s weaknesses and dampened with the AI’s strengths. I’d develop a symbiosis with it.” (P18) Several described how they would dedicate less energy toward scenarios where the AI is known to do well (“If I employed AI, I would probably just focus on a few areas here." (P18)), and dedicate more attention and care toward areas that are known weaknesses of the algorithm (“Things to watch out for that it might miss ...you should actually still put it at higher power and look around for yourself." (P20)). These descriptions support prior work on competence articulation in medical teams [45], which enable colleagues to leverage each others’ expertise and complement each other, thereby enhancing collaborative work.

To some participants, the AI’s objective was to be as accurate as possible, independent of its end-user. These participants quickly lost trust in the AI when they observed that it fell short of being a gold standard: “Their grading of4 is wrong, I would say forget about this, give me a clean slide and I’ll make my own decision...I cannot trust this grading at all.” (P16). To others, however, the AI’s role was to merely draw their attention to suspicious regions, given that the pathologist will be the one to make sense of those regions anyway: “It just gives you a big picture of this is the area it thinks is suspicious. You can just look at it and it doesn’t have to be very accurate.” (P14) Some compared the AI’s predictions to the help they typically get from medical residents, who make rough mark-ups of questionable regions for them to review. Rather than expecting pixel-perfect predictions, these pathologists interpreted the AI’s objective as that of drawing attention to worrisome regions, which a human will ultimately interpret. These user expectations surrounding the imperfect nature of annotations are consistent with prior research on how medical experts communicate decision thought processes to one another through lightweight, informal mark-ups [30]. As human–AI decision-making becomes more prevalent, it may become even more crucial to make explicit the extent to which an algorithm’s objective function accounts for the presence of a human collaborator.

Dalton-2020

When prescribers initially saw reports that contained recommendations that were of low relevance, this would have resulted in their devaluation of the perceived benefits of future reports, contributing to decreased engagement with the SENATOR reports and non-implementation of the recommendations.

I think when people have seen these reports and they’ve seen recommendations that are inappropriate or irrelevant, I think that can sort of change their perception of the study and of these SENATOR reports, and it can sort of devalue them as well. So, I think maybe…when they see a report the next time that they pay less attention, that they have less trust in it. [Primary Researcher 7]

Grau-2019

P7, Internal Medicine, female: I remember my very first day as being an intern, the order set popping up and then feeling like I didn’t even know how to use [the EMR] and I didn’t even know how to do anything, and I specifically remember just cancelling out of it so that I could get back to trying to figure everything out, and then never really. And then I think just moving forward, every time it would pop I’ve just gotten accustomed to just trying to get rid of it.

Page-2019

The most common design advice was to start with a small number of well-designed alerts (n= 10, 24%). Respondents elaborated, commenting that: No alert is better than a poorly designed alert. Get alert design right the ﬁrst time. Do not lose the end-user’s trust in alerts.

Patel-2018-additional file

GP: It’s not, it [HT] doesn’t really take any more time or effort; you get to the stage where you can use it without even thinking anymore

Petitgand-2020

This type of feedback was directly reported to the DSS developers during focus groups. In response, adjustments were made to the design of the medical history to better classify patient information and simulate the clinical reasoning of physicians (presentation of pre-existing conditions, chief complaint, etc.). However, adoption rates did not increase significantly. Implementation data collected by the DSS developers showed that the proportion of annotated medical histories remained almost the same (around 30%) before and after these adjustments.

At the start of implementation, several physicians were positively disposed toward

using an AI-based DSS to enhance their diagnostic practice. However, some reported having discovered "errors" in the medical histories. In particular, two physicians reported that reading the medical history led them down the wrong diagnostic path. Had they not questioned the patient again, they would have made a serious clinical error:

But, you know, there are times when it completely took me down the wrong path... Not often... But it happened and it should not happen. It’s like, me... I have zero tolerance. It’s a tool that's supposed to help us... not at the price of losing a patient... But to miss something, something huge, you know... So that's... It didn’t happen often. But it happened, so it cooled my enthusiasm. (Physician 6)

Porter-2018

The impact of the CCDS on paramedic practice was not necessarily maintained over time. In site one in particular, where there were technical problems, many paramedics reported abandoning CCDS within the first few months of the study:

It was just like I was fighting all the time to get [printer] paper or get the password or get it working, I just gave up in the end. (End S1 05)

I used it a couple of times at the beginning, and then towards the end, no. It’s just too slow. (End S1 04)

Rapoport-2020

Alignment of the tool recommendations with clinical assessment I went into it thinking that it was going to be more conservative than I am. I was a little bit worried that, suddenly, I was going to have to tell many more patients to stop driving than was my practice and I didn’t find that. I felt that it was aligning with what I was doing quite well. Just getting some familiarity with it and trust in it that it made sense and resonated with my understanding of patients and the literature was an important learning stage. [MD09-SP]

Yeah, [the recommendations] did [align with my clinical judgement]. I think it helped to work through some of the things that you should be thinking about and it helps to kind of alleviate some of those fears that you have, as far as making that decision to report or not to report. … I sort of went in with a preconceived notion of what the answer would be from the tool and then that basically helped to sort of clarify it. [NP03]

Reynolds-2019

Once we get used to them, it’ll be pretty helpful ... but at ﬁrst, just the learning curve that’s going to be a bit of a pain.”

Nurses (at least 2) report machine is most helpful w/ drips. One nurse used PAC2 in addition to hand calculating doses. Another nurse reports doing the same initially but using the PAC2 in place of hand calculations after having gotten used to using the PAC2

Roebroek-2020

Most clinicians had to become familiar with TREAT and find ways to use it effectively during consultations:

“You really need to work with it [TREAT] a few times because you can get questions for which you were not prepared or reminded of things you might have missed.” [C7]

Wang-2018

Similarly, the senior accredited and hospital-based pharmacists (with ⩾40 years of experience) also stated that CARATV2.0 validated or organised their own decision-making process

Yang-2019

Seasoned physicians shared that their dream DST should play a role similar to mid-level clinicians. They should provide additional context for the seasoned physicians’ decision. The DST could provide additional context and a different perspective to the senior physicians.
