# Supplementary material for: Stakeholder Perspectives of Clinical Artificial Intelligence Implementation: Systematic Review of Qualitative Evidence
Source: J Med Internet Res. 2023 Jan 10;25:e39742. doi: 10.2196/39742 (PMC9875023; doi:10.2196/39742)
Supplement: Multimedia Appendix 3 [file jmir_v25i1e39742_app3.zip › 4. Adopters/4d. Relationships/4d.3 Relationships between health professionals.docx]

**Name:** 4d.3 Relationships between health professionals

Alagiakrishnan-2016

Respondents reported concern about discontinuing medications ordered by other physicians. Some clinicians reported confidence treating aging adults and did not hesitate to take action based on the SMART CDS, but at times, a “crossover of physician responsibilities” was cited

Some physicians felt that the management of specific medications should be deferred to the clinician overseeing the related health problem

Ash-2015

The clinical organizations are especially sensitive about providing CDS that will frustrate physicians by slowing their work with an inordinate number of alerts or reminders.

Baysari-2017

Related to time pressure, junior doctors reported that they felt pressure from more senior doctors to prescribe antimicrobials without gaining approval from the antimicrobial stewardship doctor. Junior doctors recorded inaccurate indications to bypass the approval process and to avoid being reprimanded by consultants. Several junior doctors also reported feeling intimidated by more senior staff. As a junior doctor you do try to game the system and do what you are told to do and the less number of people trying to chase you up, making sure your prescribing is appropriate...makes life easier. (D5) It’s a physician telling you to do it and as a junior doctor you are not going to say well maybe that’s not the correct indication, arrhh whether it is wrong or right…you’ve got to do it regardless…because an expert in the field supposedly has told you to do it. (D7)

Beede-2020

The second beneﬁt was the potential to use the deep learning system’s results to prove their own readings to on-site doctors. Several nurses expressed frustration with their assessments being undervalued or dismissed by physicians, and they were excited about the potential to demonstrate their own expertise to more senior clinicians. As P7 explained, “They don’t believe us.” P11 stated, “It could conﬁrm what we already know.”

Biller-Andorno-2021

It could prompt conversations within the team and support the discussion with the patient. ‘Sometimes colleagues feel a bit offended when you question their judgement. […] With the tool, I could say: ‘Hey, I consulted this tool, it favors a different decision than you do, let’s discuss this together.’’

Clyne-2016

“I think it’s probably easier in this practice because it is single-handed. Ok, you know, it’s not like I’m changing something that one of my colleagues put them on and said to them, you must stay on this or whatever, they all deal with me, for better or worse – I don’t know!” (GP21, intervention practice).

Connell-2019

some consultants suggested impeded learning opportunities: I think it’s important for [Junior Doctors] to understand what the decisions about their patients are. They have to be across the data. And that’s why I prefer getting information from them [...] We were in the position where I was telling the Juniors what the blood results were. It makes me uncomfortable and it makes them uncomfortable. [Respondent 6: Nephrology team]

Second, the digital pathway had an impact on relationships between clinical teams. Several members of the nephrology team were uncomfortable about providing a clinical opinion when not solicited by the clinicians primarily responsible for that patient’s care:

So you might [...] call the team and say “we suggest you give some fluid,” but I don’t think it’s ethical to prescribe it yourself. After all, they might say, “Listen, he has heart failure.” So you can’t intrude. [Respondent 2: Nephrology team]

However, there was an indication among several members of the PARRT team that this concern might be limited to communication between different specialty doctors:

I think the doctors have found it more difficult because in medicine there is this real model of, “[...] I don’t see this patient unless I’m asked to see them”. There’s this formality. [...] Nurses don’t think like that, people are used to us showing up. So it’s been easier for us to think of every patient that we see as our patient, our problem, our sick patients. I think that’s been easier for this team to absorb and deal with. [Respondent 11: PARRT]

This is relevant because the type of information available also changed the professional group with whom this team directly liaised:

[I will] always [speak to] the nursing staff, because you are on their ward. It’s only polite and also you will generally be recommending frequencies of obs; they need to know what’s going on. And someone from the medical team. I would say the app is making me speak to more senior doctors more. [...] I’d be more likely to seek out a consultant and say “by the way, this person has alerted” and show them the app.

Dalton-2020

Prescribers expressed reluctance in making changes that might encroach on other prescribers’ decisions (e.g. the patient’s GP or other hospital specialists).

…you’re not the only person involved in their care so you may be reluctant to stop a medication that somebody else started. [Medical Prescriber 1]

Johansson-Pajala-2017

A common perception was that the RNs became prepared for facing the physician. They could get ideas about the problems they should bring up for discussion, and also present suggestions for the actions they thought should be taken. These involved suspicions of possible ADEs, suggestions for drug adjustments or discontinuation of treatments. Additionally, the preparedness gave them evidence which they used to motivate and even persuade the physicians about speciﬁc actions which they regarded as necessary.

‘I felt that I gained more knowledge and that I might be able to use that when I’m talking to the physician about a drug. This is on the basis that I know how the patient feels and how the patient acts in their daily life’

Johansson-Pajala-2019

More time, for the physicians and us, to have more time together…” (N2)

Liberati-2017

In other cases, obstacles to the uptake of CDSS are associated with the threat posed by the introduction of a new information technology. It is feared, for example, that the EHR would modify existing working practices and alter inter-professional relationships and communication.

Notably, rather than causing disputes over professional boundaries (as in position 2), the CDSS presents an opportunity to foster the type of inter-disciplinary practice that is vital for patient safety; it is suggested, for example, that the system allows nurses to acquire additional control of the appropriateness of prescribing.

Prescribing is the doctors’ role, but […] if we have a doubt about a prescription, we do ask questions […] and most doctors are OK with this, they don’t perceive it as a challenge. […] The CDSS helps because we are not always familiar with the drugs we administer: it is a quick way to double-check that everything looks OK with the prescription. (Nurse, setting C2)

Some participants suggest that the availability of CDSSs may foster interdisciplinary work and promote the use of scientific evidence within clinical communities that have been historically excluded from the debate (such as pharmacists and physiotherapists).

The introduction of the CDSS could have positive cultural and educational effects. It could help us developing a culture of evidence within hospital teams and with the community as well. We could use it… not just individually, but as a tool to get together and discuss complex cases and to monitor some particular patients. (Nurse, setting C2)

Marcolini-2021

There was no habit of measuring the blood pressure sitting, lying down and standing. After we started using the software, it became routine. We do not need to say anything. The nurse technician already measures the blood pressure of all patients that way. [Female, 31-year-old physician, 4.5 years’ experience working as a primary care physician]

Muth-2016

9/10 GPs experienced improved communication with HCAs (‘I certainly talked more with the HCA about one or the other patient … because she wanted to give her feedback’

Patel-2018-additional file

The PM did not know purpose of the intervention or study. GP did not believe she was interested; and PM believes GP did not want PM involved.

Main GP was impressed and learned from young GP.

Shannon-2021

Additionally, the intervention helps improve communication between different levels of the care team. Specifically, the intervention strengthens networks between primary care providers and mental health specialists, and may also improve follow up with patients.

Soling-2020

I have patients where the medication just did not really fit and where I can exchange views with the specialists, who are also named [in the digital tool], where patients are being treated. [FG4, GP_CC, p.5]

Soling-2020-supplementary file

“I think if you participate in such projects, you also have the chance to work better with patients, nursing services, with colleagues or sometimes with hospitals. So that you call back and say, is that really the case? Can't you change one or the other or don't always add the next one? Another specialist and another specialist, the urologist and the cardiologist and the hospital, then again and then the nursing service with a proposal. Well, I think it has an important control function. So, not in a negative sense, but in a very positive sense.”

Vedanthan-2015

Program managers found the experience of their colleagues in the development and implementation of other mHealth programs to be a helpful facilitator. They were able to consult with colleagues in other AMPATH programs using mobile technology to help troubleshoot when technical issues arose.

Wang-2018-Tables

think it (CARATV2.0) is good for pharmacist. It looks good to show this to doctor to say this is my recommendation. You have got something to back it up. So decision-making tool to back it up.

Wickstrom-2020

The participants described how responses from a co worker or consultant on the shared digital platform increased their own skills and knowledge of how to handle similar cases themselves.

They expressed that the DDSS could gather and organize a few involved nurses and physicians in local wound management teams for diagnosis and treatment with continuity, thereby increasing the quality of care:

Fundamentally, I guess it’s about getting a structure and, like, building up a good wound healing clinic here at our primary health care centre. [Participant 4]

chance for all of them to achieve progress in wound management. For example, the participants described how if a nurse did not have medical support from a physician, the nurse found it difficult to dare to try the DDSS. However, when there was medical support from a physician, the nurse felt safe in collaborating, and everything went well.

In addition, participants who responded on the platform expressed great engagement in delivering answers promptly: I feel a lot of engagement—it’s like I want to respond if they write questions, because I see a great benefit there. [Participant 3]

If co workers opposed and questioned continuously, that would be a barrier to engagement:

JM

If you don’t have the working team with you, if you feel like you’re being obstructed, those can be the kinds of things that counteract your engagement. [Participant 9]

They felt engaged by the opportunity to be connected to a specialist in wound management, to get feedback on their work, and to get support when assessing diagnoses and providing the best treatment. Stress and frustration were described to be reduced by knowing that there was someone to consult, who could give support and direct the participants toward the right clinical decision.
