# Supplementary material for: Stakeholder Perspectives of Clinical Artificial Intelligence Implementation: Systematic Review of Qualitative Evidence
Source: J Med Internet Res. 2023 Jan 10;25:e39742. doi: 10.2196/39742 (PMC9875023; doi:10.2196/39742)
Supplement: Multimedia Appendix 3 [file jmir_v25i1e39742_app3.zip › 5. Organisation(s)/5a. Capacity to innovate in general/5a.1 Resources needed to deliver the benefits.docx]

**Name:** 5a.1 Resources needed to deliver the benefits

Abejirinde-2018

Attempts by health workers to incorporate use of B4M while handling a high volume of work in understaffed settings, sometimes resulted in situations where not all women attending ANC were screened with B4M.

Abidi-2018

I love it...but can I do a good job with it? How can I incorporate this within the time restrictions?...at times it may not be conducive to my schedule...a patient gets just 15 minutes with the provider.

Andrews-2017

participants admitted that recent organisational changes and competing priorities meant that these procedures were sometimes neglected, and increased patient numbers in the newly formed service have increased pressure on staffpatient contact time, reducing what is possible during home visits.

it was initially in their [occupational therapists’] remit to always do a mental health screen for every patient, but as we’ve Merged the services and increased the number of patients, [..] it becomes a bit unwieldy to be doing it for every single patient

Participants who worked in frontline staff roles believed that there would be sufficient time to set up and introduce technology to older adult patients.

Ash-2020

Interviewees told us that their organizations were so burdened by responding to other needs such as designation at the time of our study that they were just “struggling to get the basics working.”

The only thing is, about the front desk asking these types of questions, is are they going to have time to ask these types of questions?

It’s like lobster at dinner. And I refuse to crack and the reward is good but I won’t order it in a restaurant because of how much work it takes to get there

So much happening so quickly with our providers, with the new system and with us on ICD10 and with all these different areas you know. I think PCMH, meaningful use, we have to do this too? We have to do that too?

Beede-2020

With patient volume already a burden, nurses were concerned that following the study protocol (including uploading images) would add to their workload and ability to screen all patients arriving each day.

Benda-2020

Multiple participants also mentioned that external rules drive potential responses to the information. One specific barrier was that eligibility criteria for programs such as care management or housing assistance were complex, driven by payers, grants, and other funding sources.

If .. . the funder is only going to give the resource to vulnerable patients for these preventable reasons, then why ask me to identify 100 patients, two of which are going to qualify? Just go ahead and find those two. – EU02 [Challenge]

There was also concern that the HNHC classifier would identify patients for whom there were no available resources.

Worst-case scenario would be you tell me I have a high-risk patient for whom I should be intervening on, but I’m not armed with any additional interventions to give .... Then it feels ... bad to say, ‘Hey, they’re really sick.’ And you’re like, ‘I know that!’ – EU02 [Challenge]

Perceived resource constraints commonly related to social determinants of health, such as housing, food insecurity, or mental health needs.

If you screen for food insecurity and you go, ‘Great, my patients are hungry and they’re going to go to the ER for a sandwich’, unless you can give them a resource, we don’t want to ask.

Clyne-2016

GPs highlighted that current workloads made dedicated reviews for all older patients unfeasible due to time constraints:

“General practice at the moment now, as far as I can see, is getting hit by about 30 % more extra work, due to the economic downturn, so most medical card list have gone up by about 30 %, and that is increasing a huge volume of work, because those patients before,

happened to be in the non-medical card area and they weren’t consulting as much. So they are now consulting, eh, much more frequently so it’svery little time left … if you had to do that every 6 months, to review all those patients. Where would you get the time?” (GP5 control practice).

Dikomitis-2015

Few of our respondents, however, used the calculated PPV tables in this proactive way, reasoning that it was not feasible for large practices to call patients in.

Jacobs-2014

Backload existing data would require significant extra work.

Johansson-Pajala-2019

The physicians need to engage in the process of using the CDSS. They need to take time to audit and assess the quality reports generated. The RNs suggested that the physicians spend enough time in the nursing homes to conduct structured drug reviews. Some of the RNs reported that the physicians' lack oftime and, sometimes, interest prevented them from working with the CDSS as intended. More time also implies that the RNs should have opportunity to bring up questions and discuss any drug-related issues with the physicians.

Lugtenberg-2015

Limited time available

“In daily practice I can’t manage to create time for this. It just doesn’t fit in the regular consultation hours”.

McDermott-2014

In some instances, despite being curious about information within the prompts, GPs felt they did not have enough time to consider them, and therefore did not use them.

"So it was sort of a nice idea but it's just that sort of real pressure on time, thinking you know, I can't go through all of this and it put me off" (P06)

Moullet-2020

the lack of time was a barrier to using these tools systematically.

The majority of physicians explained that a lack of time was a barrier to using the computerised system and to management of nutritional support. The majority of junior physicians reported that they reviewed nutritional goals if they had time during the night shift. Some fellows stated that lack of time during morning medical rounds impeded recalculation of nutritional goals. “Well, it depends on the activity of the unit, if we have time to recalculate at the morning medical round … it is possible that one or two days go by because we can't recalculate the same day.” [JP15] “… the nutrition computerised system, we don't necessarily have time … sometimes at night I look at it because we have more time. Otherwise we don't have much time during the day.” [JP17] “I look at the nutrition section of the computerised system but not systematically enough but it depends a lot on the time available … it is true that when you have a few patients in the unit, you tend to go around more.” [SP1]

Mozaffar-2016

Nursing as a whole has had a very small voice and one of the main reasons behind that and one of the significant issues that [Site F] has as an organization is that there’s a 50 % vacancy rate, so ultimately it’s very difficult to try and engage a workforce that’s very transient in its population and within its workforce… (Site F, Lead Nurse for HIT)

Secondly, funding issues made it difficult for hospitals to recruit the required staff for long-term.

… NHS England is giving money over for a finite amount of time, there’s no money in there for roll out and the [hospitals] have got to think about this going forward because that project team that’s there for that implementation is going to go and unless you get to that point where the organization is mature enough to take that on board and move that forward and has the money to release those people, and we were talking conservatively at what £300,000 for staff going forward. So it’s not just about the IT structure and it’s not just about the project phase… it’s a long road. (Supplier Workshop, Participant 1)

Orchard-2014

Receptionists also had multiple competing tasks, often quite urgent (eg. phone ringing). The study paperwork and consent forms meant the screening process took longer, which would have made it even more difficult for receptionists at busy times.

‘When we were really busy, it was impossible even with 3 receptionists’ (Receptionist 1)

Orchard-2019

GPs and particularly nurses both had relatively little control over their time:

“The nurses’ schedule seems to be so hectic they did what they could…it’s more like a mini emergency room at times so it made it difficult.” (Practice Manager, Practice P).

“[Patients have] their own list of things that we’ve got to get through and then we’ve got a couple of things that we might need to do urgently, so it was often a struggle to find the time to do it.” (GP 2, Practice I).

Pannebakker-2019

However, almost all the GPs expressed concerns that using the melanoma eCDS could lead to

increased or unnecessary referrals, and were worried about the impact on specialist care. At the same time, many acknowledged that more referrals could lead to more diagnoses:

’The danger is if we start referring too many people and they all turn out not to have melanoma, then actually we’ve clogged up the system and it’s more difficult for people who do have to be seen. But the counterargument is it may be that more people need to be seen to be picking up some extra cases.’

Patel-2018-additional file

CAT would stop working occasionally. PM would have to call PEN support. Needed a reminder on using it and how to use it on a regular basis.

Young doctors would likely be proactive and use HT; however due to staff turnover (registrars), use of HT was variable at the practice.

GP: if you're going to go through all this you actually, you've got to be prepared to have a good 10 minute chat with the patient because you actually want to engage them and help them understand where they're at and make a difference and that's the time. So it's not the program time it's actually alright we're going to have a proper chat today…..that's what takes the time. So to just have that chat without this tool would, you know you'd be drawing all sorts of pictures over the paper and the patient might get the point but not really. But with this tool if you've got the time you can really get your message across. Yeah but it's that, sort of that 10 minutes to have a proper chat with the patient that I haven't, yeah.

GP: It's got a big potential to help the practice but you've got to have people willing to use the computers and have the time in the consultation to go through it with the patient and unfortunately that hasn't been the situation in the last six to nine months at xxxx [ACCHS name]

100% staff turnover for AHWs during middle of the trial. Lack of transfer of training and knowledge about HT and study.

Petitgand-2020

medical histories had to be printed and handed to physicians in paper form. These tasks were assigned to nurses and clerks who were already overworked with their regular obligations. As a result, medical histories were often not printed and thus were not provided to physicians.

Petkus-2020-supplementary file

“CDSS needs to be continuously reviewed and updated by a team of experts, including the EPR clinicians, pharmacists, nurses and medical informaticians,

Porter-2018

One impact of the CCDS in some cases was that it may prompt paramedics to spend longer on scene with patients in order to confirm whether they are safe to leave at home. Paramedics reported that they could not predict in advance how long an assessment would take to complete, as that depended on the patient’s history, but it could be well over an hour. Against a backdrop of services under pressure, this presented anxieties for staff about whether the additional on-scene time is acceptable to managers:

I think the actual idea is good, but obviously there are times when management are getting on my case ‘cause obviously I take longer on scene than others would. (End S2 03)

Reynolds-2019

Now, there were a couple of times where I was trying real hard to use it and I would check every med on my MAR with your device and tonight I would not have had time to do that.”

Sharpe-2019

Study neurologists were asked if they considered it feasible as they were currently resourced to provide real time review and response to cEEG for at risk neonates outside of the research study and as part of routine clinical care. None felt that they were adequately resourced to provide this and several stated that they were already under resourced clinically.

Sukums-2015

One provider said: “We do not have enough time to use the computer during patient care, because there are many tasks to be accomplished by the same person” (female nurse midwife).

Urquhart-2018

Some expressed ethical concerns about early identification given that many patients’ and family members’ goals may not be met at present because of a lack of available community-based programmes and services for patients nearing end of life. As one participant stated:

‘I think about the lack of resources though. So if there is this strategy [for early identification] … uhm, what do you do with that when you don’t have the resources in the community in terms of, like, intervening?’

Vedanthan-2015

In addition, transportation to the more remote clinical sites was identified as a barrier to rollout and implementation. However, this barrier was addressed during implementation by the procurement of a dedicated vehicle for the CDM team

Velez-2014

“Last week I was up for 48 hours delivering babies, seeing patients, and then more deliveries. This will be too much. Someone else should do it.”

Watson-2020

Financial concerns varied across institutions. Several AMCs stated that large portions of their work were entirely unfunded, while other interviewees expressed challenges with model development stalling implementation.

Wickstrom-2020

One manager brought 3 smartphones to the wound management team at the beginning of the DDSS introduction; this made the participants feel that introducing this working tool was highly prioritized and important, which positively influenced engagement

The lack of working time and timing were disadvantageous circumstances for participants’ engagement when introducing the DDSS:

One thing that counteracts engagement, is the lack of time, that ... this compassion and participation ... can fail because you don’t have the time to do things in the way that you would always like to. [Participant 5]
