# Supplementary material for: Stakeholder Perspectives of Clinical Artificial Intelligence Implementation: Systematic Review of Qualitative Evidence
Source: J Med Internet Res. 2023 Jan 10;25:e39742. doi: 10.2196/39742 (PMC9875023; doi:10.2196/39742)
Supplement: Multimedia Appendix 3 [file jmir_v25i1e39742_app3.zip › 5. Organisation(s)/5a. Capacity to innovate in general/5a.2 Leadership.docx]

**Name:** 5a.2 Leadership

Abejirinde-2018

In health facilities where workers helped women (re)negotiate their ANC experience and workflow, women better understood the value and benefits of the box and this improved acceptability while diminishing complaints about time delays.

Ash-2015

Within the clinical sites, we found that the term CDS meant different things to different study subjects [29]. Many clinical users were not familiar with the term at all. Those who did know the term described it in either very narrow terms (e.g. alerts and reminders) or broad terms such as guidance provided by the EHR for making clinical decisions. Because our study subjects within the vendor organizations were selected due to their knowledge of CDS, they offered careful and detailed descriptions of what CDS means to them. They universally believe a broad definition that includes population-based reports and analytics in the definition.

Ash-2020

There really is not a template for doing this kind of work. You know, the research is thin on integrated health … There’s not a book that says this is how you do it.

Benda-2020

Our participants reinforced lessons from other HIT implementations that champions in the clinical environment were crucial and would be important for instilling ownership over action within local departments.

So, there needs to be a clinician champion, particularly as you look within specific departments or institutes .... You can’t do this from a complete top down. – OPS10 [Facilitator]

Lastly, participants expressed that dissemination and implementation of the predictive algorithm would be best executed through face-to-face communication and leveraging clinical champions.

It does require pretty much face-to-face communication. Sending an e-mail that this tool is now available ... won’t register. – OPS20 [Facilitator]

Chirambo-2019

The top-level managers indicated that they had welcomed this innovation as an effective tool that could help their officers in decision-making. However, the sustainability of using mHealth apps depended on the HSAs acceptance to use them. The HSAs also stated that they were in favor of using these decision-making tools, and would therefore advocate for the continued use of the eCCM tools.

“This is a wonderful innovation; however, it all depends on whether the HSAs who use it, accept using it.” PSA-12.

Clyne-2016

Longer completion times generally reflected a lack of resources to dedicate to the study within the practice:

“That was difficult, because, the person who manages such things is [practice nurse], who was on sick leave for most of the study. So there was nobody driving the process because [practice nurse] was away, we had only very little nursing cover in her absence, then we were doing tasks that would have been previously done by the nurse, so it was a very busy time.” (GP7, intervention practice).

Dikomitis-2015

However, in many cases, respondents conﬁrmed that only one GP or only the practice manager had watched the WebEx and then cascaded the information to other eRATs-users in the practice. This had consequences for the use and understanding of the tools.

Gillan-2018

A unique element of the discussion in the RTT groups was advocating that they be involved in guiding the introduction of AI, rather than passively accepting new roles or the elimination of jobs. RT05 worded it as a potential ‘turning point for the profession’; needing to create ‘the opportunity to drive that change… to be at the forefront of that… and improving those processes’.

Jackson-2017

Involvement of general practitioners was also suggested to ensure maximal uptake.

Jauk-2021

One senior physician raised concerns about the frequency of use among other physicians:

“I absolutely want to continue with the application. Now the question is how to bring it closer to the users – many don’t know much about it yet.”

Johansson-Pajala-2019

The RNs' perceptions of their own responsibility affected the implementation. Some of the RNs stated that the physicians need to request that RNs use the CDSS for drug monitoring. They argued that the physicians are ultimately responsible for the patients' treatments, so the interest and requirements

must come from them. Other RNs spoke more in terms of “we need to get the physicians involved in the use of the CDSS.” They suggested that the RNs themselves must be interested, committed, and motivated to use the CDSS for drug monitoring.

Interprofessional collaboration is necessary for the successful implementation of a CDSS for drug monitoring. Although this occurs primarily between the RNs and physicians, support from unlicensed personnel is also essential. This was expressed as “the entire chain needs to be synchronized.” The collaboration also extends to management, who, according to the RNs, must be committed and driven in the change process. Collaboration is as much about planning and establishing routines for how and when to use the CDSS. Some RNs suggested that routines need to be established in order to facilitate the implementation process. This lowers the risk that use of the CDSS will be perceived as burdensome.

“The manager acknowledges that we need to do this, we have to set aside time for it” (N1)

Those who know the patients best are the unlicensed personnel, you need support from them too” (N2)

“We have the manager with us as well” (N1) “We are a small stable group of nurses working in the same direction” (N1)

“It probably has to come from the physician, and he decides that he wants to work like this” (N2)

“You need to get the physicians with you, so that the physician is aware of why drug reviews need to be conducted” (N1)

“Physicians do not have the time to carry out drug reviews… it is up to their bosses” (N2)

In addition to making demands, management should provide the proper resources in terms of education and opportunities for continuous training

Lee-2015

There was considerable variation in the extent of radiologists’ involvement in the implementation of the MID. Some radiologists were heavily involved in clinician recruitment and training, whereas others were told of the MID only after its implementation. In general, radiologists expressed a strong desire to be more involved in the actual implementation of CDS at their respective institutions, including educational and outreach efforts directed at referring clinicians. One radiologist stated:

The people that are implementing CDS are actually our colleagues, and so the primary people that have this responsibility are our fellow radiologists.. They can take that information and use that [to decrease unnecessary advanced imaging]. In general, radiologists were supportive of the overall goals of CDS and its potential to improve imaging appropriateness and eliminate the additional step of prior authorization if CDS is found to be effective. Regardless of their actual level of involvement in the MID, most radiologists stated that they would have been active participants in its implementation and in clinician recruitment and educational outreach efforts had they been given the opportunity.

Liberati-2017

A hospital manager and a member of the IT staff both suggest that these resistances can be overcome by communicating the CDSS’s benefits in contextual activities, creating opportunities to experience the system first hand, and involving frontline clinicians in the implementation process (for example, using clinical champions that serve as catalysts for change). Reporting positive implementation experiences and promoting discussion between actual and potential users may also enable bridging the gap between perceptions and actual use.

Marcolini-2021

However, there was a statement of more difficulties among older professionals, who received help from the younger team members:

I started handling the software for patient assistance, as a nurse. I had no difficulties. Nowadays I help other professionals, such as doctors, who are more used to pen and paper. My unit has a doctor with 30 years experience, who had more difficulty adapting

(using the software). As I showed him step by step and all functionalities of the software, he became interested and found it cool When he learnt that he could write patient prescriptions and print it using the software, he was very satisfied. So now, he prints the patient prescription and the exams, correctly. This way, he saves a lot of time. [Female, 37-year-old nurse, 13 years’ experience working as a primary care nurse]

McDermott-2014

This was due to the fact that permission for the study IT company to implement prompts on the practice system could not be given until a staff member who knew about the study confirmed participation. It was often the case that no other members of the practice staff had been informed of the study, therefore delays in this process were common.

“we soon learned that they had absolutely no clue or awareness about the study……the study just ends up running for 18 months if not longer and I think a lot of that frankly was us not being able to get hold of the right person” (P IS 2)

Morgenstern-2021

Many respondents identified barriers to applying AI in public health, such as limited availability of AI expertise and a lack of leadership on it in public health.

Right now, it’s hard to see that drive coming from within public health and […]it’s hard to see it […] coming from the AI community because there’s so many more low-hanging fruits that are […]competing for their attention right now.

Mozaffar-2016

Such a business case enabled both suppliers and adopters to gain an overall understanding of their own as well as each other’s needs and offerings. However, due to shortage of capacity and resources in hospitals, business cases were often developed too quickly and procurements were made without adequate understanding of the problems needing to be addressed, which in turn led to unrealistic timeframes being specified.

…your outline business case should [be] clear… [you need to have] put your business case together, justified the finances, justified the patient safety quality and other issues, got an outline of what you plan to do, issues around the pre-implementation work you don’t need it all process mapped but you need a clear understanding of what’s required, resources, skill mix on their side, not so much on the supplier side but on their side in order to go ahead, and so that sort of readiness-check type approach and an outline business case that should be clear that it should go to market, it shouldn’t go to market until they’ve done all that. (Supplier Workshop, Participant 7)

This forced suppliers to enter into contracts without sufficient prior planning, understanding and agreement from both sides.

…there’s a klaxon going off in my mind because if they’re (hospitals) not prepared to share the outline business case with you… There’s a problem with the contracting process again here, going back to contracting because I think we’re, to ask the question from a supplier point of view you’re boxed into a corner to sign up to an unrealistic project plan… (Supplier Workshop, Participant 8)

The lack of clarity in developing business cases further led to diverse translations of the visions by different stakeholders, ranging from better management of data and processes to achieving a patient-centric health service. Such views resulted in lack of ability to align system configuration with long-term goals.

A high level business case on its own was seen as not allowing detailed execution of steps on the ground. In several cases, we found that neither vendors nor adopting hospitals drew up detailed project plans. This led to inaccurate implementation time calculations and impractical implementation plans. Despite the necessity to have clear and reasonable milestones, some adopting hospitals were not rigorous in defining or enforcing manageable timelines at the early stages of the project.

…the high level planning that went on to support the business case […] was at a very high level, it was blocks of time and it was an estimate based on relatively little information… So the first job I had to do when I got here was to take the high level plan which was effectively dots on an Excel worksheet that were months and break that down to a 500 line Microsoft Project plan with individual tasks and individual responsibilities and durations, start and finish dates and everything else… So once you start to look at the detail for the individual tasks you get a project plan that extends quite a bit further than you first anticipated… (Site E, Senior Project Manager)

Hospitals relative unfamiliarity with many of the CPOE and CDS systems in the UK market added to the issues caused by complexity, leading to limited understanding by adopting organization about the nature of the COPE and CDS products. This lack of awareness about project scale, complexity of implementation process, and systems was identified as another major reason for lack of detailed planning.

…until you know what the product looks like and how it works and how it functions and how long it takes to deploy it you can’t do detailed planning and obviously, and this is not a criticism but the people that procured the product very few of them knew anything about how to deploy an electronic prescribing product… (Site E, Senior Project Manager)

Some suppliers had begun to address these issues by suggesting stepwise implementations: i.e., implementing and going-live with the basic functionalities first and then implementing more complex functionalities (e.g., decision support). They suggested that this would enable hospitals to gain an understanding of what was to be developed, allow them to better plan for their needs and as a result have better project time and resource management.

I think it’s much better from my experience to go live with something which is relatively simple, potentially replicates paper so you roll out this advance functionality be it drug checking, adverse drug reaction forms that kind of stuff after the initial adoption stage has pitted out I guess. (Supplier Workshop, Participant 5)

Further to the above, the lack of detailed planning added to the delays already being experienced. This meant that delays became longer as a result of the yearly patterns of change/patient fluctuation that occurred in the case sites. Winter pressures meant that when a project was delayed by a few months from summer it was unlikely that the site would be able to start its roll-out until around February (hospitals chose to delay roll-out until after the winter busy times as the pressures could lead to complications at go-live), so a delay of two to three months turned into one closer to six months.

A lack of understanding and experience around the system being acquired and its implementation challenges, coupled with reliance upon high level business cases without detailed implementation plans also led to underestimation of resources in hospitals.

We also observed problems with user engagement when trainers were not familiar with healthcare practices and settings.

You do classroom based training and people don’t turn up that’s the problem. I have anecdotes here and tales that when you’ve got some senior clinicians being trained by somebody who’s not from a clinical background they’re switching off… (Site D, HITManager)

So we have postponed by three months the going live on the first two wards in essence because the supplier hasn’t been able to have the product ready for use so you could say we’re a bit frustrated by that… And there are things like […] you can get log outs, so you could get access to certain patients blocked if certain events happen and that’s quite tricky and is taking time to get resolved. Their new version of software that is going to come will make that a much smoother process but that isn’t yet available, it should be available before we rollout further. (Site C, Clinical Effectiveness and Medicines Manager)

Orchard-2019

The GP champion provided leadership and increased motivation, as well as reinforcing the idea of practice-level participation:

“A couple of GPs really took it on, while a couple of them found it a burden. [A GP champion] spoke to them and said ‘we really need to be doing this’.” (Nurse, Practice A).

Nurses also performed better when there was a GP champion as well as a nursing team leader providing leadership with screening:

patients are treated according to guideline, increase overall motivation and quality Practices appreciated receiving regular screening data reports, including the number of people with AF, the number screened by each staff member and the proportion of AF patients treated according to guideline. In many cases, GPs had never seen their practice data presented in that way. In some cases, it led to quality improvement, e.g. review of management for AF patients who were not previously treated according to guideline.

“From a practicing GP’s point ofview, you need to keep an eye on these people [patients not eligible for OAC at the time ofAF diagnosis] because as time goes by, they can drift into the category that probably does benefit from anticoagulant.” (GP, Practice D).

“When I saw the data…my first reaction was that’s actually a really good study.” (Practice Manager, Practice I).

These data reports often increased motivation, and facilitated internal competition:

“Feedback’s always great and timely feedback is particularly important. It is lovely to have been able to receive timely feedback each month, and we have enjoyed the friendly competition within our team!” (GP 2, Practice C).

“The nurses got really competitive because [the GP champion] kept telling them who’d done the most.” (Practice Manager, Practice G).

“Just on the reporting...We’re very competitive so I love the fact that I’m leading the nursing team. Our nurse

“We work together and we just say ‘oh that’s right we do that’, so usually we can work through [problems].” (Nurse, Practice P).

“I don’t feel motivated to do it because nobody else was interested.” (Nurse, Practice N).

Interestingly, there were still a substantial number of

GPs in each practice who either did not participate at all, or only to a very limited degree, in screening.

“A few nurses and one ofthe doctors really ran with it…. we tend to find in a larger practice we get varied levels ofengagement.” (Practice Manager, Practice G).

Orchard et al. BMC Family Practice (2019) 20:170 Page 8 of 13

who has just come back from maternity leave is keen to see the next round ofdata so she can see her name up there” (Nurse, Practice C).

Page-2019

Respondents also shared a common insight that governance groups are often reluctant to endorse the removal of an alert once it has been implemented, even if users are dissatisﬁed.

Pannebakker-2019

No GPs recollected any practice discussions about whether they should implement the melanoma eCDS, or how its use would be supported:

‘It just appeared in EMIS’ (M, >51 years).

Most GPs discussed a lack of clear implementation strategies for guidelines and IT tools in their practice. Therefore, few felt that they had been made aware of it:

’It may be that we [partners] just decide by email . . . Now it might probably go to the IT hub to decide on whether we should use it. But on the whole when these things arrive I think they tend to just get installed, especially something like templates which don’t force itself on you. You can still choose to make use of it. But the problem I had with that, we weren’t made aware of it.’ (M, 40 years)

Patel-2018-additional file

The PM is the main driver in helping coordinate implementation of intervention. PM is familiar with e-health and its benefits of improving patient care through data being linked with various e-health tools.

PM is familiar with the intricacies of all the GPs and staff work/habits and use of technology.

PM gives GP reassurance that he is performing well in screening his patients using HT

GP given the capability of the knowledge and reasoning behind the tool and its benefit by a ‘champion’ via webinar. This motivated GP and gave him confidence to use two components of HT: prompt (traffic light) and patient risk communication.

Main GP was the main driver of implementation of the intervention at the practice. GP has the knowledge and skills to be a ‘champion’ of the intervention for his staff and patients. However time and resource constraints prevented him from using it regularly.

PM is not interested in any additional work besides what has been delegated to her due to time constraints.

Main GP is the sole user of all components of intervention. Due to time constraints, he is unable to sustain long term use of HT beyond the study. He needs more external incentives to get others at practice involved.

Sukums-2015

Throughout the study, missing leadership and management support from facility managers or district ofﬁcials and inadequate peer cooperation and teamwork among providers were perceived to hinder system use. This was also expressed during interviews as one provider said “There is a lack of cooperation among providers in our health centre as you ﬁnd some clients with incomplete information in the CDSS” (female nurse midwife). Another provider said that “Some health workers are not regularly using the CDSS during patient care, which demoralizes other providers who are using the system” (female medical attendant).

Sun-2019

One IT firm manager highlights how Chinese firms lack a strategy plan for AI development: “From a tactical point of view, [Chinese firms] have good teams doing something [AI products]. But it is not from a strategy level” [3IT02].

Tsang-2021

When champion roles became established, the dashboard had increased usage, allowing potential to improve patient care. Without a championing role, users viewed it as delegated work, which lost momentum and had poor sustainability

“Until you’ve used it yourself you don’t understand the value of it, and it’s like, trying to get everyone on the same page.” [P8, pharmacist]

Vedanthan-2015

However, the clinical mentors did not use the tablets during site visits and instead managed patients with paper forms. Nurses reported that having mentors who did not use DESIRE hampered their ability to learn to use the tablet. Nurses requested that their clinical mentors use DESIRE during their mentorship visits.

In addition to using the tablet to manage hypertension, they are required to submit monthly reports on paper to the Kenyan Ministry of Health . The administrative obligation of reporting to both the CDM project and Ministry of Health was referred to by one nurse as “double work.”

However, trainings were identified as a major source of motivation for the participants. Nurses requested continual trainings with DESIRE and preferred using mock patients to simulate use instead of theoretical instruction. Nurses also endorsed peer learning as a critical component of learning to use DESIRE.

Participant:... there are those who are sharp with the tablet they can show some of us things...So maybe during break you find someone they explain to you

Wickstrom-2020

Leadership awareness and understanding of the introduction colored and influenced the participants’ engagement. The participants considered it important for the leadership in their own organization to be supportive, enthusiastic, and willing to arrange technical resources: The manager, she’s very positive about this as well, and of course that makes it easier when you’re doing something that involves the entire team and so on. [Participant 10]

The participants expressed that leadership should show confidence in the ability of engaged employees to plan and run the introduction of new eHealth solutions. The participants experienced a negative impact on engagement if the idea of introducing eHealth solutions was top to bottom:

It can come from the top, from the administration ... where we don’t really, like, see the needs, and then it won’t be something ... that also counteracts the engagement. [Participant 2]
