# Supplementary material for: Stakeholder Perspectives of Clinical Artificial Intelligence Implementation: Systematic Review of Qualitative Evidence
Source: J Med Internet Res. 2023 Jan 10;25:e39742. doi: 10.2196/39742 (PMC9875023; doi:10.2196/39742)
Supplement: Multimedia Appendix 3 [file jmir_v25i1e39742_app3.zip › 5. Organisation(s)/5b. Readiness for this technology/5b.1 Pressure to find a way of improving things.docx]

**Name:** 5b.1 Pressure to find a way of improving things

Abejirinde-2018

Interestingly, while ANC attendees in the district hospital (facility A) were particularly grateful to B4M for saving time, their peers in the health centres did not make this association. Contrarily, up to two women from health centres B, C and D associated the use of B4M with a time delay. Observations revealed that the reported time saving benefits of B4M from respondents in facility A was connected to women otherwise having to spend up to 2 hours at the hospital laboratory

“It has helped to make her efficient. It is because the things we could have gone to the lab to do that requires so much time, this one is faster and easier.”

Blease-2019

Interestingly, many participants chose not to interpret the question as directly asking about the impact of AI on the future of primary care, and instead, commented on the growing pressures on the GP workforce, including the risks that this was believed to pose to professionals and patients: The only reason that I'm not burned out is that I reduced my workload and traded money for sanity. [Participant 280]

I changed job due to stress as where I worked I had an unsafe workload [Participant 608] I hate the current stress due to understaffing which is so dangerous [Participant 516]

Risk-taking is not admired or valued yet without it – or AI – general practice will be destroyed. [Participant 464] Technology and non-medical clinicians can replace GPs easily. My burnout is because of my frustration with colleagues and their Luddite working practices. [Participant 495]

Goetz-2020

They noted that using a virtual PCP would result in less time being spent in waiting rooms or traveling to clinics or hospitals, saving time.

“I don’t wanna waste the time to go wait in the waiting room. . . whereas I could just sit home in my pajamas and talk to someone for 5 minutes–in and out.” (First year medical student)

Jackson-2017

Swollen wait lists – 6 months; cost per patient is enormous; frustration; long wait times et cetera; they’re really interested in eHealth resources’.

Morgenstern-2021

Beyond improving existing disease surveillance systems, it was thought that AI might allow leapfrogging in places lacking traditional infrastructure. In so doing, AI could enable earlier and more effective control of burgeoning epidemics. … there [are] a lot of places in the world where there is absolutely no public health infrastructure whatsoever and when things kick off there, that’s where you start to get your […] Ebola outbreaks or your Zika virus outbreaks. […] I picture [AI] as being […] an easy sort of tractable way to get surveillance into places where spill over events, where emerging infections are likely to kind of pop up and where they would otherwise generally go missed due to a lack of laboratories and infrastructure.

Nelson-2020

Increased healthcare access was multifaceted, deriving from gains in labor efficiency and time for physician-patient interaction, remote diagnosis, and unburdening of the health care system. One patient noted that AI “could reach people who don't have great access to health care but may have an iPhone.

Patel-2018-additional file

Further, he believes he a high performing site and didn’t need to change much after initial training from implementation team.

Porter-2018

They seemed to be receptive to the new care model in part because of their frustrations with existing practice:

I think …the majority of us are so frustrated with the system that we just want an alternative somewhere. Pre S1 FG1

Velez-2014

End of the month is so hectic for us. If this will help, I will use it.”
