# Supplementary material for: Stakeholder Perspectives of Clinical Artificial Intelligence Implementation: Systematic Review of Qualitative Evidence
Source: J Med Internet Res. 2023 Jan 10;25:e39742. doi: 10.2196/39742 (PMC9875023; doi:10.2196/39742)
Supplement: Multimedia Appendix 3 [file jmir_v25i1e39742_app3.zip › 5. Organisation(s)/5b. Readiness for this technology/5b.2 Suitability of hosts' premises and technology.docx]

**Name:** 5b.2 Suitability of hosts' premises and technology

Abejirinde-2018

In two of the three facilities where B4M use was observed, the physical positioning of the device relative to the woman and B4Muser prohibited (in the case of opposite seating) or was suboptimal for (e.g. screen not within field of vision) visual engagement by the woman.

Ash-2015

A content vendor representative noted: “I don’t care if it’s a community [hospital] or large [hospital], they don’t have the resources or the bandwidth to do this [CDS]”.

Beede-2020

The setting and locations where eye screenings took place were also highly varied across clinics. Only two clinics had a dedicated screening room that could be darkened to ensure patients’ pupils were large enough to take a high-quality fundus photo. In other clinics, eye screening took place in the nurses’ ofﬁces, or where additional patients received a foot sensitivity screening or nutritional counseling. As a result, the lights were rarely turned off in these settings while capturing a fundus photo, even when a ﬂuorescent light was situated directly above the camera. We were interested to see how these real-world conditions would affect our model performance.

Out of 1838 images that were put through the system (in the ﬁrst six months of usage), 393 (21%) didn’t meet the system’s high standards for grading. Through our observations and interviews, we found that low quality images were caused by fundus photos being taken in a non-darkened environment, as observed in our pre-deployment ﬁndings, or from a camera that needed repair.

However, these solutions are often difﬁcult to implement in practice. For instance, turning a light off is difﬁcult when the eye screening takes place in the same room where another patient is discussing their results with a nurse, receiving a foot sensitivity screening, or receiving nutritional counseling. Waiting 60 seconds before imaging the second eye is nearly impossible when a nurse has 150 patients in the queue waiting to be screened.

In one clinic, the internet went out for a period of two hours during eye screening, reducing the number of patients screened from 200 to only 100. Patients like the instant results but the internet is slow and patients complain. They’ve been waiting here since 6 a.m. and for the ﬁrst two hours we could only screen 10 patients. -P8

Chirambo-2019

We established that there are some areas within northern Malawi where there is poor network coverage, so much so that it becomes difficult for the HSA to send data to the server.

“After doing a child’s assessment, it becomes difficult for me to synchronize data because we don’t have network in this area.” PSA-7

“Network is a challenge. This place doesn’t have both TNM and Airtel networks; as such I have to move for about 2kms for me to find network to synchronize data.” PSA-8

Cresswell-2019

Despite the overall positive attitudes among participants, we also observed concerns that expectations may not necessarily match system functionality. For example, many clinicians discussed a more general problem of information overload and the difficulty of navigating different sources of information. One GP stated that she often had open 10 tabs on her computer at a time, while another mentioned going in and out of different systems, and several stated carrying folders of paper-based records

Johansson-Pajala-2019

The aversion could also involve a general reluctance to use computers. Another impeding factor is the workplace itself, in which there is a need for more than one computer and monitor

Mozaffar-2016

Implementations were also delayed due to lack of appropriate infrastructures in hospitals.

… Clearly you can’t go live with such a thing without having the appropriate infrastructure in place which was a major reason for the delays at the beginning. (Site D, Consultant)

… probably took three months for us to do our work and then maybe another month, month and a half to get machines ready because we identified that if we were going to go down this whole Terminal Server 2008 route machines had to be upgraded to the latest service pack and there was a few other little fixes that had to be done. Like if you’ve got Word open but you haven’t got it in full screen mode you can click it and drag it round your screen and what we found was if the machine weren’t service pack if they had something else if you dragged it would make like a drag mark on the screen and it would leave trace marks everywhere so we had to do like this prep work, again it was to make the end user experience better. (Site A, Implementation Team)

Muth-2016

GPs reported in interviews that the ‘poor’ rating was mainly due to a lack of connectivity with their practice software systems and the amount of time required

Orchard-2014

Nurses also performed the screening in a treatment room, which was more private and clinically focused than the waiting room.

Patel-2018-additional file

She was persistent in trying to use HT at different periods of the trial. However never could overcome technical issues.

Reynolds-2019

“The only thing I see is like availability because like if we each had one and I have it in my pocket then probably it would be just as easy to pull out as my phone but if I’m having to go get it because somebody else is using it probably in a lot of situations we don’t have the time to do that.”

Silveira-2019

Barriers to CDSS use were also identified. Low computerization of the primary health centers was a major limitation for the CDSS implementation. There was a perceptible demand for communication technologies in primary health system and much of the demand presented by physicians was related to the need to register and organize patient records and clinical data.

Although TeleHAS was developed to be fully integrated into primary health care routine, its use as part of clinician workflow must improve to avoid work duplication. This was not possible at that time due to the fact that all primary care units, just like the majority of primary care units in Brazil, still use paper-based patient records.

Sukums-2015

In the structured surveys, unreliable power supply was perceived as one of the major hurdles limiting the eCDSS use.

Minor computer problems (e.g. mouse or touchpad not working) were addressed offsite via phone calls or onsite during site visits. The absence of Internet services hindered online support, regular operating system and antivirus program updates, however the eCDSS software was updated regularly by the IT support team

The single computer with the eCDSS was to be used by all providers for ANC and delivery care and also for self-learning and exercises. In some cases concurrent activities affected the use of the eCDSS as reported by one of the users “There is only one computer in the facility so when there is a woman in the maternity ward while ANC is on-going, it is difﬁcult to use the CDSS (in both sections simultaneously)” (female nurse midwife).

It was also observed that towards the end of our study, the system performance was decreasing due to the growing number of patient records in its memory.

Vedanthan-2015

The most frequently cited barrier to implementation of DESIRE was “Cellular Network Issues”. At the outset of the project, the implementation team discovered that several of the proposed implementation sites did not have reliable wireless data coverage.

Various administrative requirements and regulations involved with the process of tablet procurement led to delays that resulted in a substantial time lag between training and implementation. This led to the need for repeat training and may have also affected morale of the dispensary nurses.

Watson-2020

The technology platform needed to execute and maintain PM and ML models have not been well developed in health care. As one informant explained:

I think that the key challenges in the implementation phase are integration with what will inevitably be legacy technology in an enterprise setting. Technology moves generally at a slower pace than sort of cutting edge, modern tools, and machine learning in particular is one that leverages the most modern tools. Integrating these modern tools with existing frameworks can pose interesting challenges.
