# Supplementary material for: Stakeholder Perspectives of Clinical Artificial Intelligence Implementation: Systematic Review of Qualitative Evidence
Source: J Med Internet Res. 2023 Jan 10;25:e39742. doi: 10.2196/39742 (PMC9875023; doi:10.2196/39742)
Supplement: Multimedia Appendix 3 [file jmir_v25i1e39742_app3.zip › 5. Organisation(s)/5c. Nature of adoption or funding decision/5c. Nature of adoption or funding decision.docx]

**Name:** 5c. Nature of adoption or funding decision

Ash-2015

Interoperability between EHR and content vendor products was described by interviewees as necessary but challenging. A content vendor representative noted the need for collaborating: “But at times you really do need to come together to say, you know, we are doing this together for a site”.

Ash-2020

We were told that, because of the team orientation of PCMH activities, the CDS should target the team, not just the provider.

Clyne-2016

A number of GPs highlighted that focusing on a select number of high-risk or “cardinal PIPs” would make the process more manageable:

“I think that if you keep it simple, and maybe in a structured way if you could layer it, so that you know, for 2012 we are focusing on these five issues and in 2013 we’re focusing on these, you know. There would be a little bit of slippage with last year’s issues, but over time you would introduce better prescribing.” (GP13, intervention practice).

Dikomitis-2015

As aforementioned, participants felt that secondary-care practitioners should be made aware that eRATs were being used in primary care, and that they were informed the tools were evidence-based:

‘The biggest challenge [for a general roll-out of the eRATs] is of course the extra pressure, I think, on secondary care (…)I think you would have to liaise with secondary care, and maybe, there may well be implications for the workload, particularly for secondary care’. (GP

Mozaffar-2016

so the question is not so much what’s there now… but it’s also about what’s there now and how it’s going to align over a longer period of time with a whole load of other changes coming through, so does the procurement process take that into account, the health service procurement process or is it just a single one off purchase that they do with it? (Business Case Workshop, Participant 10)

Also, in some cases, plans were highly aspirational and did not present the reality of everyday care provision. This lack of alignment between “must-haves” and peripheral functions (or “want-haves”) led to higher costs of implementations and possibly contributed to suppliers failing to meet the original requirements.

It’s that incremental process isn’t it? There are certain [features] which are very much aspirational, there are certain ones which are must haves and there are certain ones that are a bridge between those two ends of the spectrum and you need to be able to migrate from the must haves into the aspirational wants if they’re still relevant… So if you allow people to specify all of their aspirational wants, their wish list then any supplier will eventually turn round and say yeah we can do all of that, this is how much it will cost you, at which point most NHS organizations will turn round and go we can’t have that one… (Business Case Workshop, Participant 2)

This was a major issue especially in CPOE/CDS products, which were new on the UK market due to differences between the systems functionalities and the needs of UK hospitals. As a result, suppliers had to go through complex cycles of market analysis, development and implementation at once. Also, joint procurements [51] led to further delays as a large number of adaptations in initial implementations were taking place all at the same time and suppliers had to deal with various requirements at once.

Two case study hospitals were part of the NPfIT geographical clusters (North West/West Midlands, North East, East of England/East Midlands, London, Southern) programme [51]. This meant that a single contract with a local service provider (LSP) was signed for a joint development of information infrastructure, and as a result the supplier of CPOE/CDS was subcontracted by the LSP. In these cases, the National Programme created delays because of the structure of the contracts which meant that change requests could not go directly to the supplier and had to instead go first through the LSP. This arrangement added bureaucratic layers and thus slowed down the pace of implementation in these two sites. The contract also defined staffing levels and resource management strategies, which led to further implementation delays.

[The contract] constrains or limits what the companies can respond to because they’re constrained by a fairly large contract and constraints around that so it defines both the staffing, the product and the services that you can get so you get a standard approach rather than a customized approach if you went directly. (Site F, Change Manager)

Pope-2017

The problem with implementing digital technologies is that all too often, we fail to recognise or support the human effort necessary to bring them into use and keep them in use. The

Santillo-2019

Participants in Stakeholder Workshop 2 expressed concerns about how the implementation team would be trained and updated about new developments in the long term.
