# Supplementary material for: Stakeholder Perspectives of Clinical Artificial Intelligence Implementation: Systematic Review of Qualitative Evidence
Source: J Med Internet Res. 2023 Jan 10;25:e39742. doi: 10.2196/39742 (PMC9875023; doi:10.2196/39742)
Supplement: Multimedia Appendix 3 [file jmir_v25i1e39742_app3.zip › 5. Organisation(s)/5d. Extent of change needed to organisational routine/5d.1 Fitting the tool with current practices.docx]

**Name:** 5d.1 Fitting the tool with current practices

Adams-2020

Participants expressed frustration with incidental and indeterminate ﬁndings requiring follow-up imaging if a deﬁnitive diagnosis could not be achieved based on initial imaging

Some participants also noted the importance of evaluating downstream impacts ofAI on quality of life and mental health, referencing long intervals between follow-up imaging of indeterminate ﬁndings as particularly anxiety provoking.

Beede-2020

With the addition of the deep learning system, patients generally followed the same journey through the clinic as normal, with the exception of now being able to receive an immediate determination of whether or not a referral is needed

As the system provides an immediate referral recommendation, nurses knew that they may need to convince a patient to visit a specialist, depending upon the results. This was a large change from their previous workﬂow, where results may not be available for up to 10 weeks, long after a patient has left the clinic.

In addition, clinics in Patham Thani were not using dilation drops on patients, which could have aided in capturing a quality image

Chrimes-2014

I am not sure how this interface [will work] with my work back and forth with the patient. Comment is fine, but if I can toggle well afterwards, this is problem. Plus, doc flowsheets are not something I usually work with.

Dalton-2020

Even though prescribers may have intended on reviewing or implementing the recommendations, they may have simply forgot about the intervention as it was not ubiquitous for all older patients within the hospital sites.

…it’s all about reminders. I think people are well intentioned, I think they just forget. [Medical Prescriber 11]

de Watteville-2021

Giving insulin adaptation advices is more logical than nutrition adaptation. Decreasing nutrition debit to correct blood sugar is the opposite of their usual practices.”

Jacobs-2014

Changes in workflow are a barrier.

systems already in place, comfort level with current workflow, computer giving a suggestion that physician may not agree with

Lai-2020

Thus, the research said that before integrating AI into medical practice, it would be important to ask ourselves what can be transferred, to identify repetitive tasks that AI can do without risking the loss of skills by the physicians. Therefore, the physician interviews often highlighted the need to have AI tools which would fit in with practice, like any other tool, so that healthcare professionals could use them.

Liberati-2015

[where technologies have reached a good level of integration with the practice, and the electronic medical record it is an accepted work tool, insertion of the SSDC appears to be a natural and little passage burdensome, as if to imply that the bulk of the work has already been done]

Rapoport-2020

In contrast, nurse practitioners highlighted the goodness-of-fit between the tool and their established workflow and patterns of practice. Longer patient visits and the fact that nurse practitioners mostly worked as part of multidisciplinary teams were two factors that were felt to encourage easy incorporation of the tool.

Shannon-2021

“Everything, the whole process has to adapt. From training of doctors, from the structural processes, infrastructure…It has not been so easy in the operation or in the day to day of the patient.”

Torenholt-2021

‘It’s not much different from what we already do’ a physician and member of the clinical coordination group said, referring to standardised procedures and guidelines for interviewing and triaging patients. The algorithmic sorting of patient data is seen as a ‘natural’ prolongation of existing guidelines and best practices, thus expressing a logic of continuation.

As representing standardised care, the algorithm is established as good clinical practice. The example demonstrates how in clinical practice the algorithm is considered a natural extension of what the physician normally does when applying other standardised procedures.

Trinkley-2019

Clinicians repeatedly reported the alerts were “one more thing to get through” to complete a task, reporting interruptive alerts to be a barrier to completing their tasks. One clinician stated ‘…[the alert] not only disrupts your flow but it actually paralyzes you, that I think is the worst of all’

They want ‘a gentle reminder that doesn’t actually interrupt you’, or ‘just sort of there and available’. Many clinicians desired CDS they could access in a way that fits into their workflow

Vedanthan-2015

In one instance, a nurse stated that she had encountered a recommendation with which she disagreed. She was uncertain how to proceed when her clinical judgment had conflicted with the clinical decision support.
