# Supplementary material for: Stakeholder Perspectives of Clinical Artificial Intelligence Implementation: Systematic Review of Qualitative Evidence
Source: J Med Internet Res. 2023 Jan 10;25:e39742. doi: 10.2196/39742 (PMC9875023; doi:10.2196/39742)
Supplement: Multimedia Appendix 3 [file jmir_v25i1e39742_app3.zip › 5. Organisation(s)/5d. Extent of change needed to organisational routine/5d.2 Change to intensity of work for staff.docx]

**Name:** 5d.2 Change to intensity of work for staff

Abejirinde-2018

Prior to the intervention, diagnostic screening was not frequently conducted at the point of care and now constituted an additional task in the ANC workflow, necessitating more time. For this reason, a drawback on the perceived benefits of B4M use was that the ANC process took longer time, which made women impatient

Abidi-2018

PCPs also stated that including DWISE in their practice might result in additional work for them

Ash-2020

So, if you, you know, start asking these questions in addition to what they already do, it’s more clicks. That’s a factor

Catho-2020

CDSSs are considered time-consuming On the other hand, some participants, at all levels of seniority, described CDSSs as tools that can reduce time spent with patients. Computer facilities, in particular, EHR, were described as tools requiring additional work to enter data, ﬁll in boxes and understand how the system works. Digital tools were also perceived as increasingly numerous and overwhelming in the physician’s daily routine

GE_06 (M, resident): “If the application is not worked on upstream and if it is not ergonomic, it is a disaster, it is experienced as a real suﬀering by prescribers, by the accumulation of tasks”.

GE_02 (M, senior physician):“The most important reticence is the extra work, if it’s more complicated, once again to use the tool than not to use it, well we’re not going to use it”.

GE_04 (M, fellow): “Because if we have to enter the data ourselves, it’s a waste of time

Clyne-2016

“So I didn’t do it with the patients but what I did was, I think you saw from the patient records, I highlighted the notes on it, and I’d have put tags on charts when I found, yeah that needs to be done, to be addressed with their next prescription. (GP21, intervention practice).

Connell-2019

The provision of results and real-time clinical alerts and team communication via mobile phones introduced workload for clinicians in a new modality. Overall, experienced clinicians were able to integrate AKI alerts into their existing duties, discriminating between high- and low-priority cases and using this information to adjust their current priorities:

I would intermittently [...] check it, like I would [...] check emails, [...] check it every hour or so, something like that. And within 5 minutes or so I could easily flick through the alerts and [...] identify which ones I needed to see. [...] I felt it was very easy to use, I think some people when they were trying to use it would try and respond immediately to every alert. I wouldn’t personally, I didn’t think that was the best way to do it. Intermittently checking it throughout the day, I managed to keep on top of things. [Respondent 9: Nephrology team]

Some respondents from the nephrology team pointed out that although important information was now more readily available, this created additional anxiety because it was not clear who was primarily responsible for delivering timely care. Once again, the need to expand the responsive workforce was suggested as a way to mitigate the workload-associated stress:

...as Renal [Registrars], [...] you are always now, in the back of your head, thinking “I’ve got this other job to do.” And I think it does create... not anxiety that keeps you up at night... But it’s another anxiety when you already have enough anxiety! So I think even if it was available in the hands of more people, or we were a bit clearer that during times of people being unwell who are your own patients, you shouldn’t prioritise Streams people because they are under another team, then that’s fine. That’s one way of dealing with it. [Respondent 3: Nephrology team]

Gance-Cleveland-2019

a fair number of concerns and a significant amount of resistance on the part of the support staff when they were asked to add additional tasks to their current workflow.

Jauk-2021

The common impression for the perceived ease of use was highly positive. The expert group appreciated that there was no need of additional data entry and that the prediction was available within few seconds in the user interface of the HIS.

Keogh-2019

However, their main concern was the need to enter the same data twice; once to draw up the pedigree using practice software; and once to generate a risk assessment from iPrevent (Table 5, Quote 9).

I think if we've had our data management staff go through and spend the time to … draw up a pedigree in Progeny [clinic software] from the information the woman's provided, and then we have to put the information into a different program as well.

Knoble-2015

When asked if they would use it more if they could by-pass

some of the mandatory steps to save time such as patient demographic data and vital signs, they indicated this would encourage them to use it more.

Liberati-2015

[the discrepancy between the flows of action e thought of clinicians and modes of operation of the system

"In the beginning, technology doesn't buy me time, makes me lose it! If I have an alert and a series of inputs related to my practice I expect that I will need to time to process it. If I agree to follow it, I follow it, if I don't follow it I will have to report it. Work becomes therefore more complex. It can get interesting for those who use it and safer for the patient, but it becomes more complex and takes more time ». (Intermediate physician, setting A)]

Lugtenberg-2015

Too much additional work required

•“These systems, the way they’re currently introduced, just take too much time. And then I deliberately choose not to use them”.

•“It’s a lot of extra work! It has almost become a task of its own. With all the items you have to fill out”.

Marcolini-2021

Finally, each software screen was projected and we discussed which variables they felt could be removed. All participants were consistent to affirm that all variables were very useful, except for patient admission, in which they had to fill in the date of admission and patient discharge, as well as the reason for admission. They considered these variables were not necessary, and their work could be reduced if they could be removed.

Orchard-2019

More than three-quarters of respondents indicated that time was the main barrier to AF screening in general practice. This was the case even for GPs and nurses who were

generally very motivated. Importantly, screening was always done as an ‘adjunct’ task – that is, it was not the main reason patients were at the practice – and therefore, it added time to the consultation. Beyond a certain point, performing screening risked ‘hijacking the consultation’:

“It was something to be done on quieter days, not horrendous clinical days… when we were running behind time, you tended not to get the screening done because it was on top of what the consultation was about.” (GP, Practice H)

Patel-2018-additional file

Main GP: Prior to the Torpedo trial I used a fair bit of that [online absolute risk calculation]. Very similar in terms of what to do. But they are a bit more tedious. You got to punch in the information and so on. Whereas the HealthTracker can just extract the information much quicker. So those guidelines I look at more often. I haven’t looked at a lot of other cardiovascular guidelines...normal practice you might not consciously go in and do it. Being having a HealthTracker sitting there, giving you a prompt and say “You need to go look at it”.

It [absolute risk calculation] was tedious, it was hard work. It still is like that but that gives us a bit more about absolute risk calculations.

Pope-2017

Counterintuitively, it seemed that labour substitution involving the digital technology required more staff, both clinical and non-clinical. In the context of NHS 111, this seemed in part because the new service attracted more calls on a wider range of health issues compared with previous calls to out-of-hours providers, and partly it seemed this was because calls answered by the clerical staff using the CDSS took longer than the kinds of “expert” clinician triage previously provided by doctors.

Using the new CDSS technology to deliver urgent and emergency care required considerable human work. Call handling involved multitasking by human call handlers – reading prompts

and scripts, talking through a headset, clicking a mouse, and typing text. The call handler had to control the pace of the call, establish a working rapport with the caller, and ensure that the right information was obtained:

Call handler 1: it’s quite technical and stressful,

Trainer: Although they’ve got, they passed the [unclear] exams very well, interviewed very well, and then the reality […]. Call handler 2: of multi-tasking. Trainer: Yes. Call handler 2: Listening, doing, watching, thinking, learning the process […].

[…] sitting with three/four screens, listening one ear for your dispatcher, one ear for this, one ear for that, and knowing what’s happening there, it’s difficult.

Trainer: And also there’s that sense of urgency about it as well, which is very important, and just, um, confidence, and […]. Call handler 1: It’s also being able to talk to people, it’s very important. (Focus group, NHS 999).

Reynolds-2019

“It was actually a little bit less useful than I thought that it would be, just in that the amount of information that it asks for. Well I feel that redundancy is good, you’re having to look-up so much more information that actually impedes the process of giving the medication ... It can be up to 10 to 12 minutes .. . I think it slows me down... I can use my phone and get the information this quick or just even call the pharmacy and get the information faster.”

Shannon-2021

For providers, the biggest challenge is a lack of time. Primary care providers are only allotted twenty minutes per appointment. When a patient comes in for an unrelated issue, but screens positive for depression or AUD, providers find it challenging to adequately address mental health, in addition to the patient’s chief complaint. As one provider described, “The disadvantages are always going to be what I told you, time, because they are patients who do not come only for a reason of consultation, they come for many reasons.”

Silveira-2019

Work duplication was also identified as a problem,

Sukums-2015

An increase in workload due to the eCDSS use was reported by slightly over half of the respondents in Ghana in both surveys whereas in Tanzania there was perceived increase in workload towards the end (p< 0.05, Table 1). Interviewees expressed that the ﬁrst ANC visit accounts for this challenge as it required a lot of activities and duplicate documentation.

Van de velde-2018

Within the EMR, physicians already need to click a lot. CDS requires additional clicks and I don't know if I am motivated to make that additional effort. [GP, Belgium]

Yang-2019

One year later, they stopped this practice because two recent journal articles reported that the models used were “horribly mis-calibrated”.

“That was a lot of work to type in all that sh-t and generate that number, and that’s not that helpful.” Their EMR held four other implant outcome prediction models, which predicted things such as the chance of depression. However, the clinicians never used these models, stating that each required manually entry of all of a patient’s data.
