# Supplementary material for: Stakeholder Perspectives of Clinical Artificial Intelligence Implementation: Systematic Review of Qualitative Evidence
Source: J Med Internet Res. 2023 Jan 10;25:e39742. doi: 10.2196/39742 (PMC9875023; doi:10.2196/39742)
Supplement: Multimedia Appendix 3 [file jmir_v25i1e39742_app3.zip › 5. Organisation(s)/5e. Work needed to plan, implement and monitor change/5e.1 Training requirements.docx]

**Name:** 5e.1 Training requirements

Abidi-2018

Both PCPs and patients felt that technology ineptness might be a deterrent to their use of DWISE:

One of my colleague is not tech savvy...there might be other providers like her. How can these people benefit from DWISE?...would they be interested? [PCP

Ash-2015

At the clinical sites, vendors often do training. In the case of large clinical organizations, the EHR vendors train trainers within the organization. They may also train analysts and IT staff within those organizations. In smaller organizations, the vendors may train users

Catho-2020

Some physicians were concerned about electronic skills required to use CDSSs. Familiarity with current tools and reluctance to change were also mentioned as potential barriers to their implementation.

TI_07 (M, senior physician):“The presence of a team that knows the instrument well and that can direct me on its use… I consider it an extremely interesting thing”

• GE_03 (F, senior physician): “We have to do a motivational campaign. To support and explain why”

Several participants described the importance of providing technical assistance to physicians among the incentives for adopting CDSSs.

Chang-2017

Proficiency in system operation. Because of the high number of “chief complaint” choices in the TTAS computerized system, ED triage nurses thought that proficiency in operating the system was also an important factor affecting ED triage.

If I only allocate to triage once in a while, I will only choose certain items from the system. Because there are many items and many divisions I am not unfamiliar with. . . . I may never choose those items, thus may affect the triage level. (P9)

Chrimes-2014

First impression is that would take a lot of education on the part of providers to get me to use this on a regular basis. Probably I would do what I usually do in my notes, like how many sodas and juices they are drinking, and how much exercise.

Grau-2019

A second issue pertained primarily to hospitalists’ schedules. Residents have regularly scheduled, mandatory meetings and receive weekly emails from chief residents, thereby making it easier to schedule their trainings. Hospitalists have no mandatory meeting requirements. Hence, it is less clear whether extended or ongoing, in-person training is possible for hospitalists. One hospitalist recommended that it could be added to their annual remote training requirements as a “video presentation that you can click on your own at your own leisure”

P18, Hospitalist, male: I wouldn’t have minded somebody telling me how effective [the medications listed in the NRT order set were] or where they’re getting this from.

P8, Internal Medicine, male: If we can distribute the slides or if we can have every six months a small e-mail that would say, “Here is an online refresher course, ” because we have refresher courses for the hospital.

Horsfall-2021

additional “technical training” (11/33; 33%

Joshi-2020

“[We created a video] comparing predictive models to a weather forecast. It doesn't mean you're going to put the rainboots on now because it's not raining right now.”

Klarenbeek-2021

The professionals were asked to share their thoughts about how to organize successful

implementation of the CCDSS. In order to actively engage end users, they emphasized the importance to inform clinicians about the added value that the system will bring to clinical practice. Therefore, the purpose and beneﬁts of the system should be made very clear in contextual activities, such as an introduction, training or e-learning

Lugtenberg-2015

Also, most of the PCPs reported never to have received a formal introduction of the system and/or education or training on how to use the CDSS.

McDermott-2014

"We talked about it in practice so I was expecting it…. I thought it was a very useful aid for me" (P08)

some GPs reported that they would have been happy to use and try the prompts if they had been aware of this information sooner.

"I don't think anyone actually pointed it out to me…..I might have just though 'Oh is that some sort of advertisement'…I probably would have used it, but definitely I would you know" (P05)

Miller-2019

extensive staff training and/or specialized staff

Orchard-2019

A minority of interviewees (less than a quarter) reported resistance to technology, either in relation to their own attitudes or as a perceived barrier to uptake at their practice:

“I’m probably technically challenged sometimes…I don’t want to push any wrong buttons.” (Nurse, Practice P).

“Some GPs are uncomfortable and uncertain with new technology.” (GP 1, Practice I)

Patel-2018-additional file

GP needs additional training and support to understand the rationale and use of the intervention. He found the initial training to be overwhelming. Incremental training and delegated time flagged explicitly with administrative assistant

Doesn’t feel comfortable using absolute risk to prescribe. Needs more training and skill development. Age is a barrier in using health information technology

Main GP: I mean I think you guys came around and did a few live tutorials, and that was helpful, and especially when new doctors came on board they could hear it from someone else; they don’t want to hear it everything from me. I’m not very good at explaining things, just ask my kids.

Santillo-2019

Participants were sceptical about the acceptability of the digital education module due to high workload and the large amount of existing e-learning modules, which many found boring and pointless. Although discussions about ‘review and revise’ were seen as helpful, participants stated that it can be difﬁcult to get people to

attend seminars. Alternative suggestions were to link in with ward ‘huddles’ and handover meetings

Sukums-2015

during interviews the need for a more frequent and faster onsite support was expressed by staff members. One reason for this request could have also originated from the perceived inadequate computer skills that the respondents indicated as one of the most critical challenges and that remained the second largest challenge in Ghana and third in Tanzania throughout the study.

Reasons given for these challenges were again inadequate computer skills like typing speed
