# Supplementary material for: Stakeholder Perspectives of Clinical Artificial Intelligence Implementation: Systematic Review of Qualitative Evidence
Source: J Med Internet Res. 2023 Jan 10;25:e39742. doi: 10.2196/39742 (PMC9875023; doi:10.2196/39742)
Supplement: Multimedia Appendix 3 [file jmir_v25i1e39742_app3.zip › 5. Organisation(s)/5e. Work needed to plan, implement and monitor change/5e.2 Effort and resources for tool launch.docx]

**Name:** 5e.2 Effort and resources for tool launch

Andrews-2017

Where participants struggled with the system, contingency plans were in place to ensure care was not affected.

Researcher: And when, so when someone didn’t cope with the technology, were you, was the team ready then to step back in and say ok we can take over this? P2: Yes. It would just be taken out and say, you know you would say, oh that’s fine.

Some participants suggested possible procedures for prompting, although there were concerns around staffing to implement these procedures.

P5: whether it’s a telephone call to say ‘have you done it?’, or whether it’s somebody actually saying, come on, have you, let’s do it today. I don’t know.

P7: I mean, I suppose, a telephone call, could be done, at a set time every day, but it would be, who’s gonna take that, who’s gonna do that call, and it’s not possible to guarantee that on a set day you’re gonna be free to do that, so that would probably be quite difficult to set up.

One service manager was concerned about the amount of time it would take to train the patients, and how many would need to be trained, but believed it would be possible.

Researcher: Do you think there’s capacity within the teams to provide that training to the patients? Do you think they’d have time to do that? P6: So it would depend upon the amount of training that was needed. There are, so within the teams we have different grades of staff. And sometimes if the clinician doesn’t necessarily have the time, they could arrange perhaps for the support worker to go on and spend some time with the patient, so there is the potential there, but it would come down to the numbers. However, participants expressed concerns regarding staffing for monitoring the data generated by the system and following up with full mental health screens if the system predicted the incidence of depression or anxiety.

P4: The community nurses are overworked, who’s going to do the full screening [..] What do you do with the information? Who’s gonna monitor, control it, who’s gonna read it?

Beede-2020

Some clinics attempted to screen two hundred patients within ﬁve hours, allotting only ninety seconds to screen each patient, which often resulted in extended clinic hours or patients being rescheduled for another day (Figure 4).

The screening process], it’s okay, but that’s if there aren’t 300 people putting pressure on you. With 300, it’s not okay, there’s not enough time in that crowded hospital setting.” -P2

Patients come in, around 100 at a time, and it’s not just eye screening that needs to happen...There are many things to do. After the medical history, the patient goes to the waiting area, and if there are a lot of people I have to pitch in and help out everywhere in the clinic. That’s a lot of work... Things would be better if I could just be focused.” -P7

Chrimes-2014

Yes, I think it [Flowsheet] would be useful. It can be pulled up on the next column. But my question is going to be who populates this each time.

Connell-2019

However, some more junior clinicians in both teams felt that the pathway created additional workload and suggested that clinical review might not be deliverable to all patients by the AKI response team as configured currently:

It does increase our work. Some days [...] we can have eight or nine referrals. But there is obviously a huge issue about workload for many people. But if we need to increase the size of our team because of this then that’s a good thing. And also it highlights [...] the acuity of our patients in our hospital. These patients are not straightforward. [Respondent 19: PARRT]

Cresswell-2019

there were uncertainties around the use of the Platform in real-life settings. For example, clinicians mentioned the risk of duplication of work and increased workloads despite their desire for more time with patients.

… to introduce that amount of potentially new information into a consultation that’s very, very tight time wise is always going to be a challenge. (Participant 21, male, GP, Glasgow)

Dalton-2020

But I think I don’t use it enough to immediately be able to read it through quickly which…when you’re working under pressure and time constraints in a hospital—if something isn’t easy and intuitive to read quickly in less than 30 seconds, you don’t have time, you just move on. [Medical Prescriber 8]

Gillan-2018

It was also acknowledged that this knowledge could instead come in the form of a new role in the team; someone with a strong background in AI, coding and informatics, to act as a liaison.

Johansson-Pajala-2019

To win over the reluctance to learn another system, to enter it… and the technical difficulties” (N2)

“I believe many think it's a burden [to start using the new system], that is probably the first step… I think that is the biggest obstacle to get past” (N1)

Joshi-2020

We had a 6 month project which ultimately took like 3 years. Yeah, it was years on the design, and then I still remember the email saying our first “go live” date was going to be August 8, 2017 and our actual go live date ended up being June 7, 2018 so it’s almost an entire year between when we thought we were going to be ready to go live and when we actually did.” (RB)

“we created a resource through the virtual care team that allowed nursing staff, provider staff to call anytime 24/7”

Klarenbeek-2021

In attempt to overcome these barriers of implementation, all professionals suggested to

perform a usability test and validation period for the prototype CCDSS in the organization’s real-life setting prior to roll-out to test performance. Further, most professionals expressed willingness to change current workﬂows in order to beneﬁt from the CCDSS.

[Professional ID:10] ‘We can schedule some extra time before each MDTM in order to discuss a couple ofpatients with use ofthe system. Straightforward cases are preferred.’

Lee-2015

During group discussions, radiologists confirmed that there was no substantial change in the frequency or nature of conversations with referring physicians in either direction (more or less) after CDS implementation in the MID. One radiologist stated:

We were expecting to perhaps get calls with questions about CDS. You know, “This doesn’t make sense to me.” Or “I think I have a special case here that this doesn’t apply.” But I for one have not gotten a single call about questions about the decision support advice.

For the minority of radiologists who did experience a slight increase in referring clinician contacts, increased communication was often with regard to less common MRI examinations and often with generalists rather than specialists.

Marcolini-2021

However, the time spent in the consultation was seen as a challenge for some participants. In some cases, they were forced by the health department to attend each patient for a maximum of 15 minutes, but this time was not sufficient when attending the patients using the software: I take an average of 30 minutes per consultation, i.e. two consultations. This is the same I do with prenatal consultations for pregnant women. [Male, 39-year-old physician, 6 years’ experience working as a primary care physician]

In other cases, there was an organizational issue in the primary care, which made it difficult to attend patients using the software:

My primary care center works as an emergency care. So we set aside a day to attend the patients from the project, which is a longer consultation. However, for example, we book ten patients from the program, but 20 show up; six from the project and the rest are patients with acute diseases. It is a challenge to attend the patients using the software, as there are other patients waiting. That makes it difficult. [Female, 32-year-old physician, 6 years’ experience working as a primary care physician]

Morgenstern-2021

Experts also thought that hiring staff with dedicated AI expertise was important, but noted it was difficult to compete with industry and more healthcare-oriented sectors (see Additional file 6).

… if you need talent to do stuff like this, you’re competing with industry offering […] eye watering salaries. [Participant ID # 4].

Mozaffar-2016

I think the staffing needs that we started with are very different […] I think we underestimated how much support we’d need in terms of data analysis and informatics, that’s probably something we didn’t think about very much at the beginning. (Business Case Workshop, Participant 7)

As a result, as implementations proceeded, there was often a need to increase staffing to deliver the project.

So it’s just learning for us as well of how to roll out and what support [is needed]… Because sometimes you plan for things, and then you just suddenly realize actually we need to do this a bit more. (Site D, Pharmacist)

Simplistic assumptions of the extent and the type of training required for each profession and the time required by each individual to be released from daily activities led to new challenges and extended timelines. As a result, further staffing was required to deal with temporary absence of staff from their daily duties to attend training classes. I mean we’ve had some sort of difficulties with training only in as much as people being released to attend classroom training because for nurses it’s a whole day and for doctors it’s halfa day. (Site D, Clinical Change Lead)

In many cases, the CPOE/CDS system relied upon the same HIT infrastructure that was used for other applications, and installed through organization-wide planning processes. As a result, planning of HIT capacity (hardware and networks) for CPOE/CDS became dependent on other HIT projects, which led to timelines being extended.

However, we observed that limited supplier capacity resulted in delays of system implementation. This was a particularly important issue for overseas suppliers as they had a limited number of employees present in the UK. Thus, most of the development work had to be sent back to the countries of origin. This in turn, caused complications in capturing the needs of U.K. settings.

[We were] the first to go live with meds management in [system name] in the UK fully. Therefore there was no front runner that you could say well yeah you’ve already built this for this [hospital…] so there was a limited resource in the [supplier name] world to assist the [hospital] in developing and deploying that product and that does cause delay definitely. (Site F, Interim director for HIT)

Patel-2018-additional file

GP had problems signing in and needed regular extra support. He would prefer someone to call to check in on how things are progressing with the intervention.

Main GP would like intervention to be quicker, integrated within EHR and have an allocated staff to help drive the intervention at the practice to use long term.

Lead GP: [GP3] got a whole day a week that he’s supposed to do this [data review extracted from audit tool], then that’s the place because I don’t really know what he does on most days, and I occasionally ask him and then I drift off when he tells me. So I think that’s, you’ve got him being paid one a day a week, you know, that’s where it should happen.

Philips-2015

two comments highlighted the need for more promotion of the service to inform doctors of its existence.

‘I am not aware of a dedicated therapeutic drug monitoring service. Although on few occasions I have spoken to on call pharmacist about the dosing in renally impaired, and they are always helpful. A link to this service should come up if you search gentamicin on intranet home page’

‘Worked in NHS and RGH, just started in FMC, so less exposure to be aware of A-TDM’

Pope-2017

The workforce has also grown (it did not exist in this form before the introduction of these services), and the range of roles within it has expanded to include call handlers, clinical advisors of various grades, and training and auditing staff as well as managers. These features do not seem to adhere to the promissory rhetoric of digital technologies as labour saving. Indeed

Santillo-2019

be difﬁcult communicating with different clinical teams about ARK and that there might be poor completion of the online tool before kick-off.

Shannon-2021

The other advantage at the level of technology that I see is that the implementation does not require much on a structural or infrastructure level

Sharpe-2019

Study neurologist and neonatologists were asked what could improve the feasibility of a real time response to neonatal seizure detection. Suggestions included better automated seizure detection, and a larger contingent of electroencephalographers. It was considered that a separated on call roster would be required, such that the neurologist on duty to review and respond to neonatal (and other) EEG monitoring was not also on duty for general paediatric neurology, for neurologists to sustain this level of care.

Sukums-2015

It was also observed during visits indicated that facilities with good teamwork and technology enthusiasts were able to orient new users and provide peer support to those who needed help in the eCDSS use.

Tsang-2021

Limited resources (staff & time)

“I think at least they’ve got the ring fenced resource in terms of a pharmacist. We’re not just finding patients and, there you are. We are trying to actively fix the patients where we can.” [GP1, doctor]“It’s difficult getting that time. And I found that often it was only when everything else had been done I would do it.” [A4, administrator]“It requires somebody to actually have that ring-fenced time to do it without other priorities coming out on top. And that’s difficult in the NHS.”

Six users described “ring-fenced” time and training opportunities for improvement work being crucial for the use of the dashboard to be effective, which was more likely for clinical pharmacists and nurse practitioners.

“It all comes back to the fundamental crux is that if you had more time, people and ideally money for what you’re doing I think you could proactively look after this whole group of patients that are more prone to it or predisposed to it.” [P8 pharmacist]

Wells-2014

Higher usage was reported at the AS where systems existed to support technology implementation.
