# Supplementary material for: Stakeholder Perspectives of Clinical Artificial Intelligence Implementation: Systematic Review of Qualitative Evidence
Source: J Med Internet Res. 2023 Jan 10;25:e39742. doi: 10.2196/39742 (PMC9875023; doi:10.2196/39742)
Supplement: Multimedia Appendix 3 [file jmir_v25i1e39742_app3.zip › 6. Wider system/6a. Political or policy context/6a.1 Different ways to incentivise providers.docx]

**Name:** 6a.1 Different ways to incentivise providers

Abidi-2018

Patients felt that they should have autonomy in choosing self-management support delivery method. One patient said: I don't believe that one size fits all...it is good to have platforms like apps...DWISE is easily available...it should not be made mandatory for every patient...I mean it has to be my choice.

Catho-2020

GE_03 (F, senior physician): “The feedback, to know if it is useful for something, if it works, can really motivate them”.

Johansson-Pajala-2019

Management must be committed and require that the CDSS be implemented and used for drug monitoring. This includes providing guidelines and requirements for the implementation to take place. Some RNs suggested that the

physicians should also be given reasonable prerequisites to perform systematic drug reviews and use the CDSS in this process. Others argued that conducting the drug reviews was not a choice because, according to Swedish regulations, they must be done and that the question was actually about how they should be done.

Knoble-2015

Perception of time-taking was the primary factor that impacted its use. They requested short-cuts to key features such as the differential diagnosis, emergency treatments and management options without going through the whole patient encounter process. This issue may be speciﬁc to the Nepal context where government HCWs generally work from 10 am to 2 pm. Most have their own private medical practice which they use to supplement their income. Thus, they do not have an incentive to spend the necessary time with patients in the government clinic.

Melo-2020

They also suggested that government should foster innovation through the development of startups and their integration into the healthcare sector through the creation of research and innovation centers with easy funding. They stressed that government should promote incentives for training health professionals in new technologies, so that they can take on the new paradigm.

Morgenstern-2021

And while participants had found significant interest in AI applications for public health from decisionmakers, it was difficult to obtain the financial support needed to innovate.

All of that sort of technical and brain power stuff that’s been tough for me to get, um, because I have no money. I have no money or no resources, so I’m trying to build relationships to get us to do that. So, the government in themselves have not been able to get me there. They like the concepts. Everybody agrees with the concepts. All the big people agree with the concepts. It’s delivering it. [Participant ID # 9].

Orchard-2019

Providing remuneration for screening in the context of a fee-for-service primary care system was important, as it provided an incentive to allocate extra staff time to screening.

“Thank you also for being one of the rare research studies to have been aware enough of the pressures on primary care to have built practice remuneration into your research protocol.” (GP 1, Practice C)

Patel-2018-additional file

Thought tool would be good platform to view how he was performing. This would assist in picking up additional patients that have incomplete screening and prescribing. Motivated by competition

But after I look at all the other practices and I wasn’t feeling too bad, even though it was bad”. Even though the number was quite low, and I said that I’m not far difference.

I’d like to use it more. Unfortunately timing issues. But at the end of the day if there are some criteria that are set, that it becomes compulsory for accreditations, that could be a way of forcing all the doctors and the staff and the nurses, if you have one, to look at it at the regular fashion

Find these [peer-ranked reports from web portal] very valuable in looking at how we’re going compared to other services, and whether or not we’re actually getting to the potential risk factors in the patients that we’re seeing.

Sun-2019

First, managers of IT firms highlight the lack of an “innovation spirit” in Chinese society as one of the social challenges affecting the adoption of AI in the public healthcare sector. A director of IBM China, for instance, noted the lack of social driving forces on innovation in China, especially when compared to other countries, such as the U.S.: “We have to say the innovation spirit in the U.S. should be admired by us [Chinese]. […] We need to learn from them” [2IBM01]

Tsang-2021-Supplementary file

“It is something that’s quite interesting…one of the GPs were really motivated in finding out about, why one practice does so much better than another for a different thing.” [GP2, doctor]

• “It tends to make people open their eyes and think why am I at the wrong end of a graph. Nobody really wants to be outlier in the wrong place, do they? I do think benchmarking against others is useful and it gives them their own assurance as well.” [P5, pharmacist]

• “I always like to compare so I’ve got the comparison and I usually print these off and use them for discussions and things like that.” [P6, pharmacist]
