# Supplementary material for: Stakeholder Perspectives of Clinical Artificial Intelligence Implementation: Systematic Review of Qualitative Evidence
Source: J Med Internet Res. 2023 Jan 10;25:e39742. doi: 10.2196/39742 (PMC9875023; doi:10.2196/39742)
Supplement: Multimedia Appendix 3 [file jmir_v25i1e39742_app3.zip › 6. Wider system/6a. Political or policy context/6a.2 Importance of government strategy.docx]

**Name:** 6a.2 Importance of government strategy

Ash-2020

We have to prioritize based on ﬁrst of all what our federal funders are going to require.

Benda-2020

all groups agreed that external rules and regulations were central to adoption of the predictive algorithm. However, in 2 of the 3 healthcare systems, their relatively high percentage of value-based reimbursement contracts was seen as a facilitator; whereas, in another healthcare system, which had fewer value-based contracts, this reimbursement structure was perceived as a barrier to prioritizing identification of HNHC patients. Additionally, participants from all locations agreed that local customizations may be necessary, given unique information infrastructures, and went on to describe the nuances in their particular systems. For example, 1 health system described that they typically created dashboards to convey predictive risk algorithms. Alternatively, another healthcare system reported that they typically delivered predictive algorithms in the patients’ record via “hover-overs” or “best practice alerts” (a reminder function from the Epic EHR).

Chirambo-2019

We established that for the mHealth program to run smoothly there was need for adequate funding. To this end, participants expressed the need for government commitment to funding the program. Currently the eCCM mHealth program is operated with the financial support from the development partners channelled through the implementing partners. The government contributes to the program by paying salaries to the HSAs implementing the program and IMCI coordinators supervising the implementation of the program.

The mHealth decision-making tools program is being funded by different implementing partners. The government is contributing by paying salaries of the HSAs who are implementing the program. PSA-10 “There is a good collaboration between the government and other stakeholders in the implementation of the mHealth decision making tools program such that all the parties involved are able to execute their roles smoothly.” PSA-19

.3. Funding support Some participants emphasized the need for greater financial support

from the government for it to be sustainable. Others stated that it would be good if the program operated within the MoH headquarters instead of leaving it in the district assemblies. They said that this is because the money which is sent to the district assembly for the programs is not enough to finance the mHealth program at district level.

“This is a wonderful program ……. But it needs more financial support especially from the government.” PSA-3

There is a need for the government to put in their plans to procure phones for all the HSAs in the village clinics across the country. Unfortunately this will demand a lot of funds. As such if the MoH at Capitol Hill could fund the program other than leaving it to us in the district assembly it would be much better. PSA-5

The study also out that stakeholders in the implementation of mHealth technologies would like the government to procure more phones as well as continue supporting the HSAs with extra finances for airtime for their program operations.

The HSAs will need to have airtime for them to buy data bundles so that they can send data to the servers. The HSAs cannot manage to buy airtime on their own hence the government needs to buy airtime for the HSAs every month. PSA-18

Government officials acknowledge that the government is not providing enough funds to support the mHealth program and as such they are appealing to the government to continue its collaboration with the development partners so that more funds can be channelled to the program.

The government usually sends the funds for every activity in a district including health to the district council. The funds are not enough to even support the mHealth program. As such, if donors can collaborate with the government to help fund this program, it would be welcome.

We found that the MoH officials together with the implementing partners do evaluate the mHealth decision-making tools program regularly. The evaluation results are presented to different stakeholders in the District Executive Committee (DEC) meetings.

“The program is being evaluated by the MoH officials and the implementing partners to assess if the activities are still in line with what was planned at the beginning.” PSA-4

The top-level managers involved in this program indicated that one of the major factors impacting the sustainability of the program is the availability and implementation of a strategic plan. They added that programs that start without a strategic plan can easily collapse midway through. On the other hand, those that are grounded into the strategic plan are more likely to be successful and sustainable. In this case, they revealed that the Malawi MoH has a strategic plan guiding all the mHealth programs activities as a way of assuring greater sustainability.

“The mHealth decision-making tools program falls within the MoH strategic plan and its implementation is based on the planned activities.” PSA19

The presence of the strategic plan in the MoH guides what has to be done. I feel the program will be sustainable because it is falling within the strategic plan which is currently implemented by the ministry of health. PSA-19

Sometimes programs fail to be sustainable because those implementing the programs do not follow what is on the strategic plan. If the strategic plan is followed, the programs within it would be sustainable.

Cresswell-2019

All interviewees and workshop attendees agreed that, in line with existing empirical evidence, the strategic decision to implement DSS in NHS Scotland was the right way forward.

With good supportive decision-making, people have a better chance of getting the right care at the right time in the right place. Not the wrong care too late at excessive cost, with disabling consequences.

We also observed a tension between a perceived lack of strong national-level leadership (which was considered to be needed for the successful implementation of the strategy) and staff changes within the eHealth Directorate of the Scottish Government. Several interviewees stated that these changes caused challenges surrounding strategic priorities and direction of travel.

…my preference is…we have a sense of where we’re going to prioritise the initial investment and that is all coordinated from the one position, rather than from a variety of different piecemeal pockets and funded…it needs the central coordinating function. (Participant 13, male, Clinical Lead

While calling for central leadership and direction, participants also acknowledged that a variety of different projects had to be managed under a portfolio-based approach. Here, there appeared to be a tension between the perceived need to have a firm direction of travel beyond Platform implementation and the multitude of ongoing DSS initiatives across settings (including primary, secondary and social care). While leadership was seen to be required to ensure alignment of initiatives and avoid silos, there was also a perceived need to recognise that strategy was sufficiently agile to cope with changing demands and stakeholder experiences. I think [Platform] is one of the potential ways…the fact that there’s a number of pilots which have been started to test and to see what learning we can take from that, to me feels that the right approach. But it might not be the only approach…we have to make sure that we look slightly beyond that to say, okay, and this is what we’ve got, this is the tools that we have in the here and the now. But we know that the world is changing…as technology’s concerned it’s changing really, really quickly. So, we need to be preparing ourselves for that next leap, as well. (Participant 12, female, Policy)

Melo-2020

it was agreed that government should have a determining role in creating various incentives. Legal incentives for digitization should be accompanied by an ecosystem vision, and a transformation of governance models is needed to enable opportunities to be seized and to respond to digital threats within both public and private systems, thereby reducing barriers to technology incorporation

Government and business will need to be able to participate and develop professional retraining programs because workplaces will demand new interactions from professionals with machines and with the physical and virtual world.

Finally, they expressed that government should put in place legislation to promote the protection of user data because huge amounts of data will be generated. Government must step up its role in providing universal health coverage, adapting from a global perspective, across country borders

Morgenstern-2021

Despite major advances, interviewees believed that AI is shrouded in hype, and that this could lead to it taking resources away from proven approaches. That whole concept that, you know, this is what’s new, this is what’s getting hyped, this is what’s absolutely sexy and is starting to suck in policymakers, funders, etcetera. To the point where we take from where we should be investing because the historical track record indicates that it has produced good. [Participant ID # 1]

Sun-2019

As remarked by one of the government policy-maker informants: “This time, we [AI policy-makers] emphasize institutional, legal, and ethical issues. This is what we don't have in previous plans [on other policies related to Science and Technology]” [5GOV01]. Government policy-makers all agree that AI has ethical considerations that differ from other traditional technologies [5GOV02, 6GOV01, 6GOV02].

A first challenge, framed by government policy-makers and hospital managers/doctors, stresses the potential threat to national security when a foreign company, such as IBM, collects and stores large amounts of personal data on Chinese patients. Letting a corporation of a foreign country have access to the health records of Chinese citizens could make China more vulnerable, for instance, to biological warfare. This is considered to be no less than an existential threat for the continuation of AI in the public sector as a whole. As highlighted by one of the IT firm managers: “Once the [healthcare] data is used by bad people for evil purposes, AI will die” [1HP03]. Government policy-makers remark the importance of sensitive data not being held by foreign companies, and that, beyond limited experiments, the government will not support non-Chinese firms at a national level: “AI use in healthcare will be controlled […]. Personal information is very important. Thus, foreign firms will not get [policy] support. Experiments are ok […]. But, once there is a [security] problem, the Chinese government will close the door [on AI use]” [5GOV01].

Van de velde-2018

Participants suggested public governance of CDS. Some thought it could also be private but not financed by the drug industry:
