# Supplementary material for: Stakeholder Perspectives of Clinical Artificial Intelligence Implementation: Systematic Review of Qualitative Evidence
Source: J Med Internet Res. 2023 Jan 10;25:e39742. doi: 10.2196/39742 (PMC9875023; doi:10.2196/39742)
Supplement: Multimedia Appendix 3 [file jmir_v25i1e39742_app3.zip › 6. Wider system/6a. Political or policy context/6a.3 Policy and practice influene each other more.docx]

**Name:** 6a.3 Policy and practice influene each other more

Benda-2020

Multiple participants described how stakeholders and organizations might use the information provided by the analytic model. Possible goals included identifying previously unknown subsets of at-risk patients, helping care managers prioritize patient lists, and helping organizations advocate for additional resources on the basis of their prevalence of HNHC patients.27

Even if we systematize it and take it out of that subjectivity ... that would be a godsend. – INF01 [Facilitator] I could easily see us going to that payer and saying, ‘Well, our risk model .. . shows your patient population is higher risk. We need to do more intervention, so we need more money.’ – OPS04 [Facilitator]

Key metrics that could be tracked and improved with implementation of the predictive algorithm included patient outcomes and utilization-based metrics (eg, emergency department visits, unplanned readmission).

Catho-2020

• GE_08 (M, senior physician):“We are a little detached, a little further away and what is not bad is to have us, a feedback, because if we see indeed that there are important deviations towards certain guidelines...”.

• GE_06 (M, resident):“There is so much work to do, that in the end it’s a huge help, it’s reassuring”.

Motivation or intention to use CDSSs were frequently described as linked to the amount of eﬀort required to use them. Participants would like a system to be user-friendly, fast and with limited need for data entry. The source of recommendations available directly in the system was described as an important factor for adherence, particularly by senior physicians who perceived the possibility to provide feedback on compliance with CDSS recommendations as a tool to increase younger physicians’ motivation to adopt them. Sev

Chirambo-2019

The authorities in mHealth implementation organizations and the MoH pledged to support continuation of the program because they had seen clients benefitting from mHealth apps support.

“It is necessary that the program is extended to other parts of the entire country because when using mHealth technology, we know HSAs will give the right treatment to children”

Flynn-2015

COMPASS was also used to assess the potential (missed) outcomes for a patient that had not been referred to the stroke team. Opportunities to use COMPASS, but where it was not used by clinicians were reported on eight occasions.

Gance-Cleveland-2019

Administrators reported that StartSmart was useful to promote adherence to national Referral to Sleep Center guidelines

Johansson-Pajala-2017

Some RNs suggested that comparisons could even be made on a community or national level.

‘We can withdraw statistics which we can bring to the team meetings... we can see that we have made this many medication reviews and these drugs have been removed’

Joshi-2020

“I think we are challenged generally in evaluating our decision support efficacy…where we struggle the most is looking at the patient outcome once the system’s triggered just because it’s hard to track patients like that. But we're certainly able to track when it triggered, what did someone do. What did someone order, what's the turnaround time from the order to the administration of whatever they ordered, and then the part where we have trouble is figuring out did the patient actually become septic, did they get transferred to the ICU, did they walk out of the hospital, or did they die, things like that.” (RB)

Liberati-2015

This system could have a cultural relapse ral and important didactics. It might help to develop to develop a culture of evidence in a group of work. You could use it not individually, but as a tool to reason together on the diagnostics yes and to monitor some particular beds in a Department". (Orthopedic surgeon, setting A)

Orchard-2019

In many cases, GPs had never seen their practice data presented in that way. In some cases, it led to quality improvement, e.g. review of management for AF patients who were not previously treated according to guideline.

Petkus-2020-supplementary file

There also must be clarity on the governance around its use (likelihood of audit trail to assess the impact of CDSS on efficiency, effectiveness and improvement to patient care).”

Shannon-2021

“For us total advantages, because it is a population that did not have a measurement of mental health pathologies and that now we can quantify it, know how much is the prevalence … and to be able to follow up on the patients is a total advantage.” Not only are administrators better equipped to create infrastructure to support patients with mental health conditions, they now have data to convince government funders and insurers of the importance of providing mental health services for their patients

Torenholt-2021

PRO-data and algorithms were consequentially recast, no longer being tools for ‘process automation’, as they appeared to be in the consultancy report, but rather tools for ‘systematic’ and ‘efficient’ patient involvement. These digitised capabilities are referred to in various and less conspicuous ways: ‘data are sent to the database for further processing’; data are used for ‘advanced decision-support’, or in ‘weighting’, or as ‘a smart selection of patients’ – here quoting from a number of internal reports, policy documents and public meetings. The objective around the use of PRO-tools for automated triage therefore seemed to change when PRO-tools were presented closer to clinical practice.

Tsang-2021

users described utilising this indicator page to spot high-risk patients and explore reasons why they had been missed, without perceiving the task of AKI coding to be a priority. Further challenges of coding are discussed in the next Section 3.3

“That’s really useful because I suppose that shows who we drop the ball on or who doesn’t want to come and see us.” [P8, pharmacist]

Tsang-2021-Supplementary file

“We can set up projects to make sure the practice improves, then test it without necessarily having to audit religiously because we can see the figures from it. So that’s helpful.” [P5, pharmacist]

• “I think it’s good because it’s easy to use it as an audit after; you can do a before and after.” [P9, pharmacist]

Watson-2020

Finally, interviewees expressed concern that clinically implemented models might only improve process metrics without impacting clinical outcomes. As one interviewee explained:

How do you prove that an outcome was improved because someone used the dashboard? It’s really hard because most of our business surrounds process improvement. So, just because I improved the process doesn’t mean I made your disease process better. We think it does, but really there has yet to be good solid evidence that links that.
