# Supplementary material for: Stakeholder Perspectives of Clinical Artificial Intelligence Implementation: Systematic Review of Qualitative Evidence
Source: J Med Internet Res. 2023 Jan 10;25:e39742. doi: 10.2196/39742 (PMC9875023; doi:10.2196/39742)
Supplement: Multimedia Appendix 3 [file jmir_v25i1e39742_app3.zip › 6. Wider system/6b. Regulatory or legal issues/6b.1 Deciding who is responsible.docx]

**Name:** 6b.1 Deciding who is responsible

Abdi-2021

“there are some potential safety, ethical and policy issues that need to be addressed, which may take longer than the 10 year time frame to properly address. For example, if a person falls at home and the system does not recognize it accurately, do we blame the system”

Abidi-2018

Ok, suppose I am using this app with my patients, what if I missed something or I fail to do what is expected of me? Would I be liable...what will be the impact?

Alagiakrishnan-2016

Many respondents expressed concern about how

CDS overrides might be documented or communicated. Physicians described a “balancing act” weighing benefits and risks and noted that a patient may be compromised by drug discontinuation

Considerable concern arose when physicians opted to continue prescribing a medication despite cautionary CDS alerts. Uncertainty about whether CDS-suppression logs could be referenced in any future legal proceedings was troubling some respondents

Ash-2015

The EHR vendors and the content vendors clearly state that they do not practice medicine and therefore should not be liable for decisions that can only be made by clinicians. They provide a vehicle for using CDS content, but it is the responsibility of the clinician to decide the medical relevance of that content for the patient’s care.

Blease-2019

Technology will be supporting clinicians in the very near future – the issue is responsibility and liability in legal terms for such tools. [Participant 453]

Bourla-2018

Data privacy

Medical liability

Catho-2020

GE_08 (M, senior physician): “If there is a problem, if the choice has not been the right one, if there is a therapeutic complication, who is responsible. Is it the doctor, or is it the system?

GE_05 (M, resident):“There could be really big complications if people follow the recommendations in the system. There could be errors and serious complications”

GE_08 (M, senior physician):“In 2019 we have increasingly powerful computer tools. We must use them. If we don’t use them, we could be blamed for it”.

Some participants described defensive medicine as a factor that may limit the uptake of CDSSs (e.g. if the CDSS suggests a narrow spectrum antibiotic but the patient is very ill). Conversely, others suggested they could be accused of negligence if CDSS recommendations were not followed (e.g. by making a decision not in accordance with local guidelines)

Dikomitis-2015

Although not speciﬁcally asked about in the interviews, several respondents expressed concern with regard to the medico-legal implications of using eRATs, for example, of not referring a patient who had been brought to their attention via a prompt: ‘Quite a few partners were worried about any medico-legal implications with that. I think the one criticism was having the list of patient with PPVs. No one really liked that. That was worrying. We’re worried about if patients knew that you had a list of them with the risk and you hadn’t acted on it, what would be the implications? That was probably a point that put people off, really’.

Goetz-2020

The students questioned who would be responsible for mistakes made by the vPCP. Many

students expressed that this would need to be made clear to them before they would use a virtual physician.

“. . .it’s worse if a computer makes the mistake because then the idea is, well, who do you sue? Whose fault is it?” (Fourth year medical student)

Haan-2019

Patients note that radiologists can be held accountable for their mistakes, and they wonder who can be held responsible for errors made by computers. Some patients report that a computer is just a “giant calculator” or a “dead thing” and that humans will always be responsible

Horsfall-2021

responsibility and ethics (5/33; 15%),

Johansson-Pajala-2017

The physician should take the ultimate responsibility The RNs claimed that the physicians were ultimately responsible for the patients’ drug treatments. Still, they perceived that much responsibility was put on them, and they felt compelled to be well informed and prepared before the medical rounds. Subsequently, they read the reports and prepared questions and suggestions about the current drug treatments, even although they expressed it should primarily be the physicians’ responsibility to assess them.

‘It is the physicians’ responsibility to prescribe and end drug treatments, I insist on that, it is not RNs’ responsibility to keep track of adverse drug reactions and which drugs do this and that...of course I am interested but it is not really our responsibility to do this’

Klarenbeek-2021

Further, with the introduction of the second component of the CCDSS, professionals felt legally more vulnerable for potential criticism from patients since the system would expose discrepancies between formal guidelines and contextualized decisions.

Lai-2020

These expected developments could thereafter cause various ethical problems, depending on the type of information provided by the AI tool.

Moreover, some physicians believe that they should first ask themselves what the needs and possible changes are, rather than go directly to the AI tools. Thus, if AI tools become crucial in medical decisions, physicians stated that they were not prepared (would not agree?) to be held criminally responsible if a medical error was made by an AI tool

Paradoxically, they said that the question concerning responsibility in case of injury was not yet relevant. For them, AI tools are only meant to help doctors with their decisions and not to replace them. The rule of law should therefore remain unchanged in their view. In addition, those in industry were quite clear about their not being ready to be held responsible for their AI tools if such a tool induced harm to a patient because of an unpredictable evolution of the tool due to a “black box” phenomenon. They also pointed out that their being considered to be partially responsible in case of injury would hinder the development of health AI tools in France.

Liberati-2017

The fear of losing control over autonomous decision-making becomes particularly acute in the event of medical-legal controversies. Some participants expressed concern that the presence of CDSSs may provide non-medical experts with the formal authority to judge medical decisions, thus forcing physicians to follow the system’s advice to avoid the risk of medical and legal charges. Since the CDSS would reveal the discrepancy between formal guidelines and contextualized decisions, clinicians may become legally vulnerable. Judges, lawyers, or other professionals lacking specific medical expertise may appraise physicians’ actions based solely on the indications of CDSSs, without considering other aspects that guide the clinical decision-making process.

It’s a double-edged sword. Let’s say I don’t turn it on and for whatever unrelated reason something happens to the patient. A judge may say “How is it that you didn’t use the CDSS even if you could have?” It can be used against us. (Physician, setting B)

Some doctors asked me […] what if I do something that is different from what the CDSS suggested? […]So what happens if a doctor refuses to follow the system’s recommendation? […] I think the legal framework needs to be clarified. (IT staff, setting C1)

Lugtenberg-2015

Fear of misuse of data by third parties

“And then the healthcare inspectorate comes down to visit and asks: why didn’t you do this or that when there was an alert. It shouldn’t be used for this purpose!”.

McCradden-2020

that they had expected such a mistake, and nearly all were accepting of the notion that mistakes happen. Participants almost universally supported disclosure (although 1 patient disagreed, fearing repercussions to the algorithm developers) and reparations, including lawsuits (“they should suck it up and pay” [participant 18–007, caregiver])

When asked who was responsible for the mistake, most participants pointed to those who developed the algorithm, with a few specifically blaming the people who input the data into the computer. One participant said that the person most in charge was responsible for the outcome. One provider described the need to publish and report the negative results so that others would not repeat the mistake.

Nelson-2020

When asked to identify entities responsible for AI accuracy, patients most often named the technology company (25 [52%]) and the physician (20 [42%]), followed by the collective (12 [25%]) and the health care institution (11 [23%]). When asked to identify entities responsible for AI data privacy, patients most often named the health care institution (25 [52%]) and the technology company (19 [40%]).

Sun-2019

The last challenge is related to the lack of rules of accountability in the use of AI for decision-making. As AI technology replaces parts of the decision-making process traditionally carried out by humans (e.g., the design of treatment plans), there is no regulation on how to include non-human actors in the legal accountability system. The challenge [of AI adoption at hospitals] is: how to clarify responsibilities and what are the standards or regulations? A machine cannot take responsibility by itself, as a human being can. [2IBM02]

In China it is illegal for an AI system to make a decision [1HP05].

This is a big obstacle to the adoption of AI in the hospital. As told by one of the hospital managers/doctors:

In our hospital, we use Watson to assist the Multidisciplinary Team (MDT). […] We discussed how to use Watson for a long time. […] As the first hospital to use Watson, we find this way [to use Watson together with the MDT]. […] But this really gives us a heavy burden! Because when we use Watson, we must have at least five doctors to work together with Watson [as required by regulation]. They [the five doctors] will sign on the report. [1HP05]
