# Supplementary material for: Stakeholder Perspectives of Clinical Artificial Intelligence Implementation: Systematic Review of Qualitative Evidence
Source: J Med Internet Res. 2023 Jan 10;25:e39742. doi: 10.2196/39742 (PMC9875023; doi:10.2196/39742)
Supplement: Multimedia Appendix 3 [file jmir_v25i1e39742_app3.zip › 6. Wider system/6b. Regulatory or legal issues/6b.2 Product assurance.docx]

**Name:** 6b.2 Product assurance

Abdi-2021

Safety and ethical concerns were also raised about the use of some technologies, such

as AI-based technologies

Technology will likely mature and pass regulations over the next ten years” Portable diagnostics in access to healthcare domain, P18

Adams-2020

When evaluating an AI tool, many participants felt conﬁdent trusting their physician as an expert to recommend whether they should rely on an AI tool; if their physician determined the AI tool to be acceptable in terms of diagnostic accuracy and impact on health system processes, they would generally accept the tool as well.

Alaqra-2020

Many interviewees, especially those with some basic technical knowledge, are skeptical on how the data analysis on encrypted data is plausible, they highlight the need for testing and certiﬁcations in order to trust PAPAYA

They noted that the service provider must also show reports containing details of the PIA method, process, and the qualiﬁcations of the evaluator. In addition, the evaluator must be an independent expert not associated with the service provider

Cai-2019

While participants naturally insisted that the AI Assistant be accurate, many were not sure what

should constitute a reasonable performance threshold. Instead, they desired to contextualize and compare its behavior relative to “human benchmarks.” For example, participants wanted to know how its diagnoses correlate with a panel of GU pathologists; what its error rates are relative to personally observed human error rates; or how it compares to published rates of concordance between general pathologists. In sum, pathologists desired empirical measures of performance, but in order for these metrics to be meaningful, they may need to be reported relative to human benchmarks, and with a precise definition of metrics used (e.g., how accuracy is being defined).

Whereas initial needs tended to center on high-level performance metrics, participants eventually expressed a deeper desire to understand the AI’s specific categorical strengths and limitations. The most common desire was to understand the pitfalls of the AI system, so that they could anticipate those weaknesses and account for them during decision-making: “What is difficult for the AI to know? Where is it too sensitive? What criteria is it good at recognizing or not good at recognizing?” (P18) Participants described parallels to the current onboarding materials they use in medical practice, which typically highlight known pitfalls and limitations: “There’s a prep for pap smear and...at the edge, the cells dry out and look bigger, so it’s a known thing to not evaluate or you might think there’s dysplasia where there’s not. So they point that out in the training materials.” (P4) Others described learning about pitfalls from colleagues who have time-tested experience using the technology.

Many participants were able to describe specific scenarios that the AI Assistant should be

validated against. These scenarios are known to be difficult for humans, such as benign mimickers of cancer (e.g., atrophy) or special sub-patterns (e.g., cribriform, perineural invasion): “Maybe it has really good accuracy except for perineural invasion. If you see perineural invasion...don’t fall for that.” (P20) Instinctively, pathologists often assumed that the AI would have difficulty with the same special cases that they themselves struggle with. However, they usually gave themselves more credit in being able to properly handle these cases, referring to their own perceived ability to “correct” for those exceptions: “I would call it not-interpretable. Most pathologists aren’t going to interpret something right at the tissue margin.” (P17) Several participants eventually wondered whether the AI could have already corrected for those factors as well: “It’s important to know that the AI is correcting for it or to know that it’s a flaw.” (P4). Given these well-known special cases, one could imagine stating in onboarding whether the AI has been trained to handle known edge-cases, to aid participants in building correct mental models.

Cresswell-2019

Another concern expressed at workshops and in interviews was that, for clinicians, the patient-centred approach may be in direct contrast to other policy drivers such as efficiency and patient outcomes. GPs described scenarios where patients might choose minimal intervention and therefore have a worse outcome. You’re trying to reduce variability for drugs etc. but this can go against [patient centred approach] where you are giving personal choice. How can success be measured? (Glasgow Workshop, Table Feedback

Dikomitis-2015

Although there were instances when GPs found the eRATs to be at odds with referral guidelines from their administrative and commissioning bodies or with existing local referral guidelines, overall, respondents felt that the tools complemented the National Institute for Clinical Excellence cancer referral guidance: ‘Other colleagues have said as well we’re far more aware of thrombocytosis and increased platelets. We weren’t aware that tended to increase the score for increased cancer risk’. (GP/10)

‘Particularly for the lung cancer or the risk of lung cancer patients, I found it useful; I would say not so much for the bowel ones because it was based on the symptoms we’d already thought of as potentially risky’.

(GP/6

Gillan-2018

TP-RTTs tended to focus on the risks of adopting novel techniques, facilitated by AI, before evidence supported their use. They suggested that caution should be employed until the data produced supports the value to patients and their care

Goetz-2020

They also iterated that any vPCP must be adequately tested before its release:

“So, if I know that it’s an effective tool, it’s been proven, it’s been vetted, that would make me feel more comfortable.” (First year medical student)

Students expressed that data entered into the vPCP must come from or be verified by multiple trustworthy sources without competing interests.

“[Y]ou have this human checking, was this product right or wrong. . . we have to make sure that those companies. . .they’re not implementing a product just for the sake of having it tomorrow. . .we need some professionals in the field, and everybody who is trying to implement this sort of AI would have to meet [their] requirements before they can actually send it to hospitals” (Fourth year graduate student

Haan-2019

Proof of technology is related to the belief of patents that they can trust the efficacy and reliability of Al throughout the entire process of scan evaluation. Patents report that scientific evidence is required to validate the use of Al systems in radiology before they can be used in practice.

Patients also mention they prefer humans when research shows that humans and computers are equally skilled in performing their job. However, when scientific research shows that computers are indeed superior to humans, most patients indicate that they would rather let the computer do the work than the radiologist

Lai-2020

Another pitfall that was always brought up was the sense of excessive regulation concerning health data used by private companies in France. However, at the same time, they had the impression of there being a legal loophole and a lack of clarity of the legal documents. They considered that current laws are able to address the new issues that arise with AI, thus triggering a will to legislate in the hope of devising a better framework for AI

Members of regulatory agencies are beginning to take an interest in the subject but appear to be currently overwhelmed. The primary role of the regulatory agencies will be to provide recommendations and regulate the implementation of AI. However, one of the interviewees shortened the interview by saying that AI was pure speculation and that it has not been a topical issue thus far. AI was still a relatively unclear concept for several other interviewees. The posture they adopted could be defined as more-or-less informed expectation. It appeared that some work groups were emerging, but in the absence of concrete integration of AI into care, the regulatory agencies appeared to have a relatively poor grasp of the subject. However, some participants reported that there were already actions underway to facilitate the development of AI, even though they were not always visible to those in industry, who complained of a lack of proactivity from the ministries. Although regulatory agencies were not particularly in favor of succumbing to the ambient willingness to legislate, some participants suggested that regulatory agencies could rely more on soft law, as well as guidelines, to be more helpful and visible for both healthcare industrialists and physicians. Nonetheless, some of members of the regulatory agencies expressed that they will likely be the first to be involved in the assessment of AI tools concerned by the “black box” phenomenon. However, if healthcare professionals are to use these tools, the regulatory agencies know that it will be necessary for them to be able to trust the assessment process, as in the past. This appeared to be a fundamental point for integrating AI into the current practice of healthcare professionals and health regulatory agencies sometimes appeared to be ill prepared to take on this huge responsibility

Lennox-Chhugani-2021

The women assumed that this technology would never be used without clear evidence of its effectiveness. They expected the impact on equity of access to breast screening to be closely monitored through governance processes

Morgenstern-2021

Experts indicated that AI has been most impactful so far in commercial efforts, such as sales and marketing, where there is often higher tolerance for errors than in public health.

[A soda company brand] can maybe afford some level of error […] in what a machine spits out when it’s doing a marketing campaign to sell more [beverages.] [….] We don’t have that same luxury […]where we’re dealing with human life and human health. And so, I think that’s going to be a struggle for us because we’re going to need to have that higher threshold of accuracy and more confidence that [the] machine-created algorithm is going to be acceptable. [Participant ID # 2]

As such, most participants agreed that rigorous regulation is necessary. However, they were unsure of exactly what form this should take.

So, somehow, we almost need like [a Food and Drug Administration] for artificial intelligence, that will regulate constant evaluation of the AI tools that we are incorporating into practice. [Participant ID # 13].

Petkus-2020-supplementary file

regulation has tended to be 'hands-off,' so that there is a discontinuity between the rigour with which medicines are regulated and the laxity of regulation of the software that allows them to be prescribed;

Sun-2019

With regulation support we will feel safe. […] Without standards and regulations, they [the hospitals who use AI] will worry if it [Watson] can be used in this way” [3IT01]

As background, the analysis of national government policy documents reveals the awareness of the relevance of ethical challenges brought by AI. In these documents AI technology is framed as a disruptive technology that is expected to have a strong impact on ethical principles and which needs to be carefully monitored
