# Supplementary material for: Stakeholder Perspectives of Clinical Artificial Intelligence Implementation: Systematic Review of Qualitative Evidence
Source: J Med Internet Res. 2023 Jan 10;25:e39742. doi: 10.2196/39742 (PMC9875023; doi:10.2196/39742)
Supplement: Multimedia Appendix 3 [file jmir_v25i1e39742_app3.zip › 6. Wider system/6b. Regulatory or legal issues/6b.3 Impact on patient groups.docx]

**Name:** 6b.3 Impact on patient groups

Abejirinde-2018

some women felt special for being selected, others felt otherwise excluded or came up with plausible explanations of why they were not screened with the device.

two pregnant women said interaction with the box was proof that ANC was properly completed.

“You know when I come and they don’t use it to attend to me and I just get back home like that, it doesn’t actually feel like you came”

Goetz-2020

While this was a recurring theme, it was not shared among all of the students. A few students thought that availability of a vPCP could lead to increased discrimination related to medical care. For example, one student expressed concern that the vPCP would be made available only to poorer or uninsured patients, while wealthier patients would be able to see a human physician

Jutzi-2020

In addition, the risk of unequal opportunities due to potential high costs not covered by standard health insurance was pointed out.

Lai-2020

It is thus necessary to think upstream about the balance between private life and health gains to define what would be acceptable or not for society. An individual should then be able to make his/her own choice because there is also a question of freedom that arises with this subject.

In addition, other questions were raised concerning patients: would their choice really be considered, knowing it may be biased by the fact he/she is ill? Will the government make a choice concerning an individual when it comes to public health choices, instead of the individual himself/herself? It is therefore the responsibility of the regulatory authorities to also protect individuals, and the one of physicians to keep valuing the individual in order to allow patients to make their own choices.

Lennox-Chhugani-2021

2. A lack of clarity on how the AI tools will be governed. 3. Potential discriminatory bias. 4. A lack of clarity on how data privacy will be protected.

Morgenstern-2021

Bias must be controlled Participants were concerned about the potential for AI to propagate social and cognitive biases that can become part of the datasets used by AI algorithms for training.

… data and algorithms just kind of absorb and amplify the biases that we already have. [Participant ID # 10].

Despite these concerns, some interviewees pointed out the potential for AI to remove bias from judgments.

… [AI] removes that human element, which in some ways is quite good [even though] some people don’t like it. I think there’s a huge benefit of having that human element and that potential bias removed from the […] actual actions. [Participant ID # 9].

Respondents were worried that the use of AI and novel complex data sources to better target interventions in public health (sometimes referred to as precision public health) [25] could worsen health inequity, both within and between countries, based on ability to afford the necessary technologies.

… one of the huge concerns that I have around so-called precision medicine, precision public health, is it just strikes me that these are inherently inequity exacerbating […] for a variety of reasons. […] Who’s gonna be within a health system that’s got the capacity to develop these things and produce products that are used for … You know, it ain’t going to be low- middle-income countries. It’s going to be high-income countries and it’s going to be specific communities, you know, entities, economic strata, within that. [Participant ID # 1].

Furthermore, there is the potential for selection bias in the data used for AI applications to further marginalize underrepresented populations (see Additional file 6).

[If your data is not representative of the population] you could be, you know, making things worse, consistently, systematically, for people in terms of what you’re recommending or detecting or not detecting. [Participant ID # 3

However, others proposed that the increased health information and service-accessibility enabled by AI may in fact reduce inequities.

… we know that better-educated, higher-income people tend to have healthier diets and access to better information […] So, [we may reduce inequity] if we’re able to create better personalized tools, […] like a voice interface that can give someone with a low reading level access to good nutritional information. [Participant ID # 7].

Shannon-2021

That little machine there generated a lot of curiosity, and when people told them [patients] what it was about, they offered to participate, to fill out the questions there in the kiosk so that the patients had a more participatory role in their care

Vedanthan-2015

Nurses felt that patients viewed the device as futuristic and improving patient care. While the device has pre-programed decision support and does not have telemedicine features, some patients believe that the device connects in real-time with the tertiary care hospital and that they are receiving advice from a physician. One nurse has observed patients traveling from outside their catchment area to her facility for treatment because the clinic has the DESIRE tool.

Participant: in fact they are thinking, they are saying we are connecting to Referral [the tertiary hospital], that's what they think. And feedback is coming so the management is from daktari [doctor] from Referral (laugh) [...] this tablet is sending information to daktari in Referral and he is sending back feedback so it's like he is being seen by doctor in Referral (laugh)
