# Supplementary material for: Stakeholder Perspectives of Clinical Artificial Intelligence Implementation: Systematic Review of Qualitative Evidence
Source: J Med Internet Res. 2023 Jan 10;25:e39742. doi: 10.2196/39742 (PMC9875023; doi:10.2196/39742)
Supplement: Multimedia Appendix 3 [file jmir_v25i1e39742_app3.zip › 6. Wider system/6c. Professional bodies/6c.1 Resistance from professional culture.docx]

**Name:** 6c.1 Resistance from professional culture

Andrews-2017

Some expressed this as a need for a change of culture, whereby staff needed to understand the benefits of preventative systems.

P6: There’s a whole cultural change that we’re going to have to go through, in terms of getting people on board, and seeing it as part of their role, and you know, them thinking that they’ve got the time to do it, and understanding that actually by dedicating some time here, you might save some time down here

Ash-2015

Content vendors discussed the business necessity of staying (and appearing) neutral toward both EHR vendors and end-users, given that EHR vendors are also (competing) customers. They cannot afford to alienate potential or actual customers.

Catho-2020

TI_02 (F, senior physician): “Doctors are, by deﬁnition, refractory to everything that is computerised. Now that we are moving on to the computerised patient ﬁle, I see many of my colleagues in diﬃculty, because of the psychological approach more than anything else, so a priori, what is on the computer is not good and paper was better”

TI_09 (F, resident):“I believe that we doctors often show the sin of pride and low humility… we do not want someone to tell us what drugs to use in the treatment of diseases”

Moreover, the risk that CDSSs may reduce their professional autonomy (e.g. reluctance to be told what to do) was also noted.

Flynn-2015

Potential perceived barriers to use were: clinicians’ acceptance of the outcome probabilities; capabilities

Hallen-2015

Physicians viewed evidence-based prognostic estimates produced by CPMs as superior to their own experience-based estimates:

Pulmonologist 1: I am a ﬁrm believer in systematic type of population-based tools to assist in practice. I mean, that’s sort of what the whole of the ICU is designed to do and I guess another analogy that pops into my mind in this regard is the evidence on protocolized weaning assessment and extubation ... when you actually assess everybody in a systematic, structured way every day... you have much better outcomes.

Jacobs-2014

“Team members reluctance to rely solely on electronic communication

Joshi-2020

“People didn't really understand it. They were touting it as an artificial intelligence and people didn't understand what that was.”

Liberati-2015

[In surgery, the concept of evidence sometimes does clashes with the concept of art, especially in the orthomedia that you work with tools that recall that-them of the craftsman. […] We learn and build ourselves a culture by reading books and articles, but also by looking giving, watching the masters, watching how they do it them… Also because of evidence in our field there are few of them and therefore such a system to us it would be of little use ... ». (Orthopedic surgeon, setting C)]

[The directions seem aware that the over-ment of the position of total rejection of the SSDCs it will take a lot of effort and a long time negotiation with clinicians on the front line with clients, aimed at introducing a radical change cultural mentality in the organizations involved]

Liberati-2017

believe in our expertise, our conscience and experience. It’s humiliating to think that we can be substituted by a computer! (…) We need to have the courage to do what we think is right, not to merely comply with the guidelines dictated by a system. (…) The knowledge that I get from visiting 150 patients is more substantial than what (the CDSS) can give me. (Physician, setting B)

Any innovation that has the potential to affect clinical autonomy and decision-making shouldn’t be introduced like an imposition. If it’s perceived as a top-down order, clinicians will reject it. Physicians must agree and engage in the project. Having said that I think a strong endorsement from the top management is essential. (IT specialist, setting C1)

A manager suggested that, although a number of top-down strategies must be adopted to reach this implementation stage, these are only effective if combined with a constant effort to nurture clinicians’ engagement with, and perception of control over, the CDSS. However, up to this position, the desire to shape the CDSS remains mainly theoretical, lacking practical details for effective changes.

Morgenstern-2021

As such, participants thought that it should be emphasized that concerns regarding issues like bias apply equally to AI applications, as they always have in statistical applications to public health. It was posited that AI researchers have been mostly focused on developing advanced methods, while data generation and potential bias has been of less concern, leading to some of this confusion.

[The computer science] discipline has been focused on developing the method, not generating the data. So, and that’s not a criticism, that’s just what they do, and it’s great that they do. But when you’re in public health […]you have to focus on […] generating that data or understanding the pros and cons of different data for a public health application. We spend a lot of time, actually more time, on the dataset, data creation, data analysis, […] data interpretation, data integration than anything. [Participant ID # 12]

Mozaffar-2016

Customization was also responsible for delays. This type of delay was usually a result of: a) the CPOE/CDS system needing an element to run or Anglicization was needed to fit UK specific needs (e.g., the need for a particular UK specific requirements on ‘To Take-home Orders); or b) opportunities were identified to enhance the use of CPOE/CDS systems by modifying the built-in functionalities. In the first case, dependency on a particular element could be a necessity for the organization to perform (so less of a tactical choice but more of an imperative), whereas in the second case customization was a choice rather than a necessity (thus more of a tactical decision).

I think there are probably quite a lot of problems with [system name]. In the first place, it came as quite an American product and it’s taken quite a long time to Anglicize it and make it more suitable for the UK market … there are quite a lot of NHS specific things which are not built. (Site D, Information and Communication Technology Manager)

Orchard-2019

“I don’t feel motivated to do it because nobody else was interested.” (Nurse, Practice N).

Porter-2018

In site one, there were anxieties about the support which would be available from the ambulance service if a non-conveyance decision (as a result of following the CCDS guidance) led to a poor patient outcome:

This trust is pretty much, hmmm, ‘guilty until proven innocent’. So, I think a lot people want to err on the side of caution, and not leave people at home and take them in regardless of what a computer is sort of suggesting. (End S1 07)

Ruppel-2021

[Uncompromising scientists like Insel, so the argumentation of this psychiatrist, cannot be satisfied with the previous development of psychiatric classification systems, because taxonomic decisions are not only made on the basis of research results, but are ultimately dominated by interest-driven processes. As a consequence, the diagnostic categories of these systems are those that “do not [help] to get to the core of things” (P9: 628-9). They are a hindrance, so this argument could be exaggerated, because they do not reflect the natural Orning, but are socially overformed and thus have "arbitrate" or "artificial" character.]

Soling-2020-supplementary file

“It's okay to pay more attention, but I... on the basis of this, yes, the recommendation I can ... I will still not change the therapy because this is also from the cardiologist and this is the treatment for heart failure, yes, even if the side effects or the interactions are known.” [GP2, p.3]

Sun-2019

As mentioned by one of the IT firm managers: “They [the doctors] have very high expectations from Watson. […] For example, doctors think it [AI] can only do some simple jobs. It is too weak” [3IT02]. Hospital managers/ doctors report to experiencing frustration when facing the real technology after the societal hype: “We have difficulties on AI adoption in healthcare. […] especially for the top hospitals, they will think our doctors are much better than Watson” [3IT01].

Urquhart-2018

Second, there is a pervasive medicalised culture of treatment until the very end focused on ‘fixing’ the problem. This culture was exemplified by one participant when thinking through the clinical application of the identification algorithm:

‘I think my first reaction, if I see a thing pop up on my screen and say risk of dying in 12 months, my first instinct is going to be “Okay, what can I fix? What am I missing? What things can I work on to improve their health?” Which I think is OK.’

Wickstrom-2020

If the introduction took place during stressful circumstances, engagement decreased, but if the DDSS was introduced in a more structured and calmer period, engagement increased. During summertime or relocation, it was difficult and impracticable to introduce new working processes because of fewer employees and a heavier workload.

Yang-2019

Clinicians said that the DST would only ever be one factor in their decision because of “X factors”; the many factors beyond a patient’s condition that impacts the implant decision. One X factors they spoke of was O/E ratio (observed-to-expected mortality ratio). The O/E ratio is a rating that measures the surgeon and care teams’ performance. Surgeons cared about keeping a high rating. They described the implant decision for high risk patients as “taking on new O/E ratio debts.” This seemed to strongly influence whether they take on another high-risk patient. It seemed to depend strongly on how many patients had recently had poor outcomes. It’s not that we don’t help that [VAD candidate] patient, but if we take this shot and do poorly, then we cannot take on the next 10 patients like him. Because now we got too much of a cluster of high-risk patients who’ve done poorly, then we have to do some lower risk ones before we can go back up [in O/E ratings]. Insurance companies and Medicare and all that... they will mark you. They may not pay. It all plays into the complex factor for deciding who, especially sicker patients, we would take a shot. (Surgeon, B6)
