# Supplementary material for: Stakeholder Perspectives of Clinical Artificial Intelligence Implementation: Systematic Review of Qualitative Evidence
Source: J Med Internet Res. 2023 Jan 10;25:e39742. doi: 10.2196/39742 (PMC9875023; doi:10.2196/39742)
Supplement: Multimedia Appendix 3 [file jmir_v25i1e39742_app3.zip › 6. Wider system/6c. Professional bodies/6c.2 Lack of understanding between professional groups.docx]

**Name:** 6c.2 Lack of understanding between professional groups

Abidi-2018

PCPs were unsure about the professional roles and responsibilities pertaining to the usage of DWISE in a clinical setting. They wondered how doctors, CDEs, and nurses would coordinate and collaborate to ensure that a tool such as DWISE can be used effectively: How would this work...I mean how do we collaborate...should this be administered through a doctor or a nurse educator...who would monitor.

Ash-2015

Clinical customers frequently criticized EHR vendor employees for not having a comprehensive enough understanding of healthcare to be able to develop or productively customize an EHR or reports

Clinical site staff members are often frustrated that they have to put so much effort into even the most basic CDS. Modifying CDS so that it suits the local work process is time-consuming.

Some vendors of both types have difficulty gaining useful feedback from end-users or simply do not try. Several strive to gather informal feedback from customers. One actively solicits feedback through formal customer satisfaction surveys and random spot-checking during implementation.

Customers resent the large amount of work and resources necessary to fit specific content into an EHR

Content vendors also felt that most people did not fully understand what they do, and how resource-intensive the work is

Benda-2020

Participants also mentioned that an implementation toolkit may be helpful as the algorithm expands to new sites to help new adopters make customization decisions.

I’ve learned ... that this closing the loop is what makes the sale ... sometimes, we’re handed a package with the implementation science done. – OPS10 [Facilitator]

Lai-2020

Indeed, as many of the interviewees stated, it is very important to debunk this myth in people’s minds to allow them to have a clearer insight about what it is. Most were speaking about “AI” as if they were speaking of an independent entity rather than a set of various technological applications (tools), thus contributing to the perpetuation of the myth. In addition, the interviews highlighted the fuzzy notion of AI. For example, there was confusion between “weak AI”1 and “strong AI”.2

Yet, to date, no healthcare professional appears to be able to visualize what the AI of tomorrow would really change in his/her practice, and most of the ideas put forward by health professionals were close to the current societal discourse.

From the point of view of the patient association representative, it was also necessary that patient associations, authorities, and industrial partners agree on what AI really means. Indeed, it is very difficult for patients to follow the debates and express an informed view on the subject as long as industry does not adopt a more responsible posture when talking about AI and promise what they cannot deliver to patients (the advantages without the disadvantages).

Liberati-2015

[According to the opinion of medical directors, this positioning is noted, on the one hand, by the absence of an or- management that supports scientific evidence and, on the other, by the dominance of different and contrasting ones professional subcultures, each committed to a defend their clinical approach]

Morgenstern-2021

Several respondents were concerned about the largely unregulated nature of AI and its use in public health.

… a lot of these tech groups operate under the same ethos that, [various companies in Silicon Valley do], which is ‘move fast and break things’.[…]And, in [health], when you move fast and break things, lives are at risk. And so, a worry of mine is that some of these AI groups are going to potentially move too quickly. [Participant ID # 14].

Ruppel-2021

[“You have to see that such a system [the DSM and the ICD, JR] will not be developed because there are convincing scientific results that speak in favor of one or the other approach. Such systems are often the result of a negotiation process between interest groups. (...) And then hard nosed researchers like (Tom) Insel (...) say: ’So I'm sorry for that, and I don't want that, and I'll make my own!’, You can understand that. " (P9: 763-783)]

Watson-2020

The third barrier to clinical implementation of these models was managing expectations since hype often distorted clinicians’ expectations. Independent of the cultural barriers to clinical implementation of these models, there were personnel limitations. The demand for people skilled in the creation and maintenance of these models is significantly larger than the number of people available to work the models. Shortages and turnover of personnel with the requisite skill to develop the model as well as maintain the model created barriers to implementation. One interviewee captured this challenge as follows:

When people with institutional knowledge move on to other institutions and their institutional knowledge is particularly targeted on the machine learning models that are in production, that creates a knowledge gap and also a sort of responsibility gap that must be filled by someone if these are to be continued.

Yang-2019

It took even longer to explain it to clinicians with statistical depth and ML experience. They fixated on the fact that the ML systems’ performance was not the focus of our assessment. The synthetic patient data often turned this into an assessment of the DST’s quality in the minds of many meeting participants.
