# Supplementary material for: Stakeholder Perspectives of Clinical Artificial Intelligence Implementation: Systematic Review of Qualitative Evidence
Source: J Med Internet Res. 2023 Jan 10;25:e39742. doi: 10.2196/39742 (PMC9875023; doi:10.2196/39742)
Supplement: Multimedia Appendix 3 [file jmir_v25i1e39742_app3.zip › 6. Wider system/6d. Socio-cultural context/6d.1 Public reactions to tools vary.docx]

**Name:** 6d.1 Public reactions to tools vary

Abejirinde-2018

“It was because I had never seen it and that was my very first time of seeing it, so I was afraid

“I was excited because I was hopeful that if I have any sickness in my system, the machine will let me know.”

“I wasn’t happy and wasn’t afraid as well, I just knew it was part of the care they were providing”

“I thought using the box is only when it becomes necessary. So whether or not she uses it, it is the same… They are the professionals and know the best for me. As for me, I cannot tell.”

“That, because after she said she was going to use it for this particular thing, when she was doing it, I was eager and ready to see whether I belong to any of the diseases she has mentioned or I’m free, so I was just waiting to see what the machine will tell me”

most women were confident with the screening results and a couple of respondents noted that they would not have believed a referral recommendation if it had not come from the device.

“

However, there were a few reports of women who while acknowledging benefits of the device, did not necessarily trust its decisions or recommendations. In negotiating this digital trust, educated respondents were found most likely to critically reflect on results and recommendations from B4M

Adams-2020

Initial perceptions of AI by some participants reﬂected fear, and another participant described AI as “scary—the unknown.”

For some, this represented a lack of trust in AI, and for others, there was a willingness to trust outputs from AI to obtain the most accurate information possible. Participants identiﬁed an inherent battle of where to place trust, and participants recognized limitations of radiologists and AI tools: “Human brain versus computer brain: both can make errors. It’s equal.”

Some participants expressed concern that AI is at odds with cultural ways of knowing and healing.

Biller-Andorno-2021

AI systems are innovative and have immediate appeal (‘exciting stuff’). ‘I think such tools, that’s actually very exciting stuff, I must say.’ (Interview 9)

Blease-2019

The question is whether they will be acceptable to patients although they may be very accessible compared to the current system. [Participant 88]

Collard-2020

Doubts about accuracy. Although many participants anticipated a benefit of the system would be to automatically adjust dosing based on exercise, confusion remained about what the system could offer in these terms. ‘Sometimes during sports I wouldn’t trust it… Or if I had a low I probably wouldn’t trust it. For sports, sometimes I’ll get huge spikes, and it’s from adrenaline not from something else, so I’m afraid it might correct for that, and then just drop me down in the middle of my hockey game or something and I’d get really sick.’ (Adult, Focus group.) For those who already had experienced using an automated insulin delivery system in clinical trials, this was shown as one of the results of having used the system. ‘I deliberately didn’t do any major walking or cycling on the weekends while I was on the system because I just didn’t want to risk having a bad hypo in the night.’ (Adult, Focus group.)

Fan-2021

We found that users usually expressed dissatisfaction when they believed that the diagnostic suggestion was not accurate; as one user stated, “I’m 21 years old with a regular lifestyle. I don’t smoke or drink. I eat and exercise regularly. You told me I have diabetes!!!”

Some users even compared the diagnostic suggestions of DoctorBot with their physician’s diagnosis and stated that there was a discrepancy: “My doctor told me there is nothing to worry about and prescribed me an herbal medication. But the chatbot suggested a different diagnosis and I don’t trust it.”

Goetz-2020

The students also believed that patient compliance would be lower when using a vPCP.

“[I]f it were to write prescriptions, maybe I wouldn’t take them, who knows. Like, it wouldn’t be as serious to me as a real physician. . .” (First year graduate student)

Haan-2019

Furthermore, patients acknowledge that humans also have flaws and may therefore not always perform optimally when doing their job. Pat

Patients report that they are unsure about the skills of a computer. They value the experience of the radiologist

Horsfall-2021

the controversial reliability of software or data input (11/33; 33%)

Kendell-2020

An EMR-based algorithm was viewed as another tool available to physicians that could assist them in their clinical decision-making.

“I personally think it’s a great idea. I don’t have a problem with it if it means that the doctor’s going to discover sooner that there’s an issue with my health.” [Ontario, Participant #8]

Knoble-2015

The HCW e-algo users overwhelmingly reported that their patients’ views of the application were positive. They reported that the patients appeared quite comfortable and positive with them using the tablet because it required more time than a traditional visit. HCWs stated that patients had conﬁdence that the “computer” would give the correct diagnosis.

Lawton-2014

With the exception of those who highlighted very poor mathematical skills, participants, at baseline, described undertaking their own mental or manual calculations

alongside using their advisors, because as P4 pointed out, ‘‘I don’t want to rely on something to do the maths, I try to work it out myself ﬁrst and then just check it against the wizard’’ (P4.1), or, as P43 suggested, because, ‘‘I don’t quite trust it yet, won’t accept what it says for truth sort of thing. I always make the measurements in my head, just to be sure it’s right’’ (P43.1). In general, participants claimed to agree with, and admin ist he recommended doses. However, several highlighted occasions when they had made slight adjustments to take account of planned physical activity or because the blood glucose targets they were aiming for were higher or lower than those programmed in during their courses:

‘‘If it gives me 6.5 and I think to myself I’m going to go and sort the shed out and all that, 0.5 might be a bit too much. So I tend to, I might take 6, and I think well, if it’s a little bit higher in an hour and a half, I can do a correction.’’ (P26.1)

‘‘The only time I’ve not taken its advice has been at bedtime when I’ve been high and it’s told me to take 2 units, I’ve taken 3 or even 4 because I’m not worried about having hypos.’’ (M10.1)

Lennox-Chhugani-2021

many women, who had a negative or mixed view of the effect of AI in society, were unsure of why they felt this way (n=96). However, they described AI as an inevitable part of their lives in the future (n=20).

Concern about the reliability and safety of technology (n=123).

2. A lack of trust in the technology itself or the systems that sit around it (n=65)

Many of the women who took part expressed the view that the use of AI in healthcare and specifically in the breast screening programme was inevitable.

McCradden-2020

As a member of the public, my opinion doesn’t count. (Participant 18–004, patient)

Morgenstern-2021

They stressed that it is not so different from traditional statistical methods and is subject to similar limitations, which is often not fully appreciated.

You can think of AI as just […] the next set of tools in statistics. Yeah, we moved beyond t-tests and we have convolutional neural networks now but the basic principles […] are fundamentally the same … [Participant ID # 4].

Sun-2019

They [the patients] have no idea about Watson. They will think: why do I need a machine to look at [my problem]? I prefer an expert doctor” [1HP04]

Torenholt-2021

The public servant responsible for the workshops was very attentive to this tension:

She [the public servant] says to the group that she has asked in the ministry, and they have ensured her, that this is not a ‘money-saving exercise’. ‘It is to help prioritise – some patients will need more [clinical visits], some will need fewer, she says to the group. (Fieldnote).
