# Supplementary material for: Stakeholder Perspectives of Clinical Artificial Intelligence Implementation: Systematic Review of Qualitative Evidence
Source: J Med Internet Res. 2023 Jan 10;25:e39742. doi: 10.2196/39742 (PMC9875023; doi:10.2196/39742)
Supplement: Multimedia Appendix 3 [file jmir_v25i1e39742_app3.zip › 6. Wider system/6d. Socio-cultural context/6d.2 Culture's effect on tool acceptability.docx]

**Name:** 6d.2 Culture's effect on tool acceptability

Adams-2020

Many participants’ initial perceptions of AI were shaped by popular media and science ﬁction. For example, one participant responded that “[AI reminds me of] movies right away.”

Participants were willing, and many eager, for their data to be shared to assist in the development of AI tools in health care.

Collard-2020

Physical appearance and comfort. In discussions regarding specific situations where participants might want to disconnect an automated insulin delivery system, some participants – mainly teens and children – argued that they would disconnect it to prevent someone seeing them wearing it during exercise or sporting events. ‘I probably wouldn’t want to wear it when I exercise and do sports, but I don’t really have a choice in that. That’s the main time of movement where I wouldn’t want to wear it.’ (Child, Focus group.) Most participants stated that physical appearance was not a big issue for them. However, some argued that they would not wear an automated insulin delivery system if the nature of the exercise or sporting activity prohibited comfortable usage – for example: ‘When I’m doing sports, it would be cool if I’d be able to remove it. Like with my insulin pump, I could unclip it.’ (Adolescent, Focus group.)

Gance-Cleveland-2019

Other comments suggested that the substance use and IPV questions were important questions to ask patients. One commented, “I am glad you asked about substance use, we need help when we are pregnant.” Another patient commented, “No one has asked me about this [IPV] before, this is important to ask.”

One participant stated, “The tablet-based screening is a better way to ask about [IPV] because in my culture we are not allowed to talk about it, but we can answer the question on the tablet.” Us

Goetz-2020

Students felt that using a vPCP may reduce bias or discrimination. A few students gave personal stories of instances where they have felt stigmatized while using medical care.

“. . . there are certain stigma or certain assumptions people make based on how they look. . . You don’t have to deal with that when you’re dealing with a tool.” (First year medical student)

The groups thought that the lack of human contact might make it easier for patients to

speak about stigmatized or “embarrassing” things, such as sexually transmitted infections and mental health issues.

“And in many cases, you won’t feel like sharing what you have, what you’re going through with another person. So, in that case, for maintaining your confidentially or secrecy. It would be better to be with the machine. . .maybe if you have a machine you can talk, you can tell what you’re going through.” (First year graduate student)

Keogh-2019

Many commented on the overwhelming number of women interested in genetic testing and risk assessment following Angelina Jolie's 2013 disclosure of her BRCA mutation status and subsequent risk-reducing bilateral mastectomy and bilateral salpingo-oophorectomy,

Lai-2020

For most of the individuals interviewed, the perception of AI benefits, in part, from its representation in popular culture. There appears to a collective fascination with AI.

Moreover, physicians mentioned that there is a discordance between scientific advances and the thundering announcements made in the media. The buzz, particularly generated by some companies, does not correspond to the reality of operational technological advances, which diverge highly from what is currently experienced in hospitals.

Talking about the future role of physicians, the position of the industrial partners was not always clear, depending on the medical specialty. Concerning medical imaging in particular, it was clear to them that AI tools could replace radiologists, but that such replacement will not happen for a long time because society is not yet ready to accept this type of medical care.

All the participants without a conflict of interest admitted they were influenced by the discussions surrounding the subject. Thus, society appears to have certain preconceived notions, the most widespread being that “more automatic” was equivalent to “more secure”, which is questionable

A major reproach of health researchers in AI was that the kind of AI media were talking about had nothing to do with the kind of AI they were working on, which has a much more specific and narrow definition.

McCradden-2020

Patients and caregivers reported a high level of trust in health care institutions with regard to ethical practices and acting responsibly vis-à-vis health data by following regulations designed to protect the public. When asked about a duty to participate in research specifically through allowing use of their health data, a few participants stated that people had a duty to allow such use for the specific purpose of researching health-related problems, whereas others indicated no one had such a duty. Nearly all participants who did not express a yes or no answer indicated that they personally felt a sense of duty to contribute their data to research but that not everyone would agree, and individuals’ wishes should be respected. Others described a duty only if the research involved deidentified data and no potential harms to participants.

Despite a perception that data sharing is now inevitable, most participants clearly indicated discomfort with the lack of transparency regarding how their data were being used.

Morgenstern-2021

the public are a lot savvier than we give them credit for and people are very good at distinguishing between corporations use of data for […] targeting advertising […] versus […] medical and scientific use of data to improve peoples’ health. […]We often are afraid that we’re going to be painted with the same brush that people paint […] [social media companies or political consulting firms, thinking that] if we use anything to do with that and if we even say the word data people are going to get really angry. [Participant ID # 6].

Patel-2018-additional file

I think also having an indigenous person as a health worker who only help maybe on that cultural side to say, well this is what this means, and it will be really handy in screening ‘cause not all our patients want to go through to a doctor.

Sun-2019

Second, there is a perceived societal misunderstanding of the cap-

abilities of AI technologies in the public healthcare sector. On the one hand, the general public is seen as lacking knowledge on the values and advantages of AI. As mentioned by the CEO of CognitiveCare: “this is a new thing. And most of people don't know the advantages of AI.

On the other hand, society has overly high expectations from AI, which leads to difficulties in the acceptance of AI technology by doctors in the hospital. With the introduction of AI policies in China, social media and organizations began to talk about AI frequently, attributing “magic” qualities to AI technologies, often leading to disappointment in doctors using the IBM Watson system

In addition, attitudes towards how to tackle a disease are different. As remarked by one of the hospital managers/doctors:

[For the treatment of cancer] Chinese patients think surgery is better. […] In the West, as a tumor can stay within the body for a long time, there is a greater focus on the importance of the management of cancer as a chronic disease. [In China] patients do not see it this way [as a chronic disease]. [1HP01] This difference in medical approaches and practices is not taken into account by Watson, and this is seen as another key challenge
