# Supplementary material for: Stakeholder Perspectives of Clinical Artificial Intelligence Implementation: Systematic Review of Qualitative Evidence
Source: J Med Internet Res. 2023 Jan 10;25:e39742. doi: 10.2196/39742 (PMC9875023; doi:10.2196/39742)
Supplement: Multimedia Appendix 3 [file jmir_v25i1e39742_app3.zip › 6. Wider system/6e. Interorganisational networking/6e. Interorganisational networking.docx]

**Name:** 6e. Interorganisational networking

Ash-2015

The clinical organizations need analysts with both technical skills and knowledge of healthcare to customize CDS. Content vendors need clinicians with skills in evidence-based medicine and writing; EHR vendors need physician consultants who help train staff within purchasing organizations to manage CDS and use it. The notion of embedded employees and the need for vendor staff members to understand healthcare issues are two special points of interest at the intersection of the three “workforces”.

The allegiances of embedded employees are sometimes more towards either the clinical site or to the vendor; these staff members often feel conflicted.

CDS analysts at clinical sites are therefore especially delighted when they can deal with vendor staff members who have clinical backgrounds. Physician consultants working for EHR vendors are highly valued by physician customers. Those representing healthcare organizations felt strongly that vendors should hire more staff members with clinical backgrounds.

Representatives of all groups thought that sharing of CDS would be an ideal but hard to reach goal.

Collaborative communication between clinical site employees and vendor employees ranges from daily to infrequently. Some have good, close working relationships, while others experience friction. Clinical staff members who manage to find individual vendor staff members to call on a consistent basis believe that is key.

All three content vendors point to the need for open dialogue between content vendors, EHR vendors and end users.

All three groups believe the other groups do not understand what they do. Clinical sites do not understand content vendors because they do not know how much effort goes into developing CDS content; content vendors do not understand the burden on the clinical sites to modify the content; EHR vendors do not understand how difficult it is for content vendors to integrate their products into EHRs that follow different interoperability standards.

The vendor representatives’ view of customers is mainly positive in that they admire the hospitals and clinics for being at the forefront of EHR implementation.

EHR vendors tend to have relationships with specific content vendors. Although it may be theoretically feasible for a customer to use a different content vendor than one that is partnered with their EHR vendor, it is often both more difficult and costly.

All vendors talked about how they define “the customer,” the varied and complex relationships they have with these customers, and the differences in expectations and approach required by each. Customers include EHR vendors that are customers of a content vendor. Health systems, hospital IT departments, and end-user clinicians may all be customers of either content vendors or EHR vendors.. To make matters even more complicated, agreements and types of relationships often vary even within a single customer category.

Ash-2020

So, she (an IT person with a clinical background] really bridges that gap around just making sure because when IT folks talk directly to the front line, sometimes there’s just the language barrier there.

Cameron-2017

The idea of a tool was viewed as a way of potentially improving communication and transparency between physicians and transportation administrators, and addressing some of the identiﬁed barriers.

Chirambo-2019

We identified that currently there are stakeholders in place who partner with the government to run the programs. We also found that there is good collaboration between the public sector and the NGOs which are implementing the program. However, some government officials called for a continued and strong collaboration between the government and the implementing partners.

“There is good partnership between the government and the NGOs which are involved in implementing this mHealth decision-making tools program.” PSA-3

“There have been several planned meetings between the implementing partners and the District Executive Committee which comprises of different government and NGOs in the district.” PSA-16 “In some meetings, the traditional leaders are invited to attend the DEC meetings so that they can be updated on programs being implemented in their communities.” PSA-1

The government on its own cannot manage to fund this program. Just buying phones on their own it’s very expensive so what about phone replacement when stolen or broken? This means there is a need for continued collaboration between the government and the implementing partners. PSA-17

Cresswell-2019

While no participant expressed an overt negative opinion on the concept of DSS in principle, early and ongoing engagement with clinicians and other healthcare staff was viewed as crucial during system development and implementation. Participants were positive towards the concept of DSS, but had some concerns about how this would work in practice. At one workshop, a male GP emphasised that his comments should not be seen as negative but ‘in the spirit of improving the system’. So their forward travel seems to be in the right direction providing that…the final product will work as it’s supposed to work…we’ve got 10 min. That has a big impact on your time whereas if you’ve got something popping up that can do these things then it should work far more efficiently. (Participant 20, female, GP, Tayside)

Participants wanted to get ‘hands-on’ experience of using the system and be engaged in ongoing development to ensure that it worked in practice as well as in theory. Ongoing stakeholder engagement was also stated to be necessary to raise the profile of the Platform among the wider healthcare workforce.

The actual workshop…was a good step in the right direction, but I think for people who aren’t aware of these things, not just GPs but pharmacists, nursing and other kind of clinicians, I think a wee bit of work needs to be done to raise its profile. I really enjoyed looking at the screens and saw great potential. (Participant 27, female, GP)

Klarenbeek-2021

Included hospitals had close collaborations with other hospitals. Meaning that if a

second opinion was requested by one of the other hospitals, patients were discussed by use of video-conferences during MDTMs. Therefore, it is necessary to involve external partners in the implementation process in order to make sure the CCDSS could be adapted to all types of commercial EMRs.

Lai-2020

Indeed, and unlike physicians, those in industry did not appear to see why it would be meaningful for a physician to understand how a new AI tool works. Sometimes during the interviews, they mentioned mere superficial learning. For example, they believed that radiologists were mainly “here to push a button”.

Liberati-2015

[The electronic medical record is not good in the solute. It depends on who does it and how they do it. Today if engineers don't talk to clinicians, they don't know what clinicians need, because they don't know no how we work. They cannot force us to work. rare in a different way from how we work ». (Surgeon orthopedic, setting C)]

[Some IT directors finally stress the importance of maintain an open and constant dialogue between IT specialists and clinical community in order to foster reciprocal adjustments between technology and practice clinic. ]

[In addition, IT staff may see direct a few insights offered by professionals al order to achieve greater sophistication e system customization, responding to peculiarities of its various users (different disciplines pline, different healthcare contexts, etc.).]

[The technological and information evolution of SSDCs, a constructive dialogue between clinicians and IT staff e a greater habit of using the system in the practice can encourage a correct and complete adoption of SSDCs.]

["I would like the doctor to become a little more ingenious. computer engineer, and the computer engineer becomes taxes a little more doctor ». (Orthopedic surgeon, Sept-ting A) ]

Mozaffar-2016

The shortage of human resources seemed to arise because of two related underpinning factors. First of all, the particular employment practices in the health sector acted as a disincentive, discouraging individuals from taking up short-term contracts. It also dissuaded health professionals to use their newly gained expertise to change professional rout and become HIT consultants. This meant their expertise was barely transferred for implementation of HIT project in other health organizations.

It would be quite common [in other sectors] to have independent consultants to advise a procurement in the private sector… We did have two people […] who’d gone from being implementation experts to being supply side players but is there a class of people who’ve implemented [CPOE/CDS] systems who then serve as [CPOE/CDS] consultants? If not why not, is it because you can’t pay for them? (Business Case Workshop, Participant 10)

One of the biggest dependencies for us is the readiness of the software and that is being driven by [another hospital name]… there is the critical dependency on delivery to [other hospital name] but leads to our project very directly so clearly where [other hospital name] was delayed that potentially causes delay to our work … They delayed their go live… we have a dependency on the same release of software. (Site E, Senior Project Manager)

Another type of tactical delay caused by external entities was caused by suppliers’ strategies in responding to user needs. In this respect, suppliers had “sorting and sifting”

strategies in order to prioritize and plan for their future developments.

…if we need something to happen we have to log it with a user group who then have to agree it’s something we want to take forward which then goes to the vendor, they decide whether they can be bothered to do it, it then gets on their work plan and maybe three years later we get our change… (Site A, Pharmacist)

Suppliers prioritized the needs (including functional needs and resource needs) and even the customers, based on internal strategies which in turn led to late response to some of users’ requests.

Anything which is dependent on [supplier name] takes months which is not ideal and we’re also limited by when they can put the changes in so it can be quite difficult. (Site F, Consultant)

There were also delays caused by contractual issues enforced by the supplier. Suppliers sometimes introduced ‘freeze’ periods in the contracts which meant that no change requests could be put in for a certain period of time.

Nova-2020

would have to have assurance: that Home Gare would act on the information that I sent them (Dr C)

Petitgand-2020

Developers argued that physicians did not have enough opportunities to read medical histories and learn how to integrate them into their clinical practice. However, according to nurses, this did not really explain why physicians were not becoming "early AI adopters":

Every time it doesn't work, they say it's because nurses didn't print the history, or didn't direct them [patients] to the tablet, or because... "Wait a minute, you, [developers], what have you done to change our processes? What have you done to improve the medical history? What have you done to innovate?"[...] Since, it's something to help physicians, they need to play their part. (Nurse 2)

Petkus-2020-supplementary file

“Important for the user to be in contact with the provider so that the provider can update the software if problems are identified. “

Sun-2019

Organizational and managerial challenges are framed as highly relevant by government policy-makers and IT firm managers, but not by hospital managers/doctors.

AI adoption in healthcare needs staff with interdisciplinary knowledge both from technological and medical disciplines. Both government policy-makers and IT firm managers claim that in China there is an insufficient number of such inhouse staff. As remarked by a government official:

Now the in-house talent is scarce. We can learn from [Chinese big IT firms] B.A.T. [Baidu, Alibaba, and Tencent]. Most of their personnel have come from abroad. […] AI experienced three ups and downs, and China used to develop AI talent. [When AI experienced a down] people withdrew from the industry, […] universities quit developing AI talent. So, the shortage of AI talent is severe. [5GOV01]

These technological challenges include the lack of transparency of AI algorithms and the difficulties of the AI system in processing unstructured data. It is interesting to notice that both these technology-related concerns are voiced by either government policy-makers or by hospital managers/ doctors; IT firm managers do not frame any technological challenge as relevant in the adoption of AI in healthcare.

As one of the interviewed government policy-makers observed “[regarding data challenges,] if the government keeps the data and works on this issue, the efficiency will be too low. We [the government] need to involve the IT firms with managerial skills and professional technology to work together with us. We [the government] will take the responsibility of supervision” [5GOV01].

Watson-2020

The institutions with greater success building clinically-implemented models incorporated clinicians and other stakeholders into the full development cycle of the model. The clinicians were key to identifying the clinical need, informing the model’s development, and helping determine the optimal places for intervention while minimizing disruption to workflow. Perhaps counterintuitively, one interviewee explained that this collaborative approach was more time-intensive and resource-intensive than the technical challenge of building the model. However, the interviewee explained, it is exactly this collaboration that is one of the biggest determinants of whether a model will be successfully integrated into clinical practice. This interviewee said,

...more of the work is actually going be focused on the intervention and the program to support that intervention in a sustainable way. The tech part, the IT part is, and the analytics part is getting easier and easier you know. They can spin that up and figure that out over a matter of maybe a week or a few weeks.

Some informants reported working with their EHR vendor to clarify methods used to improve transparency into the inner workings of the model.

Another concern noted was that data exists but does not represent the reality of clinical care. For example, one interviewee stated:

There’s always enormous work in a project to identify a candidate data [element] that would be useful and then to validate that it’s clinically meaningful and relevant. There [are] many, many times where we get started building a model where we pull out data that looks meaningful and carries a lot of signal, and [when] we talk to the clinicians, that data field is laughed at. They don’t use it, or it’s just something that they have to enter because of billing reasons, and they are horrified that we would actually use that in a model and there’s no getting around that.
