# Supplementary material for: Stakeholder Perspectives of Clinical Artificial Intelligence Implementation: Systematic Review of Qualitative Evidence
Source: J Med Internet Res. 2023 Jan 10;25:e39742. doi: 10.2196/39742 (PMC9875023; doi:10.2196/39742)
Supplement: Multimedia Appendix 3 [file jmir_v25i1e39742_app3.zip › 7. Embedding and adaption over time/7a. Scope for adaption over time/7a.1 Improvement of technology and its implementation.docx]

**Name:** 7a.1 Improvement of technology and its implementation

Abdi-2021

These technologies are also expected to improve in the future as underpinning technologies (e.g., natural language processing and AI) continue to develop

Similarly, many experts agreed on the potential of portable diagnostics to facilitate older people’s access to healthcare as this technology is expected to mature in the next 10 years

“Some elderly generations are not tech savvy. Nevertheless, the improvements in user experience should increase the popularity of the mobile device use for health care purposes” Portable diagnostics, P20

Adams-2020

In the context of AI, one participant noted that it is important that AI tools continue to improve as additional data are available.

Alagiakrishnan-2016

Physician workflows, although initially disrupted by early study interventions, were less disrupted by iteratively optimized SMART CDS. Interviewed clinicians described the messages were becoming more user friendly, simple to read, and easy to navigate

Ash-2015

The vendors are all striving to improve workflow by providing more active decision support that is embedded within the EHR so that the clinician does not have to ask for it separately

Interviewees representing this vendor also talked about involving stakeholders in developing requirements for designing and developing CDS. They also monitor customer use of the product to make decisions regarding future content.

The EHR vendor representatives noted that their organizations were paying a great deal more attention to usability than ever before. One of them said: “Well, you know, I mean certainly in the past four or five years the user interface, user experience type people have become more in demand by vendors and that’s probably going to continue to grow as we hear more noise about usability”.

Ash-2020

We think it’s going to be huge to have the patients actually type in why they are there.

Blease-2019

The somewhat blunt tool of technology as it stands will need to evolve some way before the culture of clinicians and patients will accept it. [Participant 453]

Dikomitis-2015

Despite the danger of an overload of prompts, most of our participants perceived a future for electronic aids for GPs within the changing context of primary care. Most issues highlighted were germane to new interventions implemented into general practice, rather than to this particular intervention: multi-tasking expected from a GP within one consultation; constant time pressures; and the possibility of medico-legal repercussions:

‘There is, I suppose, the challenge of more and more prompts that, you know, say for us, your prompt box will get bigger and bigger. However, work in general practice is getting bigger and bigger, and more work’s going to come to general practice. So although it can cause irritation, the ﬂip side of that is you can relax a little bit more and that you don’t have to remember absolutely everything (…) You know, how to sort the wheat from the chaff. So although people might think it’s irritating, it can be reassuring’.

Gillan-2018

This was reiterated by a treatment planner, suggesting a shift in focus towards adaptive treatment, ‘But what happens tomorrow? And the second day? And the third day? That’s where your planning skills would shift, in introducing adaptation. Not the initial plan’ (TP01)

Goetz-2020

Students discussed the importance of a dynamic system.

. . . (if) the system is very open source and, like, doctors and stuff can keep putting information in there, constantly, then it might be a very robust virtual physician.” (First year graduate student

Lai-2020

Nevertheless, all possibilities of working with AI tools envisaged during the interviews were based on ongoing research. Healthcare professionals then pointed out that few of these projects have yet proven to be successful in real life and it was clear to the interviewees that AI is still in its infancy.

They appeared to be confident about the progress in their research, regretting the too-slow translation from research to practice, even if working with healthcare industry was seen as a way to accelerate the translation.

Morgenstern-2021

In addition to automating existing screening programs, AI may make entirely new forms of screening feasible.

I think the cost of these technologies is going to continue to come down. So, I don’t want to get too sci-fi, but you could imagine a future where everyone’s got a device of some type. […] So cheap that the government actually provides everyone with a personal health monitor.

the interpretability of machine learning algorithms is widely considered to be an issue. Many AI methods, especially deep learning, are considered inscrutable. However, some participants thought that these issues could be overcome.

I mean the two hottest research areas in machine learning and AI right now are explainability and causal reasoning. So, I think there is a huge demand from many, many, many perspectives to be better able to explain those things and to be, um, you know, to be able to learn causal association. Or causal, you know factors. […] My sense is we’ll get better at it. I don’t know if it’ll ever get perfect with the really, truly data-driven methods, but I think we’ll see big improvements in the next few years. [Participant ID # 3].

It was suggested that AI approaches could be trained to reduce bias, and that while bias is a risk it could be overcome with more research (seeAdditional file 6).

I think it’s easy for people to say well that data is garbage and that is garbage. I think we need to get more nuanced to say when, what measurement error counts when and where? [Participant ID # 12].

Morgenstern-2021-supplementary file 6

I think they're pretty close to the point where reading pap smears can actually just be replaced by the AI altogether. [Participant ID # 7]

Page-2019

Another common lesson learned was that a structured evaluation and monitoring program for EMM medication alerts is critical for continual reﬁnement of alerts.

Patel-2018-additional file

PM: I’m just really, really pleased that you’re continuing for another 12 months, because I think for the first six months of the project no one got it right

Van de velde-2018

The system should not be incomplete when it is implemented, because then it will not be a practical solution to the user; The system should be continuously improved. [Patients, Finland]
