# Supplementary material for: Stakeholder Perspectives of Clinical Artificial Intelligence Implementation: Systematic Review of Qualitative Evidence
Source: J Med Internet Res. 2023 Jan 10;25:e39742. doi: 10.2196/39742 (PMC9875023; doi:10.2196/39742)
Supplement: Multimedia Appendix 3 [file jmir_v25i1e39742_app3.zip › 7. Embedding and adaption over time/7a. Scope for adaption over time/7a.2 Normalisation of technology and decreased resistance.docx]

**Name:** 7a.2 Normalisation of technology and decreased resistance

Abdi-2021

However, it is important to note that some experts thought that older people’s access

and acceptability of some technologies (e.g., smartphones) are expected to change in the near future

Abejirinde-2018

“they develop some love in it and every time they come, even if it is wasting time, they will still wait”- an indication that women may have also adjusted their expectations and factored in the extra time needed for use of the device.

all, except two health workers, felt that the use of B4M in their health facility created a demand for ANC services

Health workers attributed this perception of a positive pull effect to women spreading news about the device within their communities, especially following a testimony of improved wellbeing after adhering to the B4M advice.

“When they saw it the first time and those (I screened) and told them their problem, they were telling their colleagues- ‘Oh I went to the hospital, they are now having a machine’. So everybody wants to come and see the machine.” -HC

Ash-2015

Content vendor representatives felt that because of Meaningful Use there is an emerging understanding of “what [they] do” on behalf of both the public and customers, and that this would lead to greater customer appreciation.

One vendor has offered free analytics capabilities for a number of years, yet few customer organizations have used them. However, interest among customers is increasing rapidly. An EHR vendor representative said: “many practices are interested in quality measures because of reimbursement, but many are also interested in improving their care

Haan-2019

Most patients think that using Al in radiology is a development that will happen, but not in the near future.

Henshall-2019

All psychiatrists felt that the DST could be utilised clinically, both in cases of first-episode psychosis and for long-term service users, as a monitoring and review tool. They felt that the more they used the DST, the more likely it would be to change their clinical practice. I imagine that if it is something that is very routinely used … I’d like to think somebody like me would make changes over time. Not within a week, 2 weeks, but maybe over a year. Psychiatrist

Knoble-2015

Each was followed up with another phone interview. The primary objective was to determine if and why usage of the e-algo went down during the non-compulsory phase of the study. All admitted that they had not used the algorithms as before. Three of the nine did not use it at all, the other six reported reduced usage

Lawton-2014

Participants with poor mathematical skills reported an ongoing and heavy dependence on their advisors at 6 months, a situation which became apparent when equipment broke or was misplaced, with individuals reporting being ‘‘absolutely at a loss’’ (M14.2)

Amongst other participants, there were notable changes in how they used their advisors over time. While some (n = 6) continued to do their own calculations, the majority, like M13, who had initially ‘‘double-checked everything’’, discussed how, ‘‘lately I don’t do any calculations at all’’ (M13.2). As these participants suggested, having access to technology which was fast and easy to use had led to their becoming ‘‘lazy’’ (P39.2) and to their administering the doses recommended by their advisors in increasingly unreﬂective and unquestioning ways:

‘‘It probably does make you lazy, because I don’t really have to think about it much, you know, you can do your blood and then put in what you’re having, have a quick look at it and not really think any more about it.’’ (P39.2)

‘‘I’ll click it and I enter the amount of carbs and that’s it, I just go with whatever the wizard tells me, without really thinking about it.’’ (P31.2)

By virtue of no longer doing their own calculations, participants also described how easy it was to forget what their ratios actually were: ‘‘the only thing I could forget is the ratios of DAFNE, but the machine knows that’’ (M9.2); ‘‘they’re not in my mind but they’re programmed into the machine’’ (M 9.2). Hence, these participants had inadvertently become dependent on their advisors to determine their doses for them.

Furthermore, whilst during their courses, participants kept a manual (diary) record of the blood glucose readings and other data (e.g. carbohydrate portions consumed) and received training on how to review these data in order to adjust their ratios and other parameters, many described manual record keeping as burdensome. Hence, the majority (n = 27) reported how, over time, they had taken advantage of, and become reliant on, the data storage facilities on their advisors: ‘‘I’m basically now entering everything into the advisor rather than writing stuff down’’ (M13.2). However, use of this automated feature, as such participants further reﬂected, also resulted in them reviewing their data less frequently, and sometimes not all: ‘‘I haven’t looked at the data really’’ (M13.2); ‘‘Um, I’m relying too much on the meter’s memory for that rather than making a record and going through it, trying to ﬁgure out patterns’’ (M16.2). Hence, it was not until a review appointment was attended that individuals, such as M13, actually recognised that there was problem with their blood glucose readings which required a ratio or other parameter to be changed:

‘‘since the course I’ve never once set down and looked at the data myself so the meeting [6 month follow-up] was the ﬁrst time I actually saw the data, and things were. . .the ﬁgures weren’t as good as I anticipated.’’ (M13.2)

Miller-2019

Many providers commented that although integration would likely meet resistance at first, people will “come around eventually.”

Patel-2018-additional file

GP: …eventually I’ll get to the stage where I’ll want to use them also for my resources and guidelines as well. It’s just a matter of time, just get used to it because, you know, just once I get the hang of it and it gets more useful I guess I just need to expand what I’m using, because I guess it can offer me so much more than what I’m using it for.

Vedanthan-2015

Overall, nurses found DESIRE to be initially challenging to learn but, after one month of use generally preferred it to paper forms and would strongly recommend it to their colleagues. Participant : okay just like she has said, I thought it would really give a lot of hard time, going through the paper work then the tablet, but with time we've found it easier to use the tablet than the paper work.

Participant: I think it's not a complicated thing; yes it's easy to use it. Participant: with time it will be easier to use the tablet rather than paper work.

Participant: ... as time goes by after using it, it build[s] confidence in me because when you are using it, it leads you to the answer, like if the patient is hypertensive, like the pressure is this, if you insert like type the blood pressure for that day, it will automatically tell you whether to refer or manage, so confidence.

Watson-2020

There was significant variability in the maturity of ML applications across the informant AMCs. There appeared to be near unanimous consensus that the landscape is evolving rapidly,
