# Supplementary material for: Stakeholder Perspectives of Clinical Artificial Intelligence Implementation: Systematic Review of Qualitative Evidence
Source: J Med Internet Res. 2023 Jan 10;25:e39742. doi: 10.2196/39742 (PMC9875023; doi:10.2196/39742)
Supplement: Multimedia Appendix 3 [file jmir_v25i1e39742_app3.zip › 7. Embedding and adaption over time/7b. Organisational resilience/7b. Organisational resilience.docx]

**Name:** 7b. Organisational resilience

Ash-2015

They aim to be prepared for and able to take advantage of changes in federal policy. Interviewees from both EHR companies expressed the idea that having a robust data analytics capacity was a main selling point for their respective companies.

There is a history of content vendors being bought by EMR vendors and later becoming separate again [25-28], so in these cases there remains some interoperability. One content vendor representative recommended: “The optimal way for clinical content and EHR vendors to work together is to keep separate, but standardize the interfaces between them”.

Mozaffar-2016

Change of suppliers’ organizational structures which was a direct result of acquisitions, was another cause for delays in the implementation process.

Unfortunately for us when I raised the RFC [request for change] a company called [company name] came in and took over [company name- original supplier] so there was a corporate takeover and that delayed the process. They’re an American company and they needed a PAS system for the UK market so they purchased [company name- original supplier] but obviously that resulted in a five to six week delay whilst we got the request for change result… (Site E, Senior Project Manager)

Such changes could lead to contractual modifications

or even strategic change of the application which in turn caused further delays.

Lack of contract signing [by the new supplier] and unwillingness to continue to work at risk – this will likely end up in adding 2–3 months to the timeline… Issue between [supplier 1 name] and [supplier 2 name] contracts preventing deployment of software on existing hardware… (Site E, Project Document)

Shannon-2021

additional training to be prepared to carry out the study procedures. More broadly, administrators have questions about the long-term sustainability of the study. What will happen after the 5-year study period? Will insurance cover the cost of the program?
