# Supplementary material for: Stakeholder Perspectives of Clinical Artificial Intelligence Implementation: Systematic Review of Qualitative Evidence
Source: J Med Internet Res. 2023 Jan 10;25:e39742. doi: 10.2196/39742 (PMC9875023; doi:10.2196/39742)
Supplement: Multimedia Appendix 5 [file jmir_v25i1e39742_app5.pdf]

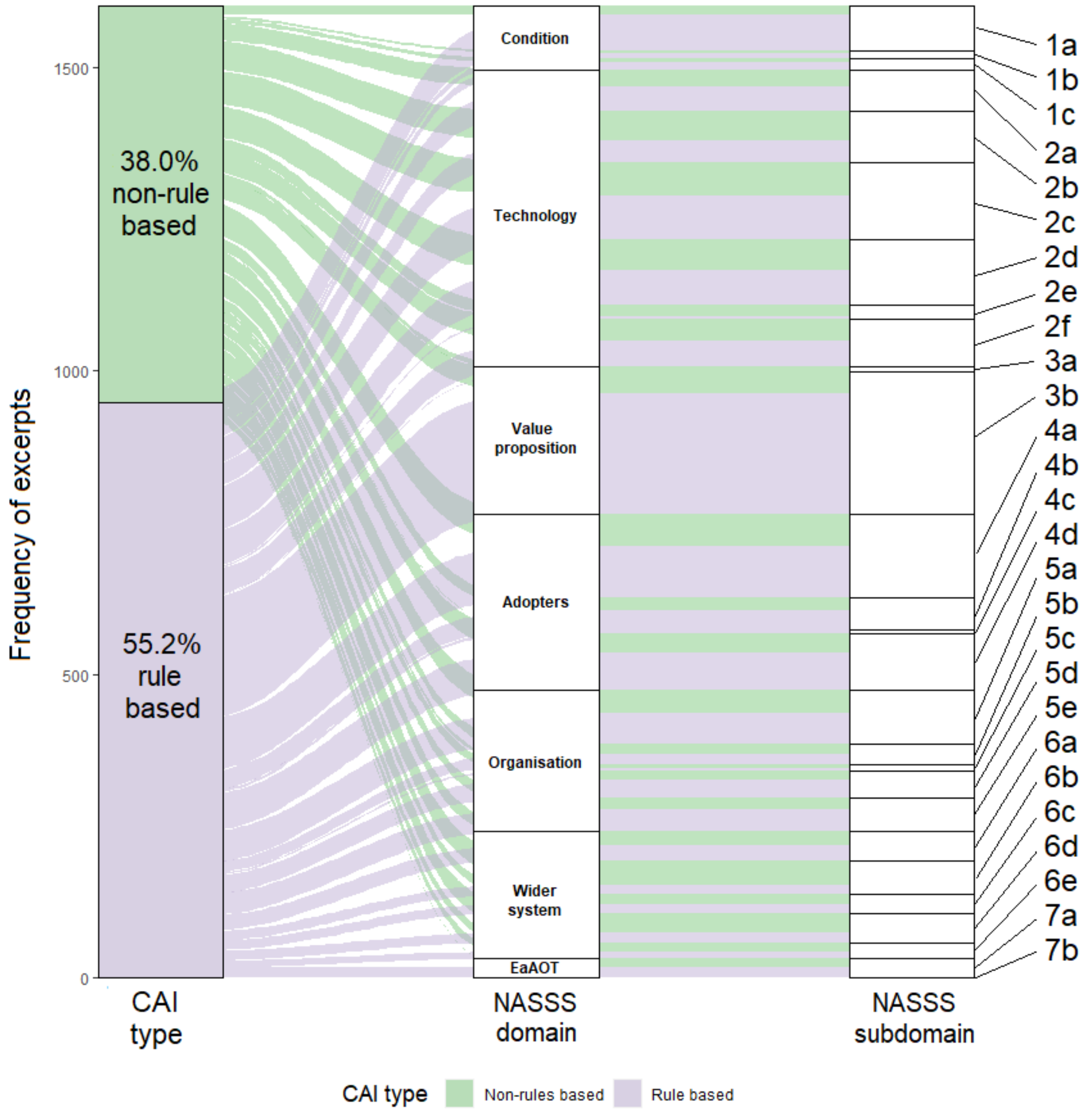

Sankey plot and accompanying table describing the relative frequency with which excerpts from eligible studies of rule-based and non-rule-based clinical artificial intelligence (CAI) relate to the various sub-domains of an adapted Non-adoption, Abandonment, Scale-up, Spread and Sustainability (NASSS) framework.

NASSS domain	NASSS sub-domain	Excerpts by NASSS sub-domain	
		Non-rule-based CAI	Rule-based CAI
**1. Condition**	1a Nature of condition or illness	21.6%	78.4%
	1b Comorbidities	30.8%	69.2%
	1c Sociocultural factors	36.8%	63.2%
**2. Technology**	2a Material properties	40.6%	59.4%
	2b Knowledge to use it	56.6%	43.4%
	2c Knowledge generated by it	44.1%	55.9%
	2d Supply model	47.2%	52.8%
	2e Who owns the intellectual property?	87.0%	13.0%
	2f Care pathway positioning	45.6%	54.4%
**3. Value proposition**	3a Supply-side value (to developer)	88.9%	11.1%
	3b Demand-side value (to patient)	15.0%	85.0%
**4. Adopters**	4a Staff (role, identity)	38.1%	61.9%
	4b Patient (simple v complex input)	38.5%	61.5%
	4c Carers	16.7%	83.3%
	4d Relationships	35.5%	64.5%
**5. Organisation**	5a Capacity to innovate in general	43.3%	56.7%
	5b Readiness for this technology	47.1%	52.9%
	5c Nature of adoption and/or funding decision	66.7%	33.3%
	5d Extent of change needed to organisational routines	34.8%	65.2%
	5e Work needed to plan, implement and monitor change	35.2%	64.8%
**6. Wider system**	6a Political/policy context	47.9%	52.1%
	6b Regulatory/legal issues	71.4%	28.6%
	6c Professional bodies	51.6%	48.4%
	6d Socio-cultural context	67.3%	32.7%
	6e Interorganisational networking	57.7%	42.3%
**7. Embedding and adaption over time**	7a Scope for adaption over time	46.7%	53.3%
	7b Organisational resilience	50.0%	50.0%
